# Author Correction: Obesity rise plateaus in developed nations and accelerates in developing nations

**DOI:** 10.1038/s41586-026-11135-w

**Published:** 2026-09-16

**Authors:** NCD Risk Factor Collaboration (NCD-RisC), Bin Zhou, Nowell H. Phelps, Agnese Galeazzi, Olivia N. O’Driscoll, James E. Bennett, Lakshya Jain, Ysé D’Ailhaud De Brisis, Ana Barradas-Pires, Fulvio Deo, Gretchen A. Stevens, Vasilis Kontis, Christopher J. Paciorek, Rodrigo M. Carrillo-Larco, Anu Mishra, Yefeng Fan, Andrea Rodriguez-Martinez, Vishwa Nath, Archie W. Rayner, Annalise Zouein, Natalie R. Evans, Jennifer L. Baker, Günther Fink, Maroje Sorić, Carlos A. Aguilar-Salinas, Ranjit Mohan Anjana, Zulfiqar A. Bhutta, Pascal Bovet, Goodarz Danaei, Kairat Davletov, Shubash Ganapathy, Edward W. Gregg, Nayu Ikeda, Andre Pascal Kengne, Young-Ho Khang, Kamlesh Khunti, Tiina Laatikainen, Avula Laxmaiah, Lee-Ling Lim, Hsien-Ho Lin, Jean Claude N. Mbanya, J. Jaime Miranda, Anja Schienkiewitz, Sylvain Sebert, Namuna Shrestha, Yi Song, Luis Adrián Soto-Mota, Gregor Starc, Limin Wang, Novie O. Younger-Coleman, Francesco Zaccardi, Julie Aarestrup, Leandra Abarca-Gómez, Mohsen Abbasi-Kangevari, Ziad A. Abdeen, Shynar Abdrakhmanova, Suhaila Abdul Ghaffar, Hanan F. Abdul Rahim, Zulfiya Abdurahmnova, Niveen M. Abu-Rmeileh, Jamila Abubakar Garba, Benjamin Acosta-Cazares, Ishag Adam, Marzena Adamczyk, Robert J. Adams, Seth Adu-Afarwuah, Wichai Aekplakorn, Tin Afifah, Kaosar Afsana, Shoaib Afzal, Imelda A. Agdeppa, Javad Aghazadeh-Attari, Åsa Ågren, Hassan Aguenaou, Charles Agyemang, Mohamad Hasnan Ahmad, Noor Ani Ahmad, Ali Ahmadi, Naser Ahmadi, Nastaran Ahmadi, Imran Ahmed, Soheir H. Ahmed, Wolfgang Ahrens, Gulmira Aitmurzaeva, Kamel Ajlouni, Dilorom Akhmedova, Nilufar Akhmedova, Nasser Al-Daghri, Sarah F. Al-Hamli, Hazzaa M. Al-Hazzaa, Halima Al-Hinai, Jawad A. Al-Lawati, Rajaa Al-Raddadi, Islam K. Al-Shami, Deena Al Asfoor, Huda M. Al Hourani, Nawal M. Al Qaoud, Monira Alarouj, Fadia AlBuhairan, Shahla AlDhukair, Maryam A. Aldwairji, Sílvia Alexius, Mohamed M. Ali, Mohammed K. Ali, Nazirah Alias, Anna V. Alieva, Abdullah Alkandari, Buthaina M. Alkhatib, Mar Alvarez-Pedrerol, Eman Aly, Deepak N. Amarapurkar, Parisa Amiri, John Amoah, Norbert Amougou, Philippe Amouyel, Atieh Amouzegar, Lars Bo Andersen, Sigmund A. Anderssen, Jonas S. Andersson, Odysseas Androutsos, Malick Anne, Alireza Ansari-Moghaddam, Elena Anufrieva, Hajer Aounallah-Skhiri, Joana Araújo, Inger Ariansen, Carmen Arias López, Tahir Aris, Raphael E. Arku, Nimmathota Arlappa, Enrique G. Artero, Krishna K. Aryal, Thor Aspelund, Felix K. Assah, Nega Assefa, Batyrbek Assembekov, Maria Cecília F. Assunção, Shiu Lun Au Yeung, May Soe Aung, Juha Auvinen, Mária Avdičová, Kishwar Azad, Ana Azevedo, Mohsen Azimi-Nezhad, Fereidoun Azizi, Bontha V. Babu, Flora Bacopoulou, Azli Baharudin, Suhad Bahijri, Borko Bajić, Izet Bajramovic, Marta Bakacs, Nagalla Balakrishna, Yulia Balanova, Mohamed Bamoshmoosh, Maciej Banach, José R. Banegas, Joanna Baran, Rafał Baran, Carlo M. Barbagallo, Valter Barbosa Filho, Alberto Barcelo, Maja Baretić, Amina Barkat, Joaquin Barnoya, Lena Barrera, Marta Barreto, Aluisio J. D. Barros, Mauro Virgílio Gomes Barros, Anna Bartosiewicz, Alicja Basiak-Rasała, Abdul Basit, Joao Luiz D. Bastos, Iqbal Bata, Anwar M. Batieha, Aline P. Batista, Rosangela L. Batista, Zhamilya Battakova, Susanne Bauer, Louise A. Baur, Pascal M. Bayauli, Robert Beaglehole, Silvia Bel-Serrat, Antonisamy Belavendra, María R. Beltran-Valls, Habiba Ben Romdhane, Theodora Benedek, Judith Benedics, Mikhail Benet, Gilda Estela Benitez Rolandi, Pedro J. Benito, Michaela Benzeval, Elling Bere, Nicolas Berger, Ingunn Holden Bergh, Yemane Berhane, Salim Berkinbayev, Antonio Bernabe-Ortiz, Gailute Bernotiene, Ximena Berrios Carrasola, Paula Berruezo, Heloísa Bettiol, Manfred E. Beutel, Augustin F. Beybey, Jorge Bezerra, Aroor Bhagyalaxmi, Sumit Bharadwaj, Santosh K. Bhargava, Hongsheng Bi, Yufang Bi, Daniel Bia, Katia Biasch, Elysée Claude Bika Lele, Mukharram M. Bikbov, Bihungum Bista, Dusko J. Bjelica, Anne A. Bjerregaard, Peter Bjerregaard, Espen Bjertness, Marius B. Bjertness, Cecilia Björkelund, Ieva Blauzde, Moran Blaychfeld Magnazi, Katia V. Bloch, Anneke Blokstra, Simona Bo, Martin Bobak, Lynne M. Boddy, Bernhard O. Boehm, Jose G. Boggia, Elena Bogova, Carlos P. Boissonnet, Stig E. Bojesen, Marialaura Bonaccio, Americo Bonanni, Vanina Bongard, Alice Bonilla-Vargas, Matthias Bopp, Elaine Borghi, Herman Borghs, Steve Botomba, Rupert Bourne, Khadichamo Boymatova, Francesca Bracone, Lien Braeckevelt, Lutgart Braeckman, Marjolijn C. E. Bragt, Tasanee Braithwaite, Imperia Brajkovich, Francesco Branca, Juergen Breckenkamp, João Breda, Hermann Brenner, Lizzy M. Brewster, Garry R. Brian, Yajaira Briceño, Lăcrămioara Brîndușe, Bettina Bringolf-Isler, Miguel Brito, Sinead Brophy, Johannes Brug, Anna Bugge, Marta Buoncristiano, Genc Burazeri, Con Burns, Antonio Cabrera de León, Joseph Cacciottolo, Cristina Cadenas-Sanchez, Hui Cai, Roberta B. Caixeta, Tilema Cama, Christine Cameron, José Camolas, Francesco Campa, Günay Can, Ana Paula C. Cândido, Felicia Cañete, Mario V. Capanzana, Naděžda Čapková, Eduardo Capuano, Rocco Capuano, Vincenzo Capuano, Marloes Cardol, Viviane C. Cardoso, Axel C. Carlsson, Esteban Carmuega, Joana Carvalho, Deborah Carvalho Malta, José A. Casajús, Felipe F. Casanueva, Jose Castro-Piñero, Ertugrul Celikcan, Laura Censi, Chiara Cerletti, Marvin Cervantes‐Loaiza, Juraci A. Cesar, Parinya Chamnan, Snehalatha Chamukuttan, Angelique W. Chan, Queenie Chan, Fadi J. Charchar, Marie-Aline Charles, Himanshu K. Chaturvedi, Nish Chaturvedi, Norsyamlina Che Abdul Rahim, Fangfang Chen, Huashuai Chen, Long-Sheng Chen, Shuohua Chen, Zhengming Chen, Ching-Yu Cheng, Yiling J. Cheng, Leila Cheraghi, Bahman Cheraghian, Angela Chetrit, Ekaterina Chikova-Iscener, Mai J. M. Chinapaw, Arnaud Chiolero, Adela Chirita-Emandi, María-Dolores Chirlaque, Chean Lin Chong, Kaare Christensen, Diego G. Christofaro, Jerzy Chudek, Silvia Ciardullo, Renata Cifkova, Michelle Cilia, Eliza Cinteza, Massimo Cirillo, Frank Claessens, Maria Clapperton, Philip Clare, Janine Clarke, Svetlana Cociu, Emmanuel Cohen, Sandra Colorado-Yohar, Laura-María Compañ-Gabucio, Hans Concin, Susana C. Confortin, Cyrus Cooper, Tara C. Coppinger, Lorraine S. Cordeiro, Eva Corpeleijn, Lilia Yadira Cortés, Cojocaru R. Cosmin, Simona Costanzo, Melanie J. Cowan, Chris Cowell, Cora L. Craig, Amelia C. Crampin, Haddy Crookes, Amanda J. Cross, Sarah Crozier, Ana B. Crujeiras, Juan J. Cruz, Tamás Csányi, Semánová Csilla, Alexandra M. Cucu, Liufu Cui, Felipe V. Cureau, Sarah Cuschieri, Ewelina Czenczek-Lewandowska, Graziella D’Arrigo, Eleonora d’Orsi, Haroldo da Silva-Ferreira, Alanna G. da Silva, Liliana Dacica, Tukur Dahiru, Christina C. Dahm, María Ángeles Dal Re Saavedra, Jean Dallongeville, Albertino Damasceno, Camilla T. Damsgaard, Maryam S. Daneshpour, Rachel Dankner, Parasmani Dasgupta, Saeed Dastgiri, Luc Dauchet, Afonso de Almeida, Francisco de Assis Guedes de Vasconcelos, Maria Alice Altenburg de Assis, Guy De Backer, Dirk De Bacquer, Jaco De Bacquer, Jeroen de Bont, Luigi G. De Filippis, Patrícia de Fragas Hinnig, Stefaan De Henauw, Pilar De Miguel-Etayo, Jan-Walter De Neve, Paula Duarte de Oliveira, Luz Maria De Regil, David De Ridder, Karin De Ridder, Susanne R. de Rooij, Ana Carolina M. G. N. de Sá, Delphine De Smedt, Thomas V. de Souza, Marco A. de Valois Correia Júnior, George Dedoussis, Mohan Deepa, Alexander D. Deev, Vincent DeGennaro, Francis Delpeuch, Stefaan Demarest, Elaine Dennison, Katarzyna Dereń, Valérie Deschamps, Ruslan D. Devrishov, Meghnath Dhimal, Juvenal Soares Dias-da-Costa, Alejandro Diaz, María Elena Díaz-Sánchez, Francisco Diez-Canseco, Zivka Dika, Shirin Djalalinia, Visnja Djordjic, Ha T. P. Do, Annette J. Dobson, Liria Dominguez, Maria Benedetta Donati, Chiara Donfrancesco, Guanghui Dong, Li Dong, Yanhui Dong, Silvana P. Donoso, Cecilia Dorado-García, Angela Döring, Maria Dorobantu, Ahmad Reza Dorosty, Marcus Dörr, Nico Dragano, Wojciech Drygas, Shufa Du, Jia Li Duan, Charmaine A. Duante, Priscilla Duboz, Marina Duishenkulova, Vesselka L. Duleva, Virginija Dulskiene, Samuel C. Dumith, Anar Dushpanova, Terence Dwyer, Azhar Dyussupova, Vilnis Dzerve, Elzbieta Dziankowska-Zaborszczyk, Anna Dzielska, Narges Ebrahimi, Guadalupe Echeverría, Ricky Eddie, Ebrahim Eftekhar, Vasiliki Efthymiou, Eruke E. Egbagbe, Robert Eggertsen, Sareh Eghtesad, Gabriele Eiben, Ulf Ekelund, Mohammad El-Khateeb, Laila El Ammari, Jalila El Ati, Denise Eldemire-Shearer, Paul Elliott, Ofem Enang, Ronit Endevelt, Reina Engle-Stone, Jonas Englund, Rajiv T. Erasmus, Cihangir Erem, Gul Ergor, Louise Eriksen, Johan G. Eriksson, Jorge Escobedo-de la Peña, Ali Esmaeili, Vanesa España-Romero, Alun Evans, Roger G. Evans, David Faeh, Guy Fagherazzi, Noushin Fahimfar, Ildar Fakhradiyev, Albina A. Fakhretdinova, Caroline H. Fall, Elnaz Faramarzi, Mojtaba Farjam, Victoria Farrugia Sant’Angelo, Farshad Farzadfar, Yosef Farzi, Mohammad Reza Fattahi, Asher Fawwad, Wafaie W. Fawzi, Rosemarie Felder-Puig, Francisco J. Felix-Redondo, Trevor S. Ferguson, Romulo A. Fernandes, Daniel Fernández-Bergés, Desha R. Fernando, Rashida A. Ferrand, Daniel Ferrante, Thomas Ferrao, Gerson Ferrari, Marika Ferrari, Marco M. Ferrario, Catterina Ferreccio, Eldridge Ferrer, Jean Ferrieres, Thamara Hubler Figueiró, Anna Fijalkowska, Mauro Fisberg, Krista Fischer, Malang N. Fofana, Maria Forsner, Edward F. Fottrell, Heba M. Fouad, Damian K. Francis, Maria do Carmo Franco, Zlatko Fras, Brooklyn Fraser, Guillermo Frontera, Flavio D. Fuchs, Sandra C. Fuchs, Yuki Fujita, Matsuda Fumihiko, Viktoriya Furdela, Takuro Furusawa, Zbigniew Gaciong, Lutfi Gafarov, Mihai Gafencu, Sonya V. Galcheva, Henrike Galenkamp, Daniela Galeone, Myriam Galfo, Fabio Galvano, Jingli Gao, Chandalene Garabwan, Natalia García-Corada, Manoli Garcia-de-la-Hera, Marta García Solano, Dickman Gareta, Sarah P. Garnett, Jean-Michel Gaspoz, Magda Gasull, Victoria Gauthier, Adroaldo Cesar Araujo Gaya, Anelise Reis Gaya, Andrea Gazzinelli, Ulrike Gehring, Johanna M. Geleijnse, Ronnie George, Eva Gerdts, Ibrahim D. Gezawa, Ebrahim Ghaderi, Seyyed-Hadi Ghamari, Ali Ghanbari, Asghar Ghasemi, Erfan Ghasemi, Hala Ghattas, Oana-Florentina Gheorghe-Fronea, Simona Giampaoli, Francesco Gianfagna, Christian Gieger, Tiffany K. Gill, Ntombifuthi Ginindza, Jonathan Giovannelli, Glen Gironella, Aleksander Giwercman, Konstantinos Gkiouras, Natalya Glushkova, Ramesh Godara, Keith M. Godfrey, Justyna Godos, Sibel Gogen, Marcel Goldberg, David Goltzman, Georgina Gómez, Luis F. Gomez, Santiago F. Gómez, Aleksandra Gomula, Bruna Gonçalves Cordeiro da Silva, Helen Gonçalves, Mauer Gonçalves, Ana D. González-Alvarez, David A. Gonzalez-Chica, Esther M. González-Gil, Marcela Gonzalez-Gross, Margot González-Leon, Juan P. González-Rivas, Angel R. Gonzalez, Frederic Gottrand, Antonio Pedro Graça, Dušan Grafnetter, Aneta Grajda, Maria G. Grammatikopoulou, Andriene Grant, Ronald D. Gregor, Maria João Gregório, Anne Sameline Grimsgaard, Else Karin Grøholt, Anders Grøntved, Giuseppe Grosso, Dongfeng Gu, Viviana Guajardo, Emanuela Gualdi-Russo, Pilar Guallar-Castillón, Elias F. Gudmundsson, Vilmundur Gudnason, Maëlenn Guerchet, Ramiro Guerrero, Idris Guessous, Andre L. Guimaraes, Unjali P. Gujral, Martin C. Gulliford, Johanna Gunnlaugsdottir, Marc J. Gunter, Xiu-Hua Guo, Yin Guo, Prakash C. Gupta, Preeti Gupta, Rajeev Gupta, Oye Gureje, Mirjana A. Gurinović, Enrique Gutiérrez González, Laura Gutierrez, Felix Gutzwiller, Mònica Guxens, Xinyi Gwee, Seongjun Ha, Farzad Hadaegh, Charalambos A. Hadjigeorgiou, Rosa Haghshenas, Gahraman Hagverdiyev, Hamid Hakimi, Jytte Halkjær, Sameh S. Hallaq, Ian R. Hambleton, Behrooz Hamzeh, Dominique Hange, Abu A. M. Hanif, Sari Hantunen, Jie Hao, Carla Menêses Hardman, Louise Hardy, Tina Harmer Lassen, Javad Harooni, Seyed Mohammad Hashemi-Shahri, Mitra Hasheminia, Maria Hassapidou, Jun Hata, Teresa Haugsgjerd, Chika Hayashi, Alison J. Hayes, Jiang He, Yuan He, Yuna He, Mehdi Hedayati, Regina Heidinger-Felső, Margit Heier, Mirjam Heinen, Tatjana Hejgaard, Marleen Elisabeth Hendriks, Rafael dos Santos Henrique, Ana Henriques, Leticia Hernandez Cadena, Sauli Herrala, Marianella Herrera-Cuenca, Victor M. Herrera, Isabelle Herter-Aeberli, Karl-Heinz Herzig, Ramin Heshmat, Barbara Heude, Allan G. Hill, Sai Yin Ho, Michael Hobbs, Doroteia A. Höfelmann, Michelle Holdsworth, Reza Homayounfar, Clara Homs, Emiel O. Hoogendijk, Wilma M. Hopman, Andrea R. V. R. Horimoto, Claudia M. Hormiga, Bernardo L. Horta, Farhad Hosseinpanah, Leila Houti, Christina Howitt, Thein Thein Htay, Aung Soe Htet, Maung Maung Than Htike, Yonghua Hu, José María Huerta, Ilpo Tapani Huhtaniemi, Laetitia Huiart, Constanta Huidumac Petrescu, Martijn Huisman, Abdullatif S. Husseini, Chinh Nguyen Huu, Inge Huybrechts, Nahla Hwalla, Jolanda Hyska, Licia Iacoviello, Ellina M. Iakupova, Jesús M. Ibarluzea, Norazizah Ibrahim Wong, Jannicke Igland, Chinwuba Ijoma, Edolem Ikerdeu, M. Arfan Ikram, Carmen Iñiguez, Violeta Iotova, Maybelline Joy B. Ipil, Vilma E. Irazola, Takafumi Ishida, Godsent C. Isiguzo, Muhammad Islam, Sheikh Mohammed Shariful Islam, Duygu Islek, Ivaila Y. Ivanova-Pandourska, Masanori Iwasaki, Tuija Jääskeläinen, Rod T. Jackson, Jeremy M. Jacobs, Michel Jadoul, Tazeen H. Jafar, Kenneth James, Konrad Jamrozik, Nataša Jan, Anna Jansson, Imre Janszky, Edward Janus, Juel Jarani, Gerald Jarnig, Marjo-Riitta Jarvelin, Grazyna Jasienska, Ana Jelaković, Bojan Jelaković, Garry Jennings, A. M. Jibo, Ramon O. Jimenez, Karl-Heinz Jöckel, Michel Joffres, Jari J. Jokelainen, Jost B. Jonas, Lars Jøran Kjerpeseth, Torben Jørgensen, Rohina Joshi, Josipa Josipović, Farahnaz Joukar, Pekka Jousilahti, Jacek J. Jóźwiak, Debra S. Judge, Anne Juolevi, Gregor Jurak, Iulia Jurca Simina, Vesna Juresa, Rudolf Kaaks, Niina E. Kaartinen, Felix O. Kaducu, Agnes L. Kadvan, Anthony Kafatos, Maria Kafyra, Mónika Kaj, Eero O. Kajantie, Sree Ramakrishna Kakani, Bernard Kakuhikire, Natia Kakutia, Daniela Kállayová, Zhanna Kalmatayeva, Natasa Kalpourtzi, Ofra Kalter-Leibovici, Yves Kameli, Kodanda R. Kanala, Srinivasan Kannan, Efthymios Kapantais, Anna Kapustina, Eva Karaglani, Line L. Kårhus, Khem B. Karki, Omat Karlsson, Adoubi Kassi Anicet, Philippe B. Katchunga, Marzieh Katibeh, Prasad Katulanda, Joanne Katz, Peter T. Katzmarzyk, Jussi Kauhanen, Prabhdeep Kaur, Maryam Kavousi, Gyulli M. Kazakbaeva, François F. Kaze, Benson M. Kazembe, Calvin Ke, Youzhi Ke, Ulrich Keil, Lital Keinan Boker, Sirkka M. Keinänen-Kiukaanniemi, Roya Kelishadi, Cecily Kelleher, Han C. G. Kemper, Maryam Keramati, Mathilde Kersting, Bobby Kgosiemang, Yousef Saleh Khader, Kazem Khalagi, Arsalan Khaledifar, Davood Khalili, Bahareh Kheiri, Motahareh Kheradmand, Irina V. Khorosheva, Alireza Khosravi Farsani, Ilse M. S. L. Khouw, Saeed Khwaja Mir Islam, Ursula Kiechl-Kohlendorfer, Stefan Kiechl, Japhet Killewo, Hyeon Chang Kim, Jenny M. Kindblom, Heidi Klakk, Suntara Klanarong, Jana Klanova, Magdalena Klimek, Jurate Klumbiene, Michael Knoflach, Susanne Kobel, Maciej Kochman, Bhawesh Koirala, Sweta Koirala, Elin Kolle, Sanda M. Kolo, Patrick Kolsteren, Jürgen König, Päivikki Koponen, Raija Korpelainen, Paul Korrovits, Magdalena Korzycka, Jelena Kos, Seppo Koskinen, Katsuyasu Kouda, Malik Koussoh Simone, Éva Kovács, Viktoria Anna Kovacs, Irina Kovalskys, Sudhir Kowlessur, Slawomir Koziel, Jana Kratenova, Wolfgang Kratzer, Vilma Kriaucioniene, Susi Kriemler, Peter Lund Kristensen, Helena Krizan, Maria F. Kroker-Lobos, Steinar Krokstad, Daan Kromhout, Herculina S. Kruger, Ruan Kruger, Łukasz Kryst, Ruzena Kubinova, Renata Kuciene, Urho M. Kujala, Enisa Kujundzic, Zbigniew Kulaga, Mukhtar Kulimbet, Vaitheeswaran Kulothungan, Richard Kumapley, R. Krishna Kumar, Meena Kumari, Marie Kunešová, Yadlapalli S. Kusuma, Vladimir Kutsenko, Kari Kuulasmaa, Catherine Kyobutungi, Quang Ngoc La, Fatima Zahra Laamiri, Demetre Labadarios, Idoia Labayen, Carl Lachat, Karl J. Lackner, Jouni Lahti, Daphne Lai, Wai Kent Lai, Youcef Laid, Lachmie Lall, Ecosse L. Lamoureux, Maritza Landaeta Jimenez, Edwige Landais, Anne Langsted, Tiina Lankila, Vera Lanska, Georg Lappas, Bagher Larijani, Mina P. Lateva, Tint Swe Latt, Martino Laurenzi, María Lazo-Porras, Gwenaëlle Le Coroller, Khanh Le Nguyen Bao, Agnès Le Port, Tuyen D. Le, Jeannette Lee, Paul H. Lee, Terho Lehtimäki, Daniel Lemogoum, David A. Leon, Elvynna Leong, Aitana Lertxundi, Nerea Lertxundi, Branimir Leskošek, Justyna Leszczak, Katja B. Leth-Møller, Gabriel M. Leung, Esko Levälahti, Sergey P. Levushkin, Yanping Li, Merike Liivak, Christa L. Lilly, Charlie Lim, Wei-Yen Lim, Maria Fernanda Lima-Costa, Yi-Jing Lin, Lars Lind, Vijaya Lingam, Birgit Linkohr, Allan Linneberg, Jakob Linseisen, Lauren Lissner, Mieczyslaw Litwin, Jing Liu, Lijuan Liu, Liping Liu, Xiaotian Liu, Yang Liu, Sabrina Llop, Wei-Cheng Lo, Helle-Mai Loit, Ayesha Lokubalasooriya, Khuong Quynh Long, Carla Lopes, Luis Lopes, Marcus V. V. Lopes, Oscar Lopes, Esther Lopez-Garcia, José Francisco López-Gil, Tania Lopez, Paulo A. Lotufo, José-Eugenio Lozano-Alonso, Gabriel Lozano-Berges, Janice L. Lukrafka, Dalia Luksiene, María Delia Luna, Annamari Lundqvist, Nuno Lunet, Charles Lunogelo, Michala Lustigová, Edyta Łuszczki, Jean-René M’Buyamba-Kabangu, Guansheng Ma, Jun Ma, Xu Ma, George L. L. Machado-Coelho, Aristides M. Machado-Rodrigues, Enguerran Macia, Luisa M. Macieira, Ahmed A. Madar, Sherilynn Madraisau, Anja L. Madsen, Gladys E. Maestre, Stefania Maggi, Dianna J. Magliano, Emmanuella Magriplis, Gowri Mahasampath, Bernard Maire, Marjeta Majer, Marcia Makdisse, Päivi Mäki, Mohammad-Reza Malekpour, Fatemeh Malekzadeh, Reza Malekzadeh, Rahul Malhotra, Laurent Malisoux, Sofia K. Malyutina, Lynell V. Maniego, Yannis Manios, Jim I. Mann, Satu Männistö, Fariborz Mansour-Ghanaei, Taru Manyanga, Enzo Manzato, Mala Ali Mapatano, Anie Marcil, Francisco Mardones, Paula Margozzini, Joany Mariño, Mihaela Marinović Glavić, Anastasia Markaki, Oonagh Markey, Josko Markic, Eliza Markidou Ioannidou, Pedro Marques-Vidal, Larissa Pruner Marques, Jaume Marrugat, Dries Martens, Yves Martin-Prevel, Rosemarie Martin, Borja Martinez-Tellez, Vicente Martínez-Vizcaíno, Reynaldo Martorell, Eva Martos, Fatai A. Maruf, Katharina Maruszczak, Stefano Marventano, Giovanna Masala, Luis P. Mascarenhas, Mannix Masimango Imani, Masoud Masinaei, Ellisiv B. Mathiesen, Prashant Mathur, Alicia Matijasevich, Piotr Matłosz, Tandi E. Matsha, Victor Matsudo, Giletta Matteo, Pallab K. Maulik, Christina Mavrogianni, Artur Mazur, Camille M. Mba, Shelly R. McFarlane, Stephen T. McGarvey, Keeley McGee, Martin McKee, Rachael M. McLean, Scott B. McLean, Breige A. McNulty, Sounnia Mediene Benchekor, Jurate Medzioniene, Kirsten Mehlig, Vrinda Mehra, Amir Houshang Mehrparvar, Jørgen Meisfjord, Christine Meisinger, Jesus D. Melgarejo, Marina Melkumova, Júlio B. Mello, Sofia Mendes, Fabián Méndez, Carlos O. Mendivil, Ana Maria B. Menezes, Geetha R. Menon, Gert B. M. Mensink, Maria Teresa Menzano, Indrapal I. Meshram, Diane T. Meto, Haakon E. Meyer, Jie Mi, Kim F. Michaelsen, Nathalie Michels, Kairit Mikkel, Jelena P. Milešević, Jody C. Miller, Olga Milushkina, Cláudia S. Minderico, G. K. Mini, Juan Francisco Miquel, Mohammad Reza Mirjalili, Daphne Mirkopoulou, Parvin Mirmiran, Masoud Mirzaei, Marjeta Mišigoj-Duraković, Antonio Mistretta, Veronica Mocanu, Ana Mocumbi, Pietro A. Modesti, Jobe Modou, Sahar Saeedi Moghaddam, Shukri F. Mohamed, Kazem Mohammad, Mohammad Reza Mohammadi, Zahra Mohammadi, Noushin Mohammadifard, Viswanathan Mohan, Sherina Mohd Sidik, Muhammad Fadhli Mohd Yusoff, Iraj Mohebbi, Diego Moliner Urdiales, Line T. Møllehave, Niels C. Møller, Dénes Molnár, Amirabbas Momenan, Charles K. Mondo, Rafael Monge-Rojas, Michele M. Monroy-Valle, Roger A. Montenegro Mendoza, Eric Monterrubio-Flores, Kotsedi Daniel K. Monyeki, Jin Soo Moon, Mahmood Moosazadeh, Hermine T. Mopa, Farhad Moradpour, Leila B. Moreira, Alain Morejon, Luis A. Moreno, Francis Morey, Karen Morgan, Suzanne N. Morin, Erik Lykke Mortensen, George Moschonis, Alireza Moslem, Mildrey Mosquera, Malgorzata Mossakowska, Aya Mostafa, Seyed-Ali Mostafavi, Anabela Mota-Pinto, Eugen Mota, Jorge Mota, Maria Mota, Mohammad Esmaeel Motlagh, Jorge Motta, Robert Moumakwa, Marcos André Moura-dos-Santos, Yeva Movsesyan, Malay K. Mridha, Kelias P. Msyamboza, Alicia Mtijasevich, Thet Thet Mu, Magdalena Muc, Florian Muca, Boban Mugoša, Maria L. Muiesan, Martina Müller-Nurasyid, Patricia B. Munroe, Adrià Muntaner-Mas, Thomas Münzel, Molly M. Murphy, Celine Murrin, Jaakko Mursu, Elaine M. Murtagh, Kamarul Imran Musa, Sanja Musić Milanović, Vera Musil, Geofrey Musinguzi, Muel Telo M. C. Muyer, Iraj Nabipour, Gabriele Nagel, Farid Najafi, Harunobu Nakamura, Hanna Nalecz, Jana Námešná, Ei Ei K. Nang, Vinay B. Nangia, Martin Nankap, Sameer Narake, K. M. Venkat Narayan, Paola Nardone, Take Naseri, Tim Nawrot, William A. Neal, Nareemarn Neelapaichit, Mayssam Nehme, Azim Nejatizadeh, Ilona Nenko, Martin Neovius, Flavio Nervi, Olena Nesterova, Dinesh Neupane, Tze Pin Ng, Chung T. Nguyen, Nguyen D. Nguyen, Quang Ngoc Nguyen, Michael Y. Ni, Rodica Nicolescu, Peng Nie, Ramfis E. Nieto-Martínez, Yury P. Nikitin, Guang Ning, Toshiharu Ninomiya, Nobuo Nishi, Sania Nishtar, Marianna Noale, Oscar A. Noboa, Helena Nogueira, Maria Nordendahl, Børge G. Nordestgaard, Kevin I. Norton, Davide Noto, Natalia Nowak-Szczepanska, Mohannad Al Nsour, Irfan Nuhoğlu, Eha Nurk, Fred Nuwaha, Moffat Nyirenda, Terence W. O’Neill, Dermot O’Reilly, Galina Obreja, Caleb Ochimana, Angélica M. Ochoa-Avilés, Eiji Oda, Augustine N. Odili, Kyungwon Oh, Kumiko Ohara, Claes Ohlsson, Ryutaro Ohtsuka, Örn Olafsson, Brian Oldenburg, Maria Teresa A. Olinto, Isabel O. Oliveira, Mohd Azahadi Omar, Saeed M. Omar, Altan Onat, Sok King Ong, N. Charlotte Onland-Moret, Lariane M. Ono, Obinna Onodugo, Pedro Ordunez, Rui Ornelas, Francisco B. Ortega, Ana P. Ortiz, Pedro J. Ortiz, Merete Osler, Clive Osmond, Sergej M. Ostojic, Afshin Ostovar, Johanna A. Otero, Charlotte B. Ottendahl, Akaninyene Otu, Kim Overvad, Ellis Owusu-Dabo, Adetoyeje Y. Oyeyemi, Adewale L. Oyeyemi, Fred Michel Paccaud, Cristina P. Padez, Ioannis Pagkalos, Marat Pahimov, Elena Pahomova, Karina Mary de Paiva, Andrzej Pająk, Natalja Pajula, Alberto Palloni, Luigi Palmieri, Demosthenes Panagiotakos, Songhomitra Panda-Jonas, Arvind Pandey, Zengchang Pang, Francesco Panza, Antonio Paoli, Mariela Paoli, Sousana K. Papadopoulou, Dimitrios Papandreou, Rossina G. Pareja, Suvi Parikka, Soon-Woo Park, Suyeon Park, Winsome R. Parnell, Mahboubeh Parsaeian, Ionela M. Pascanu, Patrick Pasquet, Chona F. Patalen, Roengrudee Patanavanich, Nikhil D. Patel, Marcos Pattussi, Halyna Pavlyshyn, Raimund Pechlaner, Ivan Pećin, Dorthe C. Pedersen, Mangesh S. Pednekar, João M. Pedro, Ana B. Peinado, Sergio Viana Peixoto, Markku Peltonen, Guillem Pera, Alexandre C. Pereira, Marco A. Peres, Napoleon Perez-Farinos, Agustín Perez-Londoño, Cynthia M. Pérez, Markus Perola, Valentina Peterkova, Annette Peters, Janina Petkeviciene, Ausra Petrauskiene, Olga Petrovna Kovtun, Emanuela Pettenuzzo, Niloofar Peykari, Norbert Pfeiffer, Son Thai Pham, Felix P. Phiri, Rafael N. Pichardo, Preux Pierre-Marie, Iris Pigeot, Hynek Pikhart, Aida Pilav, Pavel Piler, Lorenza Pilotto, Francesco Pistelli, Freda Pitakaka, Aleksandra Piwonska, Andreia N. Pizarro, Pedro Plans-Rubió, Alina G. Platonova, Bee Koon Poh, Hermann Pohlabeln, Raluca M. Pop, Barry M. Popkin, Stevo R. Popovic, Miquel Porta, Georg Posch, Anil Poudyal, Dimitrios Poulimeneas, Hamed Pouraram, Farhad Pourfarzi, Akram Pourshams, Hossein Poustchi, Dorairaj Prabhakaran, Rajendra Pradeepa, Andrew Prentice, Alison J. Price, Jacqueline F. Price, Antonio Prista, Rui Providencia, Jardena J. Puder, Iveta Pudule, Soile Puhakka, Maria Puiu, Margus Punab, Muhammed S. Qadir, Radwan F. Qasrawi, Qing Qiao, Mostafa Qorbani, Anna Quialheiro, Hedley K. Quintana, Pedro J. Quiroga-Padilla, Tran Quoc Bao, Stefan Rach, Maria-Victoria Racu, Ivana Radic, Ricardas Radisauskas, Salar Rahimikazerooni, Mahfuzar Rahman, Mahmudur Rahman, Olli Raitakari, Manu Raj, Tamerlan Rajabov, Sherali Rakhmatulloev, Ivo Rakovac, Sudha Ramachandra Rao, Ambady Ramachandran, Otim P. C. Ramadan, Virgílio V. Ramires, Manuel Ramirez-Zea, Jacqueline Ramke, Elisabete Ramos, Rafel Ramos, Lekhraj Rampal, Sanjay Rampal, Sheena E. Ramsay, João F. L. B. Rangel Junior, Lalka S. Rangelova, Harish Ranjani, Ravindra P. Rannan-Eliya, João F. Raposo, Patricia Rarau, Vayia Rarra, Ramon A. Rascon-Pacheco, Mohammad-Mahdi Rashidi, Cassiano Ricardo Rech, Cristina Recuero Carretero, Josep Redon, Valéria Regecová, Jane D. P. Renner, Judit A. Repasy, Cézane P. Reuter, Luis Revilla, Andrew Reynolds, Negar Rezaei, Abbas Rezaianzadeh, Yeunsook Rho, Lourdes Ribas-Barba, Robespierre Ribeiro, Rogério T. Ribeiro, Elio Riboli, Fernando Rigo, Attilio Rigotti, Leanne M. Riley, Natascia Rinaldo, Tobias F. Rinke de Wit, Ulf Risérus, Ana I. Rito, Raphael M. Ritti-Dias, Juan A. Rivera, Reina G. Roa, Romana Roccaldo, Daniela Rodrigues, Fernando Rodríguez-Artalejo, Manuel A. Rodríguez-Pérez, María del Cristo Rodriguez-Perez, Laura A. Rodríguez-Villamizar, Andrea Y. Rodríguez, Ulla Roggenbuck, Peter Rohloff, Fabian Rohner, Rosalba Rojas-Martinez, Gemma Rojo-Martínez, Nipa Rojroongwasinkul, Almudena Rollán Gordo, Dora Romaguera, Elisabetta L. Romeo, Gil B. Rosa, Rafaela V. Rosario, Annika Rosengren, Ian Rouse, Vanessa Rouzier, Joel G. R. Roy, Maira Ruano Estrada, Maira H. Ruano, Adolfo Rubinstein, Frank J. Rühli, Jean-Bernard Ruidavets, Blanca Sandra Ruiz-Betancourt, Maria Ruiz-Castell, Emma Ruiz Moreno, Iuliia A. Rusakova, Wojciech Rusek, Kenisha Russell Jonsson, Paola Russo, Petra Rust, Marcin Rutkowski, Katri Sääksjärvi, Marge Saamel, Crizian G. Saar, Charumathi Sabanayagam, Kalpana Sabapathy, Hamideh Sabbaghi, Shaun Sabico, Harshpal S. Sachdev, Alireza Sadjadi, Ali Reza Safarpour, Sare Safi, Mohammad Hossien Saghi, Olfa Saidi, Calogero Saieva, Satoko Sakata, Nader Saki, Sanja Šalaj, Benoit Salanave, Eduardo Salazar Martinez, Akkumis Salkhanova, Diego Salmerón, Veikko Salomaa, Jukka T. Salonen, Massimo Salvetti, Margarita Samoutian, Jose Sánchez-Abanto, Guillermo Sanchez-Delgado, Mairena Sánchez-López, Joaquin Sanchis-Moysi, Sandjaja, Susana Sans, Loreto Santa-Marina, Ethel Santacruz Lezcano, Diana A. Santos, Ina S. Santos, Lèlita C. Santos, Maria Paula Santos, Osvaldo Santos, Palmira Santos, Rute Santos, Tamara R. Santos, Vonthanak Saphonn, Jouko L. Saramies, Luis B. Sardinha, Nizal Sarrafzadegan, Yoko Sato, Kai-Uwe Saum, Stefan Savin, Savvas Savva, Mathilde Savy, Norie Sawada, Mariana Sbaraini, Marcia Scazufca, Beatriz D. Schaan, Angelika Schaffrath Rosario, Herman Schargrodsky, Karin Schindler, Amand Floriaan Schmidt, Börge Schmidt, Carsten O. Schmidt, Andrea Schneider, Peter Schnohr, Catherine Mary Schooling, Ben Schöttker, Sara Schramm, Stine Schramm, Helmut Schröder, Constance Schultsz, Gry Schultz, Matthias B. Schulze, Aletta E. Schutte, Moslem Sedaghattalab, Rusidah Selamat, Abhijit Sen, Idowu O. Senbanjo, Sadaf G. Sepanlou, Guillermo Sequera, Luis Serra-Majem, Jennifer Servais, Ľudmila Ševčíková, Ronel Sewpaul, Svetlana A. Shalnova, Teresa Shamah-Levy, Seyed Morteza Shamshirgaran, Shubash Shander, Coimbatore Subramaniam Shanthirani, Maryam Sharafkhah, Sanjib K. Sharma, Almaz Sharman, Jonathan E. Shaw, Amaneh Shayanrad, Ali Akbar Shayesteh, Nurzhamal Sheisheeva, Ching-Fen Shen, Lela Shengelia, Kenji Shibuya, Hana Shimizu-Furusawa, Tal Shimony, Avi Shina, Igor D. Shkrobanets, Marat Shoranov, Khairil Si-Ramlee, Alfonso Siani, Abla M. Sibai, Labros S. Sidossis, Mark J. Siedner, Natalia Silitrari, Antonio M. Silva, Caroline Ramos de Moura Silva, Diego Augusto Santos Silva, Kelly Samara Silva, Xueling Sim, Mary Simon, Judith Simons, Leon A. Simons, Ivan Simunovic, Agneta Sjöberg, Michael Sjöström, Elena V. Skoblina, Natalia A. Skoblina, Tatyana Slazhnyova, Jolanta Slowikowska-Hilczer, Przemysław Slusarczyk, Liam Smeeth, Lee Smith, Hung-Kwan So, Fernanda Cunha Soares, Grzegorz Sobek, Eugène Sobngwi, Morten Sodemann, Stefan Söderberg, Moesijanti Y. E. Soekatri, Agustinus Soemantri, Raquel Soler-Blasco, Vincenzo Solfrizzi, Yuliya V. Solovieva, Mohammad Hossein Somi, Emily Sonestedt, Sajid Soofi, Thorkild I. A. Sørensen, Elin P. Sørgjerd, Victoria E. Soto-Rojas, Aïcha Soumaré, Alfonso Sousa-Poza, Mafalda Sousa-Uva, Mam Sovatha, Agnieszka Sozańska, Bente Sparboe-Nilsen, Karen Sparrenberger, Vita Speckauskiene, Phoebe R. Spencer, Angela Spinelli, Igor Spiroski, Jan A. Staessen, Aleksandra Stamenova, Hanspeter Stamm, Laura Stanciulescu, Andreas Stang, Kaspar Staub, Bill Stavreski, Jostein Steene-Johannessen, Peter Stehle, Aryeh D. Stein, Silje Steinsbekk, George S. Stergiou, Jochanan Stessman, Jutta Stieber, Doris Stöckl, Jakub Stokwiszewski, Katarzyna Stoś, Ekaterina Stoyanova, Gareth Stratton, Karien Stronks, Maria Wany Strufaldi, Lela Sturua, Ramón Suárez-Medina, Milton F. Suarez-Ortegón, Phalakorn Suebsamran, Mindy S. Sugiyama, Machi Suka, Gerhard Sulo, Malin Sund, Johan Sundström, Yn-Tz Sung, Jordi Sunyer, Suparmi, Unursaikhan Surenjav, Paibul Suriyawongpaisal, Kitti Susovits, Nabil William G. Sweis, Boyd A. Swinburn, René Charles Sylva, Unni Syversen, Lucjan Szponar, Yasuharu Tabara, Lorraine Tabone, E. Shyong Tai, Konstantinos D. Tambalis, Mari-Liis Tammesoo, Abdonas Tamosiunas, Eng Joo Tan, Baimakhan Tanabayev, Nikhil Tandon, Xun Tang, Maya Tanrygulyyeva, Frank Tanser, Yong Tao, Mohammed Rasoul Tarawneh, Jakob Tarp, Carolina B. Tarqui-Mamani, Radka Taxová Braunerová, Anne Taylor, Félicité Tchibindat, Saskia Te Velde, William R. Tebar, Fahimeh R. Tehrani, Grethe S. Tell, Tania Tello, Masresha Tessema, Lukas Teufl, Yih Chung Tham, K. R. Thankappan, Holger Theobald, Xenophon Theodoridis, Sathish Thirunavukkarasu, Nihal Thomas, Barbara Thorand, Amanda G. Thrift, Ľubica Tichá, Erik J. Timmermans, Dwi Hapsari Tjandrarini, Anne Tjonneland, Ervin Toçi, Maryam Tohidi, Hanna K. Tolonen, Janne S. Tolstrup, Maciej Tomaszewski, Murat Topbas, Roman Topór-Mądry, Pere Torán-Monserrat, Liv Elin Torheim, Michael J. Tornaritis, Maties Torrent, Laura Torres-Collado, Duarte Torres, Silvia Torres, Stefania Toselli, Giota Touloumi, Luciana Tovo-Rodrigues, Pierre Traissac, Thi Tuyet-Hanh Tran, Mark S. Tremblay, Areti Triantafyllou, Antonia Trichopoulou, Oanh T. H. Trinh, Justina Trišauskė, Atul Trivedi, Alexander C. Tsai, Lechaba Tshepo, Thomas Tsiampalis, Maria Tsigga, Panagiotis Tsintavis, Shoichiro Tsugane, John Tuitele, Azaliia M. Tuliakova, Marshall K. Tulloch-Reid, Fikru Tullu, Tomi-Pekka Tuomainen, Jaakko Tuomilehto, Maria L. Turley, Gilad Twig, Per Tynelius, Evangelia Tzala, Themistoklis Tzotzas, Christophe Tzourio, Nwannedimma Udoji, Peter Ueda, Eunice Ugel, Flora A. M. Ukoli, Hanno Ulmer, Belgin Unal, Zhamyila Usupova, Hannu M. T. Uusitalo, Nalan Uysal, Sergio Valdes, Gonzalo Valdivia, Susana Vale, Popov I. Valery, Majid Valizadeh, Damaskini Valvi, Rob M. van Dam, Bert-Jan van den Born, Johan Van der Heyden, Yvonne T. van der Schouw, Koen Van Herck, Wendy Van Lippevelde, Hoang Van Minh, Natasja M. Van Schoor, Irene G. M. van Valkengoed, Dirk Vanderschueren, Diego Vanuzzo, Anette Varbo, Gregorio Varela-Moreiras, Luz Nayibe Vargas, Patricia Varona-Pérez, Senthil K. Vasan, Daniel G. Vasques, Radu Vatasescu, Tomas Vega, Toomas Veidebaum, Gustavo Velasquez-Melendez, Biruta Velika, Michel Velten, Charlotte Verdot, Maïté Verloigne, Giovanni Veronesi, W. M. Monique Verschuren, Germán Vicente-Rodríguez, Cesar G. Victora, Josep Vidal-Conti, Giovanni Viegi, Lucie Viet, Frøydis N. Vik, Monica Vilar, Salvador Villalpando, Luis Villarroel, Jesus Vioque, Napaphan Viriyautsahakul, Jyrki K. Virtanen, Marjolein Visser, Bharathi Viswanathan, Chiranthika Vithana, Mihaela Vladulescu, Tiina Vlasoff, Peter Vollenweider, Henry Völzke, Georgia Vourli, Ari Voutilainen, Martine Vrijheid, Tanja G. M. Vrijkotte, Silvije Vuletić, Alisha N. Wade, Wakenge Wakilongo, Thomas Waldhör, Janette Walton, Elvis O. A. Wambiya, Wan Mohamad Wan Bebakar, Wan Nazaimoon Wan Mohamud, Rildo de Souza Wanderley Júnior, Chongjian Wang, Huijun Wang, Ningli Wang, Qian Wang, Xiangjun Wang, Ya Xing Wang, Yi-Ren Wang, S. Goya Wannamethee, Nicholas Wareham, Olivia Wartha, Adelheid Weber, Karen Webster-Kerr, Niels Wedderkopp, Daniel Weghuber, Li Wei, Wenbin Wei, Aneta Weres, Bo Werner, Leo D. Westbury, Peter H. Whincup, Lars Wichstrøm, Kremlin Wickramasinghe, Kurt Widhalm, Indah S. Widyahening, Andrzej Więcek, Nilmini Wijemunige, Philipp S. Wild, Rainford J. Wilks, Johann Willeit, Karin Willeit, Peter Willeit, Julianne Williams, Tom Wilsgaard, James P. Wirth, Agnieszka Wiśniowska-Szurlej, Bogdan Wojtyniak, Meseret Woldeyohannes, Kathrin Wolf, Roy A. Wong-McClure, Andrew Wong, Emily B. Wong, Jyh Eiin Wong, Mark Woodward, Agnieszka E. Woźniak, Frederick C. Wu, Hon-Yen Wu, Jianfeng Wu, Li Juan Wu, Shouling Wu, Justyna Wyszyńska, Haiquan Xu, Liang Xu, Can Can Xue, Nor Azwany Yaacob, Uruwan Yamborisut, Li Yan, Lily D. Yan, Weili Yan, Ling Yang, Xiaoguang Yang, Yang Yang, Nazan Yardim, Chao-Yu Yeh, Martha Yépez García, Panayiotis K. Yiallouros, Agneta Yngve, Chandra Mandil Yogal, Moein Yoosefi, Akihiro Yoshihara, Yoto Yotov, Qi Sheng You, Yu-Ling Yu, Yunjiang Yu, Safiah Md Yusof, Ahmad Faudzi Yusoff, Luciana Zaccagni, Vassilis Zafiropulos, Ahmad A. Zainuddin, Farhad Zamani, Sabina Zambon, Antonis Zampelas, Hana Zamrazilová, Maria Elisa Zapata, Tomasz Zatoński, Ko Ko Zaw, Ayman A. Zayed, Tomasz Zdrojewski, Magdalena Żegleń, Kristyna Zejglicova, Girum Zeleke, Tajana Zeljkovic Vrkic, Yi Zeng, Andrea Zentai, Bing Zhang, Luxia Zhang, Zhen-Yu Zhang, Dong Zhao, Ming-Hui Zhao, Wenhua Zhao, Yanitsa V. Zhecheva, Wei Zheng, Bekbolat Zholdin, Maigeng Zhou, Dan Zhu, Oleg F. Zhukov, Sophia Zollner-Kiechl, Paul Zimmet, Marie Zins, Emanuel Zitt, Yanina Zocalo, Julio Zuñiga Cisneros, Monika Zuziak, Majid Ezzati

**Affiliations:** 1 Imperial College London, London, UK; 2 Johns Hopkins Bloomberg School of Public Health, Baltimore, MD USA; 3 World Health Organization, Geneva, Switzerland; 4 University of California Berkeley, Berkeley, CA USA; 5 Emory University, Atlanta, GA USA; 6 Bill & Melinda Gates Foundation, Seattle, WA USA; 7 University of Essex, Colchester, UK; 8 Copenhagen University Hospital — Bispebjerg and Frederiksberg, Copenhagen, Denmark; 9 Swiss Tropical and Public Health Institute, Basel, Switzerland; 10 University of Basel, Basel, Switzerland; 11 University of Zagreb, Zagreb, Croatia; 12 University of Ljubljana, Ljubljana, Slovenia; 13 Instituto Nacional de Ciencias Médicas y Nutrición, Mexico City, Mexico; 14 Madras Diabetes Research Foundation, Chennai, India; 15 The Hospital for Sick Children, Toronto, Ontario Canada; 16 The Aga Khan University, Karachi, Pakistan; 17 Ministry of Health, Victoria, Seychelles; 18 Unisanté, Lausanne, Switzerland; 19 Harvard TH Chan School of Public Health, Boston, MA USA; 20 Asfendiyarov Kazakh National Medical University, Almaty, Kazakhstan; 21 National Institute of Health, Kuala Lumpur, Malaysia; 22 RCSI University of Medicine and Health Sciences, Dublin, Ireland; 23 National Institutes of Biomedical Innovation, Health and Nutrition, Osaka, Japan; 24 African Population and Health Research Center, Nairobi, Kenya; 25 Seoul National University College of Medicine, Seoul, Republic of Korea; 26 University of Leicester, Leicester, UK; 27 University of Eastern Finland, Kuopio, Finland; 28 Finnish Institute for Health and Welfare, Helsinki, Finland; 29 ICMR — National Institute of Nutrition, Hyderabad, India; 30 University of Malaya, Kuala Lumpur, Malaysia; 31 National Taiwan University, Taipei, Taiwan; 32 University of Yaoundé 1, Yaoundé, Cameroon; 33 University of Sydney, Sydney, New South Wales Australia; 34 Robert Koch Institute, Berlin, Germany; 35 University of Oulu, Oulu, Finland; 36 Public Health Promotion and Development Organization, Kathmandu, Nepal; 37 Peking University, Beijing, China; 38 Chinese Center for Disease Control and Prevention, Beijing, China; 39 The University of the West Indies, Kingston, Jamaica; 40 Caja Costarricense de Seguro Social, San José, Costa Rica; 41 Non-Communicable Diseases Research Center, Tehran, Iran; 42 Al-Quds University, East Jerusalem, Palestine; 43 S Kairbekova National Research Center for Health Development, Astana, Kazakhstan; 44 Ministry of Health, Kuala Lumpur, Malaysia; 45 Qatar University, Doha, Qatar; 46 Ministry of Health and Social Protection, Dushanbe, Tajikistan; 47 Birzeit University, Birzeit, Palestine; 48 Usmanu Danfodiyo University Teaching Hospital, Sokoto, Nigeria; 49 Instituto Mexicano del Seguro Social, Mexico City, Mexico; 50 Qassim University, Unaizah, Saudi Arabia; 51 RehaKlinika, Rzeszów, Poland; 52 Flinders University, Adelaide, South Australia Australia; 53 University of Ghana, Accra, Ghana; 54 Mahidol University, Nakhon Pathom, Thailand; 55 National Research and Innovation Agency, Jakarta, Indonesia; 56 BRAC James P Grant School of Public Health, Dhaka, Bangladesh; 57 Copenhagen University Hospital — Herlev and Gentofte, Copenhagen, Denmark; 58 University of Copenhagen, Copenhagen, Denmark; 59 Food and Nutrition Research Institute, Taguig, Philippines; 60 Urmia University of Medical Sciences, Urmia, Iran; 61 Umeå University, Umeå, Sweden; 62 Ibn Tofail University, Kénitra, Morocco; 63 Amsterdam University Medical Center, Amsterdam, The Netherlands; 64 Shahrekord University of Medical Sciences, Shahrekord, Iran; 65 Tehran University of Medical Sciences, Tehran, Iran; 66 University of Hargeisa, Hargeisa, Somalia; 67 Leibniz Institute for Prevention Research and Epidemiology — BIPS, Bremen, Germany; 68 Republican Center for Health Promotion and Mass Communication, Bishkek, Kyrgyzstan; 69 National Center for Diabetes, Endocrinology and Genetics, Amman, Jordan; 70 Tashkent Pediatric Medical Institute, Tashkent, Uzbekistan; 71 King Saud University, Riyadh, Saudi Arabia; 72 Kuwait Institute for Scientific Research, Kuwait City, Kuwait; 73 Princess Nourah bint Abdulrahman University, Riyadh, Saudi Arabia; 74 Ministry of Health, Muscat, Oman; 75 King Abdulaziz University, Jeddah, Saudi Arabia; 76 The Hashemite University, Zarqa, Jordan; 77 World Health Organization Regional Office for the Eastern Mediterranean, Cairo, Egypt; 78 Ministry of Health, Kuwait City, Kuwait; 79 Dasman Diabetes Institute, Kuwait City, Kuwait; 80 Aldara Hospital and Medical Center, Riyadh, Saudi Arabia; 81 King Abdullah International Medical Research Center, Riyadh, Saudi Arabia; 82 Universidade Federal da Integração Latino-Americana, Foz do Iguaçu, Brazil; 83 Republican Specialized Scientific-and-Practical Medical Centre of Endocrinology named after academician Y.Kh. Turakulov, Tashkent, Uzbekistan; 84 CIBERESP, Madrid, Spain; 85 Barcelona Institute for Global Health, Barcelona, Spain; 86 Bombay Hospital and Medical Research Centre, Mumbai, India; 87 Research Center for Social Determinants of Health, Tehran, Iran; 88 Ghana Health Service, Kintampo, Ghana; 89 UMR CNRS-MNHN 7206, Paris, France; 90 University of Lille, Lille, France; 91 Lille University Hospital, Lille, France; 92 Endocrine Research Center, Tehran, Iran; 93 Western Norway University of Applied Sciences, Sogndal, Norway; 94 Norwegian School of Sport Sciences, Oslo, Norway; 95 University of Thessaly, Trikala, Greece; 96 Ministry of Health and Social Action, Dakar, Senegal; 97 Health Promotion Research Center, Zahedan, Iran; 98 Yekaterinburg State Medical Academy, Yekaterinburg, Russia; 99 National Institute of Public Health, Tunis, Tunisia; 100 University of Porto, Porto, Portugal; 101 Norwegian Institute of Public Health, Oslo, Norway; 102 Spanish Agency for Food Safety and Nutrition, Madrid, Spain; 103 University of Massachusetts Amherst, Amherst, MA USA; 104 University of Almería, Almería, Spain; 105 Nepal Health Economics Association, Kathmandu, Nepal; 106 University of Bergen, Bergen, Norway; 107 University of Iceland, Reykjavik, Iceland; 108 Haramaya University, Dire Dawa, Ethiopia; 109 Universidade Federal de Pelotas, Pelotas, Brazil; 110 The University of Hong Kong, Hong Kong, China; 111 University of Medicine 1, Yangon, Myanmar; 112 Oulu University Hospital, Oulu, Finland; 113 Regional Authority of Public Health, Banska Bystrica, Slovakia; 114 Diabetic Association of Bangladesh, Dhaka, Bangladesh; 115 Neyshabur University of Medical Sciences, Neyshabur, Iran; 116 Shahid Beheshti University of Medical Sciences, Tehran, Iran; 117 Indian Council of Medical Research, New Delhi, India; 118 National and Kapodistrian University of Athens, Athens, Greece; 119 Institute of Public Health, Podgorica, Montenegro; 120 University of Sarajevo, Sarajevo, Bosnia and Herzegovina; 121 National Center for Public Health and Pharmacy, Budapest, Hungary; 122 National Medical Research Center for Therapy and Preventive Medicine, Moscow, Russia; 123 University of Science and Technology, Sana’a, Yemen; 124 The John Paul II Catholic University of Lublin, Lublin, Poland; 125 Universidad Autónoma de Madrid, Madrid, Spain; 126 University of Rzeszów, Rzeszów, Poland; 127 University of Palermo, Palermo, Italy; 128 Ceara State University, Ceara, Brazil; 129 University of Miami, Miami, FL USA; 130 University Hospital Centre Zagreb, Zagreb, Croatia; 131 Mohammed V University, Rabat, Morocco; 132 Unidad de Cirugia Cardiovascular, Guatemala City, Guatemala; 133 Universidad del Valle, Cali, Colombia; 134 National Institute of Health Doutor Ricardo Jorge, Lisbon, Portugal; 135 NOVA University Lisbon, Lisbon, Portugal; 136 University of Pernambuco, Recife, Brazil; 137 Wroclaw Medical University, Wroclaw, Poland; 138 Indus Hospital & Health Network, Karachi, Pakistan; 139 Simon Fraser University, Burnaby, British Columbia Canada; 140 Dalhousie University, Halifax, Nova Scotia Canada; 141 Jordan University of Science and Technology, Irbid, Jordan; 142 Universidade Federal de Ouro Preto, Ouro Preto, Brazil; 143 Universidade Federal do Maranhão, São Luís, Brazil; 144 National Center of Public Health, Astana, Kazakhstan; 145 Agency for Preventive and Social Medicine, Bregenz, Austria; 146 Cliniques Universitaires de Kinshasa, Kinshasa, Democratic Republic of Congo; 147 University of Auckland, Auckland, New Zealand; 148 University College Dublin, Dublin, Ireland; 149 Christian Medical College Vellore, Vellore, India; 150 Universitat Jaume I, Castellon de la Plana, Spain; 151 University Tunis El Manar, Tunis, Tunisia; 152 George Emil Palade University of Medicine, Pharmacy, Sciences and Technology of Târgu Mureș, Târgu Mureș, Romania; 153 Federal Ministry of Social Affairs, Health, Care and Consumer Protection, Vienna, Austria; 154 Cafam University Foundation, Bogotá, Colombia; 155 Ministerio de Salud Pública y Bienestar Social, Asunción, Paraguay; 156 Universidad Politécnica de Madrid, Madrid, Spain; 157 University of Agder, Kristiansand, Norway; 158 Sciensano, Brussels, Belgium; 159 Addis Continental Institute of Public Health, Addis Ababa, Ethiopia; 160 Kazakh National Medical University, Almaty, Kazakhstan; 161 Universidad Científica del Sur, Lima, Peru; 162 Lithuanian University of Health Sciences, Kaunas, Lithuania; 163 Pontificia Universidad Católica de Chile, Santiago, Chile; 164 Gasol Foundation, Madrid, Spain; 165 University of Alcalá, Alcalá de Henares, Spain; 166 University of São Paulo, São Paulo, Brazil; 167 Johannes Gutenberg University, Mainz, Germany; 168 B J Medical College, Ahmedabad, India; 169 Chirayu Medical College, New Delhi, India; 170 Sunder Lal Jain Hospital, Delhi, India; 171 Shandong University of Traditional Chinese Medicine, Jinan, China; 172 Shanghai Jiao-Tong University School of Medicine, Shanghai, China; 173 Universidad de la República, Montevideo, Uruguay; 174 University of Strasbourg, Strasbourg, France; 175 Institute of Medical Research and Medicinal Plant Studies, Yaoundé, Cameroon; 176 Ufa Eye Research Institute, Ufa, Russia; 177 Nepal Health Research Council, Kathmandu, Nepal; 178 University of Montenegro, Niksic, Montenegro; 179 University of Southern Denmark, Copenhagen, Denmark; 180 University of Oslo, Oslo, Norway; 181 University of Gothenburg, Gothenburg, Sweden; 182 Ministry of Health, Jerusalem, Israel; 183 Universidade Federal do Rio de Janeiro, Rio de Janeiro, Brazil; 184 National Institute for Public Health and the Environment, Bilthoven, The Netherlands; 185 University of Turin, Turin, Italy; 186 University College London, London, UK; 187 Liverpool John Moores University, Liverpool, UK; 188 Nanyang Technological University, Singapore, Singapore; 189 National Medical Research Center for Endocrinology, Moscow, Russia; 190 Centro de Educación Médica e Investigaciones Clínicas, Buenos Aires, Argentina; 191 IRCCS Neuromed, Pozzilli, Italy; 192 University of Toulouse, Toulouse, France; 193 University of Zurich, Zurich, Switzerland; 194 KU Leuven, Leuven, Belgium; 195 University of Kinshasa, Kinshasa, Democratic Republic of Congo; 196 Anglia Ruskin University, Cambridge, UK; 197 World Health Organization Country Office, Dushanbe, Tajikistan; 198 Flemish Agency for Care and Health, Brussels, Belgium; 199 Ghent University, Ghent, Belgium; 200 FrieslandCampina, Amersfoort, The Netherlands; 201 KHP Centre for Translational Medicine, London, UK; 202 Universidad Central de Venezuela, Caracas, Venezuela; 203 University of Geneva, Geneva, Switzerland; 204 Bielefeld University, Bielefeld, Germany; 205 World Health Organization Athens Quality of Care Office, Athens, Greece; 206 German Cancer Research Center, Heidelberg, Germany; 207 University of Amsterdam, Amsterdam, The Netherlands; 208 The Fred Hollows Foundation, Auckland, New Zealand; 209 University of the Andes, Mérida, Venezuela; 210 Carol Davila University of Medicine and Pharmacy, Bucharest, Romania; 211 Instituto Politécnico de Lisboa, Lisbon, Portugal; 212 Swansea University, Swansea, UK; 213 University College Copenhagen, Copenhagen, Denmark; 214 World Health Organization Regional Office for Europe, Copenhagen, Denmark; 215 University of Medicine, Tirana, Albania; 216 Munster Technological University, Cork, Ireland; 217 Universidad de La Laguna, Tenerife, Spain; 218 University of Malta, Msida, Malta; 219 University of Granada, Granada, Spain; 220 Vanderbilt University, Nashville, TN USA; 221 Pan American Health Organization, Washington, DC USA; 222 Ministry of Health, Tongatapu, Tonga; 223 Canadian Fitness and Lifestyle Research Institute, Ottawa, Ontario Canada; 224 Hospital Santa Maria, Lisbon, Portugal; 225 University of Padua, Padua, Italy; 226 Istanbul University-Cerrahpasa, Istanbul, Türkiye; 227 Universidade Federal de Juiz de Fora, Juiz de Fora, Brazil; 228 National Institute of Public Health, Prague, Czechia; 229 Canopo Study Center - Salerno, Salerno, Italy; 230 University of Groningen, Groningen, The Netherlands; 231 Karolinska Institutet, Huddinge, Sweden; 232 Centro de Estudios Sobre Nutrición Infantil, Buenos Aires, Argentina; 233 Universidade Federal de Minas Gerais, Belo Horizonte, Brazil; 234 University of Zaragoza, Zaragoza, Spain; 235 Santiago de Compostela University, Santiago de Compostela, Spain; 236 University of Cádiz, Cádiz, Spain; 237 Ministry of Health, Ankara, Türkiye; 238 Council for Agricultural Research and Economics, Rome, Italy; 239 Universidade Federal do Rio Grande, Rio Grande, Brazil; 240 Chulalongkorn University, Bangkok, Thailand; 241 India Diabetes Research Foundation, Chennai, India; 242 Duke-NUS Medical School, Singapore, Singapore; 243 Federation University Australia, Ballarat, Victoria Australia; 244 Paris Cité University, Paris, France; 245 ICMR — National Institute of Medical Statistics, New Delhi, India; 246 Capital Institute of Pediatrics, Beijing, China; 247 Xiangtan University, Xiangtan, China; 248 Ministry of Health and Welfare, Taipei, Taiwan; 249 Kailuan General Hospital, Tangshan, China; 250 University of Oxford, Oxford, UK; 251 National University of Singapore, Singapore, Singapore; 252 US Centers for Disease Control and Prevention, Atlanta, GA USA; 253 Ahvaz Jundishapur University of Medical Sciences, Ahvaz, Iran; 254 The Gertner Institute for Epidemiology and Health Policy Research, Ramat Gan, Israel; 255 National Center of Public Health and Analyses, Sofia, Bulgaria; 256 University of Fribourg, Fribourg, Switzerland; 257 Victor Babes University of Medicine and Pharmacy, Timisoara, Romania; 258 RIPAS Hospital, Bandar Seri Begawan, Brunei; 259 University of Southern Denmark, Odense, Denmark; 260 Universidade Estadual Paulista, Presidente Prudente, Brazil; 261 Medical University of Silesia, Katowice, Poland; 262 Istituto Superiore di Sanità, Rome, Italy; 263 Charles University, Prague, Czechia; 264 Thomayer University Hospital, Prague, Czechia; 265 Ministry for Health and Active Ageing, Floriana, Malta; 266 University of Salerno, Fisciano, Italy; 267 Ministry of Health, Port of Spain, Trinidad and Tobago; 268 Statistics Canada, Ottawa, Ontario Canada; 269 Nicolae Testemițanu State University of Medicine and Pharmacy, Chisinau, Moldova; 270 Universidad Miguel Hernández de Elche, Alicante, Spain; 271 University of the Extreme South of Santa Catarina, São Luís, Brazil; 272 University of Southampton, Southampton, UK; 273 Pontificia Universidad Javeriana, Bogotá, Colombia; 274 University of Insubria, Varese, Italy; 275 Malawi Epidemiology and Intervention Research Unit, Lilongwe, Malawi; 276 National Nutrition Agency, Banjul, The Gambia; 277 CIBEROBN, Madrid, Spain; 278 Hungarian University of Sports Science, Budapest, Hungary; 279 University of Debrecen, Debrecen, Hungary; 280 National Institute of Public Health, Bucharest, Romania; 281 Universidade Federal da Paraíba, João Pessoa, Brazil; 282 National Research Council, Reggio Calabria, Italy; 283 Universidade Federal de Santa Catarina, Florianópolis, Brazil; 284 Universidade Federal de Alagoas, Alagoas, Brazil; 285 Eftimie Murgu University Resita, Resita, Romania; 286 Ahmadu Bello University, Zaria, Nigeria; 287 Aarhus University, Aarhus, Denmark; 288 Institut Pasteur de Lille, Lille, France; 289 Eduardo Mondlane University, Maputo, Mozambique; 290 Indian Statistical Institute, Kolkata, India; 291 Tabriz Health Services Management Research Center, Tabriz, Iran; 292 Universidade Nasionál Timór Lorosa’e, Dili, Timor-Leste; 293 Karolinska Institutet, Stockholm, Sweden; 294 Associazione Calabrese di Epatologia, Reggio Calabria, Italy; 295 Aragón Health Research Institute Foundation, Zaragoza, Spain; 296 University of Heidelberg, Heidelberg, Germany; 297 Harokopio University of Athens, Athens, Greece; 298 Innovating Health International, Port-au-Prince, Haiti; 299 French National Research Institute for Sustainable Development, Montpellier, France; 300 The National Public Health Agency, St Maurice, France; 301 Astrakhan State Medical University, Astrakhan, Russia; 302 Universidade do Vale do Rio dos Sinos, São Leopoldo, Brazil; 303 National Council of Scientific and Technical Research, Buenos Aires, Argentina; 304 National Institute of Hygiene, Epidemiology and Microbiology, Havana, Cuba; 305 Ministry of Health and Medical Education, Tehran, Iran; 306 University of Novi Sad, Novi Sad, Serbia; 307 National Institute of Nutrition, Hanoi, Viet Nam; 308 University of Queensland, Brisbane, Queensland Australia; 309 Instituto de Investigación Nutricional, Lima, Peru; 310 Sun Yat-sen University, Guangzhou, China; 311 Capital Medical University Beijing Tongren Hospital, Beijing, China; 312 Universidad de Cuenca, Cuenca, Ecuador; 313 University of Las Palmas de Gran Canaria, Las Palmas de Gran Canaria, Spain; 314 Helmholtz Zentrum München, Munich, Germany; 315 Romanian Academy, Bucharest, Romania; 316 University Medicine Greifswald, Greifswald, Germany; 317 University Hospital Düsseldorf, Düsseldorf, Germany; 318 Medical University of Lodz, Lodz, Poland; 319 Calisia University, Kalisz, Poland; 320 University of North Carolina at Chapel Hill, Chapel Hill, NC USA; 321 Beijing Center for Disease Prevention and Control, Beijing, China; 322 IRL 3189 ESS, Marseille, France; 323 Scuola Superiore Sant’Anna, Pisa, Italy; 324 Al-Farabi Kazakh National University, Almaty, Kazakhstan; 325 Murdoch Children’s Research Institute, Parkville, Victoria Australia; 326 Semey Medical University, Semey, Kazakhstan; 327 University of Latvia, Riga, Latvia; 328 Institute of Mother and Child, Warsaw, Poland; 329 Ministry of Health and Medical Services, Gizo, Solomon Islands; 330 Hormozgan University of Medical Sciences, Bandar Abbas, Iran; 331 University of Benin, Benin City, Nigeria; 332 University of Skövde, Skövde, Sweden; 333 Ministry of Health, Rabat, Morocco; 334 National Institute of Nutrition and Food Technology, Tunis, Tunisia; 335 University of Calabar, Calabar, Nigeria; 336 University of Haifa, Haifa, Israel; 337 University of California Davis, Davis, CA USA; 338 Region Västernorrland, Härnösand, Sweden; 339 Stellenbosch University, Stellenbosch, South Africa; 340 Karadeniz Technical University, Trabzon, Türkiye; 341 Dokuz Eylul University, Izmir, Türkiye; 342 University of Helsinki, Helsinki, Finland; 343 Rafsanjan University of Medical Sciences, Rafsanjan, Iran; 344 Queen’s University Belfast, Belfast, UK; 345 Monash University, Melbourne, Victoria Australia; 346 Luxembourg Institute of Health, Strassen, Luxembourg; 347 Tabriz University of Medical Sciences, Tabriz, Iran; 348 Fasa University of Medical Sciences, Fasa, Iran; 349 Shiraz University of Medical Sciences, Shiraz, Iran; 350 Baqai Medical University, Karachi, Pakistan; 351 Gesundheit Österreich, Wien, Austria; 352 Centro de Salud Villanueva Norte, Badajoz, Spain; 353 Hospital Don Benito-Villanueva de la Serena, Badajoz, Spain; 354 University of Colombo, Colombo, Sri Lanka; 355 London School of Hygiene & Tropical Medicine, London, UK; 356 Ministry of Health, Buenos Aires, Argentina; 357 Universidad de Santiago de Chile, Santiago, Chile; 358 Instituto PENSI Sabara Hospital, São Paulo, Brazil; 359 University of Tartu, Tartu, Estonia; 360 Georgia College and State University, Milledgeville, GA USA; 361 Universidade Federal de São Paulo, São Paulo, Brazil; 362 University Medical Centre Ljubljana, Ljubljana, Slovenia; 363 University of Tasmania, Hobart, Tasmania Australia; 364 Hospital Universitario Son Espases, Palma, Spain; 365 Hospital de Clinicas de Porto Alegre, Porto Alegre, Brazil; 366 Universidade Federal do Rio Grande do Sul, Porto Alegre, Brazil; 367 Kansai Medical University, Hirakata, Japan; 368 Kyoto University, Kyoto, Japan; 369 Ternopil National Medical University, Ternopil, Ukraine; 370 Medical University of Warsaw, Warsaw, Poland; 371 Public Health and Reforms Center, Baku, Azerbaijan; 372 Medical University of Varna, Varna, Bulgaria; 373 Ministero della Salute DG Prevenzione Sanitaria, Rome, Italy; 374 University of Catania, Catania, Italy; 375 Ministry of Public Health, Yaren, Nauru; 376 University of Deusto, Bilbao, Spain; 377 Africa Health Research Institute, Mtubatuba, South Africa; 378 Geneva University Hospitals, Geneva, Switzerland; 379 Hospital del Mar Research Institute, Barcelona, Spain; 380 Utrecht University, Utrecht, The Netherlands; 381 Wageningen University, Wageningen, The Netherlands; 382 Medical Research Foundation, Chennai, India; 383 Bayero University, Kano, Nigeria; 384 Kurdistan University of Medical Sciences, Sanandaj, Iran; 385 Endocrine Physiology Research Center, Tehran, Iran; 386 University of South Carolina, Columbia, SC USA; 387 American University of Beirut, Beirut, Lebanon; 388 University of Adelaide, Adelaide, South Australia Australia; 389 Ministry of Health, Mbabane, Eswatini; 390 Lund University, Lund, Sweden; 391 Aristotle University of Thessaloniki, Thessaloniki, Greece; 392 Central University of Kerala, Kasaragod, India; 393 Institut National de la Santé et de la Recherche Médicale, Paris, France; 394 McGill University, Montreal, Québec Canada; 395 Universidad de Costa Rica, San José, Costa Rica; 396 Gasol Foundation, Barcelona, Spain; 397 University of Lleida, Sant Boi de Llobregat, Spain; 398 PASs Hirszfeld Institute of Immunology and Experimental Therapy, Wroclaw, Poland; 399 University Agostinho Neto, Luanda, Angola; 400 Kansas State University, Manhattan, KS USA; 401 St. Ann’s University Hospital, Brno, Czechia; 402 Universidad Autónoma de Santo Domingo, Santo Domingo, Dominican Republic; 403 Ministry of Health, Lisbon, Portugal; 404 Institute for Clinical and Experimental Medicine, Prague, Czechia; 405 Children’s Memorial Health Institute, Warsaw, Poland; 406 University of Thessaly, Larissa, Greece; 407 Ministry of Health and Wellness, Kingston, Jamaica; 408 UiT The Arctic University of Norway, Tromsø, Norway; 409 National Center of Cardiovascular Diseases, Beijing, China; 410 International Life Science Institute, Buenos Aires, Argentina; 411 University of Ferrara, Ferrara, Italy; 412 Icelandic Heart Association, Kopavogur, Iceland; 413 French National Research Institute for Sustainable Development, Limoges, France; 414 Universidad Icesi, Cali, Colombia; 415 State University of Montes Claros, Montes Claros, Brazil; 416 King’s College London, London, UK; 417 Capital Medical University, Beijing, China; 418 Healis-Sekhsaria Institute for Public Health, Navi Mumbai, India; 419 Singapore National Eye Centre, Singapore, Singapore; 420 Eternal Heart Care Centre and Research Institute, Jaipur, India; 421 University of Ibadan, Ibadan, Nigeria; 422 University of Belgrade Institute for Medical Research, Belgrade, Serbia; 423 Institute for Clinical Effectiveness and Health Policy, Buenos Aires, Argentina; 424 ISGlobal, Barcelona, Spain; 425 National Health Insurance Service, Wonju, Republic of Korea; 426 Prevention of Metabolic Disorders Research Center, Tehran, Iran; 427 Research and Education Institute of Child Health, Nicosia, Cyprus; 428 Danish Cancer Institute, Copenhagen, Denmark; 429 The University of the West Indies, Cave Hill, Barbados; 430 Kermanshah University of Medical Sciences, Kermanshah, Iran; 431 Universidade Federal de Pernambuco, Recife, Brazil; 432 National Institute of Public Health, Copenhagen, Denmark; 433 Yasuj University of Medical Sciences, Yasuj, Iran; 434 Zahedan University of Medical Sciences, Zahedan, Iran; 435 International Hellenic University, Thessaloniki, Greece; 436 Kyushu University, Fukuoka, Japan; 437 UNICEF, New York City, NY USA; 438 University of Texas Southwestern Medical Center, Dallas, TX USA; 439 National Research Institute for Health and Family Planning, Beijing, China; 440 University of Pécs, Pécs, Hungary; 441 Danish Health Authority, Copenhagen, Denmark; 442 Joep Lange Institute, Amsterdam, The Netherlands; 443 National Institute of Public Health, Cuernavaca, Mexico; 444 Universidad Autónoma de Bucaramanga, Bucaramanga, Colombia; 445 ETH Zurich, Zurich, Switzerland; 446 Chronic Diseases Research Center, Tehran, Iran; 447 University of Western Australia, Perth, Western Australia Australia; 448 Universidade Federal do Paraná, Curitiba, Brazil; 449 University Ramon Llull, Barcelona, Spain; 450 Vrije Universiteit Amsterdam, Amsterdam, The Netherlands; 451 Kingston Health Sciences Centre, Kingston, Ontario, Canada; 452 Fundación Oftalmológica de Santander, Bucaramanga, Colombia; 453 Obesity Research Center, Tehran, Iran; 454 University Oran 1, Oran, Algeria; 455 Independent Public Health Specialist, Nay Pyi Taw, Myanmar; 456 Ministry of Health and Sports, Nay Pyi Taw, Myanmar; 457 Instituto Murciano de Investigación Biosanitaria, Murcia, Spain; 458 International Agency for Research on Cancer, Lyon, France; 459 Institute of Public Health, Tirana, Albania; 460 Giuseppe Degennaro LUM University, Casamassima, Italy; 461 University of Nigeria, Enugu, Nigeria; 462 Ministry of Health and Human Services, Koror, Palau; 463 Erasmus Medical Center Rotterdam, Rotterdam, The Netherlands; 464 Fundación para la Investigación Sanitaria y Biomédica de la Comunidad Valenciana, Valencia, Spain; 465 Marshall Islands Epidemiology & Prevention Initiatives, Majuro, Marshall Islands; 466 The University of Tokyo, Tokyo, Japan; 467 Alex Ekwueme Federal University Teaching Hospital, Abakaliki, Nigeria; 468 Texas Tech University, Dallas, TX USA; 469 Bulgarian Academy of Sciences, Sofia, Bulgaria; 470 Hokkaido University, Tokyo, Japan; 471 Hadassah University Medical Center, Jerusalem, Israel; 472 Université Catholique de Louvain, Brussels, Belgium; 473 Slovenian Heart Foundation, Ljubljana, Slovenia; 474 Public Health Agency of Sweden, Stockholm, Sweden; 475 Norwegian University of Science and Technology, Trondheim, Norway; 476 University of Melbourne, Melbourne, Victoria, Australia; 477 Sports University of Tirana, Tirana, Albania; 478 University of Graz, Graz, Austria; 479 Jagiellonian University Medical College, Kraków, Poland; 480 University of Rijeka, Rijeka, Croatia; 481 Heart Foundation, Melbourne, Victoria, Australia; 482 University of Bisha, Bisha, Saudi Arabia; 483 Universidad Eugenio Maria de Hostos, Santo Domingo, Dominican Republic; 484 University of Duisburg-Essen, Essen, Germany; 485 Rothschild Foundation Hospital, Paris, France; 486 University of New South Wales, Sydney, New South Wales Australia; 487 University Hospital Centre Sestre Milosrdnice, Zagreb, Croatia; 488 Guilan University of Medical Sciences, Rasht, Iran; 489 University of Opole, Opole, Poland; 490 Gulu University, Gulu, Uganda; 491 Capacity Development in Nutrition CAPNUTRA, Belgrade, Serbia; 492 University of Crete, Heraklion, Greece; 493 Hungarian School Sport Federation, Budapest, Hungary; 494 Mbarara University of Science and Technology, Mbarara, Uganda; 495 National Center for Disease Control and Public Health, Tbilisi, Georgia; 496 Tbilisi State Medical University, Tbilisi, Georgia; 497 Ministry of Health, Bratislava, Slovakia; 498 Reichman University, Herzliya, Israel; 499 Sri Venkateswara University, Tirupati, India; 500 Sree Chitra Tirunal Institute for Medical Sciences and Technology, Trivandrum, India; 501 Hellenic Medical Association for Obesity, Athens, Greece; 502 Maharajgunj Medical Campus, Kathmandu, Nepal; 503 Duke University, Durham, NC USA; 504 University of Bouaké, Bouaké, Côte d’Ivoire; 505 Université Officielle de Bukavu, Bukavu, Democratic Republic of Congo; 506 Pennington Biomedical Research Center, Baton Rouge, LA USA; 507 National Institute of Epidemiology, Chennai, India; 508 UNICEF, Lilongwe, Malawi; 509 University of Toronto, Toronto, Ontario, Canada; 510 Shanghai University of Sport, Shanghai, China; 511 University of Münster, Münster, Germany; 512 Israel Center for Disease Control, Ramat Gan, Israel; 513 Child Growth and Development Research Center, Isfahan, Iran; 514 Mashhad University of Medical Sciences, Mashhad, Iran; 515 Research Institute of Child Nutrition, Dortmund, Germany; 516 Ministry of Health, Gaborone, Botswana; 517 Research Institute for Endocrine Sciences, Tehran, Iran; 518 Mazandaran University of Medical Sciences, Sari, Iran; 519 Hypertension Research Center, Isfahan, Iran; 520 Ministry of Public Health, Kabul, Afghanistan; 521 Medical University of Innsbruck, Innsbruck, Austria; 522 VASCage Research Centre on Vascular Ageing and Stroke, Innsbruck, Austria; 523 Muhimbili University of Health and Allied Sciences, Dar es Salaam, Tanzania; 524 Yonsei University College of Medicine, Seoul, Republic of Korea; 525 Sahlgrenska University Hospital, Gothenburg, Sweden; 526 University College South Denmark, Haderslev, Denmark; 527 Thaksin University, Songkhla, Thailand; 528 Masaryk University, Brno, Czechia; 529 Ulm University Hospital, Ulm, Germany; 530 B P Koirala Institute of Health Sciences, Dharan, Nepal; 531 Nepal Development Society, Kathmandu, Nepal; 532 International Committee of Red Cross, Maiduguri, Nigeria; 533 University of Vienna, Vienna, Austria; 534 Tartu University Clinics, Tartu, Estonia; 535 National Institute of Public Health, Abidjan, Côte d’Ivoire; 536 Hildburghausen District Department of State Public Health Service, Hildburghausen, Germany; 537 Pontificia Universidad Católica Argentina, Buenos Aires, Argentina; 538 Ministry of Health and Wellness, Port Louis, Mauritius; 539 University Hospital Ulm, Ulm, Germany; 540 Croatian Institute of Public Health, Zagreb, Croatia; 541 Institute of Nutrition of Central America and Panama, Guatemala City, Guatemala; 542 North-West University, Potchefstroom, South Africa; 543 South African Medical Research Council, Cape Town, South Africa; 544 University of Physical Culture in Kraków, Kraków, Poland; 545 University of Jyväskylä, Jyväskylä, Finland; 546 Research Institute of Cardiology and Internal Diseases, Almaty, Kazakhstan; 547 ICMR — National Centre for Disease Informatics and Research, Bengaluru, India; 548 Amrita Institute of Medical Sciences, Cochin, India; 549 Institute of Endocrinology, Prague, Czechia; 550 All India Institute of Medical Sciences, New Delhi, India; 551 Hanoi University of Public Health, Hanoi, Viet Nam; 552 Hassan I University, Settat, Morocco; 553 University of Limpopo, Polokwane, South Africa; 554 Public University of Navarra, Pamplona, Spain; 555 Universiti Brunei Darussalam, Bandar Seri Begawan, Brunei; 556 Ministry of Health, Algiers, Algeria; 557 Ministry of Health, Georgetown, Guyana; 558 Oulu Deaconess Institute Foundation, Oulu, Finland; 559 Sahlgrenska Academy, Gothenburg, Sweden; 560 Endocrinology and Metabolism Research Center, Tehran, Iran; 561 University of Public Health, Yangon, Myanmar; 562 Centro Studi Epidemiologici di Gubbio, Gubbio, Italy; 563 Universidad Peruana Cayetano Heredia, Lima, Peru; 564 National University Health System, Singapore, Singapore; 565 Tampere University Hospital, Tampere, Finland; 566 Tampere University, Tampere, Finland; 567 University of Douala, Douala, Cameroon; 568 University of the Basque Country, Leioa, Spain; 569 University of the Basque Country, Donostia-San Sebastian, Spain; 570 The Russian University of Sport, Moscow, Russia; 571 National Institute for Health Development, Tallinn, Estonia; 572 West Virginia University, Morgantown, WV USA; 573 Tan Tock Seng Hospital, Singapore, Singapore; 574 Fundação Oswaldo Cruz, Belo Horizonte, Brazil; 575 Uppsala University, Uppsala, Sweden; 576 University of Augsburg, Augsburg, Germany; 577 Capital Medical University Beijing An Zhen Hospital, Beijing, China; 578 Zhengzhou University, Zhengzhou, China; 579 FISABIO-Universitat Jaume I-Universitat de València, Valencia, Spain; 580 Taipei Medical University, Taipei, Taiwan; 581 Ministry of Health, Colombo, Sri Lanka; 582 Children’s Hospital of Eastern Ontario Research Institute, Ottawa, Ontario, Canada; 583 Sports Medical Center of Minho, Braga, Portugal; 584 Universidad Espíritu Santo, Samborondón, Ecuador; 585 Universidad de Los Lagos, Osorno, Chile; 586 Universidad San Martín de Porres, Lima, Peru; 587 Regional Ministry of Health, Valladolid, Spain; 588 Universidade Federal de Ciências da Saúde de Porto Alegre, Porto Alegre, Brazil; 589 Ministerio de Salud Pública, Quito, Ecuador; 590 Ilembula Lutheran Hospital, Ilembula, Tanzania; 591 University of Kinshasa Hospital, Kinshasa, Democratic Republic of Congo; 592 University of Coimbra, Coimbra, Portugal; 593 UMR 7268 ADES, Marseille, France; 594 Coimbra University Hospital Center, Coimbra, Portugal; 595 University of Texas Rio Grande Valley, Harlingen, TX USA; 596 Institute of Neuroscience of the National Research Council, Padua, Italy; 597 Baker Heart and Diabetes Institute, Melbourne, Victoria, Australia; 598 Agricultural University of Athens, Athens, Greece; 599 Academia VBHC, São Paulo, Brazil; 600 SB RAS Federal Research Center Institute of Cytology and Genetics, Novosibirsk, Russia; 601 University of Otago, Dunedin, New Zealand; 602 University of Northern British Columbia, Prince George, British Columbia Canada; 603 Hellenic Mediterranean University, Siteia, Greece; 604 Loughborough University, Loughborough, UK; 605 University of Split, Split, Croatia; 606 Ministry of Health, Nicosia, Cyprus; 607 Lausanne University Hospital, Lausanne, Switzerland; 608 University of Lausanne, Lausanne, Switzerland; 609 Fundação Oswaldo Cruz, Rio de Janeiro, Brazil; 610 CIBERCV, Madrid, Spain; 611 Hasselt University, Hasselt, Belgium; 612 Mary Immaculate College, Limerick, Ireland; 613 Universidad de Castilla-La Mancha, Cuenca, Spain; 614 Hungarian Society of Sports Medicine, Budapest, Hungary; 615 Nnamdi Azikiwe University, Awka, Nigeria; 616 Paracelsus Medical University, Salzburg, Austria; 617 Institute for Cancer Research, Prevention and Clinical Network, Florence, Italy; 618 Universidade Estadual do Centro-Oeste, Guarapuava, Brazil; 619 Université Catholique de Bukavu, Bukavu, Democratic Republic of Congo; 620 Sefako Makgatho Health Sciences University, Pretoria, South Africa; 621 Centro de Estudos do Laboratório de Aptidão Física de São Caetano do Sul, São Paulo, Brazil; 622 George Institute for Global Health, New Delhi, India; 623 Brown University, Providence, RI USA; 624 Shahid Sadoughi University of Medical Sciences, Yazd, Iran; 625 Arabkir Medical Centre — Institute of Child and Adolescent Health, Yerevan, Armenia; 626 Pontificia Universidad Católica de Valparaíso, Valparaíso, Chile; 627 Center for Studies and Research on Social Dynamics and Health, Lisbon, Portugal; 628 Universidad de los Andes, Bogotá, Colombia; 629 University of Abidjan, Abidjan, Côte d’Ivoire; 630 Pirogov Russian National Research Medical University, Moscow, Russia; 631 Universidade de Lisboa, Lisbon, Portugal; 632 Saveetha Dental College and Hospitals, Chennai, India; 633 Democritus University, Alexandroupolis, Greece; 634 Nutrition and Endocrine Research Center, Tehran, Iran; 635 Grigore T Popa University of Medicine and Pharmacy, Iasi, Romania; 636 Instituto Nacional de Saúde, Marracuene, Mozambique; 637 Università degli Studi di Firenze, Florence, Italy; 638 Psychiatry and Psychology Research Center, Tehran, Iran; 639 Isfahan Cardiovascular Research Center, Isfahan, Iran; 640 Universiti Putra Malaysia, Serdang, Malaysia; 641 University Medical School of Pécs, Pécs, Hungary; 642 Mulago Hospital, Kampala, Uganda; 643 Instituto Costarricense de Investigación y Enseñanza en Nutrición y Salud, San José, Costa Rica; 644 Universidad de San Carlos, Guatemala City, Guatemala; 645 Gorgas Memorial Institute for Studies of Health, Panama City, Panama; 646 University of Limpopo, Sovenga, South Africa; 647 Seoul National University, Seoul, Republic of Korea; 648 University of Medical Sciences of Cienfuegos, Cienfuegos, Cuba; 649 Ministry of Health and Wellness, Belmopan, Belize; 650 La Trobe University, Melbourne, Victoria, Australia; 651 Sabzevar University of Medical Sciences, Sabzevar, Iran; 652 International Institute of Molecular and Cell Biology, Warsaw, Poland; 653 Ain Shams University, Cairo, Egypt; 654 University of Medicine and Pharmacy Craiova, Craiova, Romania; 655 Instituto Conmemorativo Gorgas de Estudios de la Salud, Panama City, Panama; 656 World Health Organization Country Office, Lilongwe, Malawi; 657 Universidade de São Paulo, São Paulo, Brazil; 658 Department of Public Health, Nay Pyi Taw, Myanmar; 659 Albanian Sport Science Association, Tirana, Albania; 660 University of Brescia, Brescia, Italy; 661 Queen Mary University, London, UK; 662 University of the Balearic Islands, Palma de Mallorca, Spain; 663 University of Limerick, Limerick, Ireland; 664 Universiti Sains Malaysia, Kelantan, Malaysia; 665 Makerere University School of Public Health, Kampala, Uganda; 666 Bushehr University of Medical Sciences, Bushehr, Iran; 667 Ulm University, Ulm, Germany; 668 Jozef Pilsudski University of Physical Education in Warsaw, Warsaw, Poland; 669 Suraj Eye Institute, Nagpur, India; 670 UNICEF, Yaoundé, Cameroon; 671 Ministry of Health, Apia, Samoa; 672 Leuven University, Leuven, Belgium; 673 Public Health Center of the Ministry of Health, Kyiv, Ukraine; 674 National Institute of Hygiene and Epidemiology, Hanoi, Viet Nam; 675 University of Medicine and Pharmacy at Ho Chi Minh City, Ho Chi Minh City, Viet Nam; 676 Hanoi Medical University, Hanoi, Viet Nam; 677 Xi’an Jiaotong University, Xi’an, China; 678 Precision Care Clinic Corp, St. Cloud, FL USA; 679 Heartfile, Islamabad, Pakistan; 680 Eastern Mediterranean Public Health Network, Amman, Jordan; 681 University of Manchester, Manchester, UK; 682 Tachikawa General Hospital, Nagaoka, Japan; 683 University of Abuja College of Health Sciences, Abuja, Nigeria; 684 Korea Disease Control and Prevention Agency, Cheongju-si, Republic of Korea; 685 Kyoto Prefectural University of Medicine, Kyoto, Japan; 686 Japan Wildlife Research Center, Tokyo, Japan; 687 Gadarif University, Gadarif, Sudan; 688 Istanbul University, Istanbul, Türkiye; 689 Ministry of Health, Bandar Seri Begawan, Brunei; 690 University of Madeira, Funchal, Portugal; 691 University of Puerto Rico, San Juan, Puerto Rico; 692 Universidad de Santander, Bucaramanga, Colombia; 693 Kwame Nkrumah University of Science and Technology, Kumasi, Ghana; 694 University of Maiduguri, Maiduguri, Nigeria; 695 Arizona State University, Tempe, AZ USA; 696 University of Wisconsin-Madison, Madison, WI USA; 697 University Hospital Heidelberg, Heidelberg, Germany; 698 Qingdao Centers for Disease Control and Prevention, Qingdao, China; 699 University of Bari Aldo Moro, Bari, Italy; 700 University of Sharjah, Abu Dhabi, United Arab Emirates; 701 Catholic University of Daegu, Daegu, Republic of Korea; 702 Faculty of Medicine Ramathibodi Hospital, Maidol, Thailand; 703 Jivandeep Hospital, Anand, India; 704 Centro de Investigação em Saúde de Angola, Caxito, Angola; 705 Fundació Institut Universitari per a la recerca a l’Atenció Primària de Salut Jordi Gol i Gurina, Mataró, Spain; 706 National Dental Centre Singapore, Singapore, Singapore; 707 University Hospital of Varese, Varese, Italy; 708 Vietnam National Heart Institute, Hanoi, Viet Nam; 709 Ministry of Health Malawi, Lilongwe, Malawi; 710 Clínica de Medicina Avanzada Dr. Abel González, Santo Domingo, Dominican Republic; 711 Université de Limoges, Limoges, France; 712 Cardiovascular Prevention Centre Udine, Udine, Italy; 713 University of Pisa, Pisa, Italy; 714 Ministry of Health and Medical Services, Honiara, Solomon Islands; 715 National Institute of Cardiology, Warsaw, Poland; 716 College of Physicians of Barcelona, Barcelona, Spain; 717 O.M. Marzieiev Institute for Public Health of the National Academy of the Medical Sciences of Ukraine, Kyiv, Ukraine; 718 Universiti Kebangsaan Malaysia, Kuala Lumpur, Malaysia; 719 Ardabil University of Medical Sciences, Ardabil, Iran; 720 Public Health Foundation of India, New Delhi, India; 721 University of Edinburgh, Edinburgh, UK; 722 Universidade Pedagógica, Maputo, Mozambique; 723 Centre for Disease Prevention and Control, Riga, Latvia; 724 Sulaimani Polytechnic University, Sulaymaniyah, Iraq; 725 Boehringer Ingelheim, Ingelheim, Germany; 726 Alborz University of Medical Sciences, Karaj, Iran; 727 Cooperativa de Ensino Superior Politécnico e Universitário, Famalicão, Portugal; 728 Ministry of Health, Hanoi, Viet Nam; 729 National Agency for Public Health, Chisinau, Moldova; 730 University of Novi Sad Faculty of Medicine, Novi Sad, Serbia; 731 Pure Earth, Dhaka, Bangladesh; 732 Institute of Epidemiology Disease Control and Research, Dhaka, Bangladesh; 733 University of Turku, Turku, Finland; 734 UNICEF, Baku, Azerbaijan; 735 World Health Organization Country Office, Juba, South Sudan; 736 Instituto Federal Riograndense, Rio Grande, Brazil; 737 Institut Universitari d’Investigació en Atenció Primària Jordi Gol, Girona, Spain; 738 University of Girona, Girona, Spain; 739 Newcastle University, Newcastle, UK; 740 Institute for Health Policy, Colombo, Sri Lanka; 741 APDP Diabetes Portugal, Lisboa, Portugal; 742 Sotiria Hospital, Athens, Greece; 743 INCLIVA, Valencia, Spain; 744 Slovak Academy of Sciences, Bratislava, Slovakia; 745 University of Santa Cruz do Sul, Santa Cruz do Sul, Brazil; 746 Nutrition Research Foundation, Barcelona, Spain; 747 Minas Gerais State Secretariat for Health, Belo Horizonte, Brazil; 748 UB Bunyola Ibsalut, Palma, Spain; 749 Amsterdam Institute for Global Health and Development, Amsterdam, The Netherlands; 750 Universidade Nove de Julho, São Paulo, Brazil; 751 Ministerio de Salud, Panama City, Panama; 752 Canarian Health Service, Tenerife, Spain; 753 Universidad Industrial de Santander, Bucaramanga, Colombia; 754 Ministry of Health and Social Protection, Bogotá, Colombia; 755 Wuqu’ Kawoq, Tecpan, Guatemala; 756 GroundWork, Fläsch, Switzerland; 757 CIBERDEM, Málaga, Spain; 758 Instituto de Investigación Biomédica de Málaga-Plataforma Bionand, Málaga, Spain; 759 Fundación Instituto de Investigación Sanitaria Illes Baleares, Madrid, Spain; 760 University of Minho, Braga, Portugal; 761 Fiji National University, Suva, Fiji; 762 GHESKIO Clinics, Port-au-Prince, Haiti; 763 Universidad Galileo, Guatemala City, Guatemala; 764 Universidad de San Carlos, Quetzaltenango, Guatemala; 765 National Center of Epidemiology ISCIII, Madrid, Spain; 766 Rehamed-Center, Tajęcina, Poland; 767 Institute of Food Sciences of the National Research Council, Avellino, Italy; 768 Medical University of Gdańsk, Gdańsk, Poland; 769 Singapore Eye Research Institute, Singapore, Singapore; 770 Sitaram Bhartia Institute of Science and Research, New Delhi, India; 771 Academy of Preventive Medicine, Almaty, Kazakhstan; 772 Kindergarten of Avlonari, Evia, Greece; 773 National Institute of Health, Lima, Peru; 774 Ministry of Health, Jakarta, Indonesia; 775 Catalan Department of Health, Barcelona, Spain; 776 Biogipuzkoa Health Research Institute, San Sebastián, Spain; 777 Instituto de Saúde Ambiental, Lisbon, Portugal; 778 Universidade Federal de Alagoas, Maceió, Brazil; 779 The University of Health Sciences, Phnom Pen, Cambodia; 780 Wellbeing Services County of South Karelia, Lappeenranta, Finland; 781 Shizuoka Graduate University of Public Health, Shizuoka, Japan; 782 National Cancer Center, Tokyo, Japan; 783 University of São Paulo Clinics Hospital, São Paulo, Brazil; 784 Hospital Italiano de Buenos Aires, Buenos Aires, Argentina; 785 Medical University of Vienna, Vienna, Austria; 786 Nes Municipality, Nes, Norway; 787 German Institute of Human Nutrition Potsdam-Rehbruecke, Nuthetal, Germany; 788 The George Institute for Global Health, Sydney, New South Wales Australia; 789 Center for Oral Health Services and Research Mid-Norway, Trondheim, Norway; 790 Lagos State University College of Medicine, Lagos, Nigeria; 791 Comenius University, Bratislava, Slovakia; 792 Human Sciences Research Council, Cape Town, South Africa; 793 Medical Excellence JAPAN, Tokyo, Japan; 794 Teikyo University, Tokyo, Japan; 795 Israel Defense Forces Medical Corps, Jerusalem, Israel; 796 Rutgers University, New Brunswick, NJ USA; 797 Africa Health Research Institute, Durban, South Africa; 798 University of KwaZulu-Natal, Durban, South Africa; 799 St Vincent’s Hospital, Sydney, New South Wales Australia; 800 RAS FCTAS Institute of Social Demography, Moscow, Russia; 801 Health Polytechnic Jakarta II Institute, Jakarta, Indonesia; 802 Diponegoro University, Semarang, Indonesia; 803 National Medical Research Center for Children’s Health, Moscow, Russia; 804 University of Bordeaux, Bordeaux, France; 805 University of Hohenheim, Stuttgart, Germany; 806 Oslo Metropolitan University, Oslo, Norway; 807 Institute of Public Health, Skopje, North Macedonia; 808 Ss. Cyril and Methodius University, Skopje, North Macedonia; 809 Lamprecht und Stamm Sozialforschung und Beratung AG, Zurich, Switzerland; 810 Clinical Emergency Hospital, Bucharest, Romania; 811 Bonn University, Bonn, Germany; 812 National Institute of Public Health NIH — National Research Institute, Warsaw, Poland; 813 Kalina Malina Kindergarten, Pazardjik, Bulgaria; 814 Pontificia Universidad Javeriana Seccional Cali, Cali, Colombia; 815 Ubon Ratchathani University, Ubon Ratchathani, Thailand; 816 The Jikei University School of Medicine, Tokyo, Japan; 817 The Chinese University of Hong Kong, Hong Kong, China; 818 Mongolian Ecotoxicology Center, Ulaanbaatar, Mongolia; 819 University of Jordan, Amman, Jordan; 820 National Statistical Office, Praia, Cabo Verde; 821 St. Olavs University Hospital, Trondheim, Norway; 822 South Kazakhstan Medical Academy, Shymkent, Kazakhstan; 823 All Institute of Medical Sciences, New Delhi, India; 824 Scientific Research Institute of Maternal and Child Health, Ashgabat, Turkmenistan; 825 Ministry of Health, Amman, Jordan; 826 UNICEF, Niamey, Niger; 827 University of Applied Sciences Utrecht, Utrecht, The Netherlands; 828 Reproductive Endocrinology Research Center, Tehran, Iran; 829 Ethiopian Public Health Institute, Addis Ababa, Ethiopia; 830 Amrita Vishwa Vidyapeetham, Kochi, India; 831 CMC Vellore, Vellore, India; 832 University Medical Center Utrecht, Utrecht, The Netherlands; 833 Institut d’Investigacio Sanitaria Illes Balears, Menorca, Spain; 834 University of Vic-Central University of Catalonia, Vic, Spain; 835 University of Bologna, Bologna, Italy; 836 Hellenic Health Foundation, Athens, Greece; 837 Government Medical College, Bhavnagar, India; 838 Massachusetts General Hospital, Boston, MA USA; 839 Sefako Makgatho Health Sciences University, Ga-Rankuwa, South Africa; 840 Alexander Technological Educational Institute, Thessaloniki, Greece; 841 Tafuna Family Health Center, Tafuna, American Samoa; 842 LBJ Hospital, Faga’alu, American Samoa; 843 Addis Ababa University, Addis Ababa, Ethiopia; 844 Ministry of Health, Wellington, New Zealand; 845 Sheba Medical Center, Tel HaShomer, Israel; 846 Everyage Carolina Senior Care, Lexington, NC USA; 847 Universidad Centro-Occidental Lisandro Alvarado, Barquisimeto, Venezuela; 848 Meharry Medical College, Nashville, TN USA; 849 University of Tampere Tays Eye Center, Tampere, Finland; 850 Sabiha Gokcen Ilkokulu, Ankara, Türkiye; 851 Polytechnic Institute of Porto, Porto, Portugal; 852 Voronezh N.N. Burdenko State Medical University, Voronezh, Russia; 853 Icahn School of Medicine at Mount Sinai, New York City, NY USA; 854 George Washington University, Washington, DC USA; 855 MONICA FRIULI Study Group, Udine, Italy; 856 Universidad CEU San Pablo, Madrid, Spain; 857 Consejería de Sanidad Junta de Castilla y León, Valladolid, Spain; 858 Strasbourg University Hospital, Strasbourg, France; 859 National Research Council, Pisa, Italy; 860 Universidad San Francisco de Quito, Quito, Ecuador; 861 Ministry of Public Health, Nonthaburi, Thailand; 862 Sunflower Nursery School, Craiova, Romania; 863 North Karelia Center for Public Health, Joensuu, Finland; 864 Academy of Athens, Athens, Greece; 865 University of the Witwatersrand, Johannesburg, South Africa; 866 Institute for Medical Research, Kuala Lumpur, Malaysia; 867 Xinjiang Medical University, Urumqi, China; 868 Shanghai Educational Development Co. Ltd, Shanghai, China; 869 University of Cambridge, Cambridge, UK; 870 Mingsii Co. Ltd, Beijing, China; 871 Örebro University, Örebro, Sweden; 872 St George’s, University of London, London, UK; 873 Universitas Indonesia, Jakarta, Indonesia; 874 GroundWork, Geneva, Switzerland; 875 National Yang Ming Chiao Tung University, Taipei, Taiwan; 876 Institute of Food and Nutrition Development of Ministry of Agriculture and Rural Affairs, Beijing, China; 877 Beijing Institute of Ophthalmology, Beijing, China; 878 Weill Cornell Medicine, New York City, NY USA; 879 Children’s Hospital of Fudan University, Shanghai, China; 880 University of Cyprus, Nicosia, Cyprus; 881 Niigata University, Niigata, Japan; 882 South China Institute of Environmental Sciences, Guangzhou, China; 883 International Medical University, Shah Alam, Malaysia; 884 Hellenic Mediterranean University, Heraklion, Greece; 885 Iran University of Medical Sciences, Tehran, Iran; 886 Peking University First Hospital, Beijing, China; 887 West Kazakhstan Medical University, Aktobe, Kazakhstan; 888 Inner Mongolia Medical University, Hohhot, China; 889 Institute of Correctional Pedagogy, Moscow, Russia; 890 Przedszkole No. 81, Warsaw, Poland

**Keywords:** Obesity, Epidemiology

Correction to: *Nature* https://doi.org/10.1038/s41586-026-10383-0 Published online 13 May 2026

In the version of the article initially published, María Elena Díaz-Sánchez, Patricia Varona-Pérez and Ramón Suárez-Medina (all affiliated with the National Institute of Hygiene, Epidemiology and Microbiology, Havana, Cuba) were inadvertently omitted from the list of NCD Risk Factor Collaboration (NCD-RisC) members and have now been added in the HTML and PDF versions of the article.

