# Supplementary material for: Obesity rise plateaus in developed nations and accelerates in developing nations
Source: Nature. 2026 May 13;653(8114):510–8. doi: 10.1038/s41586-026-10383-0 (PMC13171639; doi:10.1038/s41586-026-10383-0)
Supplement: Supplementary file 1 — This PDF file contains Supplementary Notes, Supplementary Tables, Supplementary Figs. and Supplementary References. Supplementary Note 1. List of countries’ ISO 3166-1 alpha-3 codes. Supplementary Table 1. List of analysis regions and super-regions, and countries in each region. Supplementary Table 2. Data sources used in the analysis. Supplementary Table 3. Specification of the Bayesian hierarchical model. Supplementary Table 4. Results of model validation. Supplementary Table 5. Average Jaccard index of the clusters. Supplementary Fig. 1. Number of data sources used in the analysis, by country. Supplementary Fig. 2. Number of data sources used in the analysis, by region and year. Supplementary Fig. 3. Age-standardized prevalence of obesity in children and adolescents from 1980 to 2024 by country. Supplementary Fig. 4. Age-standardized prevalence of obesity in adults from 1980 to 2024 by country. Supplementary Fig. 5. Velocity of obesity in children and adolescents from 1980 to 2024 by country. Supplementary Fig. 6. Velocity of obesity in adults from 1980 to 2024 by country. [file 41586_2026_10383_MOESM1_ESM.pdf]

nature portfolio

https://doi.org/10.1038/s41586-026-10383-0

Supplementary information

Obesity rise plateaus in developed nations and accelerates in developing nations

In the format provided by the authors and unedited

Supplementary Note 1. List of countries' ISO 3166-1 alpha-3 codes.

AFG=Afghanistan. AGO=Angola. ALB=Albania. AND=Andorra. ARE=United Arab Emirates. ARG=Argentina. ARM=Armenia. ASM=American Samoa. ATG=Antigua and Barbuda. AUS=Australia. AUT=Austria. AZE=Azerbaijan. BDI=Burundi. BEL=Belgium. BEN=Benin. BFA=Burkina Faso. BGD=Bangladesh. BGR=Bulgaria. BHR=Bahrain. BHS=The Bahamas. BIH=Bosnia and Herzegovina. BLR=Belarus. BLZ=Belize. BMU=Bermuda. BOL=Bolivia. BRA=Brazil. BRB=Barbados. BRN=Brunei. BTN=Bhutan. BWA=Botswana. CAF=Central African Republic. CAN=Canada. CHE=Switzerland. CHL=Chile. CHN=China. CIV=Côte d'Ivoire. CMR=Cameroon. COD=DR Congo. COG=Congo. COK=Cook Islands. COL=Colombia. COM=Comoros. CPV=Cabo Verde. CRI=Costa Rica. CUB=Cuba. CYP=Cyprus. CZE=Czechia. DEU=Germany. DJI=Djibouti. DMA=Dominica. DNK=Denmark. DOM=Dominican Republic. DZA=Algeria. ECU=Ecuador. EGY=Egypt. ERI=Eritrea. ESP=Spain. EST=Estonia. ETH=Ethiopia. FIN=Finland. FJI=Fiji. FRA=France. FSM=Federated States of Micronesia. GAB=Gabon. GBR=UK. GEO=Georgia. GHA=Ghana. GIN=Guinea. GMB=The Gambia. GNB=Guinea-Bissau. GNQ=Equatorial Guinea. GRC=Greece. GRD=Grenada. GRL=Greenland. GTM=Guatemala. GUY=Guyana. HND=Honduras. HRV=Croatia. HTI=Haiti. HUN=Hungary. IDN=Indonesia. IND=India. IRL=Ireland. IRN=Iran. IRQ=Iraq. ISL=Iceland. ISR=Israel. ITA=Italy. JAM=Jamaica. JOR=Jordan. JPN=Japan. KAZ=Kazakhstan. KEN=Kenya. KGZ=Kyrgyzstan. KHM=Cambodia. KIR=Kiribati. KNA=Saint Kitts and Nevis. KOR=South Korea. KWT=Kuwait. LAO=Laos. LBN=Lebanon. LBR=Liberia. LBY=Libya. LCA=Saint Lucia. LKA=Sri Lanka. LSO=Lesotho. LTU=Lithuania. LUX=Luxembourg. LVA=Latvia. MAR=Morocco. MDA=Moldova. MDG=Madagascar. MDV=Maldives. MEX=Mexico. MHL=Marshall Islands. MKD=North Macedonia. MLI=Mali. MLT=Malta. MMR=Myanmar. MNE=Montenegro. MNG=Mongolia. MOZ=Mozambique. MRT=Mauritania. MUS=Mauritius. MWI=Malawi. MYS=Malaysia. NAM=Namibia. NER=Niger. NGA=Nigeria. NIC=Nicaragua. NIU=Niue. NLD=Netherlands. NOR=Norway. NPL=Nepal. NRU=Nauru. NZL=New Zealand.

OMN=Oman. PAK=Pakistan. PAN=Panama. PER=Peru. PHL=Philippines. PLW=Palau. PNG=Papua New Guinea. POL=Poland. PRI=Puerto Rico. PRK=North Korea. PRT=Portugal. PRY=Paraguay. PSE=Palestine. PYF=French Polynesia. QAT=Qatar. ROU=Romania. RUS=Russia. RWA=Rwanda. SAU=Saudi Arabia. SDN=Sudan. SEN=Senegal. SGP=Singapore. SLB=Solomon Islands. SLE=Sierra Leone. SLV=El Salvador. SOM=Somalia. SRB=Serbia. SSD=South Sudan. STP=São Tomé and Príncipe. SUR=Suriname. SVK=Slovakia. SVN=Slovenia. SWE=Sweden. SWZ=Eswatini. SYC=Seychelles. SYR=Syria. TCD=Chad. TGO=Togo. THA=Thailand. TJK=Tajikistan. TKL=Tokelau. TKM=Turkmenistan. TLS=Timor-Leste. TON=Tonga. TTO=Trinidad and Tobago. TUN=Tunisia. TUR=Türkiye. TUV=Tuvalu. TWN=Taiwan. TZA=Tanzania. UGA=Uganda. UKR=Ukraine. URY=Uruguay. USA=USA. UZB=Uzbekistan. VCT=Saint Vincent and the Grenadines. VEN=Venezuela. VNM=Viet Nam. VUT=Vanuatu. WSM=Samoa. YEM=Yemen. ZAF=South Africa. ZMB=Zambia. ZWE=Zimbabwe.

Supplementary Table 1. List of analysis regions and super-regions, and countries in each region.

Super-region	Region
**Central and eastern Europe**	**Central Europe**: Albania, Bosnia and Herzegovina, Bulgaria, Croatia, Czechia, Hungary, Montenegro, North Macedonia, Poland, Romania, Serbia, Slovakia, Slovenia
	**Eastern Europe**: Belarus, Estonia, Latvia, Lithuania, Moldova, Russian Federation, Ukraine
**Central Asia, Middle East and north Africa**	**Central Asia**: Armenia, Azerbaijan, Georgia, Kazakhstan, Kyrgyzstan, Mongolia, Tajikistan, Turkmenistan, Uzbekistan
	**Middle East and north Africa**: Algeria, Bahrain, Egypt, Iran, Iraq, Jordan, Kuwait, Lebanon, Libya, Morocco, Oman, Qatar, Saudi Arabia, State of Palestine, Syrian Arab Republic, Tunisia, Türkiye, United Arab Emirates, Yemen
**East and southeast Asia**	**East Asia**: China, Japan, Singapore, South Korea, Taiwan
	**Southeast Asia**: Brunei Darussalam, Cambodia, Indonesia, Lao PDR, Malaysia, Maldives, Myanmar, North Korea, Philippines, Thailand, Timor-Leste, Viet Nam
**High-income western**	**High-income English-speaking countries***: Australia, Canada, Ireland, New Zealand, United Kingdom, United States of America
	**Northwestern Europe**: Austria, Belgium, Denmark, Finland, Germany, Greenland, Iceland, Luxembourg, Netherlands, Norway, Sweden, Switzerland
	**Southwestern Europe**: Andorra, Cyprus, France, Greece, Israel, Italy, Malta, Portugal, Spain
**Latin America and the Caribbean**	**Andean Latin America**: Bolivia, Ecuador, Peru
	**Central Latin America**: Colombia, Costa Rica, El Salvador, Guatemala, Honduras, Mexico, Nicaragua, Panama, Venezuela
	**Southern Latin America**: Argentina, Brazil, Chile, Paraguay, Uruguay
	**The Caribbean**: Antigua and Barbuda, Bahamas, Barbados, Belize, Bermuda, Cuba, Dominica, Dominican Republic, Grenada, Guyana, Haiti, Jamaica, Puerto Rico, Saint Kitts and Nevis, Saint Lucia, Saint Vincent and the Grenadines, Suriname, Trinidad and Tobago
**Pacific island nations**	**Melanesia:** Fiji, Papua New Guinea, Solomon Islands, Vanuatu
	**Polynesia and Micronesia**: American Samoa, Cook Islands, French Polynesia, Kiribati, Marshall Islands, Micronesia (Federated States of), Nauru, Niue, Palau, Samoa, Tokelau, Tonga, Tuvalu
**South Asia**	**South Asia**: Afghanistan, Bangladesh, Bhutan, India, Nepal, Pakistan, Sri Lanka

**Sub-Saharan Africa**	**Central Africa and southern Africa**: Angola, Botswana, Central African Republic, Congo, DR Congo, Equatorial Guinea, Gabon, Namibia
	**East Africa**: Burundi, Comoros, Djibouti, Eritrea, Eswatini, Ethiopia, Kenya, Lesotho, Madagascar, Malawi, Mozambique, Rwanda, Somalia, South Sudan, Sudan, Tanzania, Uganda, Zambia, Zimbabwe
	**West Africa**: Benin, Burkina Faso, Cabo Verde, Cameroon, Chad, Cote d'Ivoire, Gambia, Ghana, Guinea, Guinea Bissau, Liberia, Mali, Mauritania, Niger, Nigeria, Sao Tome and Principe, Senegal, Sierra Leone, Togo
	**Other sub-Saharan Africa**: Mauritius, Seychelles, South Africa

* Although high-income English-speaking countries are geographically separated, they experienced similar trends in cardiometabolic risk factors and outcomes[1-8]. They were therefore grouped together so that the statistical model shares information amongst them more than it does with other countries that are geographically closer but epidemiologically more distinct.

Supplementary Table 2. Data sources used in the analysis.

	Country	Study years	Survey/Study name/Citation	Level of representative-ness	Rural, urban, or both	Age range as in NCD-RisC database		Sample size		Note
						Male	Female	Male	Female	
1	Afghanistan	2010-2011	Afghanistan Multiple Indicator Cluster Survey	National	both		15-49		8592	
2	Afghanistan	2013	National Nutrition Survey	National	both		10-49		18433	
3	Afghanistan	2013	Afghanistan Non-Communicable Diseases (NCD) Risk Factors Survey (Jalalabad)	Community	urban	25-70	25-70	434	576	
4	Afghanistan	2015	Afghanistan Non-Communicable Diseases (NCD) Risk Factors Survey (Herat)	Community	urban	25-70	25-70	530	575	
5	Afghanistan	2015	Afghanistan Non-Communicable Diseases (NCD) Risk Factors Survey (Kabul)	Community	urban	25-70	25-70	564	565	
6	Afghanistan	2015	Afghanistan Non-Communicable Diseases (NCD) Risk Factors Survey (Kandahar)	Community	urban	25-70	25-70	561	589	
7	Afghanistan	2015	Afghanistan Non-Communicable Diseases (NCD) Risk Factors Survey (Mazar)	Community	urban	25-70	25-70	567	617	
8	Afghanistan	2018	STEPS	National	both	18-69	18-69	1984	1703	
9	Albania	2001	Shapo et al., Public Health Nutr 6:471-77, 2003	Community	urban	24+	24+	535	585	
10	Albania	2008-2009	DHS	National	both	15-49	15-49	2978	7386	
11	Albania	2013	Childhood Obesity Surveillance Initiative 3	National	both	7-9	7-9	2971	2794	
12	Albania	2013-2015	Balkan Survey of Inactivity in Children (BASIC)	National	both	6-16	6-16	4978	4972	
13	Albania	2015-2016	Childhood Obesity Surveillance Initiative 4	National	both	7-9	7-9	3358	3062	
14	Albania	2017-2018	DHS	National	both	15-59	15-59	5953	14447	
15	Albania	2022	Childhood Obesity Surveillance Initiative 6	National	both	7-8	7-8	2748	2649	
16	Albania	2022-2023	EUFITMOS project measurement (2022-2023)	Subnational	both	6-18	6-18	1277	1334	
17	Algeria	2003	STEPS	Subnational	both	25-64	25-64	1612	2437	
18	Algeria	2005	Transition and Health Impact in North Africa	National	both	35-70	35-70	2004	2741	
19	Algeria	2007-2009	The ISOR (InSulino-resistance in ORan) Study	Community	urban	30-64	30-64	376	408	
20	Algeria	2016-2017	STEPS	National	both	18-69	18-69	2991	3636	
21	American Samoa	1976-1978	McGarvey, Am J Clin Nutr 53(6 Suppl):1586S-1594S, 1991	National	both	5+	5+	1017	1329	1
22	American Samoa	1990	McGarvey, Pac Health Dialog 8(1):157-62, 2001	National	both	25+	25+	359	484	
23	American Samoa	1992	McGarvey, Pac Health Dialog 8(1):157-62, 2001	National	both	27+	27+	232	337	
24	American Samoa	1994	McGarvey, Pac Health Dialog 8(1):157-62, 2001	National	both	29+	29+	165	245	
25	American Samoa	2004	STEPS	National	both	25-64	25-64	949	1060	
26	Andorra	2004-2005	Enquesta Nutricional D'Andorra	National	both	18-75	18-75	400	447	
27	Angola	2013-2014	CardioBengo - Population based cardiovascular longitudinal study in Bengo Province, Angola	Community	both	14-65	14-65	875	1486	
28	Antigua and Barbuda	2009	Global School-based Student Health Survey	National	both	13-17	13-17	70	122	
29	Argentina	1981-1985	Hernandez et al., Diabetes Res Clin Pract 3:277-83, 1987	Community	urban	20-74	20-74	395	414	
30	Argentina	1985-1986	INTERSALT	Community	urban	20-59	20-59	100	100	
31	Argentina	1995-1998	de Sereday et al., Diabetes Metab 30:335-9, 2004	Subnational	urban	15-74	15-74	924	1246	
32	Argentina	2003	CEDES-Programa VIGI+A-Banco Mundial, 2004	Community	urban	15-74	15-74	151	176	
33	Argentina	2004-2005	CArdiovascular Risk factors Multiple Evaluation in Latin America (CARMELA)	Community	urban	25-64	25-64	733	742	
34	Argentina	2005	Encuesta Nacional de Nutrición y Salud 2005	National	both		10-49		6581	
35	Argentina	2006	Virasoro Survey	Community	urban	15-84	15-84	261	306	
36	Argentina	2008-2011	The VELA Project	Community	rural	5+	5+	380	543	
37	Argentina	2011	Primera Encuesta Alimentaria y Nutricional de la Ciudad Autónoma de Buenos Aires - EANCABA	Community	urban	5-18; 60+	5-49; 60+	1173	2229	
38	Argentina	2011-2012	CESCAS Study	Community	urban	35-74	35-74	1571	2359	
39	Argentina	2012-2013	Primer estudio sobre el estado nutricional y los hábitos alimentarios de la población adulta de Rosario	Community	urban	18-70	18-70	371	823	
40	Argentina	2014-2015	Latin American Study of Nutrition and Health (ELANS)	National	urban	15-65	15-65	573	693	
41	Argentina	2018	Encuesta Nacional de Factores de Riesgo 2018	National	both	18+	18+	6960	9449	
42	Argentina	2018-2019	Encuesta Nacional de Nutricion y Salud	National	urban	5+	5+	6482	7332	
43	Armenia	1998	The health and nutritional status of children and women in Armenia	National	both		18-45		2420	
44	Armenia	2000	DHS	National	both		15-49		5982	
45	Armenia	2005	DHS	National	both	15-49	15-49	1160	6123	
46	Armenia	2015-2016	DHS	National	both		15-49		5731	
47	Armenia	2016	STEPS	National	both	18-69	18-69	604	1447	
48	Armenia	2019	Childhood Obesity Surveillance Initiative 5	National	both	7-8	7-8	1860	1703	
49	Australia	1980	Risk Factor Prevalence Study	National	urban	25-64	25-64	2756	2781	
50	Australia	1981	Busselton Health Study	Community	urban	18+	18+	225	290	
51	Australia	1983	Risk Factor Prevalence Study	National	urban	25-64	25-64	3731	3813	
52	Australia	1983	MONICA, Newcastle	Subnational	urban	35-64	35-64	1215	1244	
53	Australia	1985	Australian Schools Health and Fitness Survey (ASHFS)	National	both	7-15	7-15	4302	4189	
54	Australia	1988-1989	Dubbo Study of Australian Elderly	Community	urban	59+	59+	877	1219	
55	Australia	1988-1989	MONICA, Newcastle	Subnational	urban	35-64	35-64	672	671	
56	Australia	1988-1989	MONICA, Newcastle	Community	urban	25-34	25-34	70	84	
57	Australia	1989	Risk Factor Prevalence Study	National	urban	20-69	20-69	4497	4678	
58	Australia	1992-1993	Australia Longitudinal Study of Ageing	Community	urban	65+	65+	814	746	
59	Australia	1994	MONICA, Newcastle	Subnational	urban	35-64	35-64	637	688	
60	Australia	1994	MONICA, Perth inner	Community	urban	25-64	25-64	363	349	
61	Australia	1994	MONICA, Perth outer	Community	urban	25-64	25-64	373	387	
62	Australia	1995	National Nutrition Survey 1995	National	both	5+	5+	5983	6390	
63	Australia	1996	The Nepean Longitudinal Cohort Study	Community	urban	7-8	7-8	221	215	

	Country	Study years	Survey/Study name/Citation	Level of representative-ness	Rural, urban, or both	Age range as in NCD-RisC database		Sample size		Note
						Male	Female	Male	Female	
64	Australia	1996-1998	Western Australian AAA Screening Program	Community	urban	65-84		12194		
65	Australia	1997	Australia New South Wales Schools Fitness and Physical Activity Survey	Subnational	both	7-16	7-16	2883	2505	
66	Australia	1997	South Australia Schools Fitness and Physical Activity Survey	Subnational	both	10-12	10-12	921	765	
67	Australia	1999-2000	The Australian Diabetes, Obesity and Lifestyle Study 1999-2000	National	both	25+	25+	4991	6070	
68	Australia	1999-2003	North West Adelaide Health Study	Community	urban	18+	18+	1932	2122	
69	Australia	2000	Perth children	Community	both	25	25	266	334	
70	Australia	2004	The Longitudinal Study of Australian Children, K cohort (child)	National	both	5	5	425	431	
71	Australia	2004	The Nepean Longitudinal Cohort Study	Community	urban	14-15	14-15	143	149	
72	Australia	2004	NSW schools physical activity and nutrition survey	Subnational	both	5-16	5-16	2708	2567	
73	Australia	2004-2005	The Australian Diabetes, Obesity and Lifestyle Study 2004-2005	National	both	30+	30+	2874	3472	
74	Australia	2004-2005	Janus et al., Med J Aust 187:147-52, 2007	Community	rural	25-74	25-74	381	421	
75	Australia	2004-2006	North West Adelaide Health Study	Community	urban	20+	20+	1523	1679	
76	Australia	2006	The Longitudinal Study of Australian Children, K cohort (child)	National	both	6-7	6-7	2245	2156	
77	Australia	2007	Children's Nutrition and Physical Activity Survey	National	both	5-16	5-16	1649	1669	
78	Australia	2007-2008	National Health Survey	National	both	18+	18+	5279	5655	
79	Australia	2008	The Longitudinal Study of Australian Children, B cohort (infant)	National	both	5	5	508	513	
80	Australia	2008	The Longitudinal Study of Australian Children, K cohort (child)	National	both	8-9	8-9	2121	2023	
81	Australia	2008-2010	North West Adelaide Health Study	Community	urban	24+	24+	1168	1318	
82	Australia	2010	The Longitudinal Study of Australian Children, B cohort (infant)	National	both	6-7	6-7	2148	2014	
83	Australia	2010	The Longitudinal Study of Australian Children, K cohort (child)	National	both	10-11	10-11	2052	1945	
84	Australia	2010	NSW schools physical activity and nutrition survey	Subnational	both	5-16	5-16	4006	3664	2
85	Australia	2011-2013	International Study of Childhood Obesity, Lifestyle and the Environment (ISCOLE)	Community	urban	9-11	9-11	243	285	
86	Australia	2011-2013	Australian Health Survey	National	both	5+	5+	12190	13011	3
87	Australia	2012	The Longitudinal Study of Australian Children, B cohort (infant)	National	both	8-9	8-9	2041	1947	
88	Australia	2012	The Longitudinal Study of Australian Children, K cohort (child)	National	both	12-13	12-13	1948	1851	
89	Australia	2012	The Australian Diabetes, Obesity and Lifestyle Study 2012	National	both	37+	37+	2048	2530	
90	Australia	2014	The Longitudinal Study of Australian Children, B cohort (infant)	National	both	10-11	10-11	1829	1742	
91	Australia	2014	The Longitudinal Study of Australian Children, K cohort (child)	National	both	14-15	14-15	1694	1581	
92	Australia	2014-2015	National Health Survey	National	both	5+	5+	8331	9424	
93	Australia	2015	NSW schools physical activity and nutrition survey	Subnational	both	5-16	5-16	3509	3686	2
94	Australia	2016	The Longitudinal Study of Australian Children, B cohort (infant)	National	both	12-13	12-13	1639	1529	
95	Australia	2016	The Longitudinal Study of Australian Children, K cohort (child)	National	both	16-17	16-17	1477	1380	
96	Australia	2017-2018	National Health Survey	National	both	18+	18+	7576	8729	
97	Australia	2018	The Longitudinal Study of Australian Children, B cohort (infant)	National	both	14-15	14-15	1523	1403	
98	Australia	2018	The Longitudinal Study of Australian Children, K cohort (child)	National	both	18-19	18-19	1304	1233	
99	Austria	1983	The Austrian Conscription Database	National	both	17-18		58517		
100	Austria	1984	The Austrian Conscription Database	National	both	17-18		58830		
101	Austria	1985	The Austrian Conscription Database	National	both	17-18		58638		
102	Austria	1986	The Austrian Conscription Database	National	both	17-18		57870		
103	Austria	1986	CINDI	Community	both	25-64	25-64	657	715	
104	Austria	1987	The Austrian Conscription Database	National	both	17-18		55292		
105	Austria	1988	The Austrian Conscription Database	National	both	17-18		51050		
106	Austria	1989	The Austrian Conscription Database	National	both	17-18		49380		
107	Austria	1990	The Austrian Conscription Database	National	both	17-18		47102		
108	Austria	1991	The Austrian Conscription Database	National	both	17-18		44288		
109	Austria	1991	CINDI survey Vorarlberg/Austria	Subnational	both	25-64	25-64	698	738	
110	Austria	1992	The Austrian Conscription Database	National	both	17-18		43553		
111	Austria	1992	Vorarlberg Health Monitoring and Promotion Programme (VHM&PP)	Subnational	both	18+	18+	14161	18835	
112	Austria	1993	The Austrian Conscription Database	National	both	17-18		42356		
113	Austria	1994	The Austrian Conscription Database	National	both	17-18		40401		
114	Austria	1995	The Austrian Conscription Database	National	both	17-18		39809		
115	Austria	1996	The Austrian Conscription Database	National	both	17-18		39587		
116	Austria	1997	The Austrian Conscription Database	National	both	17-18		40408		
117	Austria	1998	The Austrian Conscription Database	National	both	17-18		43131		
118	Austria	1998	Vorarlberg Health Monitoring and Promotion Programme (VHM&PP)	Subnational	both	18+	18+	16153	20915	
119	Austria	1998-1999	CINDI survey Vorarlberg/Austria	Subnational	both	25-64	25-64	409	414	
120	Austria	1999	The Austrian Conscription Database	National	both	17-18		44163		
121	Austria	2000	The Austrian Conscription Database	National	both	17-18		44275		
122	Austria	2001	The Austrian Conscription Database	National	both	17-18		43315		
123	Austria	2002	The Austrian Conscription Database	National	both	17-18		42899		
124	Austria	2003	The Austrian Conscription Database	National	both	17-18		42389		
125	Austria	2004	The Austrian Conscription Database	National	both	17-18		42774		
126	Austria	2004	Vorarlberg Health Monitoring and Promotion Programme (VHM&PP)	Subnational	both	18+	18+	20160	23893	

	Country	Study years	Survey/Study name/Citation	Level of representative-ness	Rural, urban, or both	Age range as in NCD-RisC database		Sample size		Note
						Male	Female	Male	Female	
127	Austria	2004-2005	Vorarlberg Health Monitoring and Promotion Programme (VHM&PP)	Subnational	both	6-17	6-17	17504	15823	
128	Austria	2005	The Austrian Conscription Database	National	both	17-18		42906		
129	Austria	2006	The Austrian Conscription Database	National	both	17-18		44571		
130	Austria	2006-2007	HELENA	Community	urban	12-17	12-17	191	211	
131	Austria	2007	The Austrian Conscription Database	National	both	17-18		44755		
132	Austria	2008	The Austrian Conscription Database	National	both	17-18		44880		
133	Austria	2008-2009	Vorarlberg Health Monitoring and Promotion Programme (VHM&PP)	Subnational	both	6-17	6-17	16847	15295	
134	Austria	2009	The Austrian Conscription Database	National	both	17-18		45594		
135	Austria	2009-2011	Mayer et al., Ann Hum Biol 42(1):45-55, 2015	National	both	5-17	5-17	6634	6340	
136	Austria	2010	The Austrian Conscription Database	National	both	17-18		44968		
137	Austria	2010-2012	Austrian Study on Nutritional Status 2012	National	both	6-80	6-80	363	446	
138	Austria	2011	The Austrian Conscription Database	National	both	17-18		44076		
139	Austria	2011-2012	BMI in Upper Austrian children and adolescents	Subnational	both	6-17	6-17	7808	6853	
140	Austria	2012	The Austrian Conscription Database	National	both	17-18		42768		
141	Austria	2012-2013	Vorarlberg Health Monitoring and Promotion Programme (VHM&PP)	Subnational	both	6-17	6-17	14760	13648	
142	Austria	2013	The Austrian Conscription Database	National	both	17-18		41574		
143	Austria	2013-2014	Prevalence of obesity and motor performance in Tyrolean preschool children	Subnational	both	5	5	550	513	
144	Austria	2013-2017	Austrian Study on Nutrition (ASN)	National	both	19-64	19-64	774	1332	
145	Austria	2014	The Austrian Conscription Database	National	both	17-18		41740		
146	Austria	2014-2015	Influence of selected risk factors on the motor performance of 10 to 11-year-old schoolchildren	Subnational	both	10-11	10-11	197	129	
147	Austria	2015	The Austrian Conscription Database	National	both	17-18		39154		
148	Austria	2015-2016	Childhood Obesity Surveillance Initiative 4	National	both	8-9	8-9	1220	1175	
149	Austria	2015-2016	Vorarlberg Health Monitoring and Promotion Programme (VHM&PP)	Subnational	both	6-17	6-17	13037	12048	
150	Austria	2015-2017	The Tyrolean Early Vascular Ageing-study (EVA-Tyrol) - East-Tyrol	Subnational	both	14-17	14-17	680	831	
151	Austria	2016	The Austrian Conscription Database	National	both	17-19		35944		
152	Austria	2017	The Austrian Conscription Database	National	both	17-19		34399		
153	Austria	2018	The Austrian Conscription Database	National	both	17-19		34188		
154	Austria	2019	Fitness and health status of primary school children - before and during the COVID-19 pandemic	Community	both	7-10	7-10	381	382	
155	Austria	2019	The Austrian Conscription Database	National	both	17-19		32185		
156	Austria	2019	Childhood Obesity Surveillance Initiative 5	National	both	8-9	8-9	1223	1137	
157	Austria	2020	Fitness and health status of primary school children - before and during the COVID-19 pandemic - June	Community	both	7-10	7-10	381	383	
158	Austria	2020	Fitness and health status of primary school children - before and during the COVID-19 pandemic - September	Community	both	8-11	8-11	380	382	
159	Austria	2020	The Austrian Conscription Database	National	both	17-18		27323		
160	Austria	2021	Fitness and health status of primary school children - before and during the COVID-19 pandemic	Community	both	8-11	8-11	374	372	
161	Austria	2021	The Austrian Conscription Database	National	both	17-18		39111		
162	Austria	2021-2022	Early Vascular Ageing in the YOUth (EVA4YOU)	Community	both	14-17	14-17	373	665	
163	Austria	2022	Childhood Obesity Surveillance Initiative 6	National	both	8-9	8-9	1250	1214	
164	Austria	2023	Austrian Balance Test in Klagenfurt City	Community	both	6-14	6-14	296	220	
165	Azerbaijan	1996	Health and Nutrition Survey	National	both	19-59	19-59	121	295	
166	Azerbaijan	2001	Reproductive Health Survey	National	both		15-44		1726	
167	Azerbaijan	2006	DHS	National	both	15-59	15-49	2388	7868	
168	Azerbaijan	2013	Azerbaijan Nutrition Survey	National	both		15-49		2839	
169	Azerbaijan	2017	STEPS	National	both	18-69	18-69	1117	1577	
170	Azerbaijan	2022	Childhood Obesity Surveillance Initiative 6	National	both	8	8	1527	1300	
171	Bahamas	2011-2012	STEPS	National	both	25-64	25-64	586	938	
172	Bahamas	2013	Global School-based Student Health Survey	National	both	13-17	13-17	460	533	
173	Bahamas	2019	STEPS	National	both	18-69	18-69	862	1317	
174	Bahrain	1991-1992	Al-Mannai et al., J R Soc Health 116:30-2, 7-40, 1996	Community	both	20+	20+	137	153	
175	Bahrain	1995	Musaiger et al., Ann Hum Biol 28:346-50, 2001	Community	both	30+	30+	298	216	
176	Bahrain	1998-1999	National Nutrition Survey	National	both	19+	19+	1120	1181	
177	Bahrain	2001-2004	Global database on growth and malnutrition of school children and adolescents, WHO	National	both	6-19	6-20	1268	1326	
178	Bahrain	2007	STEPS	National	both	20-64	20-64	854	858	
179	Bahrain	2016	Global School-based Student Health Survey	National	both	12-17	12-17	3416	3262	
180	Bangladesh	1992	Rahman et al., Hypertension 33:74-8, 1999	Community	rural	30+	30+	965	643	
181	Bangladesh	1996-1997	DHS	National	both		20-49		3384	
182	Bangladesh	1998	Zaman et al., J Health Popul Nutr 21:162-63, 2003	Community	rural	20+	20+	290	379	
183	Bangladesh	1999-2000	DHS	National	both		20-49		3887	
184	Bangladesh	1999-2000	Hussain et al., Eur J Public Health, 17:291-96, 2007	Community	rural	20-59	20-59	2037	2720	
185	Bangladesh	2000-2004	Nutritional Surveillance Project	National	rural		15-45		224251	
186	Bangladesh	2002	STEPS	Community	rural	25-64	25-64	2086	2038	
187	Bangladesh	2002	STEPS	Community	urban	25-64	25-64	3533	3737	
188	Bangladesh	2004	DHS	National	both		20-49		9165	
189	Bangladesh	2006	Urban Health Survey	Subnational	urban	20-59	20-59	6109	5898	

	Country	Study years	Survey/Study name/Citation	Level of representative-ness	Rural, urban, or both	Age range as in NCD-RisC database		Sample size		Note
						Male	Female	Male	Female	
190	Bangladesh	2007	DHS	National	both		20-49		9037	
191	Bangladesh	2009-2010	STEPS	National	both	25+	25+	4310	4849	
192	Bangladesh	2011	DHS	National	both	15+	20+	5254	16679	
193	Bangladesh	2011-2012	Chronic Disease Risk Factor Study	Community	rural	14+	14+	336	483	
194	Bangladesh	2013	STEPS	National	both	25+	25+	1812	2261	
195	Bangladesh	2014	DHS	National	both		20-49		14963	
196	Bangladesh	2015-2016	An Assessment of BRAC Health Nutrition and Population Programme and Benchmark Survey of Sustainable Development Goal - 2015	National	rural	35+	11+	5432	18378	
197	Bangladesh	2016	Diabetes Mellitus: Action through community Groups or Health Information for better Control of population blood glucose, risk factors, knowledge and care seeking (DMagic)	Subnational	rural	30+	30+	5630	6414	
198	Bangladesh	2017-2018	DHS	National	both		15-49		17073	
199	Bangladesh	2018	STEPS	National	both	18-69	18-69	3784	4229	
200	Bangladesh	2018-2019	National Nutrition Surveillence	National	both	10+	10+	12212	12104	
201	Bangladesh	2019-2020	Bangladesh Adolescent Health and Wellbeing Survey	National	both	15-19	15-19	2720	5884	
202	Bangladesh	2022	DHS	National	both	15+	15+	4104	8767	
203	Bangladesh	2022-2023	STEPS	National	both	18-69	18-69	3347	3554	
204	Barbados	1987-1992	Barbados Eye Study	National	both	40-84	40-84	1980	2627	
205	Barbados	1991-1994	Cooper et al., Am J Public Health 87(2):160-68, 1997	Community	urban	25-100	25-100	329	482	
206	Barbados	1997-2002	The Barbados Incidence Studies of Eye Diseases II	National	both	40-84	40-84	1004	1441	
207	Barbados	1999-2000	The Survey on Health, Well-Being, and Aging in Latin America and the Caribbean (SABE)	Community	urban	60+	60+	559	866	4
208	Barbados	2011	Global School-based Student Health Survey	National	both	13-17	13-17	627	708	
209	Barbados	2011-2013	Health of the Nation (HotN)	National	both	25+	25+	455	703	
210	Belarus	2016-2017	STEPS	National	both	18-69	18-69	2085	2894	
211	Belarus	2020	STEPS	National	both	18-69	18-69	2271	2978	
212	Belgium	1983-1985	MONICA, Luxembourg	Community	urban	35-64	35-64	944	936	
213	Belgium	1984-1985	Belgian Interuniversity Research on Nutrition and Health	National	both	25-74	25-74	5837	5242	
214	Belgium	1985-1986	INTERSALT, Ghent	Community	urban	20-59	20-59	100	100	
215	Belgium	1985-1987	INTERSALT, Charleroi	Community	urban	20-59	20-59	82	75	
216	Belgium	1985-1987	MONICA, Charleroi	Community	urban	25-64	25-64	347	327	
217	Belgium	1985-1987	MONICA, Ghent	Community	urban	25-64	25-64	549	459	
218	Belgium	1985-1990	Flemish Study on Environment, Genes and Health Outcomes	Community	rural	20+	20+	656	692	
219	Belgium	1987-1990	MONICA, Charleroi	Community	urban	25-64	25-64	325	301	
220	Belgium	1988-1990	MONICA, Ghent	Community	urban	25-64	25-64	456	449	
221	Belgium	1990-1992	MONICA, Ghent	Community	urban	25-64	25-64	507	475	
222	Belgium	1990-1993	MONICA, Charleroi	Community	urban	25-64	25-64	337	332	
223	Belgium	1991-1994	Flemish Study on Environment, Genes and Health Outcomes	Community	rural	26+	26+	393	416	
224	Belgium	1992-1995	Flemish Study on Environment, Genes and Health Outcomes	Community	rural	27+	27+	298	312	
225	Belgium	1994-1996	BIRNH Elderly: Belgian Interuniversity Research on Nutrition and Health in the Elderly	National	both	65-89	65-89	1147	959	
226	Belgium	1996-1998	Flemish Study on Environment, Genes and Health Outcomes	Community	rural	10+	10+	404	403	
227	Belgium	1998	Flemish Study on Environment, Genes and Health Outcomes	Community	rural	32+	32+	320	359	
228	Belgium	1998-2000	Flemish Study on Environment, Genes and Health Outcomes	Community	rural	10+	10+	220	217	
229	Belgium	1999-2001	Flemish Study on Environment, Genes and Health Outcomes	Community	rural	10+	10+	232	254	
230	Belgium	2001	Flemish Study on Environment, Genes and Health Outcomes	Community	rural	10+	10+	242	222	
231	Belgium	2002-2003	Flemish Study on Environment, Genes and Health Outcomes	Community	rural	10+	10+	174	197	
232	Belgium	2002-2004	SPAH	Subnational	both	18-75	18-75	2594	2307	
233	Belgium	2002-2005	Flemish Study on Environment, Genes and Health Outcomes	Community	rural	10+	10+	447	462	
234	Belgium	2003	The European Male Ageing Study	Community	both	40+		433		
235	Belgium	2005-2008	Flemish Study on Environment, Genes and Health Outcomes	Community	rural	10+	10+	348	357	
236	Belgium	2006-2007	HELENA	Community	urban	12-17	12-17	156	180	
237	Belgium	2006-2008	Flemish Study on Environment, Genes and Health Outcomes	Community	rural	10+	10+	111	110	
238	Belgium	2007-2008	Childhood Obesity Surveillance Initiative 1	Subnational	both	6-9	6-9	64322	61754	
239	Belgium	2007-2010	Identification and prevention of Dietary- and lifestyle-induced health EFfects In Children and infantS (IDEFICS)	Community	urban	5-9	5-9	822	834	
240	Belgium	2008	The European Male Ageing Study	Community	both	45+		383		
241	Belgium	2009-2010	Childhood Obesity Surveillance Initiative 2	Subnational	both	6-9	6-9	67775	65365	
242	Belgium	2009-2013	Flemish Study on Environment, Genes and Health Outcomes	Community	rural	20+	20+	330	335	
243	Belgium	2010	EuropeaN Energy balance Research to prevent excessive weight Gain among Youth - The ENERGY-project	Subnational	urban	10-12	10-12	460	497	
244	Belgium	2010-2015	Flemish Study on Environment, Genes and Health Outcomes	Community	rural	15+	15+	388	410	
245	Belgium	2012-2013	Childhood Obesity Surveillance Initiative 3	Subnational	both	6-9	6-9	70441	67874	
246	Belgium	2014-2015	Food Consumption Survey	National	both	5-64	5-64	1477	1485	
247	Belgium	2018-2019	Belgian Health Examination Survey	National	both	18+	18+	558	614	5
248	Belgium	2021-2022	OUTSIDE-IN	Community	both	11-13	11-13	229	198	
249	Belgium	2021-2025	ENVIRONmental influence ON AGEing in early life (ENVIRONAGE)	Subnational	both	9-11	9-11	165	177	
250	Belgium	2022	OUTSIDE-IN	Community	both	12-13	12-13	210	172	

	Country	Study years	Survey/Study name/Citation	Level of representative-ness	Rural, urban, or both	Age range as in NCD-RisC database		Sample size		Note
						Male	Female	Male	Female	
251	Belgium	2022	OUTSIDE-IN	Community	both	11-13	11-13	218	188	
252	Belize	2005-2006	CAMDI	National	both	20+	20+	599	1018	
253	Belize	2011	Global School-based Student Health Survey	National	both	13	13	163	188	
254	Belize	2017	Survey of Risk Factors for Chronic Kidney Disease (SRFCKD)	National	both	20-55	20-55	3019	4481	
255	Benin	1996	DHS	National	both		20-49		2137	
256	Benin	2001	DHS	National	both		15-49		5449	
257	Benin	2006	DHS	National	both		15-49		14891	
258	Benin	2007	STEPS	Community	urban	25-64	25-64	955	1508	
259	Benin	2008	Analyse Globale de la Vulnérabilité, de la Sécurité Alimentaire et de la Nutrition (AGVSAN)	National	both		15-49		4064	
260	Benin	2008	STEPS	National	both	25-64	25-64	3430	3365	
261	Benin	2011-2012	DHS	National	both		15-49		14589	
262	Benin	2015	STEPS	National	both	18-69	18-69	2304	2543	
263	Benin	2017-2018	DHS	National	both		15-49		7180	
264	Bhutan	2007	STEPS	Community	urban	25-74	25-74	1125	1322	
265	Bhutan	2014	STEPS	National	both	18-69	18-69	1069	1674	
266	Bhutan	2019	STEPS	National	both	15-69	15-69	2159	3340	
267	Bolivia	1994	DHS	National	both		20-49		2128	
268	Bolivia	1998	DHS	National	both		20-49		3939	
269	Bolivia	2003	DHS	National	both		15-49		16349	
270	Bolivia	2005-2007	Cardiovascular and metabolic syndrome risk assessment of Bolivian school children and adolescents - Relationships to obesity, diabetes, income, food intake and physical activity	National	both	12-18	12-18	1499	1841	
271	Bolivia	2008	DHS	National	both		15-49		15543	
272	Bolivia	2016	Encuesta de Demografia y Salud	National	both		14-49		11551	
273	Bolivia	2019	STEPS	National	both	18-69	18-69	1733	2371	
274	Bolivia	2023	Encuesta de Demografía y Salud	National	both	6-59	6-59	19936	19887	
275	Bosnia and Herzegovina	2002	Non-communicable disease risk factor survey, Federation of B&H	Subnational	both	25-64	25-64	1118	1613	
276	Bosnia and Herzegovina	2012	Non-communicable disease risk factor survey, Federation of B&H	Subnational	rural	18+	18+	1190	1274	
277	Bosnia and Herzegovina	2012	Non-communicable disease risk factor survey, Federation of B&H	Subnational	urban	18+	18+	591	697	
278	Bosnia and Herzegovina	2018-2019	Determining anthropometric measurements of students aged seven to eight from Sarajevo Canton, Bosnia and Herzegovina	Community	both	6-8	6-8	42	43	
279	Bosnia and Herzegovina	2024	Survey of elementary school students in Zavidovici	Community	urban	12-16	12-16	93	117	
280	Botswana	2007	STEPS	National	both	25-64	25-64	1243	2577	
281	Botswana	2007-2008	Family Health Survey	National	both	12-49	12-49	5970	6537	
282	Botswana	2014	STEPS	National	both	15-69	15-69	1298	2602	
283	Botswana	2017	Botswana Demographic Survey	National	both	5+	5+	10896	12181	
284	Botswana	2024	STEPS	National	both	18-69	18-69	1210	2016	
285	Brazil	1989	Pesquisa Nacional sobre Saude e Nutricao	National	both	5+	5+	26642	27504	
286	Brazil	1990-1991	Fornes et al., Rev Saude Publica 36:12-8, 2002	Community	urban	20+	20+	432	613	
287	Brazil	1991-1993	EPIDOSO	Community	urban	65+	65+	269	473	
288	Brazil	1992-1998	Moraes et al., Int J Cardiol 90:205-11, 2003	Community	urban	18+	18+	438	543	
289	Brazil	1995	The 1982 Pelotas (Brazil) Birth Cohort: 13 years follow-up	Community	urban	13	13	352	363	
290	Brazil	1995	Health and Nutrition Survey of Rio de Janeiro	Community	urban	60+	60+	248	385	
291	Brazil	1995-1996	Cohort study from Porto Alegre	Community	urban	18+	18+	489	596	
292	Brazil	1996	DHS	National	both		20-49		2884	
293	Brazil	1996-1997	Pesquisa sobre Padrões de Vida (PPV)	Subnational	both	5+	5+	7451	8466	
294	Brazil	1996-1997	The Bambui Cohort Study of Ageing	Community	urban	18+	18+	931	1335	
295	Brazil	1997	The 1982 Pelotas (Brazil) Birth Cohort: 15 years follow-up	Community	urban	15	15	559	513	
296	Brazil	1998	Belo Horizonte Heart Study	Community	urban	6-18	6-18	658	738	
297	Brazil	1999-2000	Projeto Esporte Brasil	National	urban	6-11	6-11	107	102	
298	Brazil	1999-2000	The Survey on Health, Well-Being, and Aging in Latin America and the Caribbean (SABE)	Community	urban	60+	60+	732	1064	4
299	Brazil	1999-2000	Prevalence of Risk Factors for Coronary Artery Disease in the State of Rio Grande do Sul	Subnational	urban	20+	20+	494	547	
300	Brazil	1999-2000	Pelotas cross-sectional survey	Community	urban	20-69	20-69	839	1096	
301	Brazil	2000	The 1982 Pelotas (Brazil) Birth Cohort: 18 years follow-up	Community	urban	18		2228		
302	Brazil	2001	Projeto Esporte Brasil	National	urban	6-14	6-17	249	225	
303	Brazil	2001	Freitas et al., Arq Bras Cardiol 88:191-99, 2007	Community	urban	15+	15+	310	331	
304	Brazil	2001	The 1982 Pelotas (Brazil) Birth Cohort: 19 years follow-up	Community	urban		19		919	
305	Brazil	2001-2003	Bustos et al., Nutr Metab Cardiovasc Dis 17:581-89, 2007	Community	both	22-28	22-28	992	1064	
306	Brazil	2002	Study of the prevalence of obesity in children and adolescents (Estudo da prevalência da obesidade em crianças e adolescentes (EPOCA))	Community	urban	7-10	7-10	1504	1430	
307	Brazil	2002-2003	Pesquisa de Orcamentos Familiares	National	both	5+	5+	81152	80163	
308	Brazil	2002-2004	1978-1979 Ribeira Preto Birth Cohort	Community	urban	22-25	22-25	1012	1082	
309	Brazil	2003	Projeto Esporte Brasil	National	both	6-14	6-17	2016	2073	
310	Brazil	2003	Nutrition, Physical Activity, and Health Survey (PNAFS)	Community	urban	20+	20+	1152	1937	
311	Brazil	2003	Women health in Southern Brazil	Community	urban		20-60		986	

	Country	Study years	Survey/Study name/Citation	Level of representative-ness	Rural, urban, or both	Age range as in NCD-RisC database		Sample size		Note
						Male	Female	Male	Female	
312	Brazil	2003-2005	Sao Paulo Health and Ageing Study	Community	urban	65+	65+	783	1198	
313	Brazil	2004	Projeto Esporte Brasil	National	both	6-14	6-17	14447	12254	
314	Brazil	2004	Caju & Virgen das Gracas	Community	rural	18+	18+	291	286	
315	Brazil	2004-2005	The 1993 Pelotas (Brazil) Birth Cohort: 11 years follow-up	Community	urban	10-12	10-12	2184	2257	
316	Brazil	2004-2005	1994 Ribeira Preto Birth Cohort	Community	urban	10-11	10-11	400	388	
317	Brazil	2004-2005	The 1982 Pelotas (Brazil) Birth Cohort: 23 years follow-up	Community	urban	23	23	2173	1935	
318	Brazil	2004-2006	Hearts of Brazil	National	urban	18+	18+	550	626	
319	Brazil	2005	Prevalência e Fatores de Risco Cardiovascular em Crianças	Community	urban	7-12	7-12	776	719	
320	Brazil	2005	Projeto Esporte Brasil	National	both	6-14	6-17	3615	3287	
321	Brazil	2005	Syndrome of Obesity and Risk Factors for Cardiovascular Disease Study	Community	urban	18-90	18-90	739	1093	
322	Brazil	2005-2006	Sao Luis Birth Cohort	Community	urban	7-8	7-8	347	325	
323	Brazil	2006	ATITUDE	Subnational	both	14	14-20	51	2665	
324	Brazil	2006	The Ouro Preto Study	Community	urban	7-14	7-14	364	399	
325	Brazil	2006	Pesquisa Nacional de Demografia e Saude 2006	National	both		15-49		14783	
326	Brazil	2006	Projeto Esporte Brasil	National	both	6-14	6-17	7390	6211	
327	Brazil	2006	Krause et al., J Aging Phys Act 17:387-97, 2009	Community	urban	60+	60+	93	1069	
328	Brazil	2006-2007	Syndrome of Obesity and Risk Factors for Cardiovascular Disease Study among Teenagers	Community	urban	11-18	11-18	230	236	
329	Brazil	2007	Study of the prevalence of obesity in children and adolescents (Estudo da prevalência da obesidade em crianças e adolescentes (EPOCA))	Community	urban	7-14	7-14	1354	1463	
330	Brazil	2007	Prevalence of overweight and obesity in children from Medianeira, Paraná, Brazil	Community	urban	5-12	5-12	529	515	
331	Brazil	2007	Projeto Esporte Brasil	National	both	6-14	6-17	4444	4215	
332	Brazil	2007	Cardiovascular Disease Risk Factors in Caxias do Sul-RS, Brazil Adolescents	Community	urban	11-17	11-17	774	886	
333	Brazil	2007-2008	Christofaro et al., Scand J Med Sci Sports 23(3):317-22, 2013	Community	urban	10-16	10-16	493	528	
334	Brazil	2007-2008	Nutritional status of children in daycare center	Community	urban	5-7	5-7	83	81	
335	Brazil	2007-2009	Schoolchildren's Health	Community	both	6-14	6-17	690	742	
336	Brazil	2008	The 1993 Pelotas (Brazil) Birth Cohort: 15 years follow-up	Community	urban	14-15	14-15	2001	2095	
337	Brazil	2008	Projeto Esporte Brasil	National	both	6-14	6-17	2979	2386	
338	Brazil	2008	The Bambui Cohort Study of Ageing	Community	urban	71+	71+	248	456	
339	Brazil	2008	Caju & Virgen das Gracas	Community	rural	18+	18+	273	287	
340	Brazil	2008-2009	Pesquisa de Orcamentos Familiares	National	both	5+	5+	85725	88156	
341	Brazil	2008-2010	Machado-Rodrigues et al., Ann Hum Biol 41(3): 271-6, 2013	Community	urban	10-17	10-17	376	507	
342	Brazil	2009	Projeto Esporte Brasil	National	both	6-14	6-17	652	767	
343	Brazil	2009	Pesquisa Nacional de Saude do Escolar (PeNSE)	National	both	13-14	13-16	17945	30060	
344	Brazil	2009-2010	EpiFloripa Cohort Study of Ageing - Wave 1	Community	urban	60+	60+	592	1047	
345	Brazil	2009-2010	EpiFloripa Adults Cohort Study	Community	urban	20-59	20-59	755	940	
346	Brazil	2010	Longitudinal Study of Health and Wellbeing in Preschool Age (Project ELOS-Pré)	Community	urban	5	5	255	247	
347	Brazil	2010	Projeto Esporte Brasil	National	both	6-14	6-17	871	1047	
348	Brazil	2010	San Pedro	Community	rural	18+	18+	153	214	
349	Brazil	2010-2011	The 2004 Pelotas (Brazil) Birth Cohort: 6 years follow-up	Community	urban	6-7	6-7	1721	1631	
350	Brazil	2010-2015	Baependi Heart Study	Community	rural	18+	18+	1002	1357	
351	Brazil	2011	ATITUDE	Subnational	both	14	14-19	79	3658	
352	Brazil	2011	Projeto Esporte Brasil	National	both	6-14	6-17	904	929	
353	Brazil	2011	Pregnancy in adolescence in municipalities of small size in the Northeast of Brazil	Community	both	13-19	13-19	512	563	
354	Brazil	2011-2012	The 1993 Pelotas (Brazil) Birth Cohort: 18 years follow-up	Community	urban	17-19	17-19	1970	2004	
355	Brazil	2011-2012	Schoolchildren's Health	Community	both	6-14	6-17	708	1052	
356	Brazil	2011-2013	International Study of Childhood Obesity, Lifestyle and the Environment (ISCOLE)	Community	urban	9-11	9-11	277	287	
357	Brazil	2011-2014	Profile of Risk Factors for Coronary Arterial Disease in Rio Grade do Sul - Revaluation After 10 Years	Subnational	urban	20+	20+	364	466	
358	Brazil	2012	Anthropometric indices in Brazilian children: Colombo, Parana, Brazil	Community	both	6-11	6-11	1022	1013	
359	Brazil	2012	Longitudinal Study of Health and Wellbeing in Preschool Age (Project ELOS-Pré)	Community	urban	5-7	5-7	388	348	
360	Brazil	2012	Projeto Esporte Brasil	National	both	6-14	6-17	2610	1976	
361	Brazil	2012	EpiFloripa Adults Cohort Study	Community	urban	22-62	22-62	486	655	
362	Brazil	2012-2013	Study of the prevalence of obesity in children and adolescents (Estudo da prevalência da obesidade em crianças e adolescentes (EPOCA))	Community	urban	7-14	7-14	1162	1322	
363	Brazil	2012-2013	Evaluation of the realization of the human right to adequate food among public and private elementary school students in Maceió	Subnational	urban	9-11	9-11	639	701	
364	Brazil	2012-2013	Prevalence of Leptin Polymorphism Gln223Arg	Community	urban	18+	18+	282	523	
365	Brazil	2012-2013	The 1982 Pelotas (Brazil) Birth Cohort: 30 years follow-up	Community	urban	30	30	1753	1798	
366	Brazil	2013	Projeto Esporte Brasil	National	urban	6-14	6-17	1026	960	
367	Brazil	2013	Pesquisas Nacional de Saude	National	both	18+	18+	24918	32351	
368	Brazil	2013-2014	Estudo de Riscos Cardiovasculares em Adolescentes (ERICA)	National	both	12-17	12-17	32728	40680	
369	Brazil	2013-2014	EpiFloripa Cohort Study of Ageing - Wave 2	Community	urban	63+	63+	404	744	
370	Brazil	2014	Longitudinal Study of Health and Wellbeing in Preschool Age (Project ELOS-Pré)	Community	urban	7-9	7-9	223	200	
371	Brazil	2014	Projeto Esporte Brasil	National	both	6-14	6-17	219	212	
372	Brazil	2014	Brazilian Guide to the Physical Fitnees related to Health Assessment and Lifestyle Habits	Community	urban	14-19	14-19	473	535	

	Country	Study years	Survey/Study name/Citation	Level of representative-ness	Rural, urban, or both	Age range as in NCD-RisC database		Sample size		Note
						Male	Female	Male	Female	
373	Brazil	2014-2015	Latin American Study of Nutrition and Health (ELANS)	National	urban	15-65	15-65	942	1058	
374	Brazil	2014-2015	Schoolchildren's Health	Community	both	6-14	6-17	923	1398	
375	Brazil	2014-2015	II Diagnóstico de Saúde da População Materno-Infantil do Estado de Alagoas	Subnational	both		19-49		3135	
376	Brazil	2014-2015	EpiFloripa Adults Cohort Study	Community	urban	25-65	25-65	353	476	
377	Brazil	2015	The 2004 Pelotas (Brazil) Birth Cohort: 11 years follow-up	Community	urban	10-11	10-11	1736	1632	
378	Brazil	2015	Projeto Esporte Brasil	National	urban	6-14	6-17	383	496	
379	Brazil	2015	Pesquisa Nacional de Saude do Escolar (PeNSE)	National	both	13-14	13-17	2219	5054	
380	Brazil	2015-2016	The Ouro Preto Study	Community	rural	18+	18+	184	329	
381	Brazil	2015-2016	Brazilian Longitudinal Study of the Elderly Health and Wellness	National	both	50+	50+	3937	5064	
382	Brazil	2015-2016	The 1993 Pelotas (Brazil) Birth Cohort: 22 years follow-up	Community	urban	21-23	21-23	1687	1872	
383	Brazil	2016	ATITUDE	Subnational	both	14	14-19	64	3198	
384	Brazil	2016	Projeto Esporte Brasil	National	urban	6-14	6-17	169	110	
385	Brazil	2016-2017	Schoolchildren's Health	Community	both	6-14	6-17	904	1424	
386	Brazil	2016-2017	Study in Presidente Prudente	Community	urban	18+	18+	304	481	
387	Brazil	2017	Projeto Esporte Brasil	National	both	6-14	6-17	2226	1350	
388	Brazil	2017	Intervention in physical education classes to reduce sedentary behavior and improve cognitive function: SACODE	Community	both	14-19	14-19	501	631	
389	Brazil	2017	Effectiveness of interventions for health promotion in frail older adults with chronic non-communicable diseases in primary healthcare in Recife: a randomized community trial	Community	urban	60+	60+	155	528	
390	Brazil	2017	HealthRise Evaluation	Subnational	both	30+	30+	599	1169	
391	Brazil	2017-2018	EpiFloripa Cohort Study of Ageing - Wave 3	Community	urban	60+	60+	361	635	
392	Brazil	2018	Healthy Living study in Lagoa do Carro	Community	both	5-15	5-15	704	657	
393	Brazil	2018	Projeto Esporte Brasil	National	both	6-14	6-17	959	1151	
394	Brazil	2018-2019	Study of the prevalence of obesity in children and adolescents (Estudo da prevalência da obesidade em crianças e adolescentes (EPOCA))	Community	urban	7-14	7-14	680	871	
395	Brazil	2018-2019	BP-SAMPA Project	Community	urban	10-17	10-17	1328	1853	
396	Brazil	2018-2019	Epidemiology in the health (Santo Anastácio Edition)	Community	urban	18+	18+	105	145	
397	Brazil	2019	Pesquisas Nacional de Saude	National	both	15+	15+	3304	3243	
398	Brazil	2019	Projeto Esporte Brasil	National	both	6-14	6-17	1126	1355	
399	Brazil	2019-2020	Pelotas 2004 Birth Cohort - 15 year follow up	Community	urban	15-16	15-16	975	928	
400	Brazil	2019-2021	Brazilian Longitudinal Study of the Elderly Health and Wellness	National	both	50+	50+	3319	5004	
401	Brazil	2020	Projeto Esporte Brasil	National	urban	6-14	6-17	277	154	
402	Brazil	2020-2021	The Ouro Preto Study	Community	both	6-17	6-17	477	456	
403	Brazil	2020-2022	ATITUDE	Subnational	both	14	14-19	36	2271	
404	Brazil	2021	Projeto Esporte Brasil	National	urban	6-14	6-17	373	372	
405	Brazil	2022	Pelotas 2004 Birth Cohort - 18 year follow up	Community	urban	17-18	17-18	1560	1508	
406	Brazil	2022	Projeto Esporte Brasil	National	both	6-14	6-17	575	795	
407	Brazil	2023	Projeto Esporte Brasil	National	both	6-14	6-17	710	772	
408	Brunei Darussalam	2010-2011	National Health And Nutritional Status Survey (NHANSS)	National	both	5-75	5-75	1027	1157	
409	Brunei Darussalam	2014	Global School-based Student Health Survey	National	both	12-17	12-17	1145	1326	
410	Brunei Darussalam	2015-2016	National Non-Communicable Diseases Survey (NNCDS)	National	both	18-69	18-69	814	1075	
411	Brunei Darussalam	2019	Global School-based Student Health Survey	National	both	12-17	12-17	1061	1152	
412	Brunei Darussalam	2022-2023	National Non-Communicable Diseases Survey (NNCDS)	National	both	18-69	18-69	1478	1520	
413	Bulgaria	1984-1988	Postnatal growth of SGA and normal born high school graduates	Community	urban	5	5	753	952	
414	Bulgaria	1985-1989	Postnatal growth of SGA and normal born high school graduates	Community	urban	6	6	788	993	
415	Bulgaria	1986-1990	Postnatal growth of SGA and normal born high school graduates	Community	urban	7	7	817	1049	
416	Bulgaria	1987-1991	Postnatal growth of SGA and normal born high school graduates	Community	urban	8	8	767	989	
417	Bulgaria	1988-1992	Postnatal growth of SGA and normal born high school graduates	Community	urban	9	9	732	940	
418	Bulgaria	1989-1993	Postnatal growth of SGA and normal born high school graduates	Community	urban	10	10	719	906	
419	Bulgaria	1990-1994	Postnatal growth of SGA and normal born high school graduates	Community	urban	11	11	729	927	
420	Bulgaria	1991-1995	Postnatal growth of SGA and normal born high school graduates	Community	urban	12	12	740	972	
421	Bulgaria	1992-1996	Postnatal growth of SGA and normal born high school graduates	Community	urban	13	13	752	947	
422	Bulgaria	1993	Anthropometric characterization of growth and development in children aged 7 to 13 years from Sofia at the beginning of the 21st century	Community	urban	6-7	6-7	109	119	
423	Bulgaria	1993-1997	Postnatal growth of SGA and normal born high school graduates	Community	urban	14	14	750	893	
424	Bulgaria	1994	Anthropometric characterization of growth and development in children aged 7 to 13 years from Sofia at the beginning of the 21st century	Community	urban	7-8	7-8	108	118	
425	Bulgaria	1994-1998	Postnatal growth of SGA and normal born high school graduates	Community	urban	15	15	816	972	
426	Bulgaria	1995	Anthropometric characterization of growth and development in children aged 7 to 13 years from Sofia at the beginning of the 21st century	Community	urban	8-9	8-9	109	120	
427	Bulgaria	1995-1999	Postnatal growth of SGA and normal born high school graduates	Community	urban	16	16	896	1012	
428	Bulgaria	1995-2001	Anthropometric characterization of growth and development in children aged 7 to17 years from Sofia at the beginning of the 21st century	Community	urban	7-17	7-17	1127	1132	

	Country	Study years	Survey/Study name/Citation	Level of representative-ness	Rural, urban, or both	Age range as in NCD-RisC database		Sample size		Note
						Male	Female	Male	Female	
429	Bulgaria	1996	Anthropometric characterization of growth and development in children aged 7 to 13 years from Sofia at the beginning of the 21st century	Community	urban	9-10	9-10	109	119	
430	Bulgaria	1996-2000	Postnatal growth of SGA and normal born high school graduates	Community	urban	17	17	816	872	
431	Bulgaria	1997	Anthropometric characterization of growth and development in children aged 7 to 13 years from Sofia at the beginning of the 21st century	Community	urban	10-11	10-11	109	119	
432	Bulgaria	1997-2000	Postnatal growth of SGA and normal born high school graduates	Community	urban	18	18	485	322	
433	Bulgaria	1998	Anthropometric characterization of growth and development in children aged 7 to 13 years from Sofia at the beginning of the 21st century	Community	urban	11-12	11-12	109	118	
434	Bulgaria	1999	Anthropometric characterization of growth and development in children aged 7 to 13 years from Sofia at the beginning of the 21st century	Community	urban	12-13	12-13	109	118	
435	Bulgaria	2004	National Survey of Food Intake and Nutritional Status 2004	National	both	16+	16+	587	617	
436	Bulgaria	2004	National Nutrition Survey	National	both	15+	15+	515	515	
437	Bulgaria	2004-2005	Anthropometric characterization of growth and development in children aged 3 to 6 years from Sofia at the beginning of the 21st century	Community	urban	5-6	5-6	160	160	
438	Bulgaria	2006-2007	Waist circumference percentile curves for Bulgarian children and adolescents aged 6-18 years	Community	urban	5-18	5-18	2052	1758	
439	Bulgaria	2008	Childhood Obesity Surveillance Initiative 1	National	both	7-8	7-8	1657	1661	
440	Bulgaria	2009	Survey of children in Varna kindergartens	Community	urban	5-6	5-6	188	157	
441	Bulgaria	2013	Childhood Obesity Surveillance Initiative 3	National	both	7	7	1671	1677	
442	Bulgaria	2014	Bulgarian National Monitoring of Dietary Intake	National	both	20+	20+	1421	1552	
443	Bulgaria	2015-2016	Childhood Obesity Surveillance Initiative 4	National	both	7	7	1702	1698	
444	Bulgaria	2016	Feel4Diabetes	Community	urban	6-10	6-10	1447	1522	
445	Bulgaria	2016-2017	Erasmus plus KA2, Healthyland	Community	urban	5	5	26	24	
446	Bulgaria	2017-2018	Erasmus plus KA2, Healthyland	Community	urban	5-6	5-6	49	51	
447	Bulgaria	2019	Childhood Obesity Surveillance Initiative 5	National	both	7	7	1536	1531	
448	Bulgaria	2020	National Survey on Risk Factors for Population's Health in Bulgaria 2020	National	both	5+	5+	1686	2297	
449	Bulgaria	2023	Childhood Obesity Surveillance Initiative 6	National	both	7	7	1577	1554	
450	Burkina Faso	1992-1993	DHS	National	both		20-49		3190	
451	Burkina Faso	1998-1999	DHS	National	both		20-49		3114	
452	Burkina Faso	2002	Vulnérabilité Alimentaire et Sécurité Nutritionnelle dans la Gnagna (VASN-Gnagna)	Subnational	rural	5+	5+	1471	3522	
453	Burkina Faso	2003	DHS	National	both		15-49		11001	
454	Burkina Faso	2004	Ouedraogo et al., Public Health Nutr 11:1280-87, 2008	Community	urban	35+	35+	956	1066	
455	Burkina Faso	2010	DHS	National	both		15-49		7755	
456	Burkina Faso	2013	STEPS	National	both	25-64	25-64	2223	2250	
457	Burkina Faso	2017	ARISE Network Adolescent Health Study (Nouna)	Community	both	12-19	12-19	915	680	
458	Burkina Faso	2021	DHS	National	both		15-49		8005	
459	Burkina Faso	2021	STEPS	National	both	18-69	18-69	1467	2002	
460	Burundi	2010	DHS	National	both		15-49		4188	
461	Burundi	2016-2017	DHS	National	both		15-49		7909	
462	Cabo Verde	2007	STEPS	National	both	25-64	25-64	658	1066	
463	Cabo Verde	2020	STEPS	National	both	18-69	18-69	1824	2636	
464	Cambodia	2000	DHS	National	both		15-49		6915	
465	Cambodia	2005	DHS	National	both		15-49		8130	
466	Cambodia	2008	Anthropometrics Survey	National	both		15-49		5955	
467	Cambodia	2010	DHS	National	both		15-49		8856	
468	Cambodia	2010	STEPS	National	both	25-64	25-64	1881	3344	
469	Cambodia	2014	DHS	National	both		15-49		10821	
470	Cambodia	2016	STEPS	National	both	18-69	18-69	927	1858	
471	Cambodia	2021-2022	DHS	National	both		15-49		9332	
472	Cambodia	2023	STEPS	National	both	18-69	18-69	1504	2669	
473	Cameroon	1998	DHS	National	both		20-49		1429	
474	Cameroon	1998-1999	Essential Non-communicable disease Health Intervention Project (ENHIP)	Community	rural	15+	15+	523	738	
475	Cameroon	1998-1999	Essential Non-communicable disease Health Intervention Project (ENHIP)	Community	urban	15+	15+	523	640	
476	Cameroon	2003	STEPS	Subnational	urban	15+	15+	3672	5490	
477	Cameroon	2004	DHS	National	both		15-49		4646	
478	Cameroon	2007	Cameroon Burden of Diabetes - Second Survey	Subnational	urban	18+	18+	3345	4633	
479	Cameroon	2009	National Survey of Micronutrient Status and Consumption of Fortifiable Foods	National	both		15-49		816	
480	Cameroon	2009-2012	Anthropologie nutritionnelle des migrants d'Afrique centrale à la ville et en France	Subnational	both	18-76	18-76	528	584	
481	Cameroon	2011	DHS	National	both		15-49		7343	
482	Cameroon	2013	Prevalence and risk factors of chronic kidney disease in urban adult Cameroonians according to three common estimators of the glomerular filtration rate: a cross-sectional study	Community	urban	19+	19+	265	232	
483	Cameroon	2014	Prevalence and determinants of chronic kidney disease in rural and urban Cameroonians: A cross-sectional study	Community	both	20+	20+	177	246	
484	Cameroon	2014-2015	Cardiovascular risk factors screening in urban and rural areas in the Far-North Region Cameroon	Subnational	both	20+	20+	520	369	
485	Cameroon	2018	Prevalence and determinants of chronic kidney disease in urban adults' populations of northern Cameroon	Community	urban	20+	20+	210	221	

	Country	Study years	Survey/Study name/Citation	Level of representative-ness	Rural, urban, or both	Age range as in NCD-RisC database		Sample size		Note
						Male	Female	Male	Female	
486	Cameroon	2018-2019	DHS	National	both		15-64		6255	
487	Canada	1981	Canada Fitness Survey	National	both	7-64	7-64	7432	7940	
488	Canada	1985-1986	INTERSALT, St Johns	Community	urban	20-59	20-59	100	100	
489	Canada	1985-1988	MONICA, Halifax	Community	both	25-64	25-64	438	420	
490	Canada	1986-1992	Canada Heart Health Survey	National	both	18-74	18-74	9644	9777	
491	Canada	1991-1992	Canadian Study of Health and Aging	Community	both	70+	70+	236	348	
492	Canada	1993	Chen et al., Int J Obes Relat Metab Disord 22:771-77, 1998	Community	rural	18-74	18-74	803	988	
493	Canada	1995	MONICA, Halifax	Community	both	25-64	25-64	274	287	
494	Canada	1995-1997	Canadian Multicentre Osteoporosis Study (CaMos) - Adult Baseline	Subnational	both	25+	25+	2803	6343	
495	Canada	1996	Canadian Study of Health and Aging	Community	both	70+	70+	236	348	
496	Canada	1997	PEI Nutrition Survey	Subnational	both	18-74	18-74	998	993	
497	Canada	1998-2000	Canadian Multicentre Osteoporosis Study (CaMos) - Adult Year 3 follow-up	Subnational	both	42-64	42-64	782	1670	
498	Canada	2000-2003	Canadian Multicentre Osteoporosis Study (CaMos) - Adult Year 5 follow-up	Subnational	both	30+	30+	1992	4820	
499	Canada	2004-2005	Canadian Multicentre Osteoporosis Study (CaMos) - Youth baseline	Subnational	both	16-24	16-24	471	521	
500	Canada	2005	Canadian Community Health Survey	National	both	15+	15+	1680	2026	
501	Canada	2005-2008	Canadian Multicentre Osteoporosis Study (CaMos) - Adult Year 10 follow-up	Subnational	both	35+	35+	1486	3661	
502	Canada	2006-2008	Canadian Multicentre Osteoporosis Study (CaMos) - Youth Year 2 follow-up	Subnational	both	17-27	17-27	331	383	
503	Canada	2007-2009	Canadian Health Measures Survey, Cycle 1	National	both	6-79	6-79	2703	2864	
504	Canada	2008	Canadian Community Health Survey	National	both	15+	15+	1687	1984	
505	Canada	2009-2011	Canadian Health Measures Survey, Cycle 2	National	both	5-79	5-79	2870	3086	
506	Canada	2011-2013	International Study of Childhood Obesity, Lifestyle and the Environment (ISCOLE)	Community	urban	9-11	9-11	238	327	
507	Canada	2012-2013	Canadian Health Measures Survey, Cycle 3	National	both	5-79	5-79	2670	2676	
508	Canada	2012-2014	Canadian Multicentre Osteoporosis Study (CaMos) - Adult Year 16 follow-up	Subnational	both	60+	60+	445	1307	
509	Canada	2014-2015	Canadian Health Measures Survey, Cycle 4	National	both	5-79	5-79	2697	2674	
510	Canada	2015	Canadian Community Health Survey - Nutrition	National	both	5+	5+	6316	7023	
511	Canada	2016-2017	Canadian Health Measures Survey, Cycle 5	National	both	5-79	5-79	2677	2660	
512	Canada	2018-2019	Canadian Health Measures Survey, Cycle 6	National	both	5-79	5-79	2685	2680	
513	Central African Republic	1994-1995	DHS	National	both		20-49		1760	
514	Central African Republic	2010	STEPS	Subnational	both	25-64	25-64	1846	1967	
515	Central African Republic	2011-2012	Epidemiology of dementia in Central Africa: Bangui	Community	urban	65+	65+	157	300	6
516	Central African Republic	2011-2012	Epidemiology of dementia in Central Africa: Nola	Community	rural	65+	65+	184	264	6
517	Central African Republic	2017	STEPS	Subnational	both	25-64	25-64	1076	1834	
518	Chad	1996-1997	DHS	National	both		20-49		3262	
519	Chad	2004	DHS	National	both		20-49		2618	
520	Chad	2008	STEPS	Community	urban	25-64	25-64	995	845	
521	Chad	2014-2015	DHS	National	both		15-49		9733	
522	Chile	1988	Chilean Health Study	Subnational	urban	15+	15+	471	741	
523	Chile	1989	INCLEN	Community	urban	35-65		199		
524	Chile	1992-1993	Miquel et al., Gastroenterology 115(4):937-46, 1998	Community	urban	18+	18+	657	1031	
525	Chile	1999-2000	The Survey on Health, Well-Being, and Aging in Latin America and the Caribbean (SABE)	Community	urban	60+	60+	410	806	4
526	Chile	2000	Nervi et al., J Hepatol 45(2):299-305, 2006	Community	urban	18+	18+	335	624	
527	Chile	2001-2003	Bustos et al., Nutr Metab Cardiovasc Dis 17:581-89, 2007	Community	both	22-28	22-28	436	562	
528	Chile	2003	Encuesta Nacional de Salud	National	both	17+	17+	1557	1867	7
529	Chile	2004-2005	CArdiovascular Risk factors Multiple Evaluation in Latin America (CARMELA)	Community	urban	25-64	25-64	783	865	
530	Chile	2005	Palomo et al., Rev Med Chil 135:904-12, 2007	Community	urban	18-74	18-74	339	668	
531	Chile	2009-2010	Encuesta Nacional de Salud	National	both	15+	15+	1935	2869	7
532	Chile	2009-2011	Nutritional status, metabolic syndrome and insulin resistance in children from Santiago (Chile)	Community	urban	10-15	10-15	1589	1736	
533	Chile	2010-2011	Encuesta Nacional de Consumo Alimentario	National	both	5+	5+	1840	2902	
534	Chile	2011-2012	CESCAS Study	Community	urban	35-74	35-74	917	1000	
535	Chile	2013	Global School-based Student Health Survey	National	both	13-17	13-17	799	793	
536	Chile	2014-2015	Latin American Study of Nutrition and Health (ELANS)	National	urban	15-65	15-65	425	454	
537	Chile	2016-2017	Encuesta Nacional de Salud	National	both	15+	15+	1977	3420	7
538	Chile	2017	Encuesta Longitudinal de Primera Infancia	National	both	10-12	10-12	2927	2817	
539	China	1979-1982	East Beijing Study 1	Community	urban	20-84	20-84	361	380	
540	China	1982	China National Nutrition Survey	National	both	7+	7+	14418	13683	
541	China	1983	Sino-MONICA Shanghai	Community	rural	30-64	30-64	624	630	
542	China	1984-1985	Sino-MONICA Beijing	Community	both	25-64	25-64	813	857	
543	China	1985	Chinese National Surveys on Students Constitution and Health	National	both	7-18	7-18	205041	204796	
544	China	1985-1986	Shatin New Town Study	Community	urban	70+	70+	276	669	
545	China	1986	INTERSALT, Beijing	Community	urban	20-59	20-59	100	100	
546	China	1986	INTERSALT, Nanning	Community	urban	20-59	20-59	100	100	
547	China	1986	INTERSALT, Tianjin	Community	urban	20-59	20-59	100	100	
548	China	1986-1989	Ewang et al., Zhonghua Liu Xing Bing Xue Za Zhi 26:394-9, 2005	Community	both	45-64		18243		

	Country	Study years	Survey/Study name/Citation	Level of representative-ness	Rural, urban, or both	Age range as in NCD-RisC database		Sample size		Note
						Male	Female	Male	Female	
549	China	1986-1989	Sino-MONICA Shanghai	Community	rural	25-64	25-64	675	753	
550	China	1987	INCLEN	Community	urban	35-65		989		
551	China	1988	Sino-MONICA Hebei	Community	both	25-64		800		
552	China	1988	Sino-MONICA Heilongjiang	Community	urban	25-64	25-64	800	800	
553	China	1988	Sino-MONICA Henan	Community	urban	25-64	25-64	345	427	
554	China	1988	Sino-MONICA Neimenggu	Community	urban	25-64	25-64	396	400	
555	China	1988	Sino-MONICA Sichuan	Community	both	25-64	25-64	312	334	
556	China	1988	Sino-MONICA Shandong	Community	urban	25-64	25-64	211	225	
557	China	1988-1989	Sino-MONICA Beijing	Community	both	25-64	25-64	701	862	
558	China	1988-1989	Sino-MONICA Jilin	Community	urban	25-64	25-64	380	400	
559	China	1988-1989	Sino-MONICA Jiangxi	Community	urban	25-64	25-64	379	386	
560	China	1988-1989	Sino-MONICA Liaoning	Community	both	25-64	25-64	728	734	
561	China	1988-1990	East Beijing Study 2	Community	urban	20-84	20-84	135	148	
562	China	1989	China Health and Nutrition Study	National	both	5-45	5-45	2556	2715	8
563	China	1989	The Tianjin Project	Community	urban	15-64	15-64	3894	3971	
564	China	1989	Sino-MONICA Fujian	Community	urban	25-64	25-64	179	191	
565	China	1989	Sino-MONICA Jiangsu	Community	rural	25-64	25-64	398	399	
566	China	1990-1991	China Prospective Study	National	both	40-79		230676		
567	China	1991	China Health and Nutrition Study	National	both	5+	5+	5586	5920	8
568	China	1991	The Hong Kong study on health, health risk and quality of life in the Chinese elderly cohort	Community	both	70+	70+	943	944	
569	China	1991	Hua et al., Zhonghua Nei Ke Za Zhi 36:18-20, 1997	Community	rural	60+	60+	288	335	
570	China	1991	China National Hypertension Survey Epidemiology Follow-up Study	National	both	40+	40+	75696	79040	
571	China	1991	Sino-MONICA Shanghai	Community	rural	30-64	30-64	564	624	
572	China	1991-1992	Fangshan Cohort Study	Community	urban	34-86	34-86	871	1736	
573	China	1992	China National Nutrition Survey	National	both	5+	5+	33714	36271	
574	China	1992	Huashan Study	Community	urban	35-75	35-75	892	965	
575	China	1992	Sino-MONICA Sichuan	Community	both	25-64	25-64	608	526	
576	China	1992-1993	Anzhen 02 Cohort Study	Community	urban	34-65	34-65	2032	2120	
577	China	1993	China Health and Nutrition Study	National	both	5+	5+	5371	5563	8
578	China	1993	Sino-MONICA Anhui	Community	urban	25-64	25-64	193	195	
579	China	1993	Sino-MONICA Beijing	Community	both	25-64	25-64	613	816	
580	China	1993	Sino-MONICA Jiangsu	Community	urban	25-64	25-64	462	365	
581	China	1993	Sino-MONICA Liaoning	Community	both	25-64	25-64	493	500	
582	China	1995	Chinese National Surveys on Students Constitution and Health	National	both	7-18	7-18	103009	101772	
583	China	1995-1996	Hong Kong Cardiovascular Risk Factor Prevalence Study 1995-1996	Community	urban	25-74	25-74	1412	1478	
584	China	1996	Wang et al., Zhonghua Liu Xing Bing Xue Za Zhi 24:272-75, 2003	Community	both	25-64	25-64	733	719	
585	China	1996	The Tianjin Project	Community	urban	15-64	15-64	722	717	
586	China	1996-2000	Shanghai Women's Health Study	Community	urban		40-70		74915	
587	China	1996-2003	Wu et al., Osteoporos Int 15:751-59, 2004	Community	urban		18+		3418	
588	China	1997	China Health and Nutrition Study	National	both	5+	5+	5550	5648	8
589	China	1997	INTERMAP, Beijing	Community	rural	40-59	40-59	133	139	
590	China	1997	INTERMAP, Guangxi	Community	rural	40-59	40-59	140	138	
591	China	1997	INTERMAP, Shanxi	Community	rural	40-59	40-59	143	146	
592	China	1998	Shanghai Diabetes Study	Community	urban	25+	25+	1264	1768	
593	China	1998-2000	Jia et al., Obes Rev 3:157-65, 2002	Community	urban	20+	20+	1106	1670	
594	China	1999	Chen et al., Zhonghua Yi Xue Za Zhi 85(40):2830-4, 2005	Subnational	both	35-85	35-85	13549	10315	
595	China	1999	Wang et al., Zhonghua Liu Xing Bing Xue Za Zhi 24:272-75, 2003	Community	both	25-64	25-64	815	683	
596	China	1999-2000	Xu et al., Public Health Nutr 8:47-51, 2005	Community	both	35+	35+	18194	18902	
597	China	2000	China Health and Nutrition Study	National	both	5+	5+	5829	6099	8
598	China	2000	Chinese National Surveys on Students Constitution and Health	National	both	7-18	7-18	107997	108096	
599	China	2000-2001	The International Collaborative Study of Cardiovascular Disease in Asia	National	both	35-74	35-74	7512	8006	
600	China	2001	Shanghai Diabetes Study	Community	urban	25+	25+	1264	1768	
601	China	2002	China National Nutrition and Health Survey	National	both	5+	5+	84194	92687	
602	China	2002	Ma et al., Zhonghua Liu Xing Bing Xue Za Zhi 25:1035-8, 2004	Subnational	both	18+	18+	7352	7352	
603	China	2002-2003	Fan et al., J Gastroenterol Hepatol 20:1825-32, 2005	Community	urban	15+	15-74	5502	7767	
604	China	2002-2006	Shanghai Men's Health Study	Community	urban	40-74		61445		
605	China	2004	Beijing Child and Adolescent Metabolic Syndrome study	Community	both	5-18	5-18	10562	10412	
606	China	2004	China Health and Nutrition Study	National	both	5+	5+	5229	5516	8
607	China	2004	China Chronic Disease and Risk Factors Surveillance (CCDRFS)	National	rural	18-69	18-69	9561	10937	
608	China	2004	China Chronic Disease and Risk Factors Surveillance (CCDRFS)	National	urban	18-69	18-69	4978	7317	
609	China	2004	Tian et al., Prev Med 48:59-63, 2009	Community	rural	15+	15+	1022669	1163313	
610	China	2004-2005	Xinjiang Children and Adolescent Survey	Community	urban	7-18	7-18	2030	2231	
611	China	2004-2006	Pang et al., Intern Med 47:893-97, 2008	Community	rural	35+	35+	22962	22960	

	Country	Study years	Survey/Study name/Citation	Level of representative-ness	Rural, urban, or both	Age range as in NCD-RisC database		Sample size		Note
						Male	Female	Male	Female	
612	China	2004-2006	Shanghai Women's Health Study	Community	urban		45-80		64545	
613	China	2004-2008	China Kadoorie Biobank baseline survey	Subnational	rural	35-74	35-74	115792	162848	
614	China	2004-2008	China Kadoorie Biobank baseline survey	Subnational	urban	35-74	35-74	89219	132860	
615	China	2004-2008	Shanghai Men's Health Study	Community	urban	41-80		54800		
616	China	2005	Chinese National Surveys on Students Constitution and Health	National	both	7-18	7-18	117598	116704	
617	China	2005	Ye et al., J Am Coll Cardiol 49:1798-805, 2007	Community	urban	50-70	50-70	743	906	
618	China	2005-2006	Hong Kong Growth Survey	Community	urban	7-19	7-19	7472	7370	
619	China	2005-2006	Zhou et al., World J Gastroenterol 13:6419-24, 2007	Community	urban	18-79	18-79	1101	2063	
620	China	2006	China Health and Nutrition Study	National	both	5+	5+	4950	5397	8
621	China	2006	Beijing Eye Study	Community	both	45+	45+	1394	1820	
622	China	2006-2007	Handan Eye Study	Community	rural	30+	30+	2995	3456	
623	China	2006-2012	Qingdao Diabetes Cohort Study	Subnational	both	35-74	35-74	4047	6346	
624	China	2007	China Chronic Disease and Risk Factors Surveillance (CCDRFS)	National	rural	18-69	18-69	14212	15006	
625	China	2007	China Chronic Disease and Risk Factors Surveillance (CCDRFS)	National	urban	18-69	18-69	8461	10160	
626	China	2007-2008	China National Diabetes & Metabolic Disorders Study	National	both	20+	20+	18419	27820	
627	China	2007-2010	WHO Study on global AGEing and adult health (SAGE)	National	both	50+	50+	5759	6616	
628	China	2007-2011	Shanghai Women's Health Study	Community	urban		47-83		52116	
629	China	2008	China Health and Retirement Longitudinal Study (CHARLS), pilot survey	Subnational	both	45+	45+	919	920	
630	China	2008-2009	Chinese Longitudinal Healthy Longevity Survey	National	both	65+	65+	6827	8976	9
631	China	2008-2010	Fangshan Family-based Ischemic Stroke Study in China (FISSIC) program	Community	rural	40+	40+	19478	36449	
632	China	2008-2011	Shanghai Men's Health Study	Community	urban	43-84		51948		
633	China	2009	China Health and Nutrition Study	National	both	5+	5+	5176	5489	8
634	China	2009	The nutrition-based comprehensive intervention study on childhood obesity in China	Subnational	urban	6-11	6-11	4495	4269	
635	China	2009	The 33 Chinese Communities Health Study (33CCHS)	Subnational	urban	18-74	18-74	7778	7144	
636	China	2009-2010	China National Survey of Chronic Kidney Disease	National	both	18+	18+	20003	26854	
637	China	2009-2011	The FAMILY Cohort	Community	urban	15+	15+	8318	9557	
638	China	2010	Chinese National Surveys on Students Constitution and Health	National	both	7-18	7-18	107611	107611	
639	China	2010	China Chronic Disease and Risk Factors Surveillance (CCDRFS)	National	rural	18+	18+	27827	31844	
640	China	2010	China Chronic Disease and Risk Factors Surveillance (CCDRFS)	National	urban	18+	18+	17239	21608	
641	China	2010-2013	China Nutrition and Health Surveillance	National	both	5+	5+	72494	87092	
642	China	2010-2014	National Free Preconception Health Examination Project	National	rural	20-64		16166534		
643	China	2011	Beijing Childhood Eye Study	Community	both	7-18	7-18	6686	6967	
644	China	2011	Beijing Children Eye Study	Community	both	5-13	5-13	291	261	
645	China	2011	China Health and Nutrition Study	National	both	5+	5+	6770	7478	10
646	China	2011	Beijing Eye Study	Community	both	50+	50+	1467	1895	
647	China	2011-2012	China Health and Retirement Longitudinal Study (CHARLS), baseline survey	National	both	45+	45+	6337	7003	
648	China	2011-2012	Chinese Longitudinal Healthy Longevity Survey	National	both	65+	65+	4035	4620	
649	China	2011-2013	International Study of Childhood Obesity, Lifestyle and the Environment (ISCOLE)	Community	urban	9-11	9-11	293	258	
650	China	2011-2014	The FAMILY Cohort	Community	urban	15+	15+	4593	5557	
651	China	2012	Beijing Children Eye Study	Community	both	5-13	5-13	283	251	
652	China	2012	Beijing Eye High School Students Study	Community	both	16-18	16-18	2088	2340	
653	China	2012	Shandong Children Study	Community	rural	5-18	5-18	1663	1385	
654	China	2012	Shandong Children Study	Community	urban	5-18	5-18	1423	1381	
655	China	2012	China Health and Retirement Longitudinal Study (CHARLS), wave 2 pilot survey	Subnational	both	45+	45+	856	934	
656	China	2012-2013	The Seven Northeastern Cities (SNEC) Study	Subnational	urban	5-17	5-17	4769	4577	
657	China	2012-2013	The Kailuan Study	Community	urban	18+	18+	80921	21385	
658	China	2012-2015	Shanghai Men's Health Study	Community	urban	47-87		40921		
659	China	2012-2015	Shanghai Women's Health Study	Community	urban		52-88		49592	
660	China	2013	Gobi Desert Children Eye Study	Community	urban	6-21	6-21	800	761	
661	China	2013	China Health and Retirement Longitudinal Study (CHARLS), wave 2 survey	National	both	45+	45+	5898	6582	
662	China	2013-2014	China Chronic Disease and Risk Factors Surveillance (CCDRFS)	National	rural	18-69	18-69	39987	51871	
663	China	2013-2014	China Chronic Disease and Risk Factors Surveillance (CCDRFS)	National	urban	18-69	18-69	26374	38695	
664	China	2013-2014	Prevalence and Risk Factors of Prehypertension and Hypertension in Southern China	Subnational	both	15-97	15-97	6188	8915	
665	China	2013-2017	Children of 1997 Birth Cohort- Biobank Clinical Follow-up	Community	both	16-20	16-20	1823	1792	
666	China	2014	Shanghai Municipal Surveys on Students Constitution and Health	Community	both	7-18	7-18	7758	7665	
667	China	2014	Chinese National Surveys on Students Constitution and Health	National	both	7-18	7-18	107216	107138	
668	China	2014	Chinese Longitudinal Healthy Longevity Survey	National	both	65+	65+	2978	3172	
669	China	2014-2015	The Kailuan Study	Community	urban	18+	18+	73161	18280	
670	China	2015	China Health and Nutrition Study	National	both	5+	5+	6538	7222	10
671	China	2015	China Health and Retirement Longitudinal Study (CHARLS), wave 3 survey	National	both	45+	45+	7032	7719	
672	China	2015	China Nutrition and Health Surveillance (Adult Chronic Disease and Nutrition Surveillance)	National	both	18+	18+	84991	97136	
673	China	2015-2016	China Chronic Disease and Risk Factors Surveillance (CCDRFS)	National	rural	18-69	18-69	43616	49395	
674	China	2015-2016	China Chronic Disease and Risk Factors Surveillance (CCDRFS)	National	urban	18-69	18-69	30987	38057	

	Country	Study years	Survey/Study name/Citation	Level of representative-ness	Rural, urban, or both	Age range as in NCD-RisC database		Sample size		Note
						Male	Female	Male	Female	
675	China	2015-2016	INTERMAP China Prospective (ICP) Study	Subnational	rural	40-79	40-79	334	413	
676	China	2015-2017	Henan Rural Cohort	Subnational	rural	18-79	18-79	15429	23653	
677	China	2016	Greater Beijing School Children Myopia Study	Subnational	rural	6-18	6-18	12873	12866	
678	China	2016	Greater Beijing School Children Myopia Study	Subnational	urban	6-18	6-18	4384	3650	
679	China	2016-2017	China Nutrition and Health Surveillance (Children and Lactating Women)	National	both	5-17	5-17	42215	42301	
680	China	2016-2018	Smart device usage, lifestyles behaviors, physical fitness, and eye problems: A prospective study in Hong Kong adolescents	Community	urban	7-15	7-15	703	741	
681	China	2016-2018	The FAMILY Cohort	Community	urban	18+	18+	839	1122	
682	China	2016-2019	Associations between economic status and risk of obesity and overweight among Chinese children and adolescents	Subnational	both	8-17	8-17	1389	1547	
683	China	2018	China Health and Nutrition Survey	National	both	5+	5+	7870	8845	10
684	China	2018	Chinese Longitudinal Healthy Longevity Survey	National	both	65+	65+	6430	7781	11
685	China	2018-2019	China Chronic Disease and Risk Factors Surveillance (CCDRFS)	National	rural	18-69	18-69	37707	46845	
686	China	2018-2019	China Chronic Disease and Risk Factors Surveillance (CCDRFS)	National	urban	18-69	18-69	29780	41081	
687	China	2019	Chinese National Surveys on Students Constitution and Health	National	both	6-18	6-18	114825	113992	
688	China	2023	Wuhu Children and Adolescents Eye Study	Community	both	5-17	5-17	42997	35306	
689	Colombia	1986	INTERSALT	Community	rural	20-59	20-59	96	95	
690	Colombia	1995	DHS	National	both		20-49		3068	
691	Colombia	1997-1998	Identification of Risk Factors of Non-Transmissible Adult Chronic Diseases in School-age Populations in the City of de Cali	Community	urban	6-17	6-17	1084	1077	
692	Colombia	2000	DHS	National	both		20-49		2929	
693	Colombia	2001	CINDI/CARMEN - Bucaramaga	Community	urban	15-74	15-74	627	1218	
694	Colombia	2002	The Santa Fe Study (Santa Fe)	Community	urban	15-69	15-69	394	684	
695	Colombia	2002	The Santa Fe Study (Tunjuelito)	Community	urban	15-29	15-29	208	312	
696	Colombia	2002	CINDI/CARMEN - Bogota	Community	urban	15-74	15-74	322	570	
697	Colombia	2004-2005	CArdiovascular Risk factors Multiple Evaluation in Latin America (CARMELA)	Community	urban	25-64	25-64	738	812	
698	Colombia	2005	DHS	National	both	5-64	5-64	43436	57778	
699	Colombia	2005	Encuesta Nacional de Situacion Nutricional	National	both	5-12	5-49	2644	6088	
700	Colombia	2007	Encuesta Nacional de Salud	National	both	18-69	18-69	5462	7686	
701	Colombia	2010	DHS	National	both	5-64	5-64	65086	76792	
702	Colombia	2010	STEPS	Subnational	both	15-64	15-64	1034	1356	
703	Colombia	2011-2013	International Study of Childhood Obesity, Lifestyle and the Environment (ISCOLE)	Community	urban	9-11	9-11	454	462	
704	Colombia	2014-2015	Latin American Study of Nutrition and Health (ELANS)	National	urban	15-65	15-65	394	404	
705	Colombia	2015	Encuesta Nacional de Situacion Nutricional	National	both	5-64	5-64	51180	58896	
706	Colombia	2015	STEPS	Subnational	both	15-64	15-64	979	1181	
707	Colombia	2016	The Survey on Health, Well-Being, and Aging in Latin America and the Caribbean (SABE)	National	both	60+	60+	9041	11467	
708	Colombia	2018	COPEN: Estudio Colombiano de Perfiles Nutricionales	Subnational	urban	5-75	5-75	916	919	
709	Colombia	2022	COPEN: Estudio Colombiano de Perfiles Nutricionales	Subnational	urban	5-80	5-80	959	1029	
710	Comoros	1996	DHS	National	both		20-49		744	
711	Comoros	2011	STEPS	National	both	25-64	25-64	1541	3505	
712	Comoros	2012	DHS	National	both		15-49		4845	
713	Congo	1986	Enquête Brazzaville 1986	Community	urban	5-50	5-50	129	1005	
714	Congo	1987	Enquête Nationale Congo 1987	National	rural		13-49		1356	
715	Congo	1987	Maire et al., Rev Epidemiol Sante Publique 40:252-58, 1992	Community	rural		16-45		750	
716	Congo	1991	Enquête Brazzaville 1991	Community	urban	5-90	5-90	2393	3149	
717	Congo	1996	Enquête Brazzaville 1996	Community	urban	5-90	5-90	2496	3073	
718	Congo	2004	STEPS	Community	urban	25-64	25-64	1013	956	
719	Congo	2005	DHS	National	both		15-49		6266	
720	Congo	2011-2012	DHS	National	both		15-49		5060	
721	Congo	2011-2012	Epidemiology of dementia in Central Africa: Brazzaville	Community	urban	65+	65+	171	262	
722	Congo	2011-2012	Epidemiology of dementia in Central Africa: Gamboma	Community	rural	65+	65+	187	299	
723	Cook Islands	2003	STEPS	National	both	25-64	25-64	925	958	
724	Cook Islands	2011	Global School-based Student Health Survey	National	both	13-17	13-17	530	543	
725	Cook Islands	2013-2015	STEPS	National	both	18-64	18-64	456	469	
726	Cook Islands	2015	Global School-based Student Health Survey	National	both	13-17	13-17	304	313	
727	Cook Islands	2022	STEPS	National	both	18-69	18-69	681	713	
728	Costa Rica	2004	CAMDI	Community	urban	20+	20+	304	624	
729	Costa Rica	2004-2006	Costa Rican Longevity and Healthy Aging Study Pre-1945 Cohort Wave 1	National	both	60+	60+	1163	1346	
730	Costa Rica	2006-2008	Costa Rican Longevity and Healthy Aging Study Pre-1945 Cohort Wave 2	National	both	62+	62+	944	1102	
731	Costa Rica	2008-2009	Encuesta Nacional de Nutricion 2008-2009	National	both		45-64		661	
732	Costa Rica	2009	Global School-based Student Health Survey	National	both	13	13-17	356	1308	
733	Costa Rica	2009-2010	Costa Rican Longevity and Healthy Aging Study Pre-1945 Cohort Wave 3	National	both	64+	64+	737	887	
734	Costa Rica	2010	Costa Rican National Cardiovascular Risk Factors Survey, 2010	National	both	20+	20+	778	1958	
735	Costa Rica	2010-2011	Costa Rican Longevity and Healthy Aging Study 1945-1955 Cohort Wave 1	National	both	54-66	54-66	1058	1676	
736	Costa Rica	2012-2014	Costa Rican Longevity and Healthy Aging Study 1945-1955 Cohort Wave 2	National	both	56-68	56-68	867	1470	
737	Costa Rica	2014	Costa Rican National Cardiovascular Risk Factors Survey, 2014	National	both	20+	20+	1003	2196	

	Country	Study years	Survey/Study name/Citation	Level of representative-ness	Rural, urban, or both	Age range as in NCD-RisC database		Sample size		Note
						Male	Female	Male	Female	
738	Costa Rica	2014-2015	Latin American Study of Nutrition and Health (ELANS)	National	urban	15-65	15-65	397	403	
739	Costa Rica	2016	Censo Escolar peso y talla	National	both	6-12	6-12	178416	168958	
740	Cote d'Ivoire	1985-1986	Côte d'Ivoire Living Standards Survey (CILSS)	National	both	5+	5+	1860	1988	
741	Cote d'Ivoire	1986-1987	Côte d'Ivoire Living Standards Survey (CILSS)	National	both	5+	5+	4489	4615	
742	Cote d'Ivoire	1987-1988	Côte d'Ivoire Living Standards Survey (CILSS)	National	both	5+	5+	4016	4065	
743	Cote d'Ivoire	1988-1989	Côte d'Ivoire Living Standards Survey (CILSS)	National	both	5+	5+	3677	3701	
744	Cote d'Ivoire	1994	DHS	National	both		20-49		2682	
745	Cote d'Ivoire	1998-1999	DHS	National	both		15-49		2740	
746	Cote d'Ivoire	2005	STEPS	Subnational	rural	15-64	15-64	894	1022	
747	Cote d'Ivoire	2005	STEPS	Subnational	urban	15-64	15-64	1071	1437	
748	Cote d'Ivoire	2011-2012	DHS	National	both		15-49		4601	
749	Cote d'Ivoire	2014	Prevalence and factors associated with obesity in a periurban west African population	Community	urban	18+	18+	153	317	
750	Cote d'Ivoire	2016	Multiple Indicator Cluster Survey	National	both		15-49		5283	
751	Cote d'Ivoire	2021	DHS	National	both		15-49		6767	
752	Croatia	1997-1999	Croatian Health Survey	National	both	18+	18+	1972	2983	
753	Croatia	2002-2007	Epidemiology of arterial hypertension in Croatia (EH-UH)	National	both	18+	18+	446	600	
754	Croatia	2003-2004	School Health Survey	National	both	7-17	7-17	1302	1153	
755	Croatia	2005	Endemic Nephropathy and Arterial hypertension (ENAH)	Subnational	rural	18+	18+	264	367	
756	Croatia	2006-2008	The cardiovascular risk factors in school age - intervention model development	National	both	6-20	6-20	6011	5625	
757	Croatia	2008	Endemic Nephropathy and Arterial hypertension (ENAH)	Subnational	rural	18+	18+	331	527	
758	Croatia	2010	Endemic Nephropathy and Arterial hypertension (ENAH)	Subnational	rural	18+	18+	252	393	
759	Croatia	2014	Croatian Physical Activity in Adolesence Longitudinal Study (CRO-PALS)	Community	both	14-17	14-17	428	410	
760	Croatia	2015	Endemic Nephropathy and Arterial hypertension (ENAH) Follow-up Study	Subnational	rural	18+	18+	224	460	
761	Croatia	2015-2016	Childhood Obesity Surveillance Initiative 4	National	both	8	8	1364	1364	
762	Croatia	2015-2019	Motor skills in preschool children	National	both	5-7	5-7	405	382	
763	Croatia	2016	Prevalence of Overweight and Obesity among Primary School Students in Split, Croatia	Community	urban	6-7	6-7	209	218	
764	Croatia	2016-2017	Croatian Physical Activity in Adolesence Longitudinal Study (CRO-PALS)	Community	both	17-20	17-20	383	384	
765	Croatia	2018-2021	Epidemiology of arterial hypertension in Croatia (EH-UH)	National	both	18+	18+	381	622	
766	Croatia	2019	Childhood Obesity Surveillance Initiative 5	National	both	8-9	8-9	2778	2677	
767	Croatia	2021	Prevalence of Overweight and Obesity among Primary School Students in Split, Croatia	Community	urban	11-12	11-12	209	218	
768	Croatia	2022	Childhood Obesity Surveillance Initiative 6	National	both	8-9	8-9	2552	2452	
769	Croatia	2023	Prevalence of Overweight and Obesity among Primary School Students in Split, Croatia	Community	urban	13-14	13-14	209	218	
770	Cuba	1981-1982	Berdasco, Eur J Clin Nutr 1994; 48 Suppl 3:S155-63; discussion S64, 1994	Subnational	both	20-59	20-59	11355	18708	
771	Cuba	1991	Non communicable disease risk factors in Cienfuegos	Community	urban	15+	15+	527	600	
772	Cuba	1999-2000	The Survey on Health, Well-Being, and Aging in Latin America and the Caribbean (SABE)	Community	urban	60+	60+	630	1044	4
773	Cuba	2001	Non communicable disease risk factors in Cienfuegos	Community	urban	15-74	15-74	727	888	
774	Cuba	2001	National Survey on Risk Factors and Chronic Diseases (NSRFCD)	National	urban	15+	15+	10163	11376	
775	Cuba	2010	National Survey on Risk Factors and Chronic Diseases (NSRFCD)	National	both	15+	15+	3344	3868	
776	Cuba	2010-2011	Noncommunicable disease risk factors in Cienfuegos	Community	urban	15-74	15-74	611	873	
777	Cuba	2018-2020	Encuesta nacional de salud Cuba	National	both	6+	6+	5991	7479	
778	Cyprus	1999-2000	Countrywide Integrated Noncommunicable Diseases Intervention Programme Cyprus	Subnational	both	25-65	25-65	457	546	
779	Cyprus	2007-2008	Childhood asthma and atopy in Cyprus	Subnational	both	7-9, 13-15	7-9, 13-15	566	590	
780	Cyprus	2007-2010	Identification and prevention of Dietary- and lifestyle-induced health EFfects In Children and infantS (IDEFICS)	Community	urban	5-9	5-9	1129	1106	
781	Cyprus	2015-2016	Childhood Obesity Surveillance Initiative 4	National	urban	6-9	6-9	685	623	
782	Cyprus	2019	Childhood Obesity Surveillance Initiative 5	National	both	6-10	6-10	1001	994	
783	Cyprus	2022	Childhood Obesity Surveillance Initiative 6	National	both	6-10	6-10	977	1007	
784	Czechia	1985	Czech-MONICA	National	both	25-64	25-64	1243	1303	
785	Czechia	1988	Czech-MONICA	National	both	25-64	25-64	1357	1408	
786	Czechia	1992	Czech-MONICA	National	both	25-64	25-64	1131	1207	
787	Czechia	1997-1998	Czech post-MONICA	National	both	25-64	25-64	1527	1665	
788	Czechia	1998-2002	Health, Lifestyle and the Environment	National	urban	45-54	45-54	1539	2044	
789	Czechia	1999-2000	ELSPAC (The European Longitudinal Study of Pregnancy and Childhood)	Community	both	8	8	445	443	
790	Czechia	2000-2001	Czech post-MONICA	National	both	25-64	25-64	1628	1690	
791	Czechia	2001	6th Nationwide Anthropometric Survey of Children and Adolescents 2001	National	both	5-19	5-20	18960	22523	
792	Czechia	2002-2003	ELSPAC (The European Longitudinal Study of Pregnancy and Childhood)	Community	both	11	11	543	519	
793	Czechia	2002-2005	Health, Alcohol and Psychosocial Factors In Eastern Europe	Subnational	urban	45-70	45-70	3289	3901	
794	Czechia	2004-2005	ELSPAC (The European Longitudinal Study of Pregnancy and Childhood)	Community	both	13	13	439	362	
795	Czechia	2004-2005	Health, Lifestyle and the Environment	National	urban	45-54	45-54	775	1072	
796	Czechia	2006-2007	ELSPAC (The European Longitudinal Study of Pregnancy and Childhood)	Community	both	15	15	516	456	
797	Czechia	2006-2009	Czech post-MONICA	National	both	25-64	25-64	1717	1861	
798	Czechia	2008	Childhood Obesity Surveillance Initiative 1	National	both	6-7	6-7	834	838	
799	Czechia	2009	Health, Lifestyle and the Environment	National	urban	45-54	45-54	307	447	
800	Czechia	2009-2010	ELSPAC (The European Longitudinal Study of Pregnancy and Childhood)	Community	both	18	18	262	277	

	Country	Study years	Survey/Study name/Citation	Level of representative-ness	Rural, urban, or both	Age range as in NCD-RisC database		Sample size		Note
						Male	Female	Male	Female	
801	Czechia	2010	Childhood Obesity Surveillance Initiative 2	National	both	6-7	6-7	1203	1239	
802	Czechia	2010-2011	ELSPAC (The European Longitudinal Study of Pregnancy and Childhood)	Community	both	19	19	127	147	
803	Czechia	2013	Childhood Obesity Surveillance Initiative 3	National	both	6-7	6-7	1267	1200	
804	Czechia	2014-2015	European Heath Examination Survey	National	both	25-64	25-64	473	691	
805	Czechia	2015-2016	Childhood Obesity Surveillance Initiative 4	National	both	6-7	6-7	809	883	
806	Czechia	2015-2018	Czech post-MONICA	National	both	25-64	25-64	1220	1345	
807	Czechia	2019-2020	European Heath Examination Survey	National	both	25-64	25-64	425	627	
808	Czechia	2019-2022	CELSPAC: YA (The Central European Longitudinal Studies of Parents and Children: Young Adults)	Community	both	27-30	27-30	147	155	
809	Czechia	2020	Childhood Obesity Surveillance Initiative 5	National	both	6-7	6-7	1149	1112	
810	Czechia	2023-2024	Childhood Obesity Surveillance Initiative 6	National	both	6-7	6-7	555	538	
811	Denmark	1977	The Danish Conscription Database	National	both	17-26		25710		1
812	Denmark	1978	The Danish Conscription Database	National	both	17-26		13580		1
813	Denmark	1979	The Danish Conscription Database	National	both	17-26		6661		1
814	Denmark	1980	Copenhagen School Health Records Register	Subnational	urban	6-13	6-13	11234	11194	
815	Denmark	1980	The Danish Conscription Database	National	both	17-26		2897		
816	Denmark	1981	Copenhagen School Health Records Register	Subnational	urban	6-13	6-13	11433	11349	
817	Denmark	1981	The Danish Conscription Database	National	both	17-26		1752		
818	Denmark	1981-1983	Copenhagen City Heart Study	Subnational	urban	20-98	20-98	5651	6967	
819	Denmark	1982	Copenhagen School Health Records Register	Subnational	urban	6-13	6-13	10971	10746	
820	Denmark	1982	The Danish Conscription Database	National	both	17-26		1186		
821	Denmark	1982-1984	MONICA I (baseline), The Glostrup Population Studies	Subnational	urban	30-61	30-61	1940	1844	
822	Denmark	1983	Copenhagen School Health Records Register	Subnational	urban	6-13	6-13	7956	7945	
823	Denmark	1983	The Danish Conscription Database	National	both	17-26		762		
824	Denmark	1984	Copenhagen School Health Records Register	Subnational	urban	6-13	6-13	4886	4947	
825	Denmark	1984	The Danish Conscription Database	National	both	17-26		379		
826	Denmark	1984-1985	The Epidemiology of Gallstones in a 70 Year-Old Danish Population	Community	both	70	70	187	157	
827	Denmark	1985	Copenhagen School Health Records Register	Subnational	urban	6-13	6-13	4266	4275	
828	Denmark	1985	INTERSALT	Community	urban	20-59	20-59	99	100	
829	Denmark	1986	Copenhagen School Health Records Register	Subnational	urban	6-13	6-13	4533	4462	
830	Denmark	1986-1987	MONICA II, The Glostrup Population Studies	Subnational	urban	29-61	29-61	746	753	
831	Denmark	1987	Copenhagen School Health Records Register	Subnational	urban	6-13	6-13	4686	4544	
832	Denmark	1987	Nilsson et al., J Intern Med 237:479-86, 1995	Community	urban	51		439		
833	Denmark	1987-1988	MONICA I (5-year follow-up), The Glostrup Population Studies	Subnational	urban	35-66	35-66	1524	1463	
834	Denmark	1988	Copenhagen School Health Records Register	Subnational	urban	6-13	6-13	4738	4501	
835	Denmark	1989	Copenhagen School Health Records Register	Subnational	urban	6-13	6-13	4845	4598	
836	Denmark	1990	Copenhagen School Health Records Register	Subnational	urban	6-13	6-13	4669	4449	
837	Denmark	1991	Copenhagen School Health Records Register	Subnational	urban	6-13	6-13	4844	4811	
838	Denmark	1991-1992	Monica III, the Glostrup population studies	Subnational	both	29-71	29-71	1009	998	
839	Denmark	1991-1994	Copenhagen City Heart Study	Subnational	urban	21-98	21-98	4287	5430	
840	Denmark	1992	Copenhagen School Health Records Register	Subnational	urban	6-13	6-13	5243	5104	
841	Denmark	1993	Copenhagen School Health Records Register	Subnational	urban	6-13	6-13	4968	4890	
842	Denmark	1993-1994	MONICA I (10-year follow-up), The Glostrup Population Studies	Subnational	urban	41-72	41-72	1333	1323	
843	Denmark	1993-1997	EPIC Aarhus	Community	urban	50-65	50-65	8430	8717	
844	Denmark	1993-1997	EPIC Copenhagen	Community	urban	50-65	50-65	18729	21133	
845	Denmark	1994	Copenhagen School Health Records Register	Subnational	urban	6-13	6-13	4065	4005	
846	Denmark	1995	Copenhagen School Health Records Register	Subnational	urban	6-13	6-13	5437	5379	
847	Denmark	1996	Copenhagen School Health Records Register	Subnational	urban	6-13	6-13	4674	4670	
848	Denmark	1996-1997	Drivsholm et al., Diabet Med 18:126-32, 2001	Subnational	urban	60	60	325	370	
849	Denmark	1997	Copenhagen School Health Records Register	Subnational	urban	7-13	7-13	4105	4025	
850	Denmark	1997-1998	European Youth Heart Study	Community	urban	8-18	8-18	485	532	
851	Denmark	1998	Copenhagen School Health Records Register	Subnational	urban	8-13	8-13	3203	3253	
852	Denmark	1999	Copenhagen School Health Records Register	Subnational	urban	9-13	9-13	2860	2777	
853	Denmark	2000	Copenhagen School Health Records Register	Subnational	urban	10-13	10-13	1911	1923	
854	Denmark	2001	Copenhagen School Health Records Register	Subnational	urban	11-13	11-13	1594	1595	
855	Denmark	2001-2002	The Copenhagen School Child Intervention Study	Community	urban	5-8	5-8	362	329	
856	Denmark	2001-2003	Copenhagen City Heart Study	Subnational	urban	20-97	20-97	2574	3459	
857	Denmark	2002	Copenhagen School Health Records Register	Subnational	urban	12-13	12-13	860	895	
858	Denmark	2002-2003	Odense Androgen Study	Community	urban	20-29		783		
859	Denmark	2003	Copenhagen School Health Records Register	Subnational	urban	13	13	322	369	
860	Denmark	2003-2004	European Youth Heart Study	Community	urban	8-17	8-17	392	509	
861	Denmark	2003-2004	Copenhagen General Population Study 1	Subnational	urban	20+	20+	4878	5412	
862	Denmark	2004-2005	The Copenhagen School Child Intervention Study	Community	urban	8-11	8-11	121	130	
863	Denmark	2005	Copenhagen General Population Study 1	Subnational	urban	20+	20+	5210	6049	

	Country	Study years	Survey/Study name/Citation	Level of representative-ness	Rural, urban, or both	Age range as in NCD-RisC database		Sample size		Note
						Male	Female	Male	Female	
864	Denmark	2006	The Danish Conscription Database	National	both	17-26		25063		
865	Denmark	2006	Copenhagen General Population Study 1	Subnational	urban	20+	20+	5124	4886	
866	Denmark	2006-2008	The Health2006 Cohort	Subnational	urban	18-69	18-69	1511	1877	
867	Denmark	2007	The Danish Conscription Database	National	both	17-26		27194		
868	Denmark	2007	Copenhagen General Population Study 1	Subnational	urban	20+	20+	4119	7027	
869	Denmark	2007-2008	The Danish Health Examination Survey 2007-2008	National	both	18+	18+	7349	10651	
870	Denmark	2008	The Childhood Health Activity and Motor Performance School Study	Community	both	5-10	5-10	544	626	
871	Denmark	2008	The Danish Conscription Database	National	both	17-26		24538		
872	Denmark	2008	The Copenhagen School Child Intervention Study	Community	urban	12-14	12-14	99	111	
873	Denmark	2008	Copenhagen General Population Study 1	Subnational	urban	20+	20+	4790	6526	
874	Denmark	2009	The Childhood Health Activity and Motor Performance School Study - Fall	Community	both	6-12	6-12	240	246	
875	Denmark	2009	The Childhood Health Activity and Motor Performance School Study - Spring	Community	both	6-11	6-11	231	243	
876	Denmark	2009	The Danish Conscription Database	National	both	17-26		27093		
877	Denmark	2009	Copenhagen General Population Study 1	Subnational	urban	20+	20+	4244	5278	
878	Denmark	2009-2010	European Youth Heart Studyy	Community	both	14-28	14-28	481	553	
879	Denmark	2010	The Childhood Health Activity and Motor Performance School Study - Fall	Community	both	7-13	7-13	254	248	
880	Denmark	2010	The Childhood Health Activity and Motor Performance School Study - Spring	Community	both	7-12	7-12	234	235	
881	Denmark	2010	The Danish Conscription Database	National	both	17-26		30814		
882	Denmark	2010	Copenhagen General Population Study 1	Subnational	urban	20+	20+	4020	4924	
883	Denmark	2011	The Childhood Health Activity and Motor Performance School Study	Community	both	8-13	8-13	245	241	
884	Denmark	2011	The Danish Conscription Database	National	both	17-26		30719		
885	Denmark	2011	Copenhagen General Population Study 1	Subnational	urban	20+	20+	4974	5875	
886	Denmark	2011-2012	The OPUS School Meal Study	Subnational	both	8-11	8-11	427	388	
887	Denmark	2011-2012	The Health2006 Cohort	Subnational	urban	24-74	24-74	1031	1228	
888	Denmark	2012	The Childhood Health Activity and Motor Performance School Study	Community	both	9-14	9-14	273	249	
889	Denmark	2012	The Danish Conscription Database	National	both	17-26		29651		
890	Denmark	2012	Copenhagen General Population Study 1	Subnational	urban	20+	20+	4570	5479	
891	Denmark	2012-2015	DanFunD (baseline), The Glostrup Population Studies	Subnational	urban	18-72	18-72	3451	4034	
892	Denmark	2013	The Childhood Health Activity and Motor Performance School Study	Community	both	10-15	10-15	225	208	
893	Denmark	2013	The Danish Conscription Database	National	both	17-26		30565		
894	Denmark	2013	Learning, Cognition and Motion (LCoMotion)	Subnational	both	11-14	11-14	353	365	
895	Denmark	2013	Copenhagen General Population Study 1	Subnational	urban	20+	20+	4063	4749	
896	Denmark	2014	The Danish Conscription Database	National	both	17-26		32397		
897	Denmark	2014-2015	Copenhagen General Population Study 1	Subnational	urban	20+	20+	2858	3479	
898	Denmark	2014-2015	Copenhagen General Population Study 2	Subnational	urban	20+	20+	5492	6889	
899	Denmark	2015	The Childhood Health Activity and Motor Performance School Study	Community	both	12-17	12-17	122	120	
900	Denmark	2015	The Danish Conscription Database	National	both	17-26		28907		
901	Denmark	2015-2016	Childhood Obesity Surveillance Initiative 4	National	both	6-7	6-7	1339	1281	
902	Denmark	2016	The Danish Conscription Database	National	both	17-29		29057		
903	Denmark	2016	Copenhagen General Population Study 2	Subnational	urban	20+	20+	4488	5493	
904	Denmark	2017	The Danish Conscription Database	National	both	17-29		31057		
905	Denmark	2017	Copenhagen General Population Study 2	Subnational	urban	20+	20+	3362	4619	
906	Denmark	2018	The Danish Conscription Database	National	both	17-29		27597		
907	Denmark	2018	Copenhagen General Population Study 2	Subnational	urban	20+	20+	3315	4446	
908	Denmark	2019	Childhood Obesity Surveillance Initiative 5	National	both	6-7	6-7	1204	1291	
909	Denmark	2019	The Danish Conscription Database	National	both	17-29		25412		
910	Denmark	2019	Copenhagen General Population Study 2	Subnational	urban	20+	20+	3659	4512	
911	Denmark	2020-2021	Copenhagen General Population Study 2	Subnational	urban	20+	20+	3776	4355	
912	Denmark	2022-2023	Childhood Obesity Surveillance Initiative 6	National	both	6-7	6-7	1101	1135	
913	Denmark	2022-2023	Copenhagen General Population Study 2	Subnational	urban	20+	20+	5646	6590	
914	Dominica	2007-2008	STEPS	National	both	15-64	15-64	459	568	
915	Dominica	2009	Global School-based Student Health Survey	National	both	13-17	13-17	508	542	
916	Dominican Republic	1991	DHS	National	both		20-49		1965	
917	Dominican Republic	1993	Aono et al., J Epidemiol 7(4):238-43, 1997	National	both	20-70	20-70	767	1149	
918	Dominican Republic	1996	DHS	National	both		15-49		7441	
919	Dominican Republic	1996-1998	Estudio factores de riesgo cardiovascular y sindrome metabolico en la Republica Dominicana I (EFRICARD I)	National	both	18-75	18-75	2087	4095	
920	Dominican Republic	2010-2012	Estudio factores de riesgo cardiovascular y sindrome metabolico en la Republica Dominicana II (EFRICARD II)	National	both	18-75	18-75	1599	3180	
921	Dominican Republic	2013	DHS	National	both	15-59	15-49	10433	8960	
922	Dominican Republic	2017	Prevalencia de hta y factores de riesgo en La Republica Dominicana al 2017 (ENPREFAR HAS 17)	National	both	18+	18+	1001	1008	
923	DR Congo	2001	Multiple Indicator Cluster Survey 2	National	both		15-49		5520	
924	DR Congo	2005	STEPS	Subnational	urban	15+	15+	761	1152	
925	DR Congo	2007	DHS	National	both		15-49		4137	
926	DR Congo	2007	Diabetes and intermediate hyperglycaemia in Kisantu, DR Congo: a cross-sectional prevalence study	Community	urban	20+	20+	653	1199	

	Country	Study years	Survey/Study name/Citation	Level of representative-ness	Rural, urban, or both	Age range as in NCD-RisC database		Sample size		Note
						Male	Female	Male	Female	
927	DR Congo	2008	Visite de la Tension Artérielle et des Facteurs de Risque Associés en Afrique subsaharienne (VITARAA) - Kinshasa, RD Congo	Community	urban	10+	10+	810	979	
928	DR Congo	2008	Visite de la Tension Artérielle et des Facteurs de Risque Associés en Afrique subsaharienne (VITARAA) - Sud-Kivu, RD Congo	Community	both	20+	20+	246	408	
929	DR Congo	2013-2014	DHS	National	both		15-49		8163	
930	DR Congo	2016-2017	Prevalence and Risk Factors of CKD in South Kivu, Democratic Republic of Congo: A Large-Scale Population Study	Subnational	both	18+	18+	499	791	
931	DR Congo	2017	Biocultural determinants of overweight-obesity among adult women experiencing the nutritional transition in the Democratic Republic of Congo	Subnational	both		20+		431	
932	DR Congo	2019	Diabetes prevalence and risk factors	Community	rural	19+	19+	721	807	
933	Ecuador	2004	Encuesta Demografica y de Salud Materno e Infantil/ Reproductive Health Survey	National	both		15-49		3850	
934	Ecuador	2004-2005	CArdiovascular Risk factors Multiple Evaluation in Latin America (CARMELA)	Community	urban	25-64	25-64	813	814	
935	Ecuador	2008-2009	Food Nutrition and Health	Community	both	10-16	10-16	379	375	
936	Ecuador	2009-2010	The Survey on Health, Well-Being, and Aging in Latin America and the Caribbean (SABE)	National	both	60+	60+	2341	2595	
937	Ecuador	2011-2013	Encuesta Nacional de Salud y Nutrición	National	both	5-59	5-59	22919	25767	
938	Ecuador	2013-2014	Encuesta de Condiciones de Vida	National	both	5+	5+	46049	47302	
939	Ecuador	2014-2015	Latin American Study of Nutrition and Health (ELANS)	National	urban	15-65	15-65	517	586	
940	Ecuador	2018	Encuesta Nacional de Salud y Nutrición	National	both	5+	5+	63176	65597	
941	Ecuador	2018	STEPS	National	both	18-69	18-69	1893	2579	
942	Ecuador	2024	Global School-based Student Health Survey	National	both	13-17	13-17	3636	3733	
943	Egypt	1992	DHS	National	both		20-49		4654	
944	Egypt	1995	DHS	National	both		20-49		6499	
945	Egypt	2000	DHS	National	both		20-49		13602	
946	Egypt	2002	National Survey of Smoking, Obesity, Blood Pressure and Blood Glucose	National	both	6+	6+	4397	5161	
947	Egypt	2003	DHS	National	both		20-49		7930	
948	Egypt	2003-2004	Marzouk et al., Gut 56(8):1105-10, 2007	Community	rural	25+	25+	322	456	
949	Egypt	2005	STEPS	National	both	15-65	15-65	4757	4428	
950	Egypt	2005	DHS	National	both		20-49		16864	
951	Egypt	2007-2009	Mostafa et al., Gut 59(8):1135-40, 2010	Community	rural	35+	35+	642	843	
952	Egypt	2008	DHS	National	both	10-59	20-49	14261	15242	
953	Egypt	2011	STEPS	National	both	15-65	15-65	1761	2977	
954	Egypt	2011	Global School-based Student Health Survey	National	both	13-17	13-17	325	454	
955	Egypt	2014	DHS	National	both		20-49		18891	
956	Egypt	2015	DHS	National	both	15-59	15-59	7235	8471	
957	Egypt	2017	STEPS	National	both	15-69	15-69	2273	3692	
958	Egypt	2021-2022	Egypt Family Health Survey	National	both	5-14	5-49	11362	30029	
959	El Salvador	2002-2003	Encuesta Nacional de Salud Familiar	National	both		15-49		3885	
960	El Salvador	2004	CAMDI	Community	urban	20+	20+	396	811	
961	El Salvador	2008	Encuesta Nacional de Salud Familiar	National	both		15-49		6808	
962	El Salvador	2013	Global School-based Student Health Survey	National	both		13-15		675	
963	El Salvador	2014-2015	Encuesta Nacional de Enfermedades Crónicas (ENECA-ELS)	National	both	20+	20+	1684	2945	
964	El Salvador	2016	National height and weight census	National	both	6-8	6-8	56034	53321	
965	Equatorial Guinea	2011	DHS	National	both		15-49		1074	
966	Eritrea	2002	DHS	National	both		15-49		3647	
967	Eritrea	2004	STEPS	National	both	15-64	15-64	1113	1089	
968	Eritrea	2010	Eritrea Population and Health Survey	National	both	15-59	15-49	5218	10343	
969	Eritrea	2010	STEPS	National	both	25-74	25-74	1712	4285	
970	Estonia	1984-1986	Abina et al., Blood Press 12:111-21, 2003	Community	urban	20-54	30-54	2477	851	
971	Estonia	1992-1994	Abina et al., Blood Press 12:111-21, 2003	Community	urban	20-54	20-54	921	678	
972	Estonia	1997	Pomerleau et al., Public Health Nutrition 3(1):3-10, 2000	National	both	19-64	19-64	525	629	
973	Estonia	1999-2001	Abina et al., Blood Press 12:111-21, 2003	Community	urban	20-54	20-54	635	692	
974	Estonia	2002	Estonian Biobank	National	both	18+	18+	89	217	
975	Estonia	2003	The European Male Ageing Study	Community	both	40+		416		
976	Estonia	2003	Estonian Biobank	National	both	18+	18+	2695	5688	
977	Estonia	2004	Estonian Biobank	National	both	18+	18+	527	947	
978	Estonia	2007	Estonian Biobank	National	both	18+	18+	1000	2187	
979	Estonia	2007-2010	Identification and prevention of Dietary- and lifestyle-induced health EFfects In Children and infantS (IDEFICS)	Community	urban	5-9	5-9	558	634	
980	Estonia	2008	The European Male Ageing Study	Community	both	45+		305		
981	Estonia	2008	Estonian Biobank	National	both	18+	18+	5147	10990	
982	Estonia	2009	Estonian Biobank	National	both	18+	18+	3963	6493	
983	Estonia	2010	Estonian Biobank	National	both	18+	18+	4052	7045	
984	Estonia	2013-2015	National Dietary Survey (RTU) 2014	National	both	5-74	5-74	1260	2202	
985	Estonia	2015-2016	Childhood Obesity Surveillance Initiative 4	National	both	7-8	7-8	6502	6198	
986	Estonia	2018-2019	Childhood Obesity Surveillance Initiative 5	National	both	7-11	7-11	6066	6038	
987	Estonia	2022	Childhood Obesity Surveillance Initiative 6	National	both	7-14	7-14	9177	8792	
988	Eswatini	2006-2007	DHS	National	both	15-49	15-49	4074	4714	

	Country	Study years	Survey/Study name/Citation	Level of representative-ness	Rural, urban, or both	Age range as in NCD-RisC database		Sample size		Note
						Male	Female	Male	Female	
989	Eswatini	2014	STEPS	National	both	15-69	15-69	1102	1976	
990	Eswatini	2024	STEPS	National	both	18-69	18-69	1806	2690	
991	Ethiopia	2000	DHS	National	both		15-49		13912	
992	Ethiopia	2005	DHS	National	both		15-49		6133	
993	Ethiopia	2006	STEPS	Subnational	urban	25-64	25-64	1642	2295	
994	Ethiopia	2011	DHS	National	both	15-59	15-49	14329	15111	
995	Ethiopia	2014	The Ethiopian Socioeconomic Survey (ESS)	National	both	5-6	5-6	614	603	12
996	Ethiopia	2015	STEPS	National	both	15-69	15-69	3912	5369	
997	Ethiopia	2015	National Micronutrient Survey	National	both	5-54	5-49	1187	2459	
998	Ethiopia	2016	DHS	National	both	15-59	15-49	12380	14104	
999	Ethiopia	2016	ARISE Network Adolescent Health Study (Harar)	Community	urban	10-19	10-19	499	556	
1000	Ethiopia	2016	ARISE Network Adolescent Health Study (Kersa)	Community	both	10-19	10-19	526	418	
1001	Ethiopia	2018	Sustainable Urban Diets (SUDS) Addis Ababa	Community	urban		18-49		994	
1002	Ethiopia	2018	Sustainable Urban Diets (SUDS) Kersa	Community	rural		18-49		1015	
1003	Ethiopia	2024	STEPS	National	both	18-69	18-69	3750	5192	
1004	Fiji	1980	National Cardiovascular and Diabetes Survey (NCVDS)	Subnational	both	20+	20+	1448	1522	
1005	Fiji	2002	STEPS	National	both	15-64	15-64	2684	3820	
1006	Fiji	2005-2007	Pacific Obesity Prevention in Communities - Healthy Youth Health Communities Study	Subnational	urban	11-19	11-19	3730	4109	
1007	Fiji	2007-2008	Pacific Obesity Prevention in Communities - Healthy Youth Health Communities Study	Subnational	urban	13-22	13-22	1492	1832	
1008	Fiji	2009	Fiji Eye Health Survey 2009	National	both	40+	40+	582	776	
1009	Fiji	2011	STEPS	National	both	25-64	25-64	1123	1417	
1010	Fiji	2016	Global School-based Student Health Survey	National	both	13-17	13-17	1394	1469	
1011	Fiji	2021	Fiji MICS6	National	both		15-49		4770	
1012	Fiji	2024-2025	STEPS	National	both	18-69	18-69	2157	2750	
1013	Finland	1980	Young Finns Study 1980	National	rural	5-18	5-18	752	814	
1014	Finland	1980	Young Finns Study 1980	National	urban	5-18	5-18	704	727	
1015	Finland	1982	MONICA, North Karelia/Kuopio/Turku/Loimaa	Subnational	both	25-64	25-64	4550	4659	
1016	Finland	1983	Young Finns Study 1983	National	rural	6-21	6-21	727	773	
1017	Finland	1983	Young Finns Study 1983	National	urban	6-21	6-21	656	685	
1018	Finland	1984	Finland, Italy, Netherlands, Elderly (FINE-Finland)	Community	rural	65-84		673		
1019	Finland	1984-1989	Kuopio Ischaemic Heart Disease Risk Factor Study	Subnational	both	42-61		2672		
1020	Finland	1985	INTERSALT, Turku	Community	urban	20-59	20-59	100	100	
1021	Finland	1985-1986	INTERSALT, Joensuu	Community	urban	20-59	20-59	100	100	
1022	Finland	1986	Young Finns Study 1986	National	rural	9-24	9-24	594	631	
1023	Finland	1986	Young Finns Study 1986	National	urban	9-24	9-24	587	666	
1024	Finland	1987	MONICA, North Karelia/Kuopio/Turku/Loimaa	Subnational	both	25-64	25-64	2896	3151	
1025	Finland	1989	Finland, Italy, Netherlands, Elderly (FINE-Finland)	Community	rural	70-89		446		
1026	Finland	1990-1992	Oulu 35 Study	Community	urban	56-57	56-57	231	326	
1027	Finland	1991-1993	Kuopio Ischaemic Heart Disease Risk Factor Study	Subnational	both	46-65		1037		
1028	Finland	1992	The National FINRISK Study	Subnational	both	25-64	25-64	2849	3140	
1029	Finland	1994	Finland, Italy, Netherlands, Elderly (FINE-Finland)	Community	rural	75-94		266		
1030	Finland	1996-1998	Oulu 35 Study	Community	urban	60-63	60-63	242	345	
1031	Finland	1996-1998	Savitaipale Study, Baseline	Community	rural	40-66	40-66	574	574	
1032	Finland	1997	The National FINRISK Study	National	both	25-74	25-74	4128	4056	
1033	Finland	1997	Northern Finland Birth Cohort 1966	Community	both	30-31	30-31	2614	141	
1034	Finland	1998-2001	Kuopio Ischaemic Heart Disease Risk Factor Study	Subnational	both	53-73	53-73	834	919	
1035	Finland	2000	Finland, Italy, Netherlands, Elderly (FINE-Finland)	Community	rural	81-96		92		
1036	Finland	2000-2001	Health 2000 Survey	National	both	30+	30+	2663	3222	
1037	Finland	2001	Young Finns Study 2001	National	rural	24-39	24-39	346	393	
1038	Finland	2001	Young Finns Study 2001	National	urban	24-39	24-39	658	769	
1039	Finland	2001-2002	Northern Finland Birth Cohort 1986	Community	both	15-17	15-17	3126	3194	
1040	Finland	2001-2003	Oulu 45 Study	Community	urban	55-58	55-58	426	550	
1041	Finland	2001-2004	Helsinki Birth Cohort Study	Community	urban	56-69	56-69	927	1074	
1042	Finland	2002	The National FINRISK Study	National	both	25-74	25-74	4070	4664	
1043	Finland	2004-2005	FIN-D2D	Subnational	both	45-74	45-74	1364	1461	
1044	Finland	2005	Mantyselka et al., Rheumatology (Oxford) 47(8):1235-38, 2008	Community	rural	30-65	30-65	230	241	
1045	Finland	2005-2008	Kuopio Ischaemic Heart Disease Risk Factor Study	Subnational	both	60-81	60-81	1241	634	
1046	Finland	2007	The National FINRISK Study	National	both	25-74	25-74	2934	3265	
1047	Finland	2007	Oulu 35 Study	Community	urban	71-73	71-73	182	271	
1048	Finland	2007	Young Finns Study 2007	National	rural	30-45	30-45	374	431	
1049	Finland	2007	Young Finns Study 2007	National	urban	30-45	30-45	602	714	
1050	Finland	2007-2008	Savitaipale Study, 10-year Follow-up	Community	rural	51-75	51-75	430	483	
1051	Finland	2008	Control group for Finnish male former elite athletes	National	both	61+		206		

	Country	Study years	Survey/Study name/Citation	Level of representative-ness	Rural, urban, or both	Age range as in NCD-RisC database		Sample size		Note
						Male	Female	Male	Female	
1052	Finland	2011	Young Finns Study 2011	National	rural	34-49	34-49	364	424	
1053	Finland	2011	Young Finns Study 2011	National	urban	34-49	34-49	506	636	
1054	Finland	2011-2012	Health 2011 Survey	National	both	30+	30+	2041	2535	
1055	Finland	2011-2013	International Study of Childhood Obesity, Lifestyle and the Environment (ISCOLE)	Community	urban	9-11	9-11	253	282	
1056	Finland	2012	The National FINRISK Study	National	both	25-74	25-74	2774	3004	
1057	Finland	2012	Northern Finland Birth Cohort 1966	Community	both	45-47	45-47	2362	2966	
1058	Finland	2016	Register of Primary Health Care visits	National	rural	5-17	5-17	37441	35048	
1059	Finland	2016	Register of Primary Health Care visits	National	urban	5-17	5-17	92177	87911	
1060	Finland	2017	Register of Primary Health Care visits	National	rural	5-17	5-17	41744	38890	
1061	Finland	2017	Register of Primary Health Care visits	National	urban	5-17	5-17	106968	100998	
1062	Finland	2017	The FinHealth Survey	National	both	18+	18+	2699	3133	
1063	Finland	2018	Register of Primary Health Care visits	National	rural	5-17	5-17	44828	41905	
1064	Finland	2018	Register of Primary Health Care visits	National	urban	5-17	5-17	112753	106296	
1065	Finland	2018-2019	Savitaipale Study, 22-year Follow-up	Community	rural	62-86	62-86	263	341	
1066	Finland	2018-2020	Young Finns Study: Follow-up	National	both	5+	5+	2766	3652	
1067	Finland	2019	Childhood Obesity Surveillance Initiative 5	National	both	7-9	7-9	5749	5096	
1068	Finland	2019-2020	Northern Finland Birth Cohort 1986	Community	both	33-35	33-35	579	916	
1069	Finland	2022	Childhood Obesity Surveillance Initiative 6	National	both	7-9	7-9	7543	6619	
1070	Finland	2022-2023	Healthy Finland Survey	National	both	20+	20+	2608	3047	
1071	France	1985-1987	MONICA, Strasbourg	Subnational	both	35-64	35-64	664	713	
1072	France	1985-1987	MONICA, Strasbourg	Subnational	both	25-34	25-34	65	78	
1073	France	1985-1987	MONICA, Toulouse	Subnational	both	35-64	35-64	675	644	
1074	France	1986-1989	MONICA, Lille	Subnational	urban	25-67	25-67	863	724	
1075	France	1988-1991	MONICA, Toulouse	Subnational	both	35-64		586		
1076	France	1994-1996	MONICA, Toulouse	Subnational	both	35-64	35-64	608	566	
1077	France	1995-1996	MONICA, Lille	Subnational	urban	36-67	36-67	598	590	
1078	France	1995-1997	MONICA, Strasbourg	Subnational	both	35-64	35-64	526	523	
1079	France	1996-2003	Jaquet et al., Diabetologia 48(5):849-55, 2005	Community	urban	15-34	15-34	173	164	
1080	France	1999-2001	The Three City Study	Community	urban	65+	65+	2423	3778	
1081	France	2000	Corpulence 7-9 ans	Subnational	both	7-9	7-9	786	796	
1082	France	2005-2007	Etude individuelle nationale des consommations alimentaires 2 (INCA2), children and adolescents	National	both	5-17	5-17	607	684	
1083	France	2005-2007	Etude individuelle nationale des consommations alimentaires 2 (INCA2), adults	National	both	18-79	18-79	1001	1368	
1084	France	2005-2007	National Monitoring of Arterial Risk in Bas-Rhin (MONA LISA Bas-Rhin)	Subnational	both	35-74	35-74	780	787	
1085	France	2005-2007	National Monitoring of Arterial Risk in Toulouse (MONA LISA Toulouse)	Subnational	both	35-74	35-74	829	796	
1086	France	2005-2008	National Monitoring of Arterial Risk in Lille (MONA LISA Lille)	Subnational	urban	35-75	35-75	783	795	
1087	France	2006-2007	Etude Nationale Nutrition Santé	National	both	5-74	5-74	1505	2030	13
1088	France	2006-2007	HELENA	Community	urban	12-17	12-17	122	165	
1089	France	2006-2008	The Three City Study	Community	urban	72+	72+	768	1217	
1090	France	2007	Corpulence 7-9 ans	National	both	7-9	7-9	1281	1244	
1091	France	2008-2011	Study of pre- and early postnatal determinants of child health and development (EDEN)	Community	both	5	5	639	569	
1092	France	2009-2012	Study of pre- and early postnatal determinants of child health and development (EDEN)	Community	both	6	6	306	283	
1093	France	2010-2013	Study of pre- and early postnatal determinants of child health and development (EDEN)	Community	both	7	7	339	320	
1094	France	2011-2013	Enquête LIttorale Souffle Air Biologie EnvironnemenT (ELISABET) Dunkerque	Community	urban	40-64	40-64	750	779	
1095	France	2011-2013	Enquête LIttorale Souffle Air Biologie EnvironnemenT (ELISABET) Lille	Community	urban	40-64	40-64	753	838	
1096	France	2011-2014	Study of pre- and early postnatal determinants of child health and development (EDEN)	Community	both	8	8	269	236	
1097	France	2012	Cohorte des consultants des Centres d’examens de santé (CONSTANCES)	National	both	18-69	18-69	3538	4351	
1098	France	2012-2015	Study of pre- and early postnatal determinants of child health and development (EDEN)	Community	both	9	9	86	79	
1099	France	2013-2015	Cohorte des consultants des Centres d’examens de santé (CONSTANCES)	National	both	18-69	18-69	6654	7273	
1100	France	2013-2016	Study of pre- and early postnatal determinants of child health and development (EDEN)	Community	both	10	10	193	178	
1101	France	2014-2016	Cohorte des consultants des Centres d’examens de santé (CONSTANCES)	National	both	18-69	18-69	9082	10020	
1102	France	2014-2016	L'Etude de Sante sur l'Environnement, la Biosurveillance, l'Activite physique et la Nutrition (Etude Esteban)	National	both	6-74	6-74	1662	1913	14
1103	France	2014-2017	Study of pre- and early postnatal determinants of child health and development (EDEN)	Community	both	11	11	246	230	
1104	France	2015-2016	Childhood Obesity Surveillance Initiative 4	National	both	7-9	7-9	2510	2561	
1105	France	2015-2018	Cohorte des consultants des Centres d’examens de santé (CONSTANCES)	National	both	18-69	18-69	12069	12456	
1106	France	2016-2018	Cohorte des consultants des Centres d’examens de santé (CONSTANCES)	National	both	18-69	18-69	10135	11750	
1107	France	2017-2019	Cohorte des consultants des Centres d’examens de santé (CONSTANCES)	National	both	18-69	18-69	11984	14250	
1108	France	2017-2019	Cohorte des consultants des Centres d’examens de santé (CONSTANCES)	Subnational	both	22-76	22-76	10443	11334	
1109	France	2018-2019	Cohorte des consultants des Centres d’examens de santé (CONSTANCES)	National	both	18-69	18-69	19540	22392	
1110	France	2020-2021	Cohorte des consultants des Centres d’examens de santé (CONSTANCES)	Subnational	both	18-69	18-69	1467	1813	
1111	France	2020-2022	Cohorte des consultants des Centres d’examens de santé (CONSTANCES)	Subnational	both	22-80	22-80	22053	23119	
1112	French Polynesia	2010	STEPS	National	both	18-64	18-64	1458	1916	
1113	French Polynesia	2015	Global School-based Student Health Survey	National	both	12-17	12-17	1310	1438	
1114	French Polynesia	2024	Global School-based Student Health Survey	National	both	12-17	12-17	1412	1492	

	Country	Study years	Survey/Study name/Citation	Level of representative-ness	Rural, urban, or both	Age range as in NCD-RisC database		Sample size		Note
						Male	Female	Male	Female	
1115	Gabon	2000	DHS	National	both		20-49		2082	
1116	Gabon	2009	STEPS	Subnational	urban	15-64	15-64	1051	1515	
1117	Gabon	2012	DHS	National	both		15-49		5066	
1118	Gabon	2019-2021	DHS	National	both		15-64		5663	
1119	Gambia	1996-1997	National Survey of Blindness and Low Vision	National	both	16+	16+	1733	2071	
1120	Gambia	2003	Siervo et al., Eur J Clin Nutr 60(4):455-63, 2006	Community	urban	14-50	14-50	50	50	
1121	Gambia	2010	STEPS	National	both	25-64	25-64	1610	1919	
1122	Gambia	2012	Gambia National Nutrition Survey	National	both		15-49		7745	
1123	Gambia	2012-2017	Kiang West Longitudinal Population Study	Subnational	rural	5+	5+	4103	5690	
1124	Gambia	2013	DHS	National	both		15-49		4180	
1125	Gambia	2018	The Gambia Micronutrient Survey (GMNS)	National	both		15-49		1640	
1126	Gambia	2019-2020	DHS	National	both		15-49		5474	
1127	Gambia	2023	STEPS	National	both	18-69	18-69	1590	3024	
1128	Georgia	2010	STEPS	National	both	18-64	18-64	1842	4460	
1129	Georgia	2015-2016	Childhood Obesity Surveillance Initiative 4	National	both	7-8	7-8	1685	1585	
1130	Georgia	2016	STEPS	National	both	18-69	18-69	1188	2784	
1131	Georgia	2019	Childhood Obesity Surveillance Initiative 5	National	both	7-8	7-8	1777	1646	
1132	Georgia	2022	Childhood Obesity Surveillance Initiative 6	National	both	7-8	7-8	1664	1510	
1133	Germany	1982	MONICA, Erfurt	Community	urban	25-64	25-64	106	103	
1134	Germany	1982-1984	MONICA, Chemnitz	Community	urban	25-64	25-64	267	295	
1135	Germany	1982-1984	MONICA, Zwickau	Community	urban	25-64	25-64	246	276	
1136	Germany	1982-1985	MONICA, Rest of Karl-Marx-Stadt County	Subnational	urban	25-64	25-64	592	657	
1137	Germany	1982-1985	MONICA, Rest of DDR-MONICA	Subnational	urban	25-64	25-64	235	232	
1138	Germany	1983-1984	MONICA, Halle County	Subnational	urban	25-64	25-64	1110	1172	
1139	Germany	1983-1987	MONICA, Rhein-Neckar Region	Community	urban	25-64	25-64	1489	1609	
1140	Germany	1984	MONICA, Bremen North/West	Community	urban	25-64	25-64	813	852	
1141	Germany	1984	The German Conscription Database	Subnational	both	19		419719		
1142	Germany	1984	German Cardiovascular Prevention Study (GCP) - National Health Survey 1984	Subnational	both	25-69	25-69	2415	2366	
1143	Germany	1984-1985	MONICA, Berlin-Lichtenberg	Community	urban	25-64	25-64	593	635	
1144	Germany	1984-1985	KORA-S1 (Cooperative Health Research in the Augsburg Region) - formerly MONICA Augsburg	Community	both	24-65	24-65	2006	1963	
1145	Germany	1984-1986	MONICA, Cottbus County	Community	urban	25-64	25-64	657	739	
1146	Germany	1985	The German Conscription Database	Subnational	both	19		402487		
1147	Germany	1985-1986	INTERSALT, Cottbus	Community	urban	20-59	20-59	99	99	
1148	Germany	1985-1986	INTERSALT, Heidelberg	Community	urban	20-59	20-59	97	99	
1149	Germany	1985-1986	INTERSALT, Bernried	Community	urban	20-59	20-59	99	98	
1150	Germany	1985-1986	CINDI	Subnational	both	25-64	25-64	1875	1990	
1151	Germany	1986	The German Conscription Database	Subnational	both	19		382632		
1152	Germany	1987	The German Conscription Database	Subnational	both	19		349083		
1153	Germany	1987-1988	MONICA, Erfurt	Community	urban	25-64	25-64	871	909	
1154	Germany	1988	MONICA, Berlin-Lichtenberg	Community	urban	25-64	25-64	690	728	
1155	Germany	1988	MONICA, Bremen North/West	Community	urban	25-69	25-69	619	632	
1156	Germany	1988	MONICA, Bremen Center/South/East	Community	urban	25-69	25-69	499	582	
1157	Germany	1988	MONICA, Chemnitz	Community	urban	25-64	25-64	288	382	
1158	Germany	1988	MONICA, Zwickau	Community	urban	25-64	25-64	193	250	
1159	Germany	1988	The German Conscription Database	Subnational	both	19		303265		
1160	Germany	1988	German Cardiovascular Prevention Study (GCP) - National Health Survey 1988	Subnational	both	25-69	25-69	2642	2678	
1161	Germany	1988-1989	MONICA, Halle County	Subnational	urban	25-64	25-64	959	1201	
1162	Germany	1988-1989	MONICA, Rest of Karl-Marx-Stadt County	Subnational	urban	25-64	25-64	541	626	
1163	Germany	1988-1989	CINDI	Subnational	both	25-64	25-64	1361	1435	
1164	Germany	1989	The German Conscription Database	Subnational	both	19		245740		
1165	Germany	1989-1990	MONICA, Cottbus County	Community	urban	25-64	25-64	539	529	
1166	Germany	1989-1990	KORA-S2 (Cooperative Health Research in the Augsburg Region) - formerly MONICA Augsburg	Community	both	24-75	24-75	2439	2374	
1167	Germany	1990	The German Conscription Database	Subnational	both	19		206599		
1168	Germany	1990-1992	European Community Respiratory Health Survey, Hamburg	Community	urban	20-47	20-47	146	138	
1169	Germany	1990-1992	European Community Respiratory Health Survey, Erfurt	Community	urban	20-47	20-47	146	124	
1170	Germany	1991	The German Conscription Database	National	both	19		138195		
1171	Germany	1991-1992	MONICA, Bremen North/West	Community	urban	25-69	25-69	599	671	
1172	Germany	1991-1992	MONICA, Bremen Center/South/East	Community	urban	25-69	25-69	524	546	
1173	Germany	1991-1992	MONICA, Erfurt	Community	urban	25-64	25-64	587	572	
1174	Germany	1991-1992	CINDI	Subnational	both	25-64	25-64	1326	1400	
1175	Germany	1991-1992	German Cardiovascular Prevention Study (GCP) - National Health Survey 1991	Subnational	both	25-69	25-69	2599	2670	
1176	Germany	1991-1992	First National Examination of life conditions, Environment and Health in East Germany 1991/92	Subnational	both	25-69	25-69	1042	1155	
1177	Germany	1992	The German Conscription Database	National	both	19		220956		

	Country	Study years	Survey/Study name/Citation	Level of representative-ness	Rural, urban, or both	Age range as in NCD-RisC database		Sample size		Note
						Male	Female	Male	Female	
1178	Germany	1993	The German Conscription Database	National	both	19		188655		
1179	Germany	1993-1994	MONICA, Chemnitz	Community	urban	25-64	25-64	408	424	
1180	Germany	1993-1994	MONICA, Zwickau	Community	urban	25-64	25-64	139	186	
1181	Germany	1994	The German Conscription Database	National	both	19		155426		
1182	Germany	1994-1995	KORA-S3 (Cooperative Health Research in the Augsburg Region) - formerly MONICA Augsburg	Community	both	24-75	24-75	2358	2372	
1183	Germany	1994-1998	EPIC Heidelberg	Community	urban	40-64	35-64	11680	13458	
1184	Germany	1994-1998	EPIC Potsdam	Community	urban	40-64	35-64	10224	15995	
1185	Germany	1995	The German Conscription Database	National	both	19		185762		
1186	Germany	1996	The German Conscription Database	National	both	19		191260		
1187	Germany	1997	The German Conscription Database	National	both	19		148738		
1188	Germany	1997-1999	German National Health Interview and Examination Survey (GNHIES98)	National	both	18-79	18-79	3406	3580	
1189	Germany	1997-2001	Study of Health in Pomerania (SHIP-START-0) baseline study	Subnational	both	20-80	20-80	2109	2168	15
1190	Germany	1998	The German Conscription Database	National	both	19		146528		
1191	Germany	1999	The German Conscription Database	National	both	19		292732		
1192	Germany	1999-2001	KORA-S4 (Cooperative Health Research in the Augsburg Region)	Community	both	24-75	24-75	2071	2138	
1193	Germany	2000-2001	European Community Respiratory Health Survey, Hamburg	Community	urban	30-57	30-57	146	138	
1194	Germany	2000-2001	European Community Respiratory Health Survey, Erfurt	Community	urban	30-57	30-57	146	124	
1195	Germany	2000-2002	Epidemiological study of the chances of prevention, early recognition and optimal treatment of chronic diseases in an elderly population (ESTHER)	Subnational	both	50-75	50-75	4344	5334	
1196	Germany	2000-2003	Heinz Nixdorf Recall Study	Subnational	urban	45-75	45-75	2380	2401	16
1197	Germany	2002	Echinoccoccus Multilocularis and Internal Diseases in Leutkirch	Community	urban	12-65	12-65	1172	1257	
1198	Germany	2002-2006	Study of Health in Pomerania (SHIP-START-1) 5-year follow-up	Subnational	both	25-85	25-85	1578	1691	15
1199	Germany	2003-2006	German Health Interview and Examination Survey for Children and Adolescents (KiGGS)	National	both	5-17	5-17	6600	6260	
1200	Germany	2005-2008	Heinz Nixdorf Recall Study	Subnational	both	50-80	50-80	2044	2099	
1201	Germany	2006-2007	HELENA	Community	urban	12-17	12-17	282	194	
1202	Germany	2006-2008	KORA-F4 (Cooperative Health Research in the Augsburg Region) - first follow up of KORA-S4	Community	both	31-82	31-82	1478	1580	
1203	Germany	2007-2010	Identification and prevention of Dietary- and lifestyle-induced health EFfects In Children and infantS (IDEFICS)	Community	urban	5-9	5-9	772	762	
1204	Germany	2007-2012	Gutenberg Health Study	Community	urban	35-74	35-74	7577	7419	
1205	Germany	2008	The German Conscription Database	National	both	19		98926		
1206	Germany	2008-2011	Epidemiological study of the chances of prevention, early recognition and optimal treatment of chronic diseases in an elderly population (ESTHER)	Subnational	both	58-84	58-84	1468	1622	
1207	Germany	2008-2011	German Health Interview and Examination Survey for Adults 2008-11 (DEGS1)	National	both	18-79	18-79	3389	3650	
1208	Germany	2008-2012	Study of Health in Pomerania (SHIP-START-2) 11-year follow-up	Subnational	both	31-81	31-81	1049	1201	15
1209	Germany	2008-2012	Study of Health in Pomerania, second cohort (SHIP-TREND-0)	Subnational	both	20-79	20-79	2099	2229	15
1210	Germany	2009	The German Conscription Database	National	both	19		111455		
1211	Germany	2009-2013	Join the Healthy Boat	Subnational	both	5-9	5-9	837	827	
1212	Germany	2010	The German Conscription Database	National	both	19		101911		
1213	Germany	2011-2014	Heinz Nixdorf Recall Study	Subnational	both	56-85	56-85	1493	1560	
1214	Germany	2012-2017	Gutenberg Health Study	Community	urban	40-80	40-80	6356	6062	
1215	Germany	2013-2014	KORA-FF4 (Cooperative Health Research in the Augsburg Region) - second follow up of KORA-S4	Community	both	38-88	38-88	1099	1176	
1216	Germany	2014-2016	Study of Health in Pomerania (SHIP-START-3) 16-year follow-up	Subnational	both	37-87	37-87	778	907	15
1217	Germany	2014-2017	German Health Interview and Examination Survey for Children and Adolescents (KiGGS) - Wave 2	National	both	5-17	5-17	1541	1584	
1218	Germany	2016-2017	Join the Healthy Boat	Subnational	both	5-6	5-6	124	129	
1219	Germany	2016-2019	Study of Health in Pomerania, second cohort (SHIP-TREND-1) 8-year follow-up	Subnational	both	28-90	28-90	1200	1273	15
1220	Germany	2017-2021	Gutenberg Health Study	Community	urban	45+	45+	4209	3927	
1221	Germany	2018-2019	Childhood Obesity Surveillance Initiative 5 - Bremen	Community	urban	7-9	7-9	1110	1111	
1222	Germany	2021-2023	Metabolism, Nutrition, and the Immune System Augsburg (MEIA)	Subnational	both	18-75	18-75	257	334	
1223	Germany	2023	Childhood Obesity Surveillance Initiative 6	Community	urban	7-9	7-9	586	605	
1224	Ghana	1987-1988	Ghana Living Standards Survey (GLSS)	National	both	5+	5+	5476	5798	
1225	Ghana	1988-1989	Ghana Living Standards Survey (GLSS)	National	both	5+	5+	5240	5537	
1226	Ghana	1993	DHS	National	both		20-49		1650	
1227	Ghana	1997	Amoah et al., Ethn Dis 13(2 Suppl 2):S97-101, 2003	Community	both	25+	25+	1857	2875	
1228	Ghana	1998	DHS	National	both		20-49		1979	
1229	Ghana	2001	Addo et al., Ethn Dis 16(4):894-99, 2006	Community	rural	15+	15+	89	206	
1230	Ghana	2001-2002	Cappuccio et al., Hypertension 43(5):1017-22, 2004	Community	both	35-84	35-84	194	338	
1231	Ghana	2002	Amoah et al., Ethn Dis 13(2 Suppl 2):S97-101, 2003	Community	both	25+	25+	1859	2947	
1232	Ghana	2003	DHS	National	both		15-49		4935	
1233	Ghana	2003	Women's Health Study of Accra (WHSA-I)	Community	urban		18+		1184	
1234	Ghana	2006	STEPS	Community	urban	25+	25+	841	1635	
1235	Ghana	2007-2008	WHO Study on global AGEing and adult health (SAGE)	National	both	50+	50+	2192	1987	
1236	Ghana	2008	DHS	National	both		15-49		4455	
1237	Ghana	2008-2010	Women's Health Study of Accra (WHSA-II)	Community	urban		18+		2677	
1238	Ghana	2012-2014	Research on Obesity and Diabetes among African Migrants (RODAM), control group	Subnational	rural	25+	25+	431	676	

	Country	Study years	Survey/Study name/Citation	Level of representative-ness	Rural, urban, or both	Age range as in NCD-RisC database		Sample size		Note
						Male	Female	Male	Female	
1239	Ghana	2012-2014	Research on Obesity and Diabetes among African Migrants (RODAM), control group	Subnational	urban	25+	25+	418	1034	
1240	Ghana	2014	DHS	National	both	15-59	15-49	4293	4393	
1241	Ghana	2016-2017	Ghana Living Standards Survey	National	both	5+	5+	18888	20977	
1242	Ghana	2017	Ghana Micronutrient Survey	National	both		15-49		1001	
1243	Ghana	2018	Intervention to reduce cardiovascular disease risk factors among secondary school students: Randomised controlled trial - Baseline	Subnational	both	14-19	14-19	411	437	
1244	Ghana	2018	Intervention to reduce cardiovascular disease risk factors among secondary school students: Randomised controlled trial - Post-intervention study	Subnational	both	14-19	14-19	199	218	
1245	Ghana	2022	Global School-based Student Health Survey (Sekondi-Takoradi)	Community	urban	13-16	13-16	1429	1700	
1246	Ghana	2022-2023	DHS	National	both	15-59	15-49	4282	12186	
1247	Ghana	2023	STEPS	National	both	18-69	18-69	2010	3240	
1248	Greece	1991	Seven Countries Study	Subnational	both	70-89		177		
1249	Greece	1997	Greece Physical Fitness Study	National	both	8	8	31619	29980	
1250	Greece	1997	The Didima Study	Community	rural	18+	18+	265	373	
1251	Greece	1997-2000	Dietary criteria of adolescents: The role of physical activity, anthropometrics, dietetic, psychological and other factors	Community	urban	12-18	12-18	494	505	
1252	Greece	1998	Greece Physical Fitness Study	National	both	8-10	8-10	33370	32057	
1253	Greece	1999	Greece Physical Fitness Study	National	both	8	8	30090	28570	
1254	Greece	2000	Greece Physical Fitness Study	National	both	8	8	32466	30109	
1255	Greece	2000-2001	Karalis et al., BMC Public Health 7:351, 2007	Community	rural	5+	5+	73	87	
1256	Greece	2001	Greece Physical Fitness Study	National	both	8	8	31504	29956	
1257	Greece	2001-2002	The ATTICA study	Subnational	urban	18+	18+	1484	1510	
1258	Greece	2003	Greece Physical Fitness Study	National	both	8	8	33575	31495	
1259	Greece	2003	National Epidemiological Survey	National	both	13-19	13-19	6675	7778	
1260	Greece	2004	Greece Physical Fitness Study	National	both	8	8	33239	31989	
1261	Greece	2004-2005	Arsakeion School Study	Community	urban	6-18	6-18	358	420	
1262	Greece	2005	Greece Physical Fitness Study	National	both	9	9	33206	31950	
1263	Greece	2005	Daphne	Community	rural	17-18	17-18	41	57	
1264	Greece	2005-2006	The Gene-Diet Attica Investigation on childhood obesity (GENDAI)	Subnational	urban	10-12	10-12	526	452	
1265	Greece	2005-2006	Prevalence of hypertension and association of dietary mineral intake with blood pressure in healthy school children from Northern Greece aged 7-15 years	Community	urban	7-15	7-15	308	297	
1266	Greece	2006	Greece Physical Fitness Study	National	both	9	9	33545	31944	
1267	Greece	2006	Samos	Community	both	5-13	5-13	55	65	
1268	Greece	2006	Paliouri Study	Community	rural	65-94	65-94	92	66	
1269	Greece	2006-2007	HELENA, Athens	Community	urban	12-17	12-17	158	162	
1270	Greece	2006-2007	HELENA, Heraklion	Community	urban	12-17	12-17	135	149	
1271	Greece	2007	Greece Physical Fitness Study	National	both	9	9	33501	31870	
1272	Greece	2007-2009	Healthy Growth Study	Subnational	both	9-13	9-13	1317	1300	
1273	Greece	2008	Greece Physical Fitness Study	National	rural	8	8	8411	8077	
1274	Greece	2008	Greece Physical Fitness Study	National	urban	8	8	28189	27247	
1275	Greece	2008-2009	Greek Childhood Obesity Study (GRECO)	National	both	10-12	10-12	2033	2160	
1276	Greece	2008-2010	TEENs of Attica: Genes and Environment (TEENAGE)	Subnational	urban	12-14	12-14	436	366	
1277	Greece	2010	EuropeaN Energy balance Research to prevent excessive weight Gain among Youth - The ENERGY-project	National	both	10-12	10-12	493	577	
1278	Greece	2010-2011	Childhood Obesity Surveillance Initiative 2	National	both	7-9	7-9	2581	2688	
1279	Greece	2010-2012	ADONUT	National	both	12-19	12-19	18668	18675	
1280	Greece	2013	Telemedicine screening adolescent metabolic syndrome in Greek schools	Community	urban	12-17	12-17	669	905	
1281	Greece	2013	Childhood Obesity Surveillance Initiative 3	National	both	7-10	7-10	3965	3908	
1282	Greece	2013-2014	Greek National Health and Nutrition Survey - HYDRIA	National	both	18+	18+	1798	2082	
1283	Greece	2013-2015	Hellenic National Nutrition and Health Survey (HNNHS)	Subnational	urban	5+	5+	476	757	
1284	Greece	2013-2016	National Survey of Morbidity and Risk Factors (EMENO)	National	both	18+	18+	2039	2726	
1285	Greece	2014-2015	Evaluation of a web-based dietary intervention among primary school children (NUTRI-WEB Children project)	Community	both	7-12	7-12	387	433	
1286	Greece	2014-2017	National Survey of Morbidity and Risk Factors, Children substudy (EMENO-Children)	National	both	5-18	5-18	187	164	
1287	Greece	2015	EYZHN National Action for Childrens Health	National	both	5-17	5-17	164793	156417	
1288	Greece	2015-2016	Childhood Obesity Surveillance Initiative 4	National	both	7-9	7-9	1907	1872	
1289	Greece	2016	SKG-Elderly	Community	urban	60+	60+	51	63	
1290	Greece	2016-2017	Erasmus plus KA2, Healthyland	Community	rural	5	5	13	9	
1291	Greece	2017-2018	Erasmus plus KA2, Healthyland	Community	rural	5-6	5-6	33	12	
1292	Greece	2018	Greece Physical Fitness Study	National	both	5-19	5-19	165298	156790	
1293	Greece	2018	STEPS-Thessaloniki	Community	both	60+	60+	353	141	
1294	Greece	2019	Childhood Obesity Surveillance Initiative 5	National	both	7-10	7-10	2013	2014	
1295	Greece	2023	Childhood Obesity Surveillance Initiative 6	National	both	7-10	7-10	1973	2033	
1296	Greenland	2005-2010	Population Health Survey in Greenland	National	both	18+	18+	1336	1714	
1297	Greenland	2016-2019	Population Health Survey in Greenland	National	both	15+	15+	1117	1308	
1298	Grenada	2011	STEPS	National	both	25-64	25-64	438	637	

	Country	Study years	Survey/Study name/Citation	Level of representative-ness	Rural, urban, or both	Age range as in NCD-RisC database		Sample size		Note
						Male	Female	Male	Female	
1299	Guatemala	1995	DHS	National	both		20-49		4547	
1300	Guatemala	1998-1999	DHS	National	both		20-49		2172	
1301	Guatemala	2000	Encuesta Nacional sobre Condiciones de Vida (ENCOVI)	National	both	5+	5+	13651	14610	
1302	Guatemala	2001-2002	CAMDI	Community	urban	20+	20+	293	638	
1303	Guatemala	2002	Reproducive Health Survey	National	both	15-59	15-49	2164	7374	
1304	Guatemala	2003-2005	The Institute of Nutrition of Central America and Panama Nutrition Supplementation Trial Cohort	Community	both	25-41	25-41	268	288	
1305	Guatemala	2008-2009	Encuesta Nacional de Salud Materno Infantil	National	both	15-59	15-49	6636	15271	
1306	Guatemala	2013	Sistema de vigilancia Epidemiológica de Salud y Nutrición (SIVESNU)	National	both		15-49		1545	
1307	Guatemala	2014-2015	DHS	National	both		15-49		24195	
1308	Guatemala	2015	Sistema de vigilancia Epidemiológica de Salud y Nutrición (SIVESNU)	National	both		15-49		1542	
1309	Guatemala	2015	STEPS	Subnational	urban	18+	18+	432	1444	
1310	Guatemala	2015-2017	Nutrition on early childhood and metabolomic and cardiometabolic profile on adulthood (META)	Community	both	37-55	37-55	213	311	
1311	Guatemala	2016	Sistema de vigilancia Epidemiológica de Salud y Nutrición (SIVESNU)	National	both		15-49		1560	
1312	Guatemala	2017-2018	Sistema de vigilancia Epidemiológica de Salud y Nutrición (SIVESNU)	National	both		15-49		1557	
1313	Guatemala	2018	Assessing the food environment inside and around rural public schools in three villages of El Progreso, Guatemala. A pilot study.	Community	rural	6-15	6-15	202	192	
1314	Guatemala	2018-2019	Sistema de vigilancia Epidemiológica de Salud y Nutrición (SIVESNU)	National	both		15-49		1588	
1315	Guatemala	2018-2019	Population-Based Survey of Chronic Kidney Disease in Guatemala	Community	rural	18+	18+	265	509	
1316	Guatemala	2019	Evaluación del estado nutricional de escolares de nivel preprimario y primario en el marco de la ley de alimentación escolar	National	both	5-14	5-14	9645	8979	
1317	Guatemala	2021	Evaluación del estado nutricional de escolares de nivel preprimario y primario en el marco de la ley de alimentación escolar	National	both	5-14	5-14	11919	11376	
1318	Guatemala	2022	Evaluacion del estado nutricional de escolares de nivel preprimario y primario en el marco de la ley de alimentacion escolar	National	both	5-19	5-19	8530	8599	
1319	Guinea	1999	DHS	National	both		20-49		2984	
1320	Guinea	2005	DHS	National	both		15-49		3574	
1321	Guinea	2009	STEPS	Subnational	both	15-64	15-64	1124	1232	
1322	Guinea	2012	DHS	National	both		15-49		4229	
1323	Guinea	2018	DHS	National	both		15-49		4905	
1324	Guinea Bissau	2010	Multiple Indicator Cluster Survey	National	both		15-49		7676	
1325	Guinea Bissau	2012	Deuxieme Enquete Nationale Sur L'etat Nutritionnel	National	both		15-49		4976	
1326	Guyana	2009	DHS	National	both	15-49	15-49	3412	4575	
1327	Guyana	2010	Global School-based Student Health Survey	National	both	13-17	13-17	987	1261	
1328	Guyana	2016	STEPS	National	both	18-69	18-69	1060	1571	
1329	Haiti	1994-1995	DHS	National	both		20-49		1788	
1330	Haiti	2000	DHS	National	both		15-49		9163	
1331	Haiti	2005-2006	DHS	National	both		15-49		5011	
1332	Haiti	2012	DHS	National	both		15-49		8993	
1333	Haiti	2015-2016	Haiti Health Study (Carrefour)	Community	urban	25-65	25-65	557	835	
1334	Haiti	2015-2016	Haiti Health Study (Thomonde)	Community	rural	25-65	25-65	257	420	
1335	Haiti	2016-2017	DHS	National	both		15-49		9049	
1336	Haiti	2019-2021	Haiti Cardiovascular Disease Cohort	Community	urban	18+	18+	1258	1709	
1337	Honduras	1996	Honduras National Micronutrient Survey	National	both		20-40		722	
1338	Honduras	2001	Encuesta Nacional de Epidemiologia y Salud Familiar	National	both		15-49		3629	
1339	Honduras	2003-2004	CAMDI	Community	urban	20+	20+	428	764	
1340	Honduras	2005-2006	DHS	National	both		15-49		18125	
1341	Honduras	2011-2012	DHS	National	both		15-49		21097	
1342	Honduras	2019	ENDESA/MICS	National	both		15-49		17951	
1343	Hungary	1982-1983	MONICA, Pecs	Community	urban	25-64	25-64	823	861	
1344	Hungary	1982-1984	MONICA, Budapest	Community	urban	25-64	25-64	774	737	
1345	Hungary	1985	INTERSALT	Community	rural	20-59	20-59	100	100	
1346	Hungary	1985-1988	First Hungarian Representative Nutrition Survey	National	both	15+	15+	3079	8916	
1347	Hungary	1987-1988	MONICA, Budapest	Community	urban	25-64	25-64	1413	1594	
1348	Hungary	1987-1988	MONICA, Pecs	Community	urban	35-64	35-64	1573	1510	
1349	Hungary	2003	The European Male Ageing Study	Community	both	40+		428		
1350	Hungary	2006-2007	HELENA	Community	urban	12-17	12-17	197	197	
1351	Hungary	2007-2010	Identification and prevention of Dietary- and lifestyle-induced health EFfects In Children and infantS (IDEFICS)	Community	urban	5-9	5-9	1128	1169	
1352	Hungary	2008	The European Male Ageing Study	Community	both	45+		349		
1353	Hungary	2009	Hungarian diet and nutritional status survey	National	both	18+	18+	463	666	
1354	Hungary	2010	Childhood Obesity Surveillance Initiative 2	National	both	7	7	553	682	
1355	Hungary	2010	EuropeaN Energy balance Research to prevent excessive weight Gain among Youth - The ENERGY-project	National	both	11-13	11-13	452	557	
1356	Hungary	2014	Hungarian diet and nutritional status survey	National	both	18+	18+	369	478	
1357	Hungary	2015-2016	Childhood Obesity Surveillance Initiative 4	National	both	6-8	6-8	2750	2572	
1358	Hungary	2016	Feel4Diabetes	Community	both	6-10	6-10	1447	1522	
1359	Hungary	2016	Hungarian National Student Fitness Test (NETFIT) - Primary	National	both	10-15	10-15	164897	158087	
1360	Hungary	2016	Hungarian National Student Fitness Test (NETFIT) - Secondary	National	both	14-19	14-19	134129	132326	

	Country	Study years	Survey/Study name/Citation	Level of representative-ness	Rural, urban, or both	Age range as in NCD-RisC database		Sample size		Note
						Male	Female	Male	Female	
1361	Hungary	2017	Hungarian National Student Fitness Test (NETFIT) - Primary	National	both	10-15	10-15	170978	163599	
1362	Hungary	2017	Hungarian National Student Fitness Test (NETFIT) - Secondary	National	both	14-19	14-19	136278	134646	
1363	Hungary	2018	Hungarian National Student Fitness Test (NETFIT) - Primary	National	both	10-15	10-15	169915	162664	
1364	Hungary	2018	Hungarian National Student Fitness Test (NETFIT) - Secondary	National	both	14-19	14-19	128717	127821	
1365	Hungary	2019	Childhood Obesity Surveillance Initiative 5	National	both	6-8	6-8	3173	2996	
1366	Hungary	2019	Hungarian National Student Fitness Test (NETFIT) - Primary	National	both	10-15	10-15	171701	165199	
1367	Hungary	2019	Hungarian National Student Fitness Test (NETFIT) - Secondary	National	both	14-19	14-19	132647	130182	
1368	Hungary	2019	Hungarian diet and nutritional status survey	National	both	18+	18+	419	493	
1369	Hungary	2022	Childhood Obesity Surveillance Initiative 6	National	both	6-8	6-8	2553	2471	
1370	Hungary	2022	Hungarian National Student Fitness Test (NETFIT) - Primary	National	both	10-15	10-15	137148	134347	
1371	Hungary	2022	Hungarian National Student Fitness Test (NETFIT) - Secondary	National	both	15-18	15-18	69966	78229	
1372	Hungary	2023	Hungarian National Student Fitness Test (NETFIT) - Primary	National	both	10-15	10-15	160543	154349	
1373	Hungary	2023	Hungarian National Student Fitness Test (NETFIT) - Secondary	National	both	15-18	15-18	126140	124802	
1374	Hungary	2024	Hungarian National Student Fitness Test (NETFIT) - Primary	National	both	10-15	10-15	159892	152855	
1375	Hungary	2024	Hungarian National Student Fitness Test (NETFIT) - Secondary	National	both	15-18	15-18	126948	125611	
1376	Iceland	1979-1981	The Reykjavik Study (Men)	Subnational	urban	45-74		3235		
1377	Iceland	1981-1984	The Reykjavik Study (Women)	Subnational	urban		46-75		3567	
1378	Iceland	1983	MONICA, Arnes County	Community	rural	25-64	25-64	388	450	
1379	Iceland	1983	MONICA, Reykjavik	Subnational	urban	25-64	25-64	434	461	
1380	Iceland	1983-1985	The Reykjavik Study for the young	Subnational	urban	29-45	29-45	823	895	
1381	Iceland	1985-1986	INTERSALT	Community	urban	20-59	20-59	100	100	
1382	Iceland	1985-1987	The Reykjavik Study (Men)	Subnational	urban	51-79		2584		
1383	Iceland	1987-1991	The Reykjavik Study (Women)	Subnational	urban		52-82		2993	
1384	Iceland	1988-1989	MONICA, Arnes County	Community	rural	25-64	25-64	385	435	
1385	Iceland	1988-1989	MONICA, Reykjavik	Subnational	urban	25-64	25-64	414	443	
1386	Iceland	1991-1994	The Reykjavik Study (Men)	Subnational	urban	70-86		797		
1387	Iceland	1993-1994	MONICA, Arnes County	Community	rural	25-64	25-64	422	484	
1388	Iceland	1993-1994	MONICA, Reykjavik	Subnational	urban	25-64	25-64	441	448	
1389	Iceland	1994-1996	The Reykjavik Study (Women)	Subnational	urban		69-88		1101	
1390	Iceland	2001-2003	The Reykjavik Study for the young	Subnational	urban	47-62	47-62	626	705	
1391	Iceland	2002-2006	AGES	Subnational	urban	66-96	66-96	2414	3282	
1392	Iceland	2005-2011	Risk Evaluation For INfarct Estimates (REFINE)	Subnational	urban	20-73	20-73	3402	3525	
1393	Iceland	2007-2011	AGESII	Subnational	urban	71-98	71-98	1374	1929	
1394	Iceland	2010-2012	Risk Evaluation For INfarct Estimates (REFINE) follow-up visit (REFINELO)	Subnational	urban	26-74	26-74	653	667	
1395	Iceland	2012-2013	Risk Evaluation For INfarct Estimates (REFINE) follow-up visit (REFLOCT)	Subnational	urban	55-73	55-73	516	561	
1396	India	1975-1979	National Nutrition Monitoring Bureau rural survey	National	rural	5+	5+	52704	37750	1
1397	India	1982-1983	Bengali School Children	Community	urban	7-21		808		
1398	India	1986	INTERSALT	Community	urban	20-59	20-59	100	99	
1399	India	1988-1989	Ramachandran et al., Diabetes Res Clin Pract 58(1):55-60, 2002	Community	urban	20-74	20-74	455	437	
1400	India	1990	National Nutrition Monitoring Bureau rural survey	National	rural	5+	5+	7607	9649	
1401	India	1991-1994	Prabhakaran et al., Chronic Illn 3(1):8-19, 2007	Community	rural	35-64	35-64	542	630	
1402	India	1991-1994	Prabhakaran et al., Chronic Illn 3(1):8-19, 2007	Community	urban	35-64	35-64	1388	1455	
1403	India	1991-1995	Reddy et al., Obes Rev 3(3):197-202, 2002	Community	rural	35-64	35-64	1070	1332	
1404	India	1991-1995	Reddy et al., Obes Rev 3(3):197-202, 2002	Community	urban	35-64	35-64	1456	1594	
1405	India	1991-1997	Mumbai Cohort Study	Community	urban	35+	35+	88658	59515	
1406	India	1992-1994	Jaipur Heart Watch 1	Community	urban	20-80	20-80	1385	782	
1407	India	1992-1994	Jaipur Heart Watch 1	Community	rural	20-80	20-80	1946	1147	
1408	India	1993-1994	Khongsdier, Eur J Clin Nutr 56(6):484-89, 2002	Community	both	18-59		575		
1409	India	1995	Shobana et al., Diabetes Res Clin Pract 42(3):181-86, 1998	Community	urban	20-74	20-74	1061	1093	
1410	India	1995-1996	Kusuma et al., Ann Hum Biol 29(5):502-12, 2002	Community	both	15-84	15-84	747	737	
1411	India	1995-1996	Epidemiology of blood pressure across cross-cultural populations of Visakhapatnam district, Andhra Pradesh, India	Community	rural	19-76	19-76	209	228	
1412	India	1995-1997	Aravind Comprehensive Eye Survey	Community	rural	40+	40+	2308	2830	
1413	India	1996-1997	National Nutrition Monitoring Bureau rural survey	National	rural	5+	5+	22155	27802	
1414	India	1996-1999	Chennai Urban Population Study	Community	urban	20+	20+	557	705	
1415	India	1997	Ramachandran et al., Diabetes Res Clin Pract 44(3):207[illegible]13, 1999	Community	rural	20-74	20-74	738	879	
1416	India	1998-1999	DHS	National	both		20-49		72536	
1417	India	1998-2001	Chennai Prospective Study	Community	urban	35+	35+	264848	235968	
1418	India	1998-2002	Vellore Birth Cohort	Subnational	both	25-31	25-31	1160	1050	
1419	India	1999-2001	Jaipur Heart Watch 2	Community	urban	20-75	20-75	534	569	
1420	India	1999-2002	Bengali School Children	Community	urban	7-21		1152		
1421	India	1999-2002	New Delhi Birth Cohort	Community	urban	26-33	26-33	886	638	
1422	India	2000	Ramachandran et al., Diabet Med 20(3):220-24, 2003	Subnational	urban	20-75	20-75	4640	5257	
1423	India	2000-2001	National Nutrition Monitoring Bureau rural survey	National	rural	5+	5+	18046	24844	

	Country	Study years	Survey/Study name/Citation	Level of representative-ness	Rural, urban, or both	Age range as in NCD-RisC database		Sample size		Note
						Male	Female	Male	Female	
1424	India	2001-2004	Chennai Urban Rural Epidemiology Study	Community	urban	20+	20+	1094	1254	
1425	India	2002-2003	Blood pressure epidemiology in tribal, rural and urban communities of Orissa with special reference to physical and social parameters	Community	rural	18-80	18-80	200	186	
1426	India	2002-2005	The Chennai Glaucoma Study	Subnational	urban	40+	40+	1577	1972	
1427	India	2003-2004	ICMR RF RHD Registry, Jai Vigyan Mission Mode, Kochi	Subnational	both	5-16	5-16	11327	13515	
1428	India	2003-2004	Jaipur Heart Watch 3	Community	urban	20-75	20-75	211	228	
1429	India	2003-2005	STEPS, Ballabgarh	Subnational	rural	15-69	15-69	1360	1468	
1430	India	2003-2005	STEPS, Ballabgarh	Subnational	urban	15-69	15-69	1263	1294	
1431	India	2003-2005	STEPS, Chennai	Subnational	rural	15-69	15-69	1372	1338	
1432	India	2003-2005	STEPS, Chennai	Subnational	urban	15-69	15-69	1282	1282	
1433	India	2003-2005	STEPS, Delhi	Subnational	urban	15-69	15-69	1250	1265	
1434	India	2003-2005	STEPS, Dibrugarh	Subnational	rural	15-69	15-69	1460	1410	
1435	India	2003-2005	STEPS, Dibrugarh	Subnational	urban	15-69	15-69	1243	1254	
1436	India	2003-2005	STEPS, Nagpur	Subnational	rural	15-69	15-69	1252	1256	
1437	India	2003-2005	STEPS, Nagpur	Subnational	urban	15-69	15-69	1252	1261	
1438	India	2003-2005	STEPS, Trivandrum	Subnational	rural	15-69	15-69	1199	1324	
1439	India	2003-2005	STEPS, Trivandrum	Subnational	urban	15-69	15-69	1250	1252	
1440	India	2004-2005	India Human Development Survey	National	both	8-11	8-11	6959	6406	17
1441	India	2005-2006	DHS	National	both	15-54	15-49	71463	115828	
1442	India	2005-2006	Risk factor profile for chronic non-communicable diseases: Results of a community-based study in Kerala, India	Community	both	15-64	15-64	2795	2926	
1443	India	2005-2006	National Nutrition Monitoring Bureau rural survey	National	rural	5+	5+	20448	25272	
1444	India	2005-2006	ICMR RF RHD Registry, Jai Vigyan Mission Mode, Kochi	Subnational	both	5-16	5-16	9754	10509	
1445	India	2005-2007	Prevalence of cardiovascular risk factors in rural Tamil Nadu	Community	rural	25-64	25-64	4927	5573	
1446	India	2005-2011	Bengali School Children	Community	urban	7-21	7-21	847	2180	
1447	India	2006	Ramachandran et al., Diabetes Care 31(5):893-98, 2008	Community	both	20+	20+	3321	3745	
1448	India	2006-2007	National Institute of Nutiriton School Survey	Subnational	both	10-16	10-16	4049	3650	
1449	India	2006-2007	Jaipur Heart Watch 4	Community	urban	20-75	20-75	533	558	
1450	India	2006-2007	Kusuma et al., Asia Pac J Public Health 21(4):497-507, 2009	Community	urban	15-74	15-74	182	192	
1451	India	2006-2008	Central India Eye and Medical Study	Community	rural	30+	30+	2190	2518	
1452	India	2006-2009	New Delhi Birth Cohort	Community	urban	33-38	33-38	650	445	
1453	India	2007-2008	Integrated Disease Surveillance Project Non-communicable Disease Risk Factors Survey, Andhra	Subnational	both	15-64	15-64	2674	3390	
1454	India	2007-2008	Integrated Disease Surveillance Project Non-communicable Disease Risk Factors Survey, Kerala	Subnational	both	15-64	15-64	1672	2403	
1455	India	2007-2008	Integrated Disease Surveillance Project Non-communicable Disease Risk Factors Survey, Madhya	Subnational	both	15-64	15-64	2797	2862	
1456	India	2007-2008	Integrated Disease Surveillance Project Non-communicable Disease Risk Factors Survey, Maharashtra	Subnational	both	15-64	15-64	3025	2921	
1457	India	2007-2008	Integrated Disease Surveillance Project Non-communicable Disease Risk Factors Survey, Mizoram	Subnational	both	15-64	15-64	2232	2108	
1458	India	2007-2008	Integrated Disease Surveillance Project Non-communicable Disease Risk Factors Survey, Tamil	Subnational	both	15-64	15-64	2039	2928	
1459	India	2007-2008	Integrated Disease Surveillance Project Non-communicable Disease Risk Factors Survey, Uttrakhand	Subnational	both	15-64	15-64	2094	3110	
1460	India	2007-2008	Urban population in Hyderabad	Community	urban	20-60	20-60	1519	1560	
1461	India	2007-2008	WHO Study on global AGEing and adult health (SAGE)	National	both	50+	50+	3213	3147	
1462	India	2007-2009	Prevalence of NCD risk factor in people above 15 year in rural area Nagpur using WHO STEP approach	Community	rural	15+	15+	1984	1828	
1463	India	2008-2010	ICMR-India Diabetes (INDIAB) Study, Phase I	Subnational	both	20+	20+	6953	6850	
1464	India	2008-2012	ORANGE Study, community component	Community	urban	6-19	6-19	774	739	
1465	India	2008-2012	ORANGE Study, school component	Community	urban	6-16	6-16	9288	7231	
1466	India	2009-2010	Baseline Survey for the assessment of prevalence of risk factors of NCDs in Gandhinagar District	Community	rural	15-64	15-64	875	774	
1467	India	2009-2010	Baseline Survey for the assessment of prevalence of risk factors of NCDs in Gandhinagar District	Community	urban	15-64	15-64	895	890	
1468	India	2009-2010	Jaipur Heart Watch 5	Community	urban	20-75	20-75	425	275	
1469	India	2010	Kerala 2010 follow-up	Community	rural	21-70	21-70	214	237	
1470	India	2010-2011	Longitudinal Aging Study in India	Subnational	both	45+	45+	630	678	
1471	India	2010-2012	Centre for cArdiometabolic Risk Reduction in South-Asia (CARRS) - Surveillance Study	Community	urban	20+	20+	4320	5138	18
1472	India	2011-2012	Body Mass Index, Social Conditions and Environmental Effect on High Blood Pressure among the Adolescent School Children	National	both	12-16	12-16	1096	1161	
1473	India	2011-2012	India Human Development Survey	National	both	8-11	8-11	6285	5758	19
1474	India	2011-2012	National Nutrition Monitoring Bureau rural survey	National	rural	5+	5+	34447	43063	
1475	India	2011-2013	International Study of Childhood Obesity, Lifestyle and the Environment (ISCOLE)	Community	urban	9-11	9-11	292	328	
1476	India	2012-2013	Health Survey in Anand School Children	Community	both	5-13	5-13	1628	959	
1477	India	2012-2013	District Level Household and Facility Survey (DLHS) 4	National	both	5+	5+	538808	602926	
1478	India	2012-2013	Processed and non-processed foods - Rural sample	National	rural	5+	5+	4957	5350	
1479	India	2012-2013	ICMR-India Diabetes (INDIAB) Study, Phase II	Subnational	both	20+	20+	8165	10738	
1480	India	2012-2014	Jaipur Heart Watch 6	Community	urban	20-75	20-75	1013	722	
1481	India	2012-2015	ICMR-India Diabetes (INDIAB) Study, North East Phase	Subnational	both	20+	20+	14071	16456	
1482	India	2013-2014	Vellore Birth Cohort	Subnational	both	39-44	39-44	580	499	
1483	India	2014	Annual Health Survey: Clinical, Anthropometric and Bio-chemical	National	both	5+	5+	658441	680611	
1484	India	2014-2015	Control of Hypertension In Rural India (CHIRI) - Rishi Valley	Community	rural	18+	18+	2591	3518	
1485	India	2014-2015	Control of Hypertension In Rural India (CHIRI) - Trivandrum	Community	rural	18+	18+	1848	1898	

	Country	Study years	Survey/Study name/Citation	Level of representative-ness	Rural, urban, or both	Age range as in NCD-RisC database		Sample size		Note
						Male	Female	Male	Female	
1486	India	2014-2015	Control of Hypertension In Rural India (CHIRI) - West Godavari	Community	rural	18+	18+	2230	2233	
1487	India	2015-2016	DHS	National	both	15-54	15-49	108751	655681	
1488	India	2015-2016	Diet and nutritional status of urban population and prevalence of hypertension	National	urban	5+	5+	68893	81999	
1489	India	2015-2018	ORANGE Study, 10-year follow-up	Community	urban	10-30	10-30	429	408	
1490	India	2016-2017	Secular TRends in DiabEtes in India (STRiDE-I) -Change in Prevalence in Ten Years among Urban and Rural Populations in Tamil Nadu	Community	both	20+	20+	4527	5321	
1491	India	2016-2019	Vellore Birth Cohort	Subnational	both	43-48	43-48	843	758	
1492	India	2017-2018	National Noncommunicable Disease Monitoring Survey (NNMS)	National	both	15-69	15-69	6223	5657	
1493	India	2017-2018	ICMR-India Diabetes (INDIAB) Study, Phase III	Subnational	both	20+	20+	7146	7550	
1494	India	2017-2019	Longitudinal Aging Study in India	National	both	45+	45+	27791	32117	
1495	India	2018-2019	ICMR-India Diabetes (INDIAB) Study, Phase IV	Subnational	both	20+	20+	8850	10154	
1496	India	2018-2021	Telemedicine PRoject for screENing Diabetes and its complications in rural Tamil Nadu (TREND) study	Community	rural	18+	18+	6283	8743	
1497	India	2019-2020	Prevalence, awareness, treatment and control of hypertension among adults aged 30 years and above in Barmer district, Rajasthan, India	Community	rural	30+	30+	153	146	
1498	India	2019-2020	ICMR-India Diabetes (INDIAB) Study, Phase V	Subnational	both	20+	20+	6597	6656	
1499	India	2019-2021	DHS	National	both	15-54	15-49	95959	672286	
1500	India	2021	STEPS, Mumbai	Community	urban	18-69	18-69	2551	2530	
1501	India	2022	Global School-based Student Health Survey (Jaipur)	Community	urban	12-15	12-15	1524	1355	
1502	Indonesia	1983-1987	Strickland et al., Eur J Clin Nutr 48 Suppl 3: S98-108; discussion S-9, 1994	Community	both	18+	18+	447	564	
1503	Indonesia	1993-1994	Indonesian Family Life Surveys	National	both	5+	5+	8211	9480	
1504	Indonesia	1997-1998	Indonesian Family Life Surveys	National	both	5+	5+	12154	13897	
1505	Indonesia	2000-2001	Indonesian Family Life Surveys	National	both	5+	5+	15442	16225	
1506	Indonesia	2001	Ng et al., Bull World Health Organ 84(4):305-13, 2006	Community	both	15-74	15-74	1261	1234	
1507	Indonesia	2001	STEPS/SURKESNAS	National	both	15-64	15-64	4100	4775	
1508	Indonesia	2003	A genetic-ecological study of the risk factors for lifestyle-related diseases in Oceanian populations, Study A	Community	rural	18-79	18-79	99	103	
1509	Indonesia	2003	A genetic-ecological study of the risk factors for lifestyle-related diseases in Oceanian populations, Study B	Community	rural	18-79	18-79	100	140	
1510	Indonesia	2006	Jakarta Non Communicable Disease Risk Factor Surveillance	Community	urban	25-64	25-64	641	950	
1511	Indonesia	2007	Indonesian Basic Health Survey (RISKESDAS) 2007	National	both	5+	5+	426975	437608	
1512	Indonesia	2007-2008	Indonesian Family Life Surveys	National	both	5+	5+	17869	19157	
1513	Indonesia	2011	SEANUTS	National	both	5-12	5-12	1363	1380	
1514	Indonesia	2013	Indonesian Basic Health Survey (RISKESDAS) 2013	National	both	5+	5+	454768	466398	
1515	Indonesia	2014-2015	Indonesian Family Life Surveys	National	both	5+	5+	20328	21540	
1516	Indonesia	2015	Global School-based Student Health Survey	National	both	13-17	13-17	3845	4688	
1517	Indonesia	2018	Indonesian Basic Health Survey (RISKESDAS) 2018	National	both	5+	5+	444327	459143	
1518	Indonesia	2023	Global School-based Student Health Survey	National	both	12-17	12-17	4572	4857	
1519	Iran	1990-1991	National Health Survey I	National	both	5-18	5-18	8883	9038	
1520	Iran	1997-1998	Khadivzadeh, East Mediterr Health J 8(4-5):612-18, 2002	Community	urban		15-49		1510	
1521	Iran	1999-2000	National Health Survey II	National	both	5+	5+	23727	26636	
1522	Iran	1999-2001	Tehran Lipid and Glucose Study	Community	urban	5+	5+	6337	7991	
1523	Iran	2000	ASADABADI Study	Community	urban	18+	18+	130	166	
1524	Iran	2001	Isfahan Healthy Heart Programme (IHHP) Students, Arak	Community	rural	11-18	11-18	144	192	
1525	Iran	2001	Isfahan Healthy Heart Programme (IHHP) Students, Arak	Community	urban	11-18	11-18	323	303	
1526	Iran	2001	Isfahan Healthy Heart Programme (IHHP) Students, Isfahan	Community	rural	11-18	11-18	89	118	
1527	Iran	2001	Isfahan Healthy Heart Programme (IHHP) Students, Isfahan	Community	urban	11-18	11-18	242	225	
1528	Iran	2001	Isfahan Healthy Heart Programme (IHHP) Students, Najaf Abad	Community	rural	11-18	11-18	61	74	
1529	Iran	2001	Isfahan Healthy Heart Programme (IHHP) Students, Najaf Abad	Community	urban	11-18	11-18	62	72	
1530	Iran	2001	Isfahan Healthy Heart Programme (IHHP), Arak	Community	rural	19+	19+	1023	1080	
1531	Iran	2001	Isfahan Healthy Heart Programme (IHHP), Arak	Community	urban	19+	19+	2084	2124	
1532	Iran	2001	Isfahan Healthy Heart Programme (IHHP), Isfahan	Community	rural	19+	19+	232	233	
1533	Iran	2001	Isfahan Healthy Heart Programme (IHHP), Isfahan	Community	urban	19+	19+	1760	1912	
1534	Iran	2001	Isfahan Healthy Heart Programme (IHHP), Najaf Abad	Community	rural	19+	19+	405	416	
1535	Iran	2001	Isfahan Healthy Heart Programme (IHHP), Najaf Abad	Community	urban	19+	19+	573	571	
1536	Iran	2001-2005	Tehran Lipid and Glucose Study	Community	urban	5+	5+	5080	6185	
1537	Iran	2003-2004	Childhood and Adolescence Surveillance and Prevention of Adult Noncommunicable Disease (CASPIAN)	National	both	6-18	6-18	10791	10170	
1538	Iran	2003-2004	The Persian Gulf Healthy Heart Study	Subnational	urban	25-75	25-75	1736	1973	
1539	Iran	2004	Hajian-Tilaki et al., Obes Rev 8(1):3-10, 2007	Community	urban	20-70	20-70	1800	1800	
1540	Iran	2004-2008	Golestan Cohort Study Main Phase	Subnational	rural	40-75	40-75	17298	22708	
1541	Iran	2004-2008	Golestan Cohort Study Main Phase	Community	urban	40-75	40-75	3931	6100	
1542	Iran	2005	STEPS	National	both	15-64	15-64	40722	39748	
1543	Iran	2005	Dastgiri et al., Public Health Nutr 9(8): 996-1000, 2006	Subnational	urban	18-70	18-70	116	151	
1544	Iran	2005-2006	Rashidy-Pour, Obes Rev (1):2-6, 2009	Subnational	both	30-70	30-70	1695	2104	
1545	Iran	2005-2008	Tehran Lipid and Glucose Study	Community	urban	5+	5+	5332	6665	
1546	Iran	2006	STEPS	National	both	16-65	16-65	14885	14617	

	Country	Study years	Survey/Study name/Citation	Level of representative-ness	Rural, urban, or both	Age range as in NCD-RisC database		Sample size		Note
						Male	Female	Male	Female	
1547	Iran	2007	Isfahan Healthy Heart Programme (IHHP) Students, Arak	Community	rural	11-18	11-18	177	164	
1548	Iran	2007	Isfahan Healthy Heart Programme (IHHP) Students, Arak	Community	urban	11-18	11-18	327	341	
1549	Iran	2007	Isfahan Healthy Heart Programme (IHHP) Students, Isfahan	Community	rural	11-18	11-18	16	19	
1550	Iran	2007	Isfahan Healthy Heart Programme (IHHP) Students, Isfahan	Community	urban	11-18	11-18	396	335	
1551	Iran	2007	Isfahan Healthy Heart Programme (IHHP) Students, Najaf Abad	Community	rural	11-18	11-18	38	39	
1552	Iran	2007	Isfahan Healthy Heart Programme (IHHP) Students, Najaf Abad	Community	urban	11-18	11-18	44	67	
1553	Iran	2007	STEPS - National	National	both	15-64	15-64	2372	2312	
1554	Iran	2007	STEPS - Provincial	National	both	15-64	15-64	14867	14550	
1555	Iran	2007	Isfahan Healthy Heart Programme (IHHP), Arak	Community	rural	19+	19+	1028	1024	
1556	Iran	2007	Isfahan Healthy Heart Programme (IHHP), Arak	Community	urban	19+	19+	1424	1359	
1557	Iran	2007	Isfahan Healthy Heart Programme (IHHP), Isfahan	Community	rural	19+	19+	155	151	
1558	Iran	2007	Isfahan Healthy Heart Programme (IHHP), Isfahan	Community	urban	19+	19+	1309	1301	
1559	Iran	2007	Isfahan Healthy Heart Programme (IHHP), Najaf Abad	Community	rural	19+	19+	253	253	
1560	Iran	2007	Isfahan Healthy Heart Programme (IHHP), Najaf Abad	Community	urban	19+	19+	494	542	
1561	Iran	2008	STEPS	National	both	15-64	15-64	14757	14353	
1562	Iran	2008-2011	Tehran Lipid and Glucose Study	Community	urban	5+	5+	5532	6791	
1563	Iran	2009	STEPS	National	both	15-64	15-64	14834	14495	
1564	Iran	2009-2010	Childhood and Adolescence Surveillance and Prevention of Adult Noncommunicable Disease (CASPIAN)	National	both	10-18	10-18	2799	2814	
1565	Iran	2009-2010	The Persian Gulf Healthy Heart Study	Subnational	urban	31-79	31-79	834	1016	
1566	Iran	2010-2011	The Yazd Eye Study	Subnational	both	40-80	40-80	876	1012	
1567	Iran	2010-2012	Golestan Cohort Study Second Phase	Subnational	rural	43-82	43-82	4325	4919	
1568	Iran	2010-2012	Golestan Cohort Study Second Phase	Community	urban	43-82	43-82	1091	1061	
1569	Iran	2011	STEPS	National	both	6-69	6-69	4903	6548	
1570	Iran	2011-2012	Amol county study	Community	rural	10+	10+	1862	1098	
1571	Iran	2011-2012	Amol county study	Community	urban	10+	10+	1624	1548	
1572	Iran	2011-2012	Childhood and Adolescence Surveillance and Prevention of Adult Noncommunicable Disease (CASPIAN)	National	both	6-18	6-18	6649	6443	
1573	Iran	2011-2015	Tehran Lipid and Glucose Study	Community	urban	5+	5+	5339	6465	
1574	Iran	2012	National Integrated Micronutrient Survey (NIMS) 2012	National	both	6-60	6-60	10526	11087	
1575	Iran	2012-2013	Tehran City	Community	urban	10-90	10-90	419	537	
1576	Iran	2012-2013	Zahedan City	Community	urban	10-90	10-90	1377	1205	
1577	Iran	2012-2014	Pars Cohort Study	Community	rural	40-90	40-90	4272	4987	
1578	Iran	2013-2014	Isfahan Salt Study (ISS)	Community	urban	6-18	6-18	400	383	
1579	Iran	2013-2014	Bushehr Elderly Health Program (BEH)	Community	urban	60+	60+	1437	1514	
1580	Iran	2013-2014	Gilan Eye Study	Subnational	both	50+	50+	1059	1439	
1581	Iran	2014-2015	Childhood and Adolescence Surveillance and Prevention of Adult Noncommunicable Disease (CASPIAN)	National	both	7-18	7-18	7164	6966	
1582	Iran	2014-2016	The PERSIAN Fasa Cohort Study	Community	rural	35-70	35-70	3742	4578	
1583	Iran	2014-2016	The PERSIAN Fasa Cohort Study	Community	urban	35-70	35-70	721	806	
1584	Iran	2014-2016	The PERSIAN Guilan Cohort Study	Community	rural	35-70	35-70	2645	3261	
1585	Iran	2014-2016	The PERSIAN Guilan Cohort Study	Community	urban	35-70	35-70	2236	2349	
1586	Iran	2014-2016	The PERSIAN Kermanshah Cohort Study	Community	rural	35-70	35-70	1806	2194	
1587	Iran	2014-2016	The PERSIAN Kermanshah Cohort Study	Community	urban	35-70	35-70	2940	2973	
1588	Iran	2014-2016	The PERSIAN Kharameh Cohort Study	Community	rural	35-70	35-70	2913	3860	
1589	Iran	2014-2016	The PERSIAN Kharameh Cohort Study	Community	urban	35-70	35-70	1794	2000	
1590	Iran	2014-2016	The PERSIAN Tabriz Cohort Study	Community	rural	35-70	35-70	1974	2548	
1591	Iran	2014-2016	The PERSIAN Tabriz Cohort Study	Community	urban	35-70	35-70	4670	5592	
1592	Iran	2014-2016	Yazd Health Study	Subnational	both	20-70	20-70	4695	4683	
1593	Iran	2015	Iranian School Measurement Database	National	both	6-18	6-18	911584	912687	
1594	Iran	2015-2017	The PERSIAN Mazandaran Cohort Study	Community	rural	35-70	35-70	936	1608	
1595	Iran	2015-2017	The PERSIAN Mazandaran Cohort Study	Community	urban	35-70	35-70	3179	4421	
1596	Iran	2015-2017	The PERSIAN Rafsanjan Cohort Study	Community	rural	35-70	35-70	1560	1030	
1597	Iran	2015-2017	The PERSIAN Rafsanjan Cohort Study	Community	urban	35-70	35-70	3591	4270	
1598	Iran	2015-2017	The PERSIAN Yazd Cohort Study	Community	urban	30-70	30-70	4947	4818	
1599	Iran	2015-2018	Tehran Lipid and Glucose Study	Community	urban	5+	5+	4947	5917	
1600	Iran	2015-2018	The PERSIAN Zahedan Cohort Study	Community	urban	35-70	35-70	3890	6013	
1601	Iran	2016	STEPS	National	both	18+	18+	14080	15036	
1602	Iran	2016-2017	Iranian Children and Adolescents Psychiatric Disorders (IRCAP) Survey	National	both	6-18	6-18	13049	13476	
1603	Iran	2016-2018	The PERSIAN Ahvaz Cohort Study	Community	rural	35-70	35-70	1475	2282	
1604	Iran	2016-2018	The PERSIAN Ahvaz Cohort Study	Community	urban	35-70	35-70	2507	3551	
1605	Iran	2016-2018	The PERSIAN BandarKong Cohort Study	Community	rural	35-70	35-70	232	366	
1606	Iran	2016-2018	The PERSIAN BandarKong Cohort Study	Community	urban	35-70	35-70	1470	1893	
1607	Iran	2016-2018	The PERSIAN Urmia Cohort Study	Community	rural	35-70	35-70	1750	2323	
1608	Iran	2016-2018	The PERSIAN Urmia Cohort Study	Community	urban	35-70	35-70	411	473	
1609	Iran	2016-2019	The Khuzestan comprehensive health study: A platform for NCDs, blood borne and mental diseases research	Subnational	both	20-65	20-65	10846	19427	

	Country	Study years	Survey/Study name/Citation	Level of representative-ness	Rural, urban, or both	Age range as in NCD-RisC database		Sample size		Note
						Male	Female	Male	Female	
1610	Iran	2016-2019	The PERSIAN Shahrekord Cohort Study	Community	rural	35-70	35-70	1136	1686	
1611	Iran	2016-2019	The PERSIAN Shahrekord Cohort Study	Community	urban	35-70	35-70	3450	3474	
1612	Iran	2016-2020	The PERSIAN Ardabil Cohort Study	Community	urban	35-70	35-70	9501	11200	
1613	Iran	2017-2018	The PERSIAN Kavar Cohort Study	Community	urban	35-70	35-70	2417	2539	
1614	Iran	2017-2018	PERSIAN Elderly Component-Iranian Longitudinal Study on Ageing	Subnational	urban	50-95	50-95	3357	3839	
1615	Iran	2017-2019	The PERSIAN Dena (Yasouj) Cohort Study	Community	rural	35-70	35-70	524	809	
1616	Iran	2017-2019	The PERSIAN Dena (Yasouj) Cohort Study	Community	urban	35-70	35-70	918	1083	
1617	Iran	2017-2019	The PERSIAN Sabzevar Cohort Study	Community	urban	35-70	35-70	1874	2320	
1618	Iran	2018-2019	Prevalence of risk factors for cardiovascular disease among a rural population in eastern Iran	Community	rural	18-69	18-69	148	146	
1619	Iran	2018-2019	The PERSIAN Dehgolan (Kordistan) Cohort Study	Community	urban	35-70	35-70	1741	2197	
1620	Iran	2018-2020	Bushehr Elderly Health program Phase II	Community	urban	50-95	50-95	848	1129	
1621	Iran	2018-2023	Tehran Lipid and Glucose Study	Community	urban	5+	5+	4636	5587	
1622	Iran	2020-2021	STEPS	National	both	18+	18+	12402	15333	
1623	Iraq	2006	STEPS	National	both	25-64	25-64	2251	2252	
1624	Iraq	2013-2014	Qadir et al., Malays J Med Health Sci, 10(2):27-38, 2014	Community	urban	13-17	13-17	832	748	
1625	Iraq	2015	STEPS	National	both	18+	18+	1589	2312	
1626	Ireland	1997-1999	North/South Ireland Food Consumption Survey	National	both	18-64	18-64	613	698	
1627	Ireland	1998	Survey of Lifestyle, Attitudes and Nutritional in Ireland 1998	National	both	18+	18+	123	296	
1628	Ireland	2002	Survey of Lifestyle, Attitudes and Nutritional in Ireland 2002	National	both	18+	18+	164	216	
1629	Ireland	2003-2004	National Children's Food Survey	National	both	5-12	5-12	293	301	
1630	Ireland	2005-2006	National Teens Food Survey	National	both	13-17	13-17	224	216	
1631	Ireland	2006-2007	Survey of Lifestyle, Attitudes and Nutritional in Ireland 2006-2007	National	both	18+	18+	945	1225	
1632	Ireland	2007-2008	Growing Up in Ireland - Child Cohort	National	both	9	9	3899	4107	20
1633	Ireland	2008	Childhood Obesity Surveillance Initiative 1	National	both	7	7	1098	1285	
1634	Ireland	2008-2010	National Adult Nutrition Survey	National	both	18+	18+	658	696	
1635	Ireland	2009-2011	The Irish Longitudinal Study on Ageing	National	both	50+	50+	2693	3170	
1636	Ireland	2010	Childhood Obesity Surveillance Initiative 2	National	both	6-9	6-9	1452	1533	
1637	Ireland	2010	Murtagh et al., Pediatr Exerc Sci 25(2):300-7, 2013	Community	rural	7-12	7-12	20	11	
1638	Ireland	2010	Murtagh et al., Pediatr Exerc Sci 25(2):300-7, 2013	Community	rural	7-12	7-12	19	19	
1639	Ireland	2010	Murtagh et al., Pediatr Exerc Sci 25(2):300-7, 2013	Community	rural	7-12	7-12	14	12	
1640	Ireland	2010	Murtagh et al., Pediatr Exerc Sci 25(2):300-7, 2013	Community	rural	7-12	7-12	16	21	
1641	Ireland	2011-2012	Growing Up in Ireland - Child Cohort	National	both	13	13	3537	3661	20
1642	Ireland	2012-2013	Childhood Obesity Surveillance Initiative 3	National	both	6-9	6-9	1087	1054	
1643	Ireland	2013	Growing Up in Ireland - Infant Cohort	National	both	5	5	4498	4382	20
1644	Ireland	2013-2016	Project Spraoi	Community	both	5-11	5-11	474	429	
1645	Ireland	2014-2015	The Irish Longitudinal Study on Ageing	National	both	54+	54+	2249	2618	
1646	Ireland	2015	Active Classrooms Study	Community	both	8-11	8-11	124	120	
1647	Ireland	2015-2016	Childhood Obesity Surveillance Initiative 4	National	both	6-10	6-10	1441	1679	
1648	Ireland	2015-2016	Growing Up in Ireland - Child Cohort	National	both	18	18	551	583	20
1649	Ireland	2016	Growing Up in Ireland - Infant Cohort	National	both	7-8	7-8	2016	1921	20
1650	Ireland	2017-2018	Growing Up in Ireland - Infant Cohort	National	both	9	9	3943	3892	20
1651	Ireland	2017-2018	National Children's Food Survey II	National	both	5-12	5-12	298	298	
1652	Ireland	2018-2019	Childhood Obesity Surveillance Initiative 5	National	both	6-12	6-12	2793	2768	
1653	Ireland	2018-2019	Growing Up in Ireland - Child Cohort	National	both	20	20	2202	2352	20
1654	Ireland	2019-2020	National Teens' Food Survey II	National	both	13-18	13-18	211	214	
1655	Ireland	2022-2023	Childhood Obesity Surveillance Initiative 6	National	both	6-8	6-8	1228	1347	
1656	Israel	1975-1979	Israeli Conscripts	National	both	16-19	16-19	125532	73424	1
1657	Israel	1980-1984	Israeli Conscripts	National	both	16-19	16-19	133146	85931	
1658	Israel	1985-1986	MONICA, Tel Aviv	Community	urban	25-64	25-64	653	685	
1659	Israel	1985-1989	Israeli Conscripts	National	both	16-19	16-19	148878	105833	
1660	Israel	1990-1991	The Jerusalem Longitudinal Cohort Study	Community	urban	69-70	69-70	245	199	
1661	Israel	1990-1994	Israeli Conscripts	National	both	16-19	16-19	191052	139555	
1662	Israel	1995-1999	Israeli Conscripts	National	both	16-19	16-19	202608	146585	
1663	Israel	1997-1998	The Jerusalem Longitudinal Cohort Study	Community	urban	76-77	76-77	422	429	
1664	Israel	1999-2001	Mabat First Israeli National Health and Nutrition Survey	National	both	25-64	25-64	1371	1411	
1665	Israel	1999-2005	The Israel Glucose Intolerance, Obesity and Hypertension Study (GOH)	National	urban	58-93	58-93	511	527	
1666	Israel	2000-2004	Israeli Conscripts	National	both	16-19	16-19	202713	152674	
1667	Israel	2002-2008	The Hadera District Study (HDS)	Subnational	urban	25-74	25-74	380	357	
1668	Israel	2003-2004	Mabat Youth First Israeli National Health and Nutrition Survey in 7th-12th grade students	National	both	12-18	12-18	2541	3055	
1669	Israel	2005-2006	The Jerusalem Longitudinal Cohort Study	Community	urban	83-85	83-85	490	584	
1670	Israel	2005-2006	Mabat Zahav First National Health and Nutrition Survey in ages 65 and over	National	urban	65+	65+	588	735	
1671	Israel	2005-2009	Israeli Conscripts	National	both	16-19	16-19	192835	146308	
1672	Israel	2010-2011	The Jerusalem Longitudinal Cohort Study	Community	urban	89-92	89-92	185	201	

	Country	Study years	Survey/Study name/Citation	Level of representative-ness	Rural, urban, or both	Age range as in NCD-RisC database		Sample size		Note
						Male	Female	Male	Female	
1673	Israel	2010-2014	Israeli Conscripts	National	both	16-19	16-19	213094	148908	
1674	Israel	2013-2015	The Hadera District Study (HDS) follow-up	Subnational	urban	33-87	33-87	377	345	
1675	Israel	2014-2015	Mabat Zahav Second National Health and Nutrition Survey ages in 65 and over	National	both	65+	65+	307	318	
1676	Israel	2014-2016	Mabat Second Israeli National Health and Nutrition Survey	National	both	18-64	18-64	1061	1073	
1677	Israel	2015-2016	Mabat Youth Second Israeli National Health and Nutrition Survey in 7th-12th grade students	National	both	12-18	12-18	1797	2094	
1678	Israel	2015-2016	Rav Mabat Kids - First National Health and Nutrition Survey in 2-11 year olds	National	rural	2-11	2-11	73	88	
1679	Israel	2015-2016	Rav Mabat Kids - First National Health and Nutrition Survey in 2-11 year olds	National	urban	2-11	2-11	515	463	
1680	Israel	2015-2016	The Jerusalem Longitudinal Cohort Study	Community	urban	94-96	94-96	50	65	
1681	Israel	2015-2019	Israeli Conscripts	National	both	16-19	16-19	199488	152571	
1682	Israel	2018-2019	Childhood Obesity Surveillance Initiative 5	National	both	6-7	6-7	73724	73561	
1683	Israel	2022-2023	Childhood Obesity Surveillance Initiative 6	National	both	6-7	6-7	75079	73985	
1684	Italy	1980-1982	Po river delta epidemiological study - first survey	Community	rural	8-64	8-64	1573	1710	
1685	Italy	1982-1987	MONICA, Latina	Community	both	24-66	24-66	852	868	
1686	Italy	1983-1984	Malattie cardiovascolari ATerosclerotiche Istituto Superiore di Sanità (MATISS)	Community	rural	19-69	19-69	1709	1921	
1687	Italy	1983-1985	Gubbio Study	Community	both	5+	5+	2431	2749	
1688	Italy	1985	Finland, Italy, Netherlands, Elderly (FINE-Italy)	Community	rural	65-84		650		
1689	Italy	1985	INTERSALT, Naples	Community	urban	20-59	20-59	100	100	
1690	Italy	1985-1988	Pisa epidemiological study - first survey	Community	urban	5-90	5-90	1834	2019	
1691	Italy	1986	INTERSALT, Bassiano	Community	urban	20-59	20-59	99	100	
1692	Italy	1986	INTERSALT, Mirano	Community	urban	20-59	20-59	100	100	
1693	Italy	1986	MONICA, Friuli	Subnational	both	25-64	25-64	921	918	
1694	Italy	1986-1987	Malattie cardiovascolari ATerosclerotiche Istituto Superiore di Sanità (MATISS)	Community	rural	19-72	19-72	1273	1568	
1695	Italy	1986-1987	MONICA, Brianza	Subnational	urban	25-64	25-64	814	832	
1696	Italy	1988-1991	Po river delta epidemiological study - second survey	Community	rural	8-73	8-73	1341	1497	
1697	Italy	1989	Ventimiglia Heart Study	Community	rural	5+	5+	603	701	
1698	Italy	1989	MONICA, Friuli	Subnational	both	25-64	25-64	902	900	
1699	Italy	1989-1990	MONICA, Brianza	Subnational	urban	25-64	25-64	787	786	
1700	Italy	1989-1992	Gubbio Study	Community	both	10+	10+	1677	1958	
1701	Italy	1990	Bruneck Study	Community	rural	40-79	40-79	469	450	
1702	Italy	1991	Finland, Italy, Netherlands, Elderly (FINE-Italy)	Community	rural	70-90		389		
1703	Italy	1991-1993	Pisa epidemiological study - second survey	Community	urban	8-97	8-97	1288	1553	
1704	Italy	1992-1993	Italian Longitudinal Study on Aging	National	both	65-84	65-84	1666	1455	
1705	Italy	1992-1998	Vobarno Study	Community	both	25-64	35-64	265	309	
1706	Italy	1993-1994	MONICA, Brianza	Subnational	urban	25-64	25-64	801	856	
1707	Italy	1993-1996	Malattie cardiovascolari ATerosclerotiche Istituto Superiore di Sanità (MATISS)	Community	rural	20-77	20-77	965	999	
1708	Italy	1993-1998	EPIC Florence	Community	urban	24-72	24-72	3498	9968	
1709	Italy	1994	MONICA, Friuli	Subnational	both	25-64	25-64	882	888	
1710	Italy	1995	Bruneck Study	Community	rural	45-84	45-84	411	408	
1711	Italy	1995-1996	Friuli Studio Emostatico	Community	urban	45-64	45-64	198	198	
1712	Italy	1995-1996	Italian Longitudinal Study on Aging	National	both	68-90	68-90	1011	808	
1713	Italy	1995-1999	PROgetto Veneto Anziani (PROVA)	Subnational	both	65+	65+	1187	1722	
1714	Italy	1997-1999	Lucca CUORE Study	Community	urban	15-84	15-84	897	1123	
1715	Italy	1998-1999	Progetto VIP	Community	both	25-74	25-74	599	600	
1716	Italy	1998-2000	InCHIANTI study	Community	both	15+	15+	560	681	
1717	Italy	1998-2002	Osservatorio Epidemiologico Cardiovascolare (OEC)	National	both	35-74	35-74	4870	4752	
1718	Italy	2000	Sorveglianza Nutrizionale Infanzia e Adolescenza (SoNIA)	Subnational	both	13-15	13-15	236	244	
1719	Italy	2000	Bruneck Study	Community	rural	50-89	50-89	331	361	
1720	Italy	2000-2001	Sorveglianza Nutrizionale Infanzia e Adolescenza (SoNIA)	Subnational	both	8-9	8-9	444	413	
1721	Italy	2000-2001	Italian Longitudinal Study on Aging	National	both	73-93	73-93	557	473	
1722	Italy	2000-2003	PROgetto Veneto Anziani (PROVA)	Subnational	both	67+	67+	795	1331	
1723	Italy	2001-2003	The Study of Asti	Community	both	45-64	45-64	780	878	
1724	Italy	2001-2007	Gubbio Study	Community	both	26+	26+	1187	1454	
1725	Italy	2002-2005	PROgetto Veneto Anziani (PROVA)	Subnational	both	68+	68+	621	1138	
1726	Italy	2003	Sorveglianza Nutrizionale Infanzia e Adolescenza (SoNIA)	Subnational	both	5-6	5-6	1355	1327	
1727	Italy	2003	The European Male Ageing Study	Community	both	40+		433		
1728	Italy	2004-2005	Italian Project on the Epidemiology of Alzheimer's Disease	National	both	65-84	65-84	1569	1421	
1729	Italy	2004-2005	Vobarno study	Community	rural	55-74	55-74	99	113	
1730	Italy	2004-2008	Cardiolab project	National	urban	40+	40+	19022	14526	
1731	Italy	2005	Bruneck Study	Community	rural	55-93	55-93	264	307	
1732	Italy	2005-2007	Moli-family Study	Subnational	both	14+	14+	243	301	
1733	Italy	2005-2010	Moli-sani Study	Subnational	both	35+	35+	11693	12614	
1734	Italy	2006-2007	HELENA	Community	urban	12-17	12-17	119	185	
1735	Italy	2007-2008	M.A.RE.A. (Metabolic Alterations in Reggio Calabria Adolescents) Study	Community	urban	11-13	11-13	301	314	

	Country	Study years	Survey/Study name/Citation	Level of representative-ness	Rural, urban, or both	Age range as in NCD-RisC database		Sample size		Note
						Male	Female	Male	Female	
1736	Italy	2007-2010	Identification and prevention of Dietary- and lifestyle-induced health EFfects In Children and infantS (IDEFICS)	Community	urban	5-9	5-9	893	847	
1737	Italy	2008	Childhood Obesity Surveillance Initiative 1	National	both	8-9	8-9	4100	3896	
1738	Italy	2008	The European Male Ageing Study	Community	both	45+		346		
1739	Italy	2008-2009	Progetto VIP	Community	both	25-74	25-74	597	598	
1740	Italy	2008-2012	Osservatorio Epidemiologico Cardiovascolare/Health Examination Survey (OEC/HES)	National	both	35-80	35-80	4368	4332	
1741	Italy	2009	The ZOOM8 study: nutrition and physical activity of primary school children	National	both	6-11	6-11	1083	1045	
1742	Italy	2009-2010	Grosso et al., J Epidemiol 24(4):327-33, 2014	Community	both	19+	19+	760	1129	
1743	Italy	2009-2011	Pisa epidemiological study - third survey	Community	urban	6+	6+	496	574	
1744	Italy	2010	Childhood Obesity Surveillance Initiative 2	National	both	8-9	8-9	21474	20190	
1745	Italy	2010	Bruneck Study	Community	rural	60+	60+	225	259	
1746	Italy	2010-2012	CArdiovascular risk MEtabolic syndrome LIver and Autoimmunity diseases (CA.ME.LI.A)	Community	both	18-75	18-75	477	515	
1747	Italy	2011	CONVERGI Study	Community	urban	13-19	13-19	159	269	
1748	Italy	2011	Grosso et al., Nutrients 5(12):4908-23, 2013	Community	rural	13-16	13-16	115	89	
1749	Italy	2011	Grosso et al., Nutrients 5(12):4908-23, 2013	Community	urban	13-16	13-16	512	419	
1750	Italy	2011-2012	ALImentazione e stile di vita negli ADOlescenti (ALIADO)	Subnational	both	15-16	15-16	149	194	
1751	Italy	2011-2012	Vobarno study	Community	rural	49-62	49-62	107	143	
1752	Italy	2012	Childhood Obesity Surveillance Initiative 3	National	both	8-9	8-9	23137	22405	
1753	Italy	2012-2014	Mistretta et al., Obes Res Clin Pract 11(2):215-226, 2017	Community	urban	11-16	11-16	878	753	
1754	Italy	2014	OKkio alla SALUTE	National	both	8-9	8-9	24457	22855	
1755	Italy	2014-2016	Mediterranean healthy Eating, Aging and Lifestyles (MEAL) study	Subnational	urban	20+	20+	762	1130	
1756	Italy	2015	Bruneck Study	Community	rural	65+	65+	171	169	
1757	Italy	2016	Childhood Obesity Surveillance Initiative 4	National	both	8-9	8-9	22732	21454	
1758	Italy	2016	The Tyrolean Early Vascular Ageing-study (EVA-Tyrol) - South-Tyrol	Subnational	both	14-18	14-18	108	200	
1759	Italy	2017-2020	Moli-sani Study	Subnational	both	47+	47+	1160	1422	
1760	Italy	2018-2019	Progetto VIP	Community	both	25-74	25-74	600	598	
1761	Italy	2018-2019	Health Examination Survey 2018-2019 - CUORE Project	National	urban	35-74	35-74	1035	1060	
1762	Italy	2019	Childhood Obesity Surveillance Initiative 5	National	both	8-9	8-9	23432	22462	
1763	Italy	2023	Childhood Obesity Surveillance Initiative 6	National	both	8-9	8-9	21522	20574	
1764	Italy	2024-2025	Campa et al., PLoS One 20(6):e0326111, 2025	Community	both	18-65	18-65	1313	1194	
1765	Jamaica	1993	Zohoori et al., West Indian Med J 52(2):111-17, 2003	Community	urban	25-74	25-74	845	1245	
1766	Jamaica	1994-1995	Cooper et al., Am J Public Health 87(2):160-68, 1997	Community	urban	25-100	25-100	597	833	
1767	Jamaica	1998	Ragoobirsingh et al., Diabetes Obes Metab 6(1):23-27, 2004	National	both	15+	15+	552	945	
1768	Jamaica	2000-2001	Jamaica Health and Lifestyle Survey	National	both	15-74	15-74	653	1281	
1769	Jamaica	2005	Jamaica Youth Risk and Resiliency Behaviour Survey 2005	National	both	10-15	10-15	1328	1386	
1770	Jamaica	2006-2007	Jamaica Youth Risk and Resiliency Behaviour Survey 2006	National	both	15-19	15-19	585	701	
1771	Jamaica	2007-2008	Jamaica Health and Lifestyle Survey	National	both	15-74	15-74	862	1904	
1772	Jamaica	2010	Global School-based Student Health Survey	National	both		13-17		787	
1773	Jamaica	2012	Older Persons in Jamaica 2012	National	both	60+	60+	158	205	21
1774	Jamaica	2016-2017	Jamaica Health and Lifestyle Survey	National	both	15+	15+	939	1491	
1775	Jamaica	2023	Global School-based Student Health Survey (St Catherine)	Community	urban	12-17	12-17	1273	1511	
1776	Japan	1977	National Nutrition Survey	National	both	5+	5+	6455	8094	1
1777	Japan	1978	National Nutrition Survey	National	both	5+	5+	6979	8694	1
1778	Japan	1979	National Nutrition Survey	National	both	5+	5+	7249	9031	1
1779	Japan	1980	National Cardiovascular Survey	National	both	5+	5+	7955	9437	
1780	Japan	1980-1983	Aito Town Study	Community	rural	20-77	20-77	741	970	
1781	Japan	1981	National Nutrition Survey	National	both	5+	5+	5922	7828	
1782	Japan	1982	National Nutrition Survey	National	both	5+	5+	6845	8831	
1783	Japan	1983	National Nutrition Survey	National	both	5+	5+	6609	8478	
1784	Japan	1984	National Nutrition Survey	National	both	5+	5+	6389	8072	
1785	Japan	1985	National Nutrition Survey	National	both	5+	5+	7461	8865	
1786	Japan	1985	INTERSALT, Osaka	Community	urban	20-59	20-59	100	97	
1787	Japan	1985	INTERSALT, Tochigi	Community	urban	20-59	20-59	95	99	
1788	Japan	1985	INTERSALT, Toyama	Community	urban	20-59	20-59	100	100	
1789	Japan	1985-1986	Akabane Study	Community	urban	40-69	40-69	812	1022	
1790	Japan	1986	National Nutrition Survey	National	both	5+	5+	7280	8635	
1791	Japan	1987	National Nutrition Survey	National	both	5+	5+	6427	8160	
1792	Japan	1987	Konan Town Study	Community	rural	20-79	20-79	70	88	
1793	Japan	1988	National Nutrition Survey	National	both	5+	5+	6885	8045	
1794	Japan	1988	Konan Town Study	Community	rural	20-79	20-79	76	85	
1795	Japan	1988	The Hisayama Study	Community	rural	40+	40+	1165	1573	
1796	Japan	1989	National Nutrition Survey	National	both	5+	5+	5767	6882	
1797	Japan	1989	Konan Town Study	Community	rural	20-79	20-79	58	63	
1798	Japan	1989	Aito Town Study	Community	rural	5-74	5-84	529	525	

	Country	Study years	Survey/Study name/Citation	Level of representative-ness	Rural, urban, or both	Age range as in NCD-RisC database		Sample size		Note
						Male	Female	Male	Female	
1799	Japan	1990	National Nutrition Survey and National Cardiovascular Survey	National	both	5+	5+	6080	7291	
1800	Japan	1990	Konan Town Study	Community	rural	20-79	20-79	27	51	
1801	Japan	1990-1994	Japan Public Health Center-based prospective Study (JPHC Study), Cohort I	Subnational	both	40-59	40-59	8749	14481	
1802	Japan	1991	National Nutrition Survey	National	both	5+	5+	6036	7098	
1803	Japan	1991	Konan Town Study	Community	rural	20-79	20-79	93	116	
1804	Japan	1991	Shigaraki Town Study	Community	rural	30-89	30-89	230	319	
1805	Japan	1992	National Nutrition Survey	National	both	5+	5+	5635	6656	
1806	Japan	1992	Konan Town Study	Community	rural	20-79	20-79	45	47	
1807	Japan	1992	Shigaraki Town Study	Community	rural	30-89	30-89	288	385	
1808	Japan	1993	Iwata kids health study	Community	urban	10	10	513	485	
1809	Japan	1993	National Nutrition Survey	National	both	5+	5+	5708	6740	
1810	Japan	1993	Konan Town Study	Community	rural	20-79	20-79	54	65	
1811	Japan	1993	Shigaraki Town Study	Community	rural	30-89	30-89	301	452	
1812	Japan	1993-1994	Japan Public Health Center-based prospective Study (JPHC Study), Cohort II	Subnational	both	40-69	40-69	8534	16190	
1813	Japan	1994	Iwata kids health study	Community	urban	10	10	569	567	
1814	Japan	1994	National Nutrition Survey	National	both	5+	5+	5439	6386	
1815	Japan	1994	Konan Town Study	Community	rural	20-79	20-79	43	59	
1816	Japan	1994	Shigaraki Town Study	Community	rural	30-89	30-89	251	336	
1817	Japan	1994	Japanese Population-Based Osteoporosis Study	Subnational	both		15-79		3222	
1818	Japan	1995	Iwata kids health study	Community	urban	10	10	524	567	
1819	Japan	1995	National Nutrition Survey	National	both	5+	5+	5480	6365	
1820	Japan	1995	Konan Town Study	Community	rural	20-79	20-79	45	61	
1821	Japan	1995	Shigaraki Town Study	Community	rural	30-89	30-89	300	470	
1822	Japan	1996	Iwata kids health study	Community	urban	10	10	552	480	
1823	Japan	1996	National Nutrition Survey	National	both	5+	5+	5277	6185	
1824	Japan	1996	Shigaraki Town Study	Community	rural	30-89	30-89	86	152	
1825	Japan	1996-1997	INTERMAP, AitoTown	Community	rural	40-59	40-59	130	129	
1826	Japan	1997	Iwata kids health study	Community	urban	10	10	506	537	
1827	Japan	1997	National Nutrition Survey	National	both	5+	5+	5104	6068	
1828	Japan	1997	Shigaraki Town Study	Community	rural	30-89	30-89	61	100	
1829	Japan	1997-1998	INTERMAP, Sapporo	Community	urban	40-59	40-59	149	148	
1830	Japan	1997-1998	INTERMAP, Toyama	Community	urban	40-59	40-59	149	150	
1831	Japan	1997-1998	INTERMAP, Wakayama	Community	urban	40-59	40-59	146	144	
1832	Japan	1997-2000	Sudo et al., J Orthop Sci 13(5):413-18, 2008	Community	rural	55+	45+	261	785	
1833	Japan	1998	Iwata kids health study	Community	urban	10	10	527	464	
1834	Japan	1998	National Nutrition Survey	National	both	5+	5+	5381	6249	
1835	Japan	1998	Niigata Study	Community	urban	70	70	287	284	
1836	Japan	1999	Iwata kids health study	Community	urban	10	10	468	463	
1837	Japan	1999	National Nutrition Survey	National	both	5+	5+	4367	5333	
1838	Japan	1999	Niigata Study	Community	urban	71	71	245	216	
1839	Japan	2000	Iwata kids health study	Community	urban	10	10	440	401	
1840	Japan	2000	National Nutrition Survey and National Cardiovascular Survey	National	both	5+	5+	4665	5430	
1841	Japan	2000	Niigata Study	Community	urban	72	72	233	202	
1842	Japan	2001	Iwata kids health study	Community	urban	10	10	452	414	
1843	Japan	2001	National Nutrition Survey	National	both	5+	5+	4527	5448	
1844	Japan	2001	Niigata Study	Community	urban	73	73	235	201	
1845	Japan	2001	The Japan Association of Health Service Database	Subnational	both	20+	20+	1471868	1231378	
1846	Japan	2002	Iwata kids health study	Community	urban	10	10	496	398	
1847	Japan	2002	National Nutrition Survey	National	both	5+	5+	4104	4941	
1848	Japan	2002	Niigata Study	Community	urban	74	74	228	202	
1849	Japan	2002-2003	The Hisayama Study	Community	rural	40+	40+	1414	1884	
1850	Japan	2003	Iwata kids health study	Community	urban	10	10	415	399	
1851	Japan	2003	National Health and Nutrition Survey	National	both	5+	5+	4035	4920	
1852	Japan	2003	Niigata Study	Community	urban	75	75	215	189	
1853	Japan	2004	Iwata kids health study	Community	urban	10	10	463	412	
1854	Japan	2004	National Health and Nutrition Survey	National	both	5+	5+	3384	3952	
1855	Japan	2004	Niigata Study	Community	urban	76	76	215	185	
1856	Japan	2005	Iwata kids health study	Community	urban	10	10	476	420	
1857	Japan	2005	National Health and Nutrition Survey	National	both	5+	5+	3154	3802	
1858	Japan	2005	Niigata Study	Community	urban	77	77	203	184	
1859	Japan	2006	Iwata kids health study	Community	urban	10	10	417	391	
1860	Japan	2006	National Health and Nutrition Survey	National	both	5+	5+	3522	4165	
1861	Japan	2006	Niigata Study	Community	urban	78	78	199	194	

	Country	Study years	Survey/Study name/Citation	Level of representative-ness	Rural, urban, or both	Age range as in NCD-RisC database		Sample size		Note
						Male	Female	Male	Female	
1862	Japan	2007	Fukuroi kids health study	Community	urban	13-14	13-14	395	372	
1863	Japan	2007	Iwata kids health study	Community	urban	10	10	439	394	
1864	Japan	2007	National Health and Nutrition Survey	National	both	5+	5+	3520	4154	
1865	Japan	2007	Niigata Study	Community	urban	79	79	183	192	
1866	Japan	2008	Fukuroi kids health study	Community	urban	13-14	13-14	381	346	
1867	Japan	2008	Iwata kids health study	Community	urban	10	10	406	417	
1868	Japan	2008	MEXT School Health Statistics	National	both	5-17	5-17	326405	326957	
1869	Japan	2008	National Health and Nutrition Survey	National	both	5+	5+	3518	4190	
1870	Japan	2008	Niigata Study	Community	urban	80	80	174	180	
1871	Japan	2008	Resident in Kanazawa City age 40+	Community	urban	40+	40+	6562	11944	
1872	Japan	2009	Fukuroi kids health study	Community	urban	13-14	13-14	388	357	
1873	Japan	2009	MEXT School Health Statistics	National	both	5-17	5-17	326525	327098	
1874	Japan	2009	National Health and Nutrition Survey	National	both	5+	5+	3486	4197	
1875	Japan	2010	Fukuroi kids health study	Community	urban	13-14	13-14	360	387	
1876	Japan	2010	MEXT School Health Statistics	National	both	5-17	5-17	326509	326401	
1877	Japan	2010	National Health and Nutrition Survey	National	both	5+	5+	3218	3822	
1878	Japan	2011	Fukuroi kids health study	Community	urban	13-14	13-14	402	369	
1879	Japan	2011	MEXT School Health Statistics	National	both	5-17	5-17	305270	306383	
1880	Japan	2011	National Health and Nutrition Survey	National	both	5+	5+	3020	3586	
1881	Japan	2011	The Tokyo Health Service Association Database	Community	urban	20+	20+	82453	54028	
1882	Japan	2012	Fukuroi kids health study	Community	urban	13-14	13-14	432	353	
1883	Japan	2012	MEXT School Health Statistics	National	both	5-17	5-17	326524	326572	
1884	Japan	2012	National Health and Nutrition Survey	National	both	5+	5+	11298	13674	
1885	Japan	2012-2016	The Nagahama study	Community	rural	35-80	35-80	3206	6620	
1886	Japan	2013	Awaji Child Health Study	Community	urban	10-14	10-14	198	203	
1887	Japan	2013	Fukuroi kids health study	Community	urban	13-14	13-14	387	404	
1888	Japan	2013	MEXT School Health Statistics	National	both	5-17	5-17	327923	327578	
1889	Japan	2013	National Health and Nutrition Survey	National	both	5+	5+	3198	3637	
1890	Japan	2014	Awaji Child Health Study	Community	urban	10-14	10-14	229	218	
1891	Japan	2014	MEXT School Health Statistics	National	both	5-17	5-17	327062	326884	
1892	Japan	2014	National Health and Nutrition Survey	National	both	5+	5+	3208	3657	
1893	Japan	2014-2015	Nagaoka Health Screening	Community	both	20-89	20-89	4938	4298	
1894	Japan	2015	Awaji Child Health Study	Community	urban	10-14	10-14	230	228	
1895	Japan	2015	MEXT School Health Statistics	National	both	5-17	5-17	326382	327210	
1896	Japan	2015	National Health and Nutrition Survey	National	both	5+	5+	2914	3457	
1897	Japan	2015-2019	The Shizuoka KDB study	Subnational	both	75-90	75-90	44361	92170	
1898	Japan	2016	MEXT School Health Statistics	National	both	5-17	5-17	334734	334444	
1899	Japan	2016	National Health and Nutrition Survey	National	both	5+	5+	9814	11638	
1900	Japan	2017	MEXT School Health Statistics	National	both	5-17	5-17	333184	333723	
1901	Japan	2017	National Health and Nutrition Survey	National	both	5+	5+	2662	3057	
1902	Japan	2017	The Tokyo Health Service Association Database	Community	urban	20+	20+	63713	47577	
1903	Japan	2017-2018	The Hisayama Study	Community	rural	40+	40+	1510	1947	
1904	Japan	2018	MEXT School Health Statistics	National	both	5-17	5-17	337022	336953	
1905	Japan	2018	National Health and Nutrition Survey	National	both	5+	5+	2790	3144	
1906	Japan	2019	MEXT School Health Statistics	National	both	5-17	5-17	332231	333546	
1907	Japan	2019	National Health and Nutrition Survey	National	both	5+	5+	2277	2606	
1908	Japan	2020	MEXT School Health Statistics	National	both	5-17	5-17	333405	333857	
1909	Japan	2021	MEXT School Health Statistics	National	both	5-17	5-17	332498	332303	
1910	Japan	2022	MEXT School Health Statistics	National	both	5-17	5-17	324388	324854	
1911	Japan	2023	MEXT School Health Statistics	National	both	5-17	5-17	324874	324873	
1912	Japan	2024	MEXT School Health Statistics	National	both	5-17	5-17	325051	324089	
1913	Jordan	1994-1996	Ajlouni, Int J Obes Relat Metab Disord 22(7), 1998	Subnational	both	25+	25+	1047	1787	
1914	Jordan	1997	DHS	National	both		20-49		3002	
1915	Jordan	2002	DHS	National	both		20-49		4839	
1916	Jordan	2004	Behavioural Risk Factor Surveillence Survey	National	rural	18+	18+	236	473	
1917	Jordan	2004	Khader et al., Metab Syndr Relat Disord 6(2):113-20, 2008	Community	both	25+	25-59	394	548	
1918	Jordan	2007	DHS	National	both		20-49		4451	
1919	Jordan	2007	Behavioural Risk Factor Surveillence Survey	National	both	18+	18+	332	433	
1920	Jordan	2009	Metablic abnomalities and vitamin D study	National	both	7+	7+	1601	3863	
1921	Jordan	2009	DHS	National	both		20-49		4054	
1922	Jordan	2012	DHS	National	both		20-49		6357	
1923	Jordan	2015-2016	Zayed et al., BMC Public Health 16(1):1040, 2016	Subnational	rural	6-15	6-17	407	288	
1924	Jordan	2015-2016	Zayed et al., BMC Public Health 16(1):1040, 2016	Subnational	urban	6-15	6-17	565	974	

	Country	Study years	Survey/Study name/Citation	Level of representative-ness	Rural, urban, or both	Age range as in NCD-RisC database		Sample size		Note
						Male	Female	Male	Female	
1925	Jordan	2016-2017	National Cardiovascular Diseases and Diabetes Study (NCDDS)	National	both	18+	18+	1187	2745	
1926	Jordan	2017-2018	DHS	National	both		15-49		6261	
1927	Jordan	2018-2019	Anthropometric Indices of Obesity as Predictors of High Blood Pressure among School Children	Community	urban	10-14	10-14	284	504	
1928	Jordan	2019	National Micronutrient and Nutrition Survey	National	both	6-12	6-12	514	483	
1929	Jordan	2019	STEPS	National	both	18-69	18-69	2009	3084	
1930	Jordan	2019	Jordan National Micronutrient and Nutrition Survey (15-49 women)	National	both		15-49		621	
1931	Jordan	2022	Jordan Population-based Food Consumption Survey	Subnational	both	8+	8+	944	1194	
1932	Jordan	2023	DHS	National	both		15-49		6788	
1933	Jordan	2024	Global School-based Student Health Survey	National	both	12-17	12-17	2080	1841	
1934	Jordan	2025	STEPS	National	both	18-69	18-69	2486	2889	22
1935	Kazakhstan	1985	Balakhmetova et l., Ter Arkh 63(1):17-20, 1991	Community	urban	20-54		2886		
1936	Kazakhstan	1995	DHS	National	both		15-49		3542	
1937	Kazakhstan	1999	DHS	National	both		15-49		2227	
1938	Kazakhstan	2011-2012	Household Health Survey	National	both	15+	15+	4578	6044	
1939	Kazakhstan	2015	Almaty STEPS	Subnational	both	18-69	18-69	381	1136	
1940	Kazakhstan	2015	Shymkent STEPS	Subnational	both	18-69	18-69	400	793	
1941	Kazakhstan	2015-2016	Childhood Obesity Surveillance Initiative 4	National	both	8-10	8-10	2755	2683	
1942	Kazakhstan	2015-2016	Aktobe STEPS	Subnational	both	18-69	18-69	348	1144	
1943	Kazakhstan	2018-2019	Early diagnosis of metabolic syndrome in children and adolescents of Semey Region	Subnational	both	6-18	6-18	1008	963	
1944	Kazakhstan	2019	A health status assessment of a population of Karaganda urban region	Community	urban	18+	18+	324	670	
1945	Kazakhstan	2020	Childhood Obesity Surveillance Initiative 5	National	both	6-9	6-9	3396	3379	
1946	Kazakhstan	2021-2022	Prevalence of risk factors for NCD in Kazakhstan	National	both	18-69	18-69	3327	3219	23
1947	Kazakhstan	2022	Childhood Obesity Surveillance Initiative 6	Community	both	7-9	7-9	1026	1042	
1948	Kenya	1985	INTERSALT	Community	rural	20-59	20-59	90	86	
1949	Kenya	1993	DHS	National	both		20-49		3113	
1950	Kenya	1998	DHS	National	both		20-49		3009	
1951	Kenya	2003	DHS	National	both		15-49		7189	
1952	Kenya	2008-2009	DHS	National	both		15-49		7827	
1953	Kenya	2011	Kenya National Micronutrient Survey	National	both		5-49		500	
1954	Kenya	2011-2013	International Study of Childhood Obesity, Lifestyle and the Environment (ISCOLE)	Community	urban	9-11	9-11	262	301	
1955	Kenya	2014	DHS	National	both		15-49		13469	
1956	Kenya	2015	STEPS	National	both	18-69	18-69	1750	2511	
1957	Kenya	2015-2016	Kenya Integrated Household Budget Survey (KIHBS)	National	both	5+	5+	30062	34334	
1958	Kenya	2018	Assessing the gaps in healthcare and determining the feasibility for the setup of a social enterprise - Viwandani Lown Community Health Center, Kenya	Community	urban	19-73	19-73	153	143	
1959	Kenya	2022	DHS	National	both	15-54	15-49	5731	9541	
1960	Kiribati	1981	Epidemiological survey of Kiribati	Subnational	rural	20+	20+	473	532	
1961	Kiribati	1981	Epidemiological survey of Kiribati	Subnational	urban	20+	20+	939	906	
1962	Kiribati	2004	STEPS	National	both	15-64	15-64	779	939	
1963	Kiribati	2011	Global School-based Student Health Survey	National	both		13		156	
1964	Kiribati	2015-2016	STEPS	National	both	18-69	18-69	557	694	
1965	Kiribati	2022	Global School-based Student Health Survey	National	both	13-17	13-17	810	1203	
1966	Kiribati	2023-2024	STEPS	National	both	18-69	18-69	1127	1397	
1967	Kuwait	1980-1981	al-Isa, Ann Nutr Metab 41(5):307-14, 1997	Community	both	18+		959		
1968	Kuwait	1993-1994	al-Isa, Ann Nutr Metab 41(5):307-14, 1997	Community	both	18+		1730		
1969	Kuwait	1995-1996	Abdella et al., Diabetes Res and Clin Pract 42(3):187-196, 1998	Subnational	both	20-84	20-84	1099	1892	
1970	Kuwait	1998	Abiaka et al., Biol Trace Elem Res 91(1):33-43, 2003	National	both	15-80	15-80	178	233	
1971	Kuwait	2001	Kuwait Nutrition Surveillance System	National	urban	5-19	5-19	4520	5389	
1972	Kuwait	2001	Kuwait Nutrition Surveillance System	National	urban	20+	20+	822	1686	
1973	Kuwait	2002	Kuwait Nutrition Surveillance System	National	urban	5-19	5-19	5517	5239	
1974	Kuwait	2002	Kuwait Nutrition Surveillance System	National	urban	20+	20+	1694	2368	
1975	Kuwait	2003	Kuwait Nutrition Surveillance System	National	both	5-19	5-19	5179	5132	
1976	Kuwait	2003	Kuwait Nutrition Surveillance System	National	both	20+	20+	1285	1192	
1977	Kuwait	2004	Kuwait Nutrition Surveillance System	National	both	5-19	5-19	5021	4880	
1978	Kuwait	2004	Kuwait Nutrition Surveillance System	National	both	20+	20+	1209	1748	
1979	Kuwait	2005	Kuwait Nutrition Surveillance System	National	both	5-19	5-19	4520	5389	
1980	Kuwait	2005	Kuwait Nutrition Surveillance System	National	both	20+	20+	1493	1642	
1981	Kuwait	2006	Kuwait Nutrition Surveillance System	National	both	5-19	5-19	5259	5066	
1982	Kuwait	2006	STEPS	National	both	20-64	20-64	918	1298	
1983	Kuwait	2006	Kuwait Nutrition Surveillance System	National	both	20+	20+	1290	1661	
1984	Kuwait	2007	Kuwait Nutrition Surveillance System	National	both	5-19	5-19	5481	5502	
1985	Kuwait	2007	Kuwait Nutrition Surveillance System	National	both	20+	20+	1711	1706	
1986	Kuwait	2008	Kuwait Nutrition Surveillance System	National	both	5-19	5-19	5757	5595	

	Country	Study years	Survey/Study name/Citation	Level of representative-ness	Rural, urban, or both	Age range as in NCD-RisC database		Sample size		Note
						Male	Female	Male	Female	
1987	Kuwait	2008	Kuwait Nutrition Surveillance System	National	both	20+	20+	1604	1669	
1988	Kuwait	2008-2009	National Nutrition Program for the State of Kuwait	National	both	5+	5+	772	830	24
1989	Kuwait	2008-2010	Gulf Cooperation Council World Health Survey	National	both	18+	18+	1598	1782	
1990	Kuwait	2009	Kuwait Nutrition Surveillance System	National	both	5-19	5-19	5358	5574	
1991	Kuwait	2009	Kuwait Nutrition Surveillance System	National	both	20+	20+	1331	1496	
1992	Kuwait	2011	Global School-based Student Health Survey	National	both	13-17	13-17	1265	1274	
1993	Kuwait	2011-2014	Kuwait Diabetes Epidemiology Program	National	both	18-82	18-82	3007	2242	
1994	Kuwait	2014	STEPS	National	both	18-69	18-69	1382	2212	
1995	Kuwait	2015	Global School-based Student Health Survey	National	both	13-17	13-17	1363	1553	
1996	Kyrgyzstan	1993	Kyrgyzstan Multipurpose Poverty Survey	National	both	5+	5+	3512	3786	
1997	Kyrgyzstan	1997	DHS	National	both		15-49		3570	
1998	Kyrgyzstan	2006-2007	Kyrgyzstan Integrated Household Survey	National	both	18+	18+	4424	5745	
1999	Kyrgyzstan	2012	DHS	National	both		15-49		7516	
2000	Kyrgyzstan	2013	STEPS	National	both	25-64	25-64	942	1600	
2001	Kyrgyzstan	2014-2015	Kyrgyzstan Integrated Household Survey	National	both	18+	18+	4958	6069	
2002	Kyrgyzstan	2015-2016	Childhood Obesity Surveillance Initiative 4	National	both	6-9	6-9	3945	3905	
2003	Kyrgyzstan	2021	National Integrated Micronutrient and Anthropometric Survey of the Kyrgyz Republic	National	both	5-9	5-49	730	2511	
2004	Kyrgyzstan	2023	Childhood Obesity Surveillance Initiative 6	National	both	7-8	7-8	1504	1494	
2005	Kyrgyzstan	2023	STEPS	National	both	18-69	18-69	850	1536	
2006	Lao PDR	2006	Multiple Indicator Cluster Survey 3	National	both		15-49		807	
2007	Lao PDR	2008	STEPS	Community	both	25-64	25-64	1568	2353	
2008	Lao PDR	2013	STEPS	National	both	18-64	18-64	984	1461	
2009	Latvia	1997	Nutrition and Lifestyle in the Baltic Republics, WHO, 1997	National	both	19-50	19-50	703	732	
2010	Latvia	2008	Childhood Obesity Surveillance Initiative 1	National	both	7-8	7-8	2283	2101	
2011	Latvia	2008-2009	Cardiovascular risk factor study	National	both	25-74	25-74	1362	2398	
2012	Latvia	2010	Childhood Obesity Surveillance Initiative 2	National	both	7-8	7-8	2093	2053	
2013	Latvia	2012	Childhood Obesity Surveillance Initiative 3	National	both	6-7	6-7	1804	1677	
2014	Latvia	2015-2016	Childhood Obesity Surveillance Initiative 4	National	both	7-9	7-9	2952	2991	
2015	Latvia	2019	Childhood Obesity Surveillance Initiative 5	National	both	6-9	6-9	3420	3441	
2016	Latvia	2020-2021	Study on physical activity, dietary habits and body mass index in 5-6 year old preschool children in Latvia	National	both	5-6	5-6	1886	1858	
2017	Latvia	2023	Childhood Obesity Surveillance Initiative 6	National	both	7-10	7-10	3171	3184	
2018	Lebanon	1997	Obesity in Lebanon: National Survey	National	both	5+	5+	871	1164	
2019	Lebanon	2008-2009	STEPS	National	both	5+	5+	1721	1886	
2020	Lebanon	2017	STEPS	National	both	18-69	18-69	729	983	
2021	Lebanon	2022	School and Community Drivers of Child Diets in Arab Cities: Identifying Levers for Intervention (SCALE)	Subnational	urban	9-13	9-13	914	1071	
2022	Lebanon	2023-2024	STEPS	National	both	18-69	18-69	1019	1790	
2023	Lesotho	1993	National survey on iodine, vitamin A and iron status of women and children in Lesotho	National	both		20-65		792	
2024	Lesotho	2004-2005	DHS	National	both		15-49		3206	
2025	Lesotho	2009-2010	DHS	National	both	15-59	15-49	3216	3781	
2026	Lesotho	2012	STEPS	National	both	25-64	25-64	726	1442	
2027	Lesotho	2014	DHS	National	both	15-59	15-49	2860	3244	
2028	Lesotho	2023-2024	DHS	National	both	15-59	15-49	1449	6741	
2029	Liberia	2006-2007	DHS	National	both		15-49		6419	
2030	Liberia	2010	Comprehensive Food Security and Nutrition Survey	National	both		15-49		7724	
2031	Liberia	2011	STEPS	Subnational	both	25-64	25-64	998	1254	
2032	Liberia	2013	DHS	National	both	15-49	15-49	4235	4718	
2033	Liberia	2019	DHS	National	both		15-49		3793	
2034	Liberia	2022	STEPS	National	both	20-69	20-69	1431	2197	
2035	Libya	1998-1999	Kadiki et al., Diabetes Metab 27(6):647-54, 2001	Community	both	15+	15+	228	398	
2036	Libya	2007	Global School-based Student Health Survey	National	both	12-15	12-15	697	995	
2037	Libya	2009	STEPS	National	both	25-64	25-64	1678	1564	
2038	Libya	2022-2023	STEPS	National	both	18-69	18-69	2246	2541	
2039	Lithuania	1983-1985	MONICA, Kaunas	Community	urban	35-64	35-64	728	735	
2040	Lithuania	1986-1987	MONICA, Kaunas	Community	urban	35-64	35-64	894	868	
2041	Lithuania	1987	Countrywide Integrated Noncommunicable Diseases Intervention Programme survey	Subnational	rural	25-64	25-64	1220	1434	
2042	Lithuania	1992-1993	MONICA, Kaunas	Community	urban	35-64	35-64	610	621	
2043	Lithuania	1992-1993	Countrywide Integrated Noncommunicable Diseases Intervention Programme survey	Subnational	rural	25-64	25-64	617	798	
2044	Lithuania	1997	Pomerleau et al., Public Health Nutrition 3(1):3-10, 2000	National	both	19+	19+	966	1130	
2045	Lithuania	1998-1999	Countrywide Integrated Noncommunicable Diseases Intervention Programme survey	Subnational	rural	25-64	25-64	816	1021	
2046	Lithuania	2001-2002	MONICA4	Community	urban	35-64	35-64	625	776	
2047	Lithuania	2002	Pomerleau, 2002	National	both	24-70	24-70	977	928	
2048	Lithuania	2006-2007	Countrywide Integrated Noncommunicable Diseases Intervention Programme survey	Subnational	rural	25-64	25-64	718	972	
2049	Lithuania	2006-2008	Health, Alcohol and Psychosocial Factors In Eastern Europe	Community	urban	45-72	45-72	3214	3857	

	Country	Study years	Survey/Study name/Citation	Level of representative-ness	Rural, urban, or both	Age range as in NCD-RisC database		Sample size		Note
						Male	Female	Male	Female	
2050	Lithuania	2008	Childhood Obesity Surveillance Initiative 1	National	both	7-8	7-8	2532	2344	
2051	Lithuania	2010	Childhood Obesity Surveillance Initiative 2	National	both	7-9	7-9	3306	3414	
2052	Lithuania	2010-2012	Prevalence and risk factors of high blood pressure in 12-15-year-old Lithuanian children and adolescents (Study 1, 2010-2012)	Community	both	12-15	12-15	3494	3963	
2053	Lithuania	2012-2013	Prevalence and risk factors of high blood pressure in 12-15-year-old Lithuanian children and adolescents (Study 2, 2012-2013)	Community	both	12-15	12-15	962	985	
2054	Lithuania	2013	Childhood Obesity Surveillance Initiative 3	National	both	7-8	7-8	1890	1895	
2055	Lithuania	2015-2016	Childhood Obesity Surveillance Initiative 4	National	both	7-8	7-8	1924	1876	
2056	Lithuania	2019	Childhood Obesity Surveillance Initiative 5	National	both	7-8	7-8	1649	1585	
2057	Lithuania	2019-2020	Prevalence and risk factors of high blood pressure and obesity in Lithuanian children and adolescents (Kaunas)	Community	both	7-19	7-19	26	16	
2058	Lithuania	2021-2022	Prevalence and risk factors of high blood pressure and obesity in Lithuanian children and adolescents (Kaunas)	Community	both	7-20	7-20	1274	1154	
2059	Lithuania	2023	Childhood Obesity Surveillance Initiative 6	National	both	7-8	7-8	1512	1613	
2060	Lithuania	2023-2024	MONICA 5	Community	urban	25-69	25-69	1522	1902	
2061	Luxembourg	2007-2009	Observation of cardiovascular risk factors in Luxembourg (ORISCAV-LUX)	National	both	18-69	18-69	696	735	
2062	Luxembourg	2013-2015	European Health Examination Survey in Luxembourg	National	both	25-64	25-64	721	785	
2063	Luxembourg	2016-2018	Observation of cardiovascular risk factors in Luxembourg (ORISCAV-LUX2)	Community	both	25-79	25-79	670	763	
2064	Luxembourg	2023	Childhood Obesity Surveillance Initiative 6	National	both	6-9	6-9	5231	5136	
2065	Madagascar	1997	DHS	National	both		20-49		2253	
2066	Madagascar	1997	Mauny et al., Ann Trop Med Parasitol 97(6):645-54, 2003	Community	both	15+	15+	248	283	
2067	Madagascar	2003-2004	DHS	National	both		15-49		7155	
2068	Madagascar	2005	STEPS	Subnational	both	25-64	25-64	2596	2494	
2069	Madagascar	2008-2009	DHS	National	both		15-49		7869	
2070	Madagascar	2021	DHS	National	both		15-49		8888	
2071	Malawi	1992	DHS	National	both		20-49		2102	
2072	Malawi	1996	Chilima et al., Eur J Clin Nutr 52(9):643-9, 1998	Community	rural	55-94	55-94	86	185	
2073	Malawi	2000	DHS	National	both		15-49		11491	
2074	Malawi	2004	DHS	National	both		15-49		9751	
2075	Malawi	2009	National Micronutrient Survey	National	both	6-12	6-49	344	932	
2076	Malawi	2009	STEPS	National	both	25-64	25-64	1666	3189	
2077	Malawi	2010	DHS	National	both		15-49		7118	
2078	Malawi	2013-2017	NCD Survey Malawi Epidemiology and Intervention Research Unit	Community	rural	18+	18+	5815	7466	
2079	Malawi	2013-2017	NCD Survey Malawi Epidemiology and Intervention Research Unit	Community	urban	18+	18+	5797	10283	
2080	Malawi	2015-2016	DHS	National	both		15-49		7415	
2081	Malawi	2017	STEPS	National	both	18-69	18-69	1478	2534	
2082	Malawi	2020	Nutrition SMART Survey Conducted in Flood- and Drought-Prone Livelihood Zones of Malawi	National	both	10-19	10-19	1539	1406	
2083	Malaysia	1996	National Health and Morbidity Survey (NHMS)	National	both	18+	18+	14520	16244	
2084	Malaysia	2002-2003	Malaysian Adult Nutrition Survey	National	both	18-59	18-59	3303	3395	
2085	Malaysia	2004	Rampal et al., Public Health 122(1):11-8, 2008	National	both	15+	15+	7028	9527	
2086	Malaysia	2005	STEPS	National	both	25-64	25-64	1286	1286	
2087	Malaysia	2006	National Health and Morbidity Survey (NHMS)	National	both	5+	5+	22970	25508	
2088	Malaysia	2008	National Iodine Deficiency Disorder (IDD) Survey	National	both	7-10	7-10	9388	8659	
2089	Malaysia	2008	Metabolic Syndrome Study in Malaysia	National	rural	18+	18+	753	1368	
2090	Malaysia	2008	Metabolic Syndrome Study in Malaysia	National	urban	18+	18+	769	1446	
2091	Malaysia	2010-2011	SEANUTS	National	both	5-12	5-12	1306	1352	
2092	Malaysia	2011	National Health and Morbidity Survey (NHMS)	National	both	5+	5+	8033	8780	
2093	Malaysia	2012	Malaysian School-Based Health Survey	National	both	9-17	9-17	20254	19652	
2094	Malaysia	2012-2013	Petaling District	Subnational	urban	12-17	12-17	882	1364	
2095	Malaysia	2013-2014	Batang Padang District	Subnational	both	12-17	12-17	2928	3319	
2096	Malaysia	2014	Malaysian Adult Nutrition Survey	National	both	18-59	18-59	1328	1495	
2097	Malaysia	2015	National Health and Morbidity Survey (NHMS)	National	both	5+	5+	12144	12871	
2098	Malaysia	2019	National Health and Morbidity Survey (NHMS)	National	both	5+	5+	6155	6838	
2099	Malaysia	2019	Prevalence and Predictors of Overweight and Obesity Among Adolescents in Seremban, Negeri Sembilan, Malaysia	Community	both	12-14	12-14	697	756	
2100	Maldives	2001	Multiple Indicator Cluster Survey	National	both		15-50		1145	
2101	Maldives	2004	STEPS	Subnational	urban	25-64	25-64	933	1086	
2102	Maldives	2009	Global School-based Student Health Survey	National	both	13-17		806		
2103	Maldives	2009	DHS	National	both		20-49		5139	
2104	Maldives	2011	STEPS	Subnational	urban	15-64	15-64	660	1060	
2105	Maldives	2014	Global School-based Student Health Survey	National	both	13-17		931		
2106	Maldives	2016-2017	DHS	National	both	15-49	15-49	3831	6839	
2107	Maldives	2020-2021	STEPS	Subnational	both	15-69	15-69	776	1861	
2108	Mali	1995-1996	DHS	National	both		20-49		3789	
2109	Mali	1997	Programme Intégré de Développement de Bafoulabé	Community	rural	15-45	15-45	425	716	
2110	Mali	1999-2000	Bafoulabe Iodine Study	Community	rural		15-45		365	
2111	Mali	2001	DHS	National	both		15-49		10526	
2112	Mali	2006	DHS	National	both		15-49		12512	

	Country	Study years	Survey/Study name/Citation	Level of representative-ness	Rural, urban, or both	Age range as in NCD-RisC database		Sample size		Note
						Male	Female	Male	Female	
2113	Mali	2007	STEPS	Subnational	both	15-64	15-64	1036	1494	
2114	Mali	2012-2013	DHS	National	both		15-49		4646	
2115	Mali	2013	STEPS	Subnational	both	15-65	15-65	525	949	
2116	Mali	2013	Santé Nutritionnelle à Assise Comunautaire dans la région de Kayes (SNACK)	Subnational	rural		20-68		4595	
2117	Mali	2015	Standardized Monitoring and Assessment of Relief and Transitions	National	both		15-49		6006	
2118	Mali	2018	DHS	National	both		15-49		4576	
2119	Mali	2019	Standardized Monitoring and Assessment of Relief and Transitions	National	both		15-49		11518	
2120	Mali	2020	Standardized Monitoring and Assessment of Relief and Transitions	National	both	10-19	10-49	1836	8703	
2121	Mali	2021	Standardized Monitoring and Assessment of Relief and Transitions	National	both	10-19	10-49	1558	7509	
2122	Malta	1984	MONICA, Malta	Community	both	25-64	25-64	948	929	
2123	Malta	1986	INTERSALT	Community	rural	20-59	20-59	100	100	
2124	Malta	2008	Childhood Obesity Surveillance Initiative 1	National	both	6	6	1084	1031	
2125	Malta	2010	Childhood Obesity Surveillance Initiative 2	National	both	6	6	1151	1170	
2126	Malta	2013	Childhood Obesity Surveillance Initiative 3	National	both	7-8	7-8	1757	1693	
2127	Malta	2014-2016	SAHHTEK - The University of Malta Health and Wellbeing Study	National	both	18-70	18-70	834	1024	
2128	Malta	2015-2016	Childhood Obesity Surveillance Initiative 4	National	both	7-8	7-8	2056	1905	
2129	Malta	2019	Childhood Obesity Surveillance Initiative 5	National	both	7-8	7-8	2085	1999	
2130	Malta	2022	Childhood Obesity Surveillance Initiative 6	National	both	7-8	7-8	1890	1642	
2131	Marshall Islands	2002	STEPS	National	both	15-64	15-64	772	1195	
2132	Marshall Islands	2017-2018	STEPS	National	both	18+	18+	1313	1488	
2133	Marshall Islands	2020	Rapid High School Survey	National	both	14-19	14-19	1158	1305	
2134	Marshall Islands	2022	Rapid High School Survey	National	both	14-19	14-19	1264	1481	
2135	Mauritania	2000-2001	DHS	National	both		15-49		7022	
2136	Mauritania	2006	STEPS	Community	urban	15-64	15-64	1132	1300	
2137	Mauritania	2011	Enquête Nutritionnelle SMART	National	both		15-49		6797	
2138	Mauritania	2019-2021	DHS	National	both		15-49		6820	
2139	Mauritius	1987	Mauritius Noncommunicable Disease Survey	National	both	25-74	25-74	2347	2653	
2140	Mauritius	1992	Mauritius Noncommunicable Disease Survey	National	both	25-74	25-74	2985	3477	
2141	Mauritius	1992	Rodrigues Noncommunicable Disease Survey 1992	Community	rural	25-64	25-64	736	770	
2142	Mauritius	1998	Mauritius Noncommunicable Disease Survey	National	both	25-74	25-74	2566	3248	
2143	Mauritius	1999	Rodrigues Noncommunicable Disease Survey 1999	Community	rural	20+	20+	974	1292	
2144	Mauritius	2009	Mauritius Noncommunicable Disease Survey	National	both	19-74	19-74	2859	3391	
2145	Mauritius	2011	Global School-based Student Health Survey	National	both	13-17	13-17	859	1043	
2146	Mauritius	2011	Global School-based Student Health Survey-Rodrigues	Subnational	both	13-17	13-17	425	546	
2147	Mauritius	2015	Mauritius Noncommunicable Disease Survey	National	both	20-74	20-74	1615	1944	
2148	Mauritius	2015	Mauritius Noncommunicable Disease Survey 1998 Follow Up	National	both	20+	20+	873	1164	
2149	Mauritius	2017	Global School-based Student Health Survey	National	both	13-17	13-17	1358	1525	
2150	Mauritius	2019	Global School-based Student Health Survey	Subnational	rural	12-17	12-17	1116	1289	
2151	Mauritius	2021	Mauritius Noncommunicable Disease Survey	National	both	19-84	19-84	1642	1927	
2152	Mexico	1988-1989	Encuesta Nacional de Nutricion	National	both		12-49		16426	
2153	Mexico	1992-1993	Encuesta Nacional de Enfermedades Cronicas	National	urban	20-69	20-69	6040	8298	
2154	Mexico	1996	Sanchez-Castillo et al., Eur J Clin Nutr 55(10):833-40, 2001	Community	rural	18+	18+	104	149	
2155	Mexico	1998-1999	Encuesta Nacional de Nutricion	National	both		12-49		17892	
2156	Mexico	1998-2004	Mexico City Prospective Study	Community	urban	35-84	35-84	51768	105313	
2157	Mexico	1999	National Survey on School Children	National	both	5-10	5-10	4898	5031	
2158	Mexico	1999-2000	The Survey on Health, Well-Being, and Aging in Latin America and the Caribbean (SABE)	Community	urban	60+	60+	359	548	4
2159	Mexico	2000	Encuesta Nacional de Salud	National	both	10+	10+	22554	39204	
2160	Mexico	2001	The Mexican Health and Aging Study	National	both	50+	50+	1030	1224	
2161	Mexico	2002	Encuesta Nacional Sobre Niveles de vida de los Hogares	National	both	5+	5+	11606	13614	
2162	Mexico	2003	The Mexican Health and Aging Study	National	both	50+	50+	893	1162	
2163	Mexico	2004-2005	CArdiovascular Risk factors Multiple Evaluation in Latin America (CARMELA)	Community	urban	25-64	25-64	833	894	
2164	Mexico	2005	Encuesta Nacional Sobre Niveles de vida de los Hogares	National	both	5+	5+	11696	13211	
2165	Mexico	2006	Encuesta Nacional de Salud y Nutrición	National	both	5+	5+	27848	34909	
2166	Mexico	2006	PREVENIMSS National Coverage Surveys	National	urban	20+	20+	8727	11335	
2167	Mexico	2009-2010	WHO Study on global AGEing and adult health (SAGE)	National	both	50+	50+	793	1234	
2168	Mexico	2009-2012	Encuesta Nacional Sobre Niveles de vida de los Hogares	National	both	5+	5+	12408	14385	
2169	Mexico	2010	PREVENIMSS National Coverage Surveys	National	urban	20+	20+	6238	6003	
2170	Mexico	2011-2012	Encuesta Nacional de Salud y Nutrición	National	both	5+	5+	31293	36834	
2171	Mexico	2012	The Mexican Health and Aging Study	National	both	50+	50+	786	1106	
2172	Mexico	2014	WHO Study on global AGEing and adult health (SAGE), Wave 2	National	both	18+	18+	1658	2417	
2173	Mexico	2016	Encuesta Nacional de Salud y Nutrición	National	both	5+	5+	5659	8424	
2174	Mexico	2016	Cognitive Aging Linked to MHAS (Mex-Cog)	National	both	55+	55+	786	1141	25
2175	Mexico	2018-2019	Encuesta Nacional de Salud y Nutrición	National	both	5+	5+	11581	13420	

	Country	Study years	Survey/Study name/Citation	Level of representative-ness	Rural, urban, or both	Age range as in NCD-RisC database		Sample size		Note
						Male	Female	Male	Female	
2176	Mexico	2020	Encuesta Nacional de Salud y Nutrición	National	both	5+	5+	5593	7459	
2177	Mexico	2021	Encuesta Nacional de Salud y Nutrición	National	both	5+	5+	5798	7949	
2178	Mexico	2022	Encuesta Nacional de Salud y Nutrición	National	both	5+	5+	5441	7550	
2179	Micronesia	2002	STEPS	Subnational	both	25-64	25-64	591	893	
2180	Micronesia	2006	STEPS	Subnational	both	15-64	15-64	918	1553	
2181	Micronesia	2008	STEPS	Subnational	both	25-64	25-64	875	1266	
2182	Micronesia	2009	STEPS, Yap	Subnational	both	15-64	15-64	405	521	
2183	Micronesia	2009	STEPS, Kosrae	Subnational	both	15-64	15-64	208	413	
2184	Micronesia	2016	STEPS	Subnational	both	18-69	18-69	516	818	
2185	Moldova	2005	DHS	National	both		15-49		7076	
2186	Moldova	2013	Childhood Obesity Surveillance Initiative 3	National	both	7-8	7-8	1931	1751	
2187	Moldova	2013	STEPS	National	both	18-69	18-69	1712	2777	
2188	Moldova	2021	STEPS	National	both	18-69	18-69	1721	2252	
2189	Moldova	2022	Childhood Obesity Surveillance Initiative 6	National	both	7	7	1568	1465	
2190	Mongolia	1999	National Nutrition Survey	National	both	35-65	35-65	907	1317	
2191	Mongolia	2004	National Nutrition Survey	National	both	15-74	15-74	248	360	
2192	Mongolia	2005	STEPS	National	both	15-64	15-64	1669	1717	
2193	Mongolia	2009	STEPS	National	both	15-64	15-64	2197	3117	
2194	Mongolia	2010	National Nutrition Survey	National	both		7-11		557	
2195	Mongolia	2010	National Nutrition Survey	National	both		15-49		1475	
2196	Mongolia	2012-2013	Coping with Shocks in Mongolia Household Panel Survey	Subnational	both	5-6	5-6	55	51	
2197	Mongolia	2013	STEPS	National	both	15-64	15-64	2698	3167	
2198	Mongolia	2013	Global School-based Student Health Survey	National	both	13-17	13-17	2095	2360	
2199	Mongolia	2013-2014	Coping with Shocks in Mongolia Household Panel Survey	Subnational	both	5-7	5-7	108	85	
2200	Mongolia	2014-2015	Coping with Shocks in Mongolia Household Panel Survey	Subnational	both	5-8	5-8	192	184	
2201	Mongolia	2016	National Nutrition Survey (household sample)	National	both	15-49		1379		
2202	Mongolia	2016	National Nutrition Survey (school sample)	National	both	6-11	6-11	879	876	
2203	Mongolia	2019	STEPS	National	both	15-69	15-69	2926	3543	
2204	Mongolia	2023	Global School-based Student Health Survey	National	both	13-17	13-17	1400	1612	
2205	Montenegro	1978	Anthropometric Characteristics of Montenegrin Recruiters	National	both	17-28		86		1
2206	Montenegro	1979	Anthropometric Characteristics of Montenegrin Recruiters	National	both	17-28		7229		1
2207	Montenegro	1980	Anthropometric Characteristics of Montenegrin Recruiters	National	both	17-28		11208		
2208	Montenegro	1981	Anthropometric Characteristics of Montenegrin Recruiters	National	both	17-28		11372		
2209	Montenegro	1982	Anthropometric Characteristics of Montenegrin Recruiters	National	both	17-28		5502		
2210	Montenegro	1983	Anthropometric Characteristics of Montenegrin Recruiters	National	both	17-28		9448		
2211	Montenegro	1984	Anthropometric Characteristics of Montenegrin Recruiters	National	both	17-28		10320		
2212	Montenegro	1985	Anthropometric Characteristics of Montenegrin Recruiters	National	both	17-28		9722		
2213	Montenegro	1986	Anthropometric Characteristics of Montenegrin Recruiters	National	both	17-28		9961		
2214	Montenegro	1987	Anthropometric Characteristics of Montenegrin Recruiters	National	both	17-28		10230		
2215	Montenegro	1988	Anthropometric Characteristics of Montenegrin Recruiters	National	both	17-28		86		
2216	Montenegro	2015-2016	Childhood Obesity Surveillance Initiative 4	National	both	6-8	6-8	1802	1623	
2217	Montenegro	2016	Anthropometric parameters as an indicator of obesity at adolescents in Montenegro	National	both	14-18	14-18	678	771	
2218	Montenegro	2018-2019	Initiative for monitoring obesity of children aged 6 to 9 in Montenegro and Slovenia	National	both	6-9	6-9	1111	999	
2219	Montenegro	2019	Childhood Obesity Surveillance Initiative 5	National	both	6-8	6-8	1729	1626	
2220	Montenegro	2019	Initiative for monitoring obesity of children aged 11 to 12 in Montenegro	National	both	11-12	11-12	666	619	
2221	Montenegro	2019	Initiative for monitoring obesity of children aged 5 to 6 in Montenegro and Slovenia	National	both	5-6	5-6	231	214	
2222	Montenegro	2019	Body composition of high school students in Montenegro and its relationship with their eating habits	National	both	18-20	18-20	504	497	
2223	Montenegro	2020-2021	Initiative for monitoring obesity of school children aged 9 to 10 in Montenegro and Slovenia	National	both	9-10	9-10	694	666	
2224	Montenegro	2021-2022	Determining the differences in the levels of anthropometric characteristics of students in the first grades of elementary schools in Montenegro	Subnational	urban	6-8	6-8	397	346	
2225	Montenegro	2022	Determining the anthropometric characteristics of final grade elementary school students in Montenegro - Bijelo Polje	Community	both	14-15	14-15	15	9	
2226	Montenegro	2022	Determining the anthropometric characteristics of final grade elementary school students in Montenegro - Herceg Novi	Community	both	14-15	14-15	25	23	
2227	Montenegro	2022	Determining the anthropometric characteristics of final grade elementary school students in Montenegro - Niksic	Community	both	14-15	14-15	97	52	
2228	Montenegro	2022	Determining the anthropometric characteristics of final grade elementary school students in Montenegro - Podgorica	Community	both	14-15	14-15	66	59	
2229	Montenegro	2022	Childhood Obesity Surveillance Initiative 6	National	both	6-8	6-8	1661	1565	
2230	Montenegro	2022	Determining the differences in the levels of anthropometric characteristics of students in the Ninth grades in Montenegro	Subnational	urban	14-15	14-15	175	144	
2231	Montenegro	2023-2024	Determining the differences in the levels of anthropometric characteristics of students in the first grades of elementary schools in Montenegro	Subnational	urban	5-9	5-9	424	398	
2232	Montenegro	2024	Determining the differences in the levels of anthropometric characteristics of students in the first grades of elementary schools in Montenegro	Subnational	urban	5-8	5-8	308	305	
2233	Morocco	1992	DHS	National	both		20-49		2804	
2234	Morocco	2000	National Survey 2000	National	both	20+	20+	755	1047	
2235	Morocco	2003-2004	DHS	National	both		15-49		15944	

	Country	Study years	Survey/Study name/Citation	Level of representative-ness	Rural, urban, or both	Age range as in NCD-RisC database		Sample size		Note
						Male	Female	Male	Female	
2236	Morocco	2017	STEPS	National	both	18+	18+	1871	3390	
2237	Morocco	2019-2020	National Nutrition Survey	National	both	6-12	6-12	574	591	
2238	Morocco	2023	Global School-based Student Health Survey (Fes)	Community	urban	12-17	12-17	1556	1786	
2239	Mozambique	1997	DHS	National	both		20-49		2824	
2240	Mozambique	2000	Growth of adolescents in Mozambique	Community	urban	9-17	9-17	690	727	
2241	Mozambique	2003	DHS	National	both		15-49		10535	
2242	Mozambique	2005	STEPS	National	both	25-64	25-64	1276	1689	
2243	Mozambique	2010	Estudo do Estado Nutricional e da Dieta em Raparigas Adolescentes na Zambézia (ZANE)	Subnational	both		14-19		478	
2244	Mozambique	2011	DHS	National	both		15-49		12201	
2245	Mozambique	2014-2015	STEPS	National	both	15-64	15-64	1147	1684	
2246	Mozambique	2017-2018	Examining lifestyle behaviours and weight status of primary schoolchildren in Mozambique	Community	rural	9-11	9-11	146	158	
2247	Mozambique	2017-2018	Examining lifestyle behaviours and weight status of primary schoolchildren in Mozambique	Community	urban	9-11	9-11	140	170	
2248	Mozambique	2022-2023	DHS	National	both		15-49		3138	
2249	Mozambique	2024	STEPS	National	both	18-69	18-69	1576	2911	
2250	Myanmar	2003-2004	STEPS	Subnational	both	25-74	25-74	1990	2449	
2251	Myanmar	2009	STEPS	National	both	15-64	15-64	2826	4421	
2252	Myanmar	2011	Underweight prevalence among young adults from rural areas, Salin Township, Magwe Region	Community	rural	15-35	15-35	156	233	
2253	Myanmar	2013-2014	STEPS, Yangon	Subnational	both	25-74	25-74	745	740	
2254	Myanmar	2014	STEPS	National	both	25-64	25-64	2947	5444	
2255	Myanmar	2015-2016	DHS	National	both		15-49		12163	
2256	Myanmar	2024	STEPS	National	both	18-69	18-69	2365	5489	
2257	Namibia	1992	DHS	National	both		20-49		2062	
2258	Namibia	2005	STEPS	National	both	25-64	25-64	1390	1778	
2259	Namibia	2006-2007	DHS	National	both		15-49		8968	
2260	Namibia	2009	Okambilimbili Survey	Community	urban	5+	5+	962	1167	
2261	Namibia	2013	DHS	National	both		15-64		5111	
2262	Nauru	1982	Trends in the prevalence and incidence of non-insulin-dependent diabetes mellitus and impaired glucose tolerance	National	both	20+	20+	701	773	
2263	Nauru	1987	Trends in the prevalence and incidence of non-insulin-dependent diabetes mellitus and impaired glucose tolerance	National	both	20+	20+	555	667	
2264	Nauru	1994	Trends in the prevalence and incidence of non-insulin-dependent diabetes mellitus and impaired glucose tolerance	National	both	25+	25+	647	731	
2265	Nauru	2004	STEPS	National	both	15-64	15-64	1082	1149	
2266	Nauru	2006	STEPS	National	both	16-65	16-65	255	236	
2267	Nauru	2011	Global School-based Student Health Survey	National	both		13-17		259	
2268	Nauru	2015	STEPS	National	both	18-69	18-69	505	540	
2269	Nauru	2025	STEPS	National	both	18-69	18-69	438	458	22
2270	Nepal	1996	DHS	National	both		20-49		3068	
2271	Nepal	1997	Ohno et al., Asia Pac J Public Health 18(3):20-9, 2006	Community	rural	17-75	17-75	36	41	
2272	Nepal	2001	DHS	National	both		20-49		7216	
2273	Nepal	2003	STEPS	Subnational	both	25-64	25-64	1010	996	
2274	Nepal	2005	STEPS	Subnational	both	15-64	15-64	3634	3998	
2275	Nepal	2006	DHS	National	both		15-49		10117	
2276	Nepal	2006-2011	Early detection and management of Kidney disease, Hypertension, Diabetes and Cardiovascular disease (KHDC Nepal), Tarahara	Community	rural	18+	18+	1175	2350	
2277	Nepal	2006-2011	Early detection and management of Kidney disease, Hypertension, Diabetes and Cardiovascular disease (KHDC Nepal), Damak	Community	urban	18+	18+	1095	1576	
2278	Nepal	2006-2011	Early detection and management of Kidney disease, Hypertension, Diabetes and Cardiovascular disease (KHDC Nepal), Dharan	Community	urban	18+	18+	4130	6126	
2279	Nepal	2007-2008	STEPS	National	both	15-64	15-64	1889	2348	
2280	Nepal	2011	DHS	National	both		15-49		5848	
2281	Nepal	2012-2013	Obesity among women in a rural district of Nepal	Subnational	rural		20+		1365	
2282	Nepal	2012-2013	STEPS	National	both	15-69	15-69	1316	2753	
2283	Nepal	2015	Community based intervention for prevention and control of non-communicable diseases risk factors (CIPCON) baseline survey, Dhankuta	Subnational	rural	15-69	15-69	555	781	
2284	Nepal	2015	Community based intervention for prevention and control of non-communicable diseases risk factors (CIPCON) baseline survey, Ilam	Subnational	rural	15-69	15-69	546	721	
2285	Nepal	2015	COBIN WAVE 1	Community	both	24-65	24-65	972	1836	
2286	Nepal	2016	DHS	National	both	15-49	15-49	4035	6165	
2287	Nepal	2016	National Micronutrient Status Survey 2016	National	both	10-19	10-49	1021	3755	
2288	Nepal	2016-2018	The Population Based Prevalence of Selected Non-Communicable Diseases In Nepal	National	both	20+	20+	4907	7529	
2289	Nepal	2019	STEPS	National	both	15-69	15-69	1996	3521	
2290	Nepal	2022	DHS	National	both	15+	15-49	4225	12517	
2291	Netherlands	1985	INTERSALT	Community	urban	20-59	20-59	100	99	
2292	Netherlands	1985	Zutphen Elderly Study	Community	urban	65-85		886		
2293	Netherlands	1987-1991	Doetinchem Cohort Study	Subnational	urban	20-59	20-59	2995	3317	
2294	Netherlands	1989-1993	The Rotterdam Study, first subcohort	Community	urban	55+	55+	2807	4103	

	Country	Study years	Survey/Study name/Citation	Level of representative-ness	Rural, urban, or both	Age range as in NCD-RisC database		Sample size		Note
						Male	Female	Male	Female	
2295	Netherlands	1990	Zutphen Elderly Study	Community	urban	69-90		552		
2296	Netherlands	1992-1993	The Longitudinal Aging Study Amsterdam (LASA)	Subnational	both	55-85	55-85	1266	1308	26
2297	Netherlands	1993-1995	The Rotterdam Study, first subcohort	Community	urban	56+	56+	2214	3105	
2298	Netherlands	1993-1997	EPIC Bilthoven	Community	urban	20-59	20-59	9941	12021	
2299	Netherlands	1993-1997	EPIC Utrecht	Community	both		49-70		17335	
2300	Netherlands	1995-1996	The Longitudinal Aging Study Amsterdam (LASA)	Subnational	both	65-88	65-88	714	764	26
2301	Netherlands	1997-1999	The Rotterdam Study, first subcohort	Community	urban	61+	61+	1718	2361	
2302	Netherlands	1998-1999	The Longitudinal Aging Study Amsterdam (LASA)	Subnational	both	61-91	61-91	604	743	26
2303	Netherlands	1998-2001	Regenboog Project	National	both	12-89	12-89	2714	2643	
2304	Netherlands	2000-2001	The Rotterdam Study, second subcohort	Community	urban	55+	55+	1210	1468	
2305	Netherlands	2001-2002	The Longitudinal Aging Study Amsterdam (LASA)	Subnational	both	64-94	64-94	577	690	26
2306	Netherlands	2001-2003	Surinamese in the Netherlands: Study on Ethnicity and Health (SUNSET)	Community	urban	35-60	35-60	251	257	
2307	Netherlands	2002-2003	The Longitudinal Aging Study Amsterdam (LASA)	Subnational	both	54-65	54-65	431	482	26
2308	Netherlands	2002-2004	The Rotterdam Study, first subcohort	Community	urban	64+	64+	1206	1708	
2309	Netherlands	2003-2007	Doetinchem Cohort Study	Subnational	urban	36-75	36-75	2135	2368	
2310	Netherlands	2004-2005	The Rotterdam Study, second subcohort	Community	urban	58+	58+	964	1244	
2311	Netherlands	2004-2006	Prevention and Incidence of Asthma and Mite Allergy (PIAMA)	National	both	7-9	7-9	1110	1104	
2312	Netherlands	2005-2006	The Longitudinal Aging Study Amsterdam (LASA)	Subnational	both	57-97	57-97	789	958	26
2313	Netherlands	2006-2008	The Rotterdam Study, third subcohort	Community	urban	45+	45+	1547	2029	
2314	Netherlands	2008-2009	The Longitudinal Aging Study Amsterdam (LASA)	Subnational	both	60-100	60-100	642	789	26
2315	Netherlands	2008-2011	Prevention and Incidence of Asthma and Mite Allergy (PIAMA)	National	both	12-13	12-13	739	769	
2316	Netherlands	2008-2011	Amsterdam Born Children and their Development Study (ABCD)	Community	both	5-7	5-7	1540	1528	
2317	Netherlands	2009-2010	Measuring the Netherlands (NL de Maat)	Subnational	both	30-70	30-70	1781	2014	
2318	Netherlands	2009-2011	The Rotterdam Study, first subcohort	Community	urban	72+	72+	690	1006	
2319	Netherlands	2010	EuropeaN Energy balance Research to prevent excessive weight Gain among Youth - The ENERGY-project	National	both	10-12	10-12	404	399	
2320	Netherlands	2011-2012	The Rotterdam Study, second subcohort	Community	urban	65+	65+	735	934	
2321	Netherlands	2011-2012	The Longitudinal Aging Study Amsterdam (LASA)	Subnational	both	63-104	63-104	532	653	26
2322	Netherlands	2011-2013	GECKO Drenthe Onderzoek	Subnational	rural	5-7	5-7	1139	1133	
2323	Netherlands	2011-2015	Healthy Life in an Urban Setting (HELIUS)	Community	urban	18-71	18-71	2088	2473	
2324	Netherlands	2012-2013	The Longitudinal Aging Study Amsterdam (LASA)	Subnational	both	55-65	55-65	426	448	26, 27
2325	Netherlands	2012-2014	Prevention and Incidence of Asthma and Mite Allergy (PIAMA)	National	both	15-17	15-17	386	414	
2326	Netherlands	2012-2014	The Rotterdam Study, third subcohort	Community	urban	52+	52+	1256	1639	
2327	Netherlands	2012-2016	Amsterdam Born Children and their Development Study (ABCD)	Community	both	9-12	9-12	1120	1136	
2328	Netherlands	2015-2016	The Longitudinal Aging Study Amsterdam (LASA)	Subnational	both	58+	58+	759	857	26
2329	Netherlands	2016-2018	GECKO Drenthe Onderzoek	Subnational	rural	8-12	8-12	1083	1114	
2330	Netherlands	2018-2019	The Longitudinal Aging Study Amsterdam (LASA)	Subnational	both	61+	61+	568	618	
2331	New Zealand	1982	MONICA, Auckland	Community	urban	35-64	35-64	1019	568	
2332	New Zealand	1989	The Life in New Zealand Survey	National	both	15+	15+	1418	1571	
2333	New Zealand	1990-1993	Williams, N Z Med J 113(1114):308-11, 2000	Community	both	18-21	18-21	932	859	
2334	New Zealand	1993-1994	MONICA, Auckland	Community	urban	35-64	35-64	723	674	
2335	New Zealand	1996-1997	National Nutrition Survey	National	both	15+	15+	1857	2522	
2336	New Zealand	2002	National Children's Nutrition Survey	National	both	5-14	5-14	1564	1485	
2337	New Zealand	2002-2003	New Zealand Health Survey	National	both	15+	15+	4594	6729	
2338	New Zealand	2006-2007	New Zealand Health Survey	National	both	5+	5+	6766	8029	
2339	New Zealand	2008-2009	New Zealand Adult Nutrition Survey	National	both	15+	15+	2003	2500	
2340	New Zealand	2011-2012	New Zealand Health Survey	National	both	5+	5+	5782	7220	
2341	New Zealand	2012-2013	New Zealand Health Survey	National	both	5+	5+	6412	7932	
2342	New Zealand	2013-2014	New Zealand Health Survey	National	both	5+	5+	6966	8309	
2343	New Zealand	2014-2015	New Zealand Health Survey	National	both	5+	5+	7124	8437	
2344	New Zealand	2015-2016	New Zealand Health Survey	National	both	5+	5+	7204	8401	
2345	New Zealand	2016-2017	New Zealand Health Survey	National	both	5+	5+	6944	8275	
2346	New Zealand	2017-2018	New Zealand Health Survey	National	both	5+	5+	7116	8399	
2347	New Zealand	2018-2019	New Zealand Health Survey	National	both	5+	5+	6809	8461	
2348	New Zealand	2019-2020	New Zealand Health Survey	National	both	5+	5+	4842	5975	
2349	New Zealand	2020-2021	New Zealand Health Survey	National	both	5+	5+	4595	5488	
2350	Nicaragua	1997-1998	DHS	National	both		15-49		12257	
2351	Nicaragua	2001	DHS	National	both		15-49		11940	
2352	Nicaragua	2003-2004	CAMDI	Community	urban	20+	20+	773	916	
2353	Nicaragua	2003-2005	Sistema Integrado de Vigilancia de Intervenciones Nutricionales (SIVIN)	National	both		15-50		1115	
2354	Nicaragua	2006-2007	Encuesta Nicaraguense de Demografia y Salud	National	both		15-49		13216	
2355	Nicaragua	2011-2012	Encuesta Nicaraguense de Demografia y Salud	National	both		15-49		14318	
2356	Niger	1992	DHS	National	both		20-49		2993	
2357	Niger	1998	DHS	National	both		20-49		2958	

	Country	Study years	Survey/Study name/Citation	Level of representative-ness	Rural, urban, or both	Age range as in NCD-RisC database		Sample size		Note
						Male	Female	Male	Female	
2358	Niger	2006	DHS	National	both		15-49		4151	
2359	Niger	2007	STEPS	National	both	15-64	15-64	1430	1215	
2360	Niger	2012	DHS	National	both		15-49		4429	
2361	Niger	2021	STEPS	National	both	18-69	18-69	2270	3059	
2362	Nigeria	1990	Non-communicable diseases National Survey	National	rural	15+	15+	3616	3678	
2363	Nigeria	1990	Non-communicable diseases National Survey	National	urban	15+	15+	1612	1642	
2364	Nigeria	1991-1994	Cooper et al., Am J Public Health 87(2):160-68, 1997	Community	both	20-100	20-100	910	1080	
2365	Nigeria	1999	DHS	National	both		20-49		2004	
2366	Nigeria	1999-2009	Prostate cancer dietary risk factors study	Subnational	both	35+		627		
2367	Nigeria	2003	DHS	National	both		15-49		6605	
2368	Nigeria	2006	Clustering of cardiovascular disease risk-factors in semiurban population in Northern Nigeria	Community	urban	15+	15+	171	249	
2369	Nigeria	2006	Senbanjo et al., West Afr J Med 30(6):425-31, 2011	Community	urban	5-19	5-19	296	274	
2370	Nigeria	2007	Ibadan Study of Ageing	Subnational	both	60+	60+	642	914	
2371	Nigeria	2007	Southeast Nigeria kidney disease study	Community	rural	25-64	25-64	168	442	
2372	Nigeria	2008	DHS	National	both		15-49		28973	
2373	Nigeria	2008	Ibadan Study of Ageing	Subnational	both	61+	61+	453	656	
2374	Nigeria	2008-2009	Prevalence of Overweight and Obesity in Maiduguri, North-Eastern Nigeria	Community	urban	15+	15+	1113	535	
2375	Nigeria	2009	Community Health Plan - Kwara Central Survey	Community	rural	5+	5+	2264	2354	
2376	Nigeria	2009	Ibadan Study of Ageing	Subnational	both	62+	62+	420	619	
2377	Nigeria	2009-2011	Anthropometric indices in Calabar	Community	urban	15-79	15-79	383	333	
2378	Nigeria	2010	Nnewi obesity study	Community	urban	19-85	19-85	774	746	
2379	Nigeria	2010-2011	Nigeria built environment	Subnational	urban	20-65	20-65	725	1093	
2380	Nigeria	2011	Community Health Plan - Kwara Central Survey	Community	rural	5+	5+	791	853	
2381	Nigeria	2012	Neighbourhood walkability and sedentary lifestyle	Community	urban	60+	60+	212	141	
2382	Nigeria	2013	DHS	National	both		15-49		33943	
2383	Nigeria	2013	Community Health Plan - Kwara Central Survey	Community	rural	5+	5+	714	754	
2384	Nigeria	2017	ARISE Network Adolescent Health Study (Ibadan)	Community	urban	10-19	10-19	336	407	
2385	Nigeria	2017-2019	Removing the Mask on Hypertension (REMAH)	National	both	18+	18+	1806	2353	
2386	Nigeria	2018	Hypertension Prevalence, Awareness, Treatment and Control in Rural Area, Nigeria	Community	rural	18+	18+	189	202	
2387	Niue	2010	Global School-based Student Health Survey	National	both	13-17	13-17	63	38	
2388	Niue	2011	STEPS	National	both	15+	15+	407	478	
2389	Niue	2019	Global School-based Student Health Survey	National	both	12-17	12-17	51	64	
2390	North Macedonia	1999	Multiple Indicator Cluster Survey	National	both		15-45		1038	
2391	North Macedonia	2009	Annual assessment of nutritional status of school children aged 7 years	National	both	7	7	1087	982	
2392	North Macedonia	2010	Childhood Obesity Surveillance Initiative 2	National	both	7	7	1427	1311	
2393	North Macedonia	2010-2011	The Macedonia National Nutrition Survey 2011	National	both		15-49		4098	
2394	North Macedonia	2013	Childhood Obesity Surveillance Initiative 3	National	both	6-7	6-7	1655	1511	
2395	North Macedonia	2015-2016	Childhood Obesity Surveillance Initiative 4	National	both	7-8	7-8	1809	1727	
2396	North Macedonia	2019	Childhood Obesity Surveillance Initiative 5	National	both	7-8	7-8	1579	1611	
2397	North Macedonia	2022	Childhood Obesity Surveillance Initiative 6	National	both	6-7	6-7	1383	1364	
2398	Norway	1979-1980	The Tromsø Study: Tromsø 2	Community	both	20-54	20-49	8312	7823	
2399	Norway	1984-1986	HUNT1 study	Subnational	rural	20+	20+	36514	37811	
2400	Norway	1986-1987	The Tromsø Study: Tromsø 3	Community	both	20-61	20-56	10374	9804	
2401	Norway	1992-1993	The Hordaland Homocysteine Study 1925-1927 birth cohort	Subnational	both	65-67	65-67	2123	2630	
2402	Norway	1992-1993	The Hordaland Homocysteine Study 1928-1949 birth cohort	Subnational	urban	43-64	43-64	335	348	
2403	Norway	1992-1993	The Hordaland Homocysteine Study 1950-1952 birth cohort	Subnational	both	40-42	40-42	6103	6475	
2404	Norway	1994-1995	The Tromsø Study: Tromsø 4	Community	both	25+	25+	12782	13836	
2405	Norway	1995-1997	YoungHUNT1 Study	Subnational	rural	13-19	13-19	4144	4202	
2406	Norway	1995-1997	HUNT2 study	Subnational	rural	20+	20+	30184	34058	
2407	Norway	1997-1998	The Hordaland Health Study (HUSK) 1925-1927 birth cohort	Subnational	both	70-74	70-74	1467	1848	
2408	Norway	1997-1998	The Hordaland Health Study (HUSK) 1950-1951 birth cohort	Subnational	both	46-49	46-49	1658	2058	
2409	Norway	1997-1998	The Hordaland Health Study (HUSK) 1953-1957 birth cohort	Subnational	both	39-46	39-46	8526	9878	
2410	Norway	1999-2000	European Youth Heart Study	Community	urban	9-15	9-15	364	369	
2411	Norway	2000-2001	YoungHUNT2 Study	Subnational	rural	13-19	13-19	769	902	
2412	Norway	2000-2003	The Oslo cohort (HUBRO), the Oppland and Hedmark cohort (OPPHED), and the Troms and Finnmark cohort (TROFINN) of Cohort of Norway (CONOR)	Subnational	both	30-76	30-76	16825	20592	
2413	Norway	2001-2002	The Tromsø Study: Tromsø 5, Tromsø Study Panel	Community	both	30-89	30-89	2525	3579	
2414	Norway	2005-2006	Physical Activity among Norwegian Children and Adolescents	National	both	8-16	8-16	1186	1055	
2415	Norway	2006-2008	YoungHUNT3 Study	Subnational	rural	13-18	13-18	3750	3700	
2416	Norway	2006-2008	HUNT3 study	Subnational	rural	19+	19+	22856	27553	
2417	Norway	2007-2008	The Tromsø Study: Tromsø 6	Community	both	30-87	30-87	6048	6889	
2418	Norway	2008	Childhood Obesity Surveillance Initiative 1	National	both	8	8	1435	1399	
2419	Norway	2009-2010	Trondheim Early Secure Study (TESS)	Community	urban	6	6	328	322	

	Country	Study years	Survey/Study name/Citation	Level of representative-ness	Rural, urban, or both	Age range as in NCD-RisC database		Sample size		Note
						Male	Female	Male	Female	
2420	Norway	2010	Childhood Obesity Surveillance Initiative 2	National	both	8	8	1335	1286	
2421	Norway	2010	EuropeaN Energy balance Research to prevent excessive weight Gain among Youth - The ENERGY-project	Subnational	urban	10-13	10-13	458	491	
2422	Norway	2011-2012	Trondheim Early Secure Study (TESS)	Community	urban	8	8	331	344	
2423	Norway	2012	Childhood Obesity Surveillance Initiative 3	National	both	8	8	1492	1381	
2424	Norway	2013-2014	Trondheim Early Secure Study (TESS)	Community	urban	10	10	332	360	
2425	Norway	2015-2016	Childhood Obesity Surveillance Initiative 4	National	both	7-8	7-8	1690	1645	
2426	Norway	2015-2016	Trondheim Early Secure Study (TESS)	Community	urban	12	12	310	333	
2427	Norway	2015-2016	The Tromsø Study: Tromsø 7	Community	both	40+	40+	9979	11006	
2428	Norway	2017-2018	Trondheim Early Secure Study (TESS)	Community	urban	14	14	289	322	
2429	Norway	2017-2019	YoungHUNT4 Study	Community	rural	13-18	13-18	3452	3747	
2430	Norway	2017-2019	HUNT4 study	Community	rural	19+	19+	24463	29683	
2431	Norway	2018-2020	The Hordaland Health Study (HUSK) 1950-1951 birth cohort	Subnational	both	67-70	67-70	986	1192	
2432	Norway	2019-2021	Trondheim Early Secure Study (TESS)	Community	urban	16	16	287	341	
2433	Oman	1991	The 1991 National Diabetes Survey of Oman	National	both	20+	20+	2024	2868	
2434	Oman	2000	Oman National Health survey	National	both	20+	20+	3069	3331	
2435	Oman	2001	Nizwa Healthy Lifestyle Project	Community	urban	20+	20+	707	726	
2436	Oman	2006	STEPS	Community	urban	20-59	20-59	540	732	
2437	Oman	2008	Gulf Cooperation Council World Health Survey	National	both	18+	18+	2389	2112	
2438	Oman	2010	Global School-based Student Health Survey	National	both	13-17	13-17	251	300	
2439	Oman	2015	Global School-based Student Health Survey	National	both	13-17	13-17	1330	1551	
2440	Oman	2017	STEPS	National	both	18+	18+	3334	2948	
2441	Pakistan	1990-1994	National Health Survey of Pakistan 1990-1994	National	both	5+	5+	7110	7405	
2442	Pakistan	1990-1994	MHS	Community	urban	18+	18+	432	478	
2443	Pakistan	1999	Shah et al., Trop Med Int Health 9(4):526-32, 2004	Community	both	18+	18+	1391	2754	
2444	Pakistan	2004-2005	COBRA-1	Community	urban	40+	40+	1500	1635	
2445	Pakistan	2005	STEPS	National	both	25-65	25-65	787	1071	
2446	Pakistan	2011	National Nutrition Survey	National	both	5-49	5-49	21461	48503	
2447	Pakistan	2012-2013	DHS	National	both		20-49		3968	
2448	Pakistan	2014	STEPS	Subnational	both	18-69	18-69	2964	3674	
2449	Pakistan	2016-2017	National Diabetes Survey of Pakistan	National	both	20+	20+	3771	4647	
2450	Pakistan	2017-2018	DHS	National	both		15-49		3637	
2451	Pakistan	2018-2019	National Nutrition Survey	National	both	5-19	5-49	11888	127307	
2452	Palau	2010	School Health Screening	National	both	5-17	5-17	537	511	
2453	Palau	2011	School Health Screening	National	both	5-17	5-17	613	599	
2454	Palau	2011-2013	STEPS	National	both	25-64	25-64	1031	1124	
2455	Palau	2012	School Health Screening	National	both	5-17	5-17	548	596	
2456	Palau	2013	School Health Screening	National	both	5-17	5-17	570	576	
2457	Palau	2014	School Health Screening	National	both	5-17	5-17	576	558	
2458	Palau	2015	School Health Screening	National	both	5-17	5-17	522	565	
2459	Palau	2016	School Health Screening	National	both	5-17	5-17	569	563	
2460	Palau	2016	STEPS	National	both	18+	18+	713	711	
2461	Palau	2017	School Health Screening	National	both	5-17	5-17	484	522	
2462	Palau	2018	School Health Screening	National	both	5-17	5-17	598	554	
2463	Palau	2019	School Health Screening	National	both	5-17	5-17	504	561	
2464	Palau	2020	School Health Screening	National	both	5-17	5-17	591	568	
2465	Palau	2021	School Health Screening	National	both	5-17	5-17	600	598	
2466	Palau	2022	School Health Screening	National	both	5-17	5-17	525	515	
2467	Palau	2023	Adult Hybrid Health Assessment Survey 2023	National	both	18+	18+	1011	879	
2468	Panama	2003	Encuesta de Niveles de Vida	National	both	5+	5+	10808	11133	
2469	Panama	2010-2011	Prevalencia de factores de riesgo asociados a enfermedad cardiovascular 2010-2011	Subnational	both	18+	18+	1067	2469	
2470	Panama	2018	Global School-based Student Health Survey	National	both	13-17	13-17	1121	1384	
2471	Panama	2019	Encuesta Nacional de Salud de Panama (ENSPA)	National	both	15+	15+	4288	10851	
2472	Papua New Guinea	1985-1986	INTERSALT	Community	rural	20-59	20-59	88	74	
2473	Papua New Guinea	1991	Dowse et al., Med J Aust 160:767-74, 1994	Subnational	both	25-88	25-88	836	1012	
2474	Paraguay	2011	Primera Encuesta Nacional de Factores de Riesgo de Enfermedades No Transmisibles en la Poblacion General	National	both	15-75	15-75	931	1574	
2475	Paraguay	2017	Global School-based Student Health Survey	National	both	12-16	12-16	1119	1231	
2476	Paraguay	2022	Segunda Encuesta Nacional de Factores de Riesgo de Enfermedades No Transmisibles, Paraguay 2022	National	both	18-69	18-69	2003	2611	
2477	Peru	1991-1992	DHS	National	both		15-49		4887	
2478	Peru	1996	DHS	National	both		20-49		10125	
2479	Peru	2000	DHS	National	both		15-49		25508	
2480	Peru	2003	Factores de Riesgo de Enfermedades No Transmisibles	Community	urban	16+	16+	327	503	
2481	Peru	2004	Factores de Riesgo de Enfermedades No Transmisibles	Community	urban	15+	15+	218	445	

	Country	Study years	Survey/Study name/Citation	Level of representative-ness	Rural, urban, or both	Age range as in NCD-RisC database		Sample size		Note
						Male	Female	Male	Female	
2482	Peru	2004-2005	Encuesta Nacional de Indicadores Nutricionales, Bioquímicos, Socioeconómicos y Culturales Relacionados con las Enfermedades Crónicas Degenerativas	National	both	20+	20+	2087	2095	
2483	Peru	2004-2005	CArdiovascular Risk factors Multiple Evaluation in Latin America (CARMELA)	Community	urban	25-64	25-64	769	876	
2484	Peru	2004-2006	DHS	National	both		15-49		5798	
2485	Peru	2005	Factores de Riesgo de Enfermedades No Transmisibles	Community	urban	15+	15+	209	550	
2486	Peru	2006	Factores de Riesgo de Enfermedades No Transmisibles	Community	urban	15+	15+	662	1101	
2487	Peru	2007-2008	DHS	National	both		15-49		20918	
2488	Peru	2007-2008	Monitoreo de Indicadores Nutricionales en la ENAHO 2007-2008	National	both	5+	5+	15041	16282	
2489	Peru	2007-2008	PERU MIGRANT Study	Community	both	30+	30+	464	522	
2490	Peru	2007-2010	Monitoreo Nacional de Indicadores Nutricionales	National	both		12-49		3804	
2491	Peru	2009	DHS	National	both		15-49		23034	
2492	Peru	2009-2011	Monitoreo de Indicadores Nutricionales en la ENAHO 2009-2010	National	both	5+	5+	27753	31269	
2493	Peru	2009-2012	CRONICAS Cohort Study	Subnational	both	35+	35+	1557	1660	
2494	Peru	2010	DHS	National	both		15-49		22425	
2495	Peru	2010	Global School-based Student Health Survey	National	both	13-17	13-17	1267	1306	
2496	Peru	2010-2013	CRONICAS Cohort Study	Subnational	both	35+	35+	1379	1468	
2497	Peru	2011	DHS	National	both		15-49		22215	
2498	Peru	2011-2012	Monitoreo de Indicadores Nutricionales en la ENAHO 2011	National	both	5+	5+	7424	8424	
2499	Peru	2012	DHS	National	both		15-49		23724	
2500	Peru	2012-2013	PERU MIGRANT Study	Community	both	35+	35+	339	427	
2501	Peru	2013	DHS	National	both	15+	15+	2932	23784	
2502	Peru	2013	Clinical functional and sociofamilial profiles of the elderly from a community in a district of Lima, Peru	Community	urban	60+	60+	185	309	
2503	Peru	2013-2014	CRONICAS Cohort Study	Subnational	both	36+	36+	1292	1361	
2504	Peru	2014	DHS	National	both	15+	15+	12670	28554	
2505	Peru	2014	Launching a salt substitute to reduce blood pressure at the population level: a cluster randomized stepped wedge trial in Peru	Community	both	18+	18+	1149	1166	
2506	Peru	2014-2015	Latin American Study of Nutrition and Health (ELANS)	National	urban	15-65	15-65	603	627	
2507	Peru	2015	DHS	National	both	15+	15+	14744	38415	
2508	Peru	2015-2016	PERU MIGRANT Study	Community	both	38+	38+	324	414	
2509	Peru	2016	DHS	National	both	15+	15+	14035	35998	
2510	Peru	2016-2017	Screening of T2DM	Community	urban	30-70	30-70	798	809	
2511	Peru	2017	DHS	National	both	15+	15+	14339	36238	
2512	Peru	2017-2018	Disadvantaged Populations eGFR Epidemiology Study	Subnational	both	18+	18+	339	406	
2513	Peru	2017-2018	Disadvantaged Populations eGFR Epidemiology Study	Community	both	18+	18+	340	406	
2514	Peru	2017-2018	Vigilancia Alimentario Nutricional por Etapas de Vida (VIANEV) 2017-2018	National	both	18-59	18-59	465	618	
2515	Peru	2018	DHS	National	both	15+	15+	14599	38329	
2516	Peru	2019	DHS	National	both	15+	15+	14198	36732	
2517	Peru	2020	DHS	National	both	15+	15+	9922	25157	
2518	Peru	2021	DHS	National	both	15+	15+	13463	35465	
2519	Peru	2022	DHS	National	both	15+	15+	13587	34946	
2520	Peru	2023	DHS	National	both	15+	12+	13389	38519	
2521	Peru	2024	DHS	National	both	15+	12+	12917	37427	
2522	Philippines	1983-1984	Cebu Longitudinal Health and Nutrition Survey Baseline 2-Month Follow-up	Community	both		15-50		2866	
2523	Philippines	1983-1984	Cebu Longitudinal Health and Nutrition Survey Baseline 4-Month Follow-up	Community	both		15-50		2728	
2524	Philippines	1983-1984	Cebu Longitudinal Health and Nutrition Survey Baseline 6-Month Follow-up	Community	both		15-50		2601	
2525	Philippines	1984-1985	Cebu Longitudinal Health and Nutrition Survey Baseline 8-Month Follow-up	Community	both		15-50		2473	
2526	Philippines	1984-1985	Cebu Longitudinal Health and Nutrition Survey Baseline 10-Month Follow-up	Community	both		15-50		2348	
2527	Philippines	1984-1985	Cebu Longitudinal Health and Nutrition Survey Baseline 12-Month Follow-up	Community	both		15-50		2263	
2528	Philippines	1984-1985	Cebu Longitudinal Health and Nutrition Survey Baseline 14-Month Follow-up	Community	both		15-50		2193	
2529	Philippines	1984-1985	Cebu Longitudinal Health and Nutrition Survey Baseline 16-Month Follow-up	Community	both		15-50		2129	
2530	Philippines	1984-1985	Cebu Longitudinal Health and Nutrition Survey Baseline 18-Month Follow-up	Community	both		15-50		2079	
2531	Philippines	1985-1986	Cebu Longitudinal Health and Nutrition Survey Baseline 20-Month Follow-up	Community	both		15-50		2047	
2532	Philippines	1985-1986	Cebu Longitudinal Health and Nutrition Survey Baseline 22-Month Follow-up	Community	both		15-50		2017	
2533	Philippines	1985-1986	Cebu Longitudinal Health and Nutrition Survey Baseline 24-Month Follow-up	Community	both		15-50		2022	
2534	Philippines	1988	INCLEN	Community	rural	35-65		274		
2535	Philippines	1991-1992	Cebu Longitudinal Health and Nutrition Survey 1991 Child Follow-up	Community	both	8	8	1202	1076	
2536	Philippines	1991-1992	Cebu Longitudinal Health and Nutrition Survey 1991 Mother Follow-up	Community	both		22-55		2195	
2537	Philippines	1993	Philippines National Safe Motherhood Survey	National	both		15-49		7308	
2538	Philippines	1993	4th National Nutrition Survey	National	both	20-70	20-70	4383	4754	
2539	Philippines	1993	National Safe Motherhood Survey	National	both		15-49		7181	
2540	Philippines	1994-1995	Cebu Longitudinal Health and Nutrition Survey 1994-1995 Mother Follow-up	Community	both		15-59		2692	
2541	Philippines	1998	5th National Nutrition Survey	National	both	20-60	20-60	1323	1340	
2542	Philippines	1998-1999	Cebu Longitudinal Health and Nutrition Survey 1998-1999 Child Follow-up	Community	both	14-16	14-16	1102	999	
2543	Philippines	1998-1999	Cebu Longitudinal Health and Nutrition Survey 1998-1999 Mother Follow-up	Community	both		15-59		1911	

	Country	Study years	Survey/Study name/Citation	Level of representative-ness	Rural, urban, or both	Age range as in NCD-RisC database		Sample size		Note
						Male	Female	Male	Female	
2544	Philippines	2002	Cebu Longitudinal Health and Nutrition Survey 2002 Child Follow-up	Community	both	17-19	17-19	1087	907	
2545	Philippines	2002	Cebu Longitudinal Health and Nutrition Survey 2002 Mother Follow-up	Community	both		32-66		2080	
2546	Philippines	2003	Global School-based Student Health Survey	National	both		13		291	
2547	Philippines	2003	6th National Nutrition Survey	National	both	5+	5+	10686	11131	
2548	Philippines	2005	Cebu Longitudinal Health and Nutrition Survey 2005 Child Follow-up	Community	both	20-22	20-22	1006	831	
2549	Philippines	2005	Cebu Longitudinal Health and Nutrition Survey 2005 Mother Follow-up	Community	both		35-69		2001	
2550	Philippines	2007	Global School-based Student Health Survey	National	both		13		254	
2551	Philippines	2007	Cebu Longitudinal Health and Nutrition Survey 2007 Child Follow-up	Community	both	23-24	23-24	937	751	
2552	Philippines	2007	Cebu Longitudinal Health and Nutrition Survey 2007 Mother Follow-up	Community	both		38-71		1925	
2553	Philippines	2008	7th National Nutrition Survey	National	both	5+	5+	64001	63616	
2554	Philippines	2009	Cebu Longitudinal Health and Nutrition Survey 2009 Child Follow-up	Community	both	24-26	24-26	864	718	
2555	Philippines	2011	Global School-based Student Health Survey	National	both		13		540	
2556	Philippines	2011	2011 Updating of Nutritional Status of Filipino Children and Other Population Groups	National	both	5+	5+	63654	68523	
2557	Philippines	2013-2014	8th National Nutrition Survey	National	both	5+	5+	57432	61620	
2558	Philippines	2015	Global School-based Student Health Survey	National	both		13-17		3730	
2559	Philippines	2015	2015 Updating of Nutritional Status of Filipino Children and Other Population Groups	National	both	5+	5+	69309	73250	
2560	Philippines	2018-2021	Philippine Expanded National Nutrition Survey	National	both	5+	5+	132037	144383	
2561	Philippines	2019	Global School-based Student Health Survey	National	both	12-16	12-16	3571	4286	
2562	Poland	1983-1984	MONICA, Tarnobrzeg Voivodship	Community	rural	35-64	35-64	1236	1441	
2563	Poland	1983-1985	MONICA, Warsaw	Community	urban	35-64	35-64	1297	1327	
2564	Poland	1986	Poland Conscripts 10% Sample Cohort	National	both	18-19		29421		
2565	Poland	1986	INTERSALT, Krakow	Community	urban	20-59	20-59	100	100	
2566	Poland	1986	INTERSALT, Warsaw	Community	urban	20-59	20-59	100	100	
2567	Poland	1987-1988	MONICA, Tarnobrzeg Voivodship	Community	rural	35-64	35-64	616	672	
2568	Poland	1988	Fourth National Survey	Subnational	both	7-19	7-19	12345	10371	
2569	Poland	1988-1989	MONICA, Warsaw	Community	urban	35-64	35-64	705	713	
2570	Poland	1989-1990	Polish Program CINDI (CINDI Lodz 1989-1990)	Community	urban	25-64	25-64	831	957	
2571	Poland	1992-1993	MONICA, Tarnobrzeg Voivodship	Community	rural	35-64	35-64	618	692	
2572	Poland	1993	MONICA, Warsaw	Community	urban	35-64	35-64	751	763	
2573	Poland	1995	Poland Conscripts 10% Sample Cohort	National	both	18-19		31043		
2574	Poland	1995-1996	Polish Program CINDI (CINDI Lodz 1995)	Community	urban	17-64	17-64	997	1459	
2575	Poland	1997	Wroclaw survey in adolescents	Community	both	12-17	12-17	2198	2141	
2576	Poland	2000	The health status, risk factors of chronic diseases and health behaviors of residents of Torun (CINDI Torun 2000)	Community	urban	16-83	16-83	989	1054	
2577	Poland	2000	Binkowska-Bury et al., Neuro Endocrinol Lett 34(8):814-20, 2013	Subnational	both	19-20		3003		
2578	Poland	2000-2001	Household Food Consumption and Anthropometric Survey	National	both	5+	5+	1766	2107	
2579	Poland	2001	Poland Conscripts 10% Sample Cohort	National	both	18-19		31213		
2580	Poland	2001	Binkowska-Bury et al., Neuro Endocrinol Lett 34(8):814-20, 2013	Subnational	both	19-20		3420		
2581	Poland	2001-2002	Young Men Cardiovascular Association Study	Community	urban	16+		1156		28
2582	Poland	2001-2002	The health status, risk factors of chronic diseases and health behaviors of residents of Lodz (CINDI Lodz 2001)	Community	urban	18-64	18-64	1000	840	
2583	Poland	2002	The health status, risk factors of chronic diseases and health behaviors of residents of Lodz - seniors (CINDI Lodz 2002)	Community	urban	65+	65+	285	532	
2584	Poland	2002	NATPOL	National	both	18+	18+	1018	1301	
2585	Poland	2002	Binkowska-Bury et al., Neuro Endocrinol Lett 34(8):814-20, 2013	Subnational	both	19-20		3544		
2586	Poland	2002-2005	Health, Alcohol and Psychosocial Factors In Eastern Europe	Community	urban	45-70	45-70	4502	4752	
2587	Poland	2003	The European Male Ageing Study	Community	both	40+		406		
2588	Poland	2003	Binkowska-Bury et al., Neuro Endocrinol Lett 34(8):814-20, 2013	Subnational	both	19-20		3633		
2589	Poland	2003-2005	National Multicenter Health Survey in Poland. Project WOBASZ	National	both	20-74	20-74	6245	6910	
2590	Poland	2003-2006	Mogielica Human Ecology Study Site	Community	rural	18+	18+	119	321	
2591	Poland	2004	LIPIDOGRAM2004 Study - National epidemiological study of lipid disorders and selected risk factors of cardiovascular disease in primary health care in Poland	National	both	30+	30+	6673	9920	
2592	Poland	2004	Binkowska-Bury et al., Neuro Endocrinol Lett 34(8):814-20, 2013	Subnational	both	19-20		3538		
2593	Poland	2005	Binkowska-Bury et al., Neuro Endocrinol Lett 34(8):814-20, 2013	Subnational	both	19-20		3308		
2594	Poland	2006	The health, risk factors for chronic diseases, attitudes and behaviors of health residents of Torun (CINDI Torun 2006)	Community	urban	15-65	15-65	790	1147	
2595	Poland	2006	LIPIDOGRAM2006 Study - National epidemiological study of lipid disorders and selected risk factors of cardiovascular disease in primary health care in Poland	National	both	32+	32+	6441	10640	
2596	Poland	2006	Binkowska-Bury et al., Neuro Endocrinol Lett 34(8):814-20, 2013	Subnational	both	19-20		3701		
2597	Poland	2006-2007	National Multicenter Health Survey in Poland. Project WOBASZ Senior	National	both	75+	75+	541	533	
2598	Poland	2007	Binkowska-Bury et al., Neuro Endocrinol Lett 34(8):814-20, 2013	Subnational	both	19-20		3612		
2599	Poland	2007-2009	Elaboration of the reference range of arterial blood pressure for the population of children and adolescents in Poland - PL0080 OLAF - Primary	National	both	6-16	6-16	6497	6603	
2600	Poland	2007-2009	Elaboration of the reference range of arterial blood pressure for the population of children and adolescents in Poland - PL0080 OLAF - Secondary	National	both	16-18	16-18	1852	2520	
2601	Poland	2007-2010	Mogielica Human Ecology Study Site	Community	rural	18+	18+	133	290	
2602	Poland	2007-2011	Medical, psychological and socioeconomic aspects of aging in Poland	National	both	55+	55+	2682	2507	

	Country	Study years	Survey/Study name/Citation	Level of representative-ness	Rural, urban, or both	Age range as in NCD-RisC database		Sample size		Note
						Male	Female	Male	Female	
2603	Poland	2008	The European Male Ageing Study	Community	both	45+		310		
2604	Poland	2008	Binkowska-Bury et al., Neuro Endocrinol Lett 34(8):814-20, 2013	Subnational	both	19-20		3435		
2605	Poland	2009	Binkowska-Bury et al., Neuro Endocrinol Lett 34(8):814-20, 2013	Subnational	both	19-20		3405		
2606	Poland	2009-2010	Poland Conscripts 10% Sample Cohort	National	both	18-19		9208		
2607	Poland	2010	Binkowska-Bury et al., Neuro Endocrinol Lett 34(8):814-20, 2013	Subnational	both	19-20		3317		
2608	Poland	2010-2012	Blood pressure references for Polish preschool children - the OLA study	National	both	5-6	5-6	926	904	
2609	Poland	2011	NATPOL	National	both	18-79	18-79	1158	1235	
2610	Poland	2011-2014	Mogielica Human Ecology Study Site	Community	rural	18+	18+	142	418	
2611	Poland	2012-2013	Fifth National Survey	Subnational	both	6-19	6-19	3091	2686	
2612	Poland	2013-2014	National Multicenter Health Survey in Poland. Project WOBASZ II	National	both	20+	20+	2626	3198	
2613	Poland	2014	Prevalence of risk factors for obesity and hypertension among Polish children and adolescents	Subnational	both	7-18	7-18	284	283	
2614	Poland	2014-2017	The impact of physical activity and selected perinatal risk factors on the occurrence of overweight and obesity and hypertension in children	Subnational	both	5-15	5-15	516	454	
2615	Poland	2015-2016	Childhood Obesity Surveillance Initiative 4	National	both	8	8	1675	1664	
2616	Poland	2015-2016	LIPIDOGRAM2015 & LIPIDOGEN2015 Study - National epidemiological study of lipid disorders and selected risk factors of cardiovascular disease in primary health care in Poland	National	both	18+	18+	5034	8690	
2617	Poland	2016	Body fat in Polish adolescents	Community	urban	11-13	11-13	76	82	
2618	Poland	2016-2017	Erasmus plus KA2, Healthyland	Community	urban	6	6	25	25	
2619	Poland	2016-2017	The occurrence of overweight and obesity in children from different place of residence	Subnational	both	7-13	7-13	164	151	
2620	Poland	2016-2020	The prevalence of overweight and obesity and the assessment of body balance among children in a rural area in Poland	Subnational	rural	7-15	7-15	580	557	
2621	Poland	2017	Multivariate assessment of the occurrence of noncommunicable diseases and their risk factors among preschool children	Community	urban	5-7	5-7	226	206	
2622	Poland	2017	Preferences for sweet and fatty taste in children and their mothers in association with weight status	Subnational	both	8-15	8-15	138	150	
2623	Poland	2017	Risk factors for obesity among Polish adolescents	Community	both	14-19	14-19	40	38	
2624	Poland	2017-2018	Erasmus plus KA2, Healthyland	Community	urban	5-6	5-6	24	26	
2625	Poland	2018	Childhood Obesity Surveillance Initiative 5	National	both	8	8	1387	1303	
2626	Poland	2018	Multivariate assessment of the occurrence of noncommunicable diseases and their risk factors among preschool children	Community	urban	5-7	5-7	393	385	
2627	Poland	2018	Mogielica Human Ecology Study Site	Community	rural	18+	18+	30	95	
2628	Poland	2018-2019	Health status and its socio-economic covariates in the older population in Poland - The PolSenior2 study	National	both	60-106	60-106	2858	2964	
2629	Poland	2018-2020	Child of Kraków 2020	Community	urban	5-18	5-18	1382	1487	
2630	Poland	2019	Multivariate assessment of the occurrence of noncommunicable diseases and their risk factors among preschool children	Community	urban	5-7	5-7	395	375	
2631	Poland	2019-2020	Nationwide Dietary Survey in Poland	National	both	5-96	5-96	1420	1408	
2632	Poland	2019-2020	Assessment of the olfactory and taste functions of children with type 1 diabetes	Community	rural	10-15	10-15	54	46	
2633	Poland	2019-2020	Child health status and risk factors	Subnational	both	6-17	6-17	371	329	
2634	Poland	2019-2021	Population Cohort Study of Wroclaw Citizens (PICTURE)	Community	urban	7-15	7-15	608	580	
2635	Poland	2019-2021	Population Cohort Study of Wroclaw Citizens (PICTURE)	Community	urban	18+	18+	389	806	
2636	Poland	2022-2023	Childhood Obesity Surveillance Initiative 6	National	both	7-9	7-9	3100	3268	
2637	Poland	2023	National conscripts survey in Poland in 2023	National	both	18-19		13830		
2638	Poland	2024	Development and Validation of the Polish Geriatric Core Set	Community	both	60+	60+	192	354	
2639	Poland	2024	Association of anthropometric measurements, sleep quality, physical activity and cardiorespiratory fitness in young adults in the Podkarpackie region	Subnational	both	18-35	18-35	40	59	
2640	Portugal	1980-1982	Growth of children from the countryside of Portugal	Community	both	6-15	6-15	1349	1316	
2641	Portugal	1985	Body-Mass Index of Portuguese Conscripts	National	both	18-20		29420		
2642	Portugal	1986	Body-Mass Index of Portuguese Conscripts	National	both	18-20		70504		
2643	Portugal	1986	INTERSALT	Community	rural	20-59	20-59	99	99	
2644	Portugal	1987	Body-Mass Index of Portuguese Conscripts	National	both	18-20		68079		
2645	Portugal	1988	Body-Mass Index of Portuguese Conscripts	National	both	18-20		67573		
2646	Portugal	1989	Body-Mass Index of Portuguese Conscripts	National	both	18-20		68827		
2647	Portugal	1990	Body-Mass Index of Portuguese Conscripts	National	both	18-20		44359		
2648	Portugal	1991	Body-Mass Index of Portuguese Conscripts	National	both	18-20		19552		
2649	Portugal	1992	Body-Mass Index of Portuguese Conscripts	National	both	18-20		52393		
2650	Portugal	1993	Body-Mass Index of Portuguese Conscripts	National	both	18-20		59780		
2651	Portugal	1994	Body-Mass Index of Portuguese Conscripts	National	both	18-20		55511		
2652	Portugal	1995	Body-Mass Index of Portuguese Conscripts	National	both	18-20		68221		
2653	Portugal	1996	Body-Mass Index of Portuguese Conscripts	National	both	18-21		106097		
2654	Portugal	1997	Body-Mass Index of Portuguese Conscripts	National	both	18-21		61215		
2655	Portugal	1998	Body-Mass Index of Portuguese Conscripts	National	both	18-21		41027		
2656	Portugal	1998-2000	European Youth Heart Study	Community	both	9-16	9-16	554	535	
2657	Portugal	1999	Body-Mass Index of Portuguese Conscripts	National	both	18-21		54187		
2658	Portugal	1999-2003	EPIPorto study	Community	urban	18+	18+	932	1507	
2659	Portugal	2000	Body-Mass Index of Portuguese Conscripts	National	both	18-21		53326		
2660	Portugal	2003-2004	EPITeen - Epidemiological Health Investigation of Teenagers in Porto	Community	urban	13-14	13-14	981	1048	
2661	Portugal	2003-2005	Estudo de Prevalência da Obesidade e Consumos Alimentares em Portugal	National	both	18-64	18-64	3796	4320	
2662	Portugal	2004	Growth of adolescents in Coimbra	Community	both	9-16	9-16	265	408	

	Country	Study years	Survey/Study name/Citation	Level of representative-ness	Rural, urban, or both	Age range as in NCD-RisC database		Sample size		Note
						Male	Female	Male	Female	
2663	Portugal	2004	Growth of adolescents in Gouveia	Community	rural	10-19	10-19	238	246	
2664	Portugal	2007	Growth of adolescents in Tondela	Community	rural	6-19	6-19	314	312	
2665	Portugal	2007-2008	EPITeen - Epidemiological Health Investigation of Teenagers in Porto	Community	urban	16-17	16-17	1186	1254	
2666	Portugal	2007-2008	European Youth Heart Study	Community	both	8-17	8-17	315	311	
2667	Portugal	2007-2008	Primary schools health promotion	Community	urban	6-12	6-12	224	238	
2668	Portugal	2007-2009	Portuguese National Survey of Physical Activity and Physical Fitness	National	both	10+	10+	15475	18196	
2669	Portugal	2007-2010	Promoção do Exercício e Saúde no Sedentarismo e Obesidade da Adolescência (PESSOA Program)	Community	urban	9-16	9-16	1931	1813	
2670	Portugal	2007-2010	The Midland Adolescent Lifestyle Study	Subnational	both	12-16	12-16	196	235	
2671	Portugal	2008	Childhood Obesity Surveillance Initiative 1	National	both	6-8	6-8	1801	1792	
2672	Portugal	2008	Azorean Physical Activity and Health Study II	Subnational	urban	15-18	15-18	608	893	
2673	Portugal	2008-2009	PREVADIAB	National	both	20-79	20-79	1957	2913	
2674	Portugal	2008-2013	Preschool Physical Activity, Body Composition and Lifestyle Study (PRESTYLE)	Community	urban	5-6	5-6	651	607	
2675	Portugal	2009	Bracara Study	Community	urban	8-14	8-14	398	336	
2676	Portugal	2009-2010	Portuguese Prevelance Study of Obesity in Childhood	National	both	5-10	5-10	6804	7099	
2677	Portugal	2010	Childhood Obesity Surveillance Initiative 2	National	both	6-8	6-8	1830	1826	
2678	Portugal	2010-2012	Promoção do Exercício e Saúde no Sedentarismo e Obesidade da Adolescência (PESSOA Program)	Community	urban	9-14	9-14	287	275	
2679	Portugal	2010-2012	Exercise for Elderly	Community	urban	60-84	60-84	48	104	
2680	Portugal	2011-2012	The association of childhood obesity with asthma and rhinitis symptoms in 6-8 years old children living in the Coimbra district, Portugal: the role of environmental, family and socioeconomic factors	Community	both	6-8	6-8	480	504	
2681	Portugal	2011-2013	International Study of Childhood Obesity, Lifestyle and the Environment (ISCOLE)	Community	urban	9-11	9-11	358	419	
2682	Portugal	2011-2013	Environmental Support for Leisure and Active Transport	Subnational	urban	10-15	10-15	294	340	
2683	Portugal	2011-2013	EPITeen - Epidemiological Health Investigation of Teenagers in Porto	Community	urban	20-23	20-23	854	898	
2684	Portugal	2011-2014	Longitudinal Analysis of Biomarkers and Environmental Determinants of Physical activity (LABMED Study)	Subnational	urban	12-18	12-18	531	460	
2685	Portugal	2013	Childhood Obesity Surveillance Initiative 3	National	both	6-8	6-8	2951	2982	
2686	Portugal	2013	Childhood obesity in Lousao	Community	rural	5-14	5-14	444	418	
2687	Portugal	2013-2014	Cultural, social, economic, and environmental factors that can influence children's sport participation and obesity levels	Community	urban	6-10	6-10	385	408	
2688	Portugal	2014-2015	EPITeen - Epidemiological Health Investigation of Teenagers in Porto	Community	urban	23-25	23-25	544	549	
2689	Portugal	2015	Inquérito Nacional de Saúde com Exame Físico (INSEF)	National	both	25-74	25-74	2249	2608	
2690	Portugal	2015-2016	Childhood Obesity Surveillance Initiative 4	National	both	6-8	6-8	3346	3399	
2691	Portugal	2015-2016	National Food, Nutrition and Physical Activity Survey of the Portuguese general population	National	both	5-84	5-84	2210	2416	
2692	Portugal	2016-2017	Overweight and obesity and their associated factors among early adolescence school children in urban and rural Portugal	National	rural	10-12	10-12	38	33	
2693	Portugal	2016-2017	Portuguese Prevalence Study of Obesity in Childhood	Subnational	both	5-10	5-10	3572	3522	
2694	Portugal	2017-2019	Portuguese National Survey of Physical Activity and Physical Fitness	National	both	10+	10+	6789	8987	
2695	Portugal	2017-2019	EPITeen - Epidemiological Health Investigation of Teenagers in Porto	Community	urban	26-28	26-28	560	571	
2696	Portugal	2018-2019	Childhood Obesity Surveillance Initiative 5	National	both	6-8	6-8	3626	3429	
2697	Portugal	2022	Childhood Obesity Surveillance Initiative 6	National	both	6-8	6-8	3121	3106	
2698	Puerto Rico	2002-2003	Puerto Rican Elderly: Health Conditions	National	both	60+	60+	1914	2850	29
2699	Puerto Rico	2005-2007	Perez et al., Ethn Dis 18(4):434-41, 2008	Community	urban	15-84	15-84	275	529	
2700	Puerto Rico	2006-2007	Puerto Rican Elderly: Health Conditions	National	both	60+	60+	1056	1669	29
2701	Puerto Rico	2010-2013	HPV Infection in a Population-Based Sample of Puerto Rican Women	Subnational	both		16-64		563	
2702	Qatar	2006	World Health Survey	National	both	18+	18+	1859	2018	
2703	Qatar	2011	Global School-based Student Health Survey	National	both	13	13	102	126	
2704	Qatar	2012	STEPS	National	both	18-64	18-64	1034	1353	
2705	Romania	1986-1987	MONICA, Bucharest	Community	urban	25-64	25-64	702	873	
2706	Romania	1997	Somatometria	National	both	25-75	25-75	3142	4063	
2707	Romania	1999	Romania physical development rural data	National	rural	5-18	5-18	20452	20043	
2708	Romania	1999	Romania physical development urban data	National	urban	5-18	5-18	21423	23605	
2709	Romania	2006-2008	Hypertension in Romanian Children and Adolescents: A Cross-Sectional Survey	Subnational	both	5-17	5-17	2313	2339	
2710	Romania	2008	Healthy traditions for healthy children	Community	rural	5-11	5-11	74	69	
2711	Romania	2008-2009	Healthy traditions for healthy children	Community	urban	7-11	7-11	562	525	
2712	Romania	2009-2011	Study on children in Dolj County, South Romania	Subnational	both	5-21	5-21	746	672	
2713	Romania	2010-2013	Healthy traditions for healthy children	Community	urban	5-11	5-11	1306	1362	
2714	Romania	2011-2012	SEPHAR II (Study for the Evaluation of Prevalence of Hypertension and Cardiovascular Risk in Romania - 2nd edition)	National	both	18-80	18-80	927	1023	
2715	Romania	2012	Healthy traditions for healthy children	Community	rural	5-10	5-10	98	75	
2716	Romania	2012-2014	PREDATORR	National	both	20-79	20-79	1284	1431	
2717	Romania	2013	Childhood Obesity Surveillance Initiative 3	National	both	8	8	2175	2173	
2718	Romania	2013	Healthy traditions for healthy children	Subnational	rural	6-10	6-10	121	107	
2719	Romania	2013-2014	Auxological evaluation of school children in Mures County	Subnational	both	6-14	6-14	936	957	
2720	Romania	2014	Timis County Study	Community	urban	6-19	6-19	237	205	
2721	Romania	2014-2015	Healthy traditions for healthy children	Subnational	rural	5-11	5-11	599	554	
2722	Romania	2014-2015	Healthy traditions for healthy children	Community	urban	5-10	5-10	393	427	
2723	Romania	2015-2016	Childhood Obesity Surveillance Initiative 4	National	both	7-9	7-9	3699	3595	
2724	Romania	2015-2016	SEPHAR III (Study for the Evaluation of Prevalence of Hypertension and Cardiovascular Risk in Romania - 3rd edition)	National	both	18-80	18-80	936	1034	

	Country	Study years	Survey/Study name/Citation	Level of representative-ness	Rural, urban, or both	Age range as in NCD-RisC database		Sample size		Note
						Male	Female	Male	Female	
2725	Romania	2016	Healthy traditions for healthy children	Community	rural	5-11	5-11	352	313	
2726	Romania	2016	Healthy traditions for healthy children	Community	urban	5-11	5-11	259	255	
2727	Romania	2016-2017	Erasmus plus KA2, Healthyland	Community	urban	5-6	5-6	14	16	
2728	Romania	2017	Healthy traditions for healthy children	Community	rural	5-11	5-11	226	231	
2729	Romania	2017	Healthy traditions for healthy children	Community	urban	5-11	5-11	582	588	
2730	Romania	2017-2018	Erasmus plus KA2, Healthyland	Community	urban	5-7	5-7	31	34	
2731	Romania	2018	Healthy traditions for healthy children	Community	rural	5-11	5-11	456	404	
2732	Romania	2018	Healthy traditions for healthy children	Community	urban	5-11	5-11	679	681	
2733	Romania	2019	Childhood Obesity Surveillance Initiative 5	National	both	7-9	7-9	5234	5142	
2734	Romania	2019	Healthy traditions for healthy children	Community	rural	5-11	5-11	78	92	
2735	Romania	2019	Healthy traditions for healthy children	Community	urban	5-11	5-11	353	340	
2736	Romania	2019	Resita preschool measurements	Community	urban	5-8	5-8	232	236	
2737	Romania	2021	SEPHAR IV (Study for the Evaluation of Prevalence of Hypertension and Cardiovascular Risk in Romania - 4th edition)	National	both	18-80	18-80	581	875	
2738	Romania	2022	Healthy traditions for healthy children	Community	rural	5-11	5-11	232	210	
2739	Romania	2022	Healthy traditions for healthy children	Community	urban	5-11	5-11	1163	982	
2740	Romania	2022	SOLUTION	National	both	18-35	18-35	145	162	
2741	Romania	2023	Childhood Obesity Surveillance Initiative 6	National	both	6-9	6-9	4359	4277	
2742	Romania	2023	Healthy traditions for healthy children	Community	both	5-10	5-10	500	451	
2743	Russian Federation	1984-1985	Novosibirsk cohort semi-MONICA	Community	urban	23-63		1603		
2744	Russian Federation	1984-1986	MONICA, Moscow (control)	Community	urban	35-64	35-64	774	642	
2745	Russian Federation	1984-1986	MONICA, Moscow, Leninsky district	Community	urban	35-64	35-64	553	622	
2746	Russian Federation	1984-1986	MONICA, Moscow, Cheremushkinsky district	Community	urban	35-64	35-64	580	579	
2747	Russian Federation	1985	MONICA, Novosibirsk (intervention)	Community	urban	25-64	25-64	797	818	
2748	Russian Federation	1985-1986	MONICA, Novosibirsk, Kirowsky district	Community	urban	25-64	25-64	758	774	
2749	Russian Federation	1985-1986	MONICA, Novosibirsk, Leninsky district	Community	urban	25-64	25-64	624	624	
2750	Russian Federation	1986	INTERSALT	Community	urban	20-59	20-59	97	97	
2751	Russian Federation	1988	MONICA, Novosibirsk (intervention)	Community	urban	25-64	25-64	837	852	
2752	Russian Federation	1988-1989	MONICA, Moscow (control)	Community	urban	35-64	35-64	620	581	
2753	Russian Federation	1988-1989	MONICA, Moscow, Leninsky district	Community	urban	35-64	35-64	597	612	
2754	Russian Federation	1988-1989	MONICA, Novosibirsk, Kirowsky district	Community	urban	25-64	25-64	871	705	
2755	Russian Federation	1992	Russian Karelia Survey in Pitkaranta	Community	both	25-64	25-64	380	455	
2756	Russian Federation	1992	CINDI	Community	rural	25-64	25-64	377	453	
2757	Russian Federation	1992-1993	Russia Longitudinal Monitoring Survey- Higher School of Economics Round II	National	both	5+	5+	4764	6348	
2758	Russian Federation	1992-1995	MONICA, Moscow (control)	Community	urban	35-64	35-64	556	527	
2759	Russian Federation	1992-1995	MONICA, Moscow, Leninsky district	Community	urban	35-64	35-64	538	858	
2760	Russian Federation	1993	Russia Longitudinal Monitoring Survey- Higher School of Economics Round III	National	both	5+	5+	6009	7685	
2761	Russian Federation	1993-1994	Russia Longitudinal Monitoring Survey- Higher School of Economics Round IV	National	both	5+	5+	5519	7094	
2762	Russian Federation	1994	Russia Longitudinal Monitoring Survey- Higher School of Economics Round V	National	both	5+	5+	4726	5788	
2763	Russian Federation	1994-1995	MONICA, Novosibirsk (intervention)	Community	urban	25-64	25-64	820	860	
2764	Russian Federation	1995	Russia Longitudinal Monitoring Survey- Higher School of Economics Round VI	National	both	5+	5+	4463	5509	
2765	Russian Federation	1995	MONICA, Novosibirsk, Kirowsky district	Community	urban	25-64	25-64	771	787	
2766	Russian Federation	1996	Russia Longitudinal Monitoring Survey- Higher School of Economics Round VII	National	both	5+	5+	4261	5300	
2767	Russian Federation	1997	Russian Karelia Survey in Pitkaranta	Community	both	25-64	25-64	309	440	
2768	Russian Federation	1998-1999	Russia Longitudinal Monitoring Survey- Higher School of Economics Round VIII	National	both	5+	5+	4053	5073	
2769	Russian Federation	2000	Russia Longitudinal Monitoring Survey- Higher School of Economics Round IX	National	both	5+	5+	3787	4807	
2770	Russian Federation	2001	Russia Longitudinal Monitoring Survey- Higher School of Economics Round X	National	both	5+	5+	3842	5037	
2771	Russian Federation	2002	Russia Longitudinal Monitoring Survey- Higher School of Economics Round XI	National	both	5+	5+	3819	4951	
2772	Russian Federation	2002	Russian Karelia Survey in Pitkaranta	Community	both	25-64	25-64	251	334	
2773	Russian Federation	2002-2005	Health, Alcohol and Psychosocial Factors In Eastern Europe	Community	urban	45-70	45-70	4240	5069	
2774	Russian Federation	2003	Russia Longitudinal Monitoring Survey- Higher School of Economics Round XII	National	both	5+	5+	3745	4875	
2775	Russian Federation	2003	School Children Moscow	Community	urban	7-8	7-8	133	112	
2776	Russian Federation	2003-2004	Monitoring Arterial Hypertension in Russia	Subnational	both	20-99	20-99	12014	17121	
2777	Russian Federation	2004	Russia Longitudinal Monitoring Survey- Higher School of Economics Round XIII	National	both	5+	5+	3688	4839	
2778	Russian Federation	2004	School Children Moscow	Community	urban	8-9	8-9	180	185	
2779	Russian Federation	2005	Russia Longitudinal Monitoring Survey- Higher School of Economics Round XIV	National	both	5+	5+	3427	4534	
2780	Russian Federation	2005	School Children Moscow	Community	urban	9-10	9-10	178	191	
2781	Russian Federation	2005-2006	Monitoring Arterial Hypertension in Russia	Subnational	both	20-100	20-100	6488	10997	
2782	Russian Federation	2006	School Children Moscow	Community	urban	10-11	10-11	163	195	
2783	Russian Federation	2007	School Children Moscow	Community	urban	11-12	11-12	143	167	
2784	Russian Federation	2007	Russian Karelia Survey in Pitkaranta	Community	both	25-64	25-64	176	276	
2785	Russian Federation	2007-2008	Monitoring Arterial Hypertension in Russia	Subnational	both	20-100	20-100	4100	6522	
2786	Russian Federation	2007-2010	WHO Study on global AGEing and adult health (SAGE)	National	both	50+	50+	1195	2148	
2787	Russian Federation	2008	School Children Moscow	Community	urban	12-13	12-13	111	141	

	Country	Study years	Survey/Study name/Citation	Level of representative-ness	Rural, urban, or both	Age range as in NCD-RisC database		Sample size		Note
						Male	Female	Male	Female	
2788	Russian Federation	2009	School Children Moscow	Community	urban	13-14	13-14	124	140	
2789	Russian Federation	2009-2010	Monitoring Arterial Hypertension in Russia	Subnational	both	20-100	20-100	4007	6407	
2790	Russian Federation	2010	School Children Moscow	Community	urban	14-15	14-15	116	137	
2791	Russian Federation	2011	School Children Moscow	Community	urban	15-16	15-16	117	125	
2792	Russian Federation	2012	School Children Moscow	Community	urban	16-17	16-17	87	108	
2793	Russian Federation	2012-2014	Epidemiology of Cardiovascular Diseases in Different Regions of Russia (ESSE-RF)	National	both	25-64	25-64	8308	13458	
2794	Russian Federation	2015-2016	Childhood Obesity Surveillance Initiative 4 - Moscow	Community	urban	6-8	6-8	1499	1529	
2795	Russian Federation	2015-2017	Ural Eye and Medical Study (UEMS)	Subnational	rural	40+	40+	1530	1870	
2796	Russian Federation	2015-2017	Ural Eye and Medical Study (UEMS)	Community	urban	40+	40+	1050	1449	
2797	Russian Federation	2015-2018	Know Your Heart - Arkhangelsk	Community	urban	35-69	35-69	985	1373	
2798	Russian Federation	2015-2018	Know Your Heart - Novobirsk	Community	urban	35-69	35-69	897	1224	
2799	Russian Federation	2015-2018	Know Your Heart - Arkhangelsk	Community	urban	36-70	36-70	108	96	
2800	Russian Federation	2015-2018	Know Your Heart - Novobirsk	Community	urban	36-70	36-70	75	116	
2801	Russian Federation	2017	Epidemiology of Cardiovascular Diseases in Different Regions of Russia - 2 (ESSE-RF-2)	Subnational	both	25-64	25-64	2979	3670	
2802	Russian Federation	2017-2020	Ural Very Old Study	Community	both	85+	85+	194	516	
2803	Russian Federation	2018	Rosstat sample observation of the population's diet	National	both	5+	5+	31513	44277	
2804	Russian Federation	2019-2020	Childhood Obesity Surveillance Initiative 5 - Moscow	Community	urban	6-7	6-7	1201	1202	
2805	Russian Federation	2019-2021	Ural Children Eye Study	Community	urban	6-18	6-18	1950	2052	
2806	Russian Federation	2020	Childhood Obesity Surveillance Initiative 5 - Ekaterinburg	Community	urban	7	7	1310	1376	
2807	Russian Federation	2021	School Children Moscow	Community	urban	7-18	7-18	1591	1435	
2808	Russian Federation	2021-2024	A study of the physical development of children (Kaluga)	Community	urban	7-18	7-18	647	670	
2809	Russian Federation	2021-2024	A study of the physical development of children (Lipetsk)	Community	urban	7-18	7-18	2469	2536	
2810	Russian Federation	2021-2024	A study of the physical development of children (Stravopol)	Community	urban	7-18	7-18	2459	2298	
2811	Russian Federation	2021-2024	A study of the physical development of children (Ulyanovsk)	Community	urban	7-18	7-18	1600	1529	
2812	Russian Federation	2023	ROSSTAT Health of the Population Study - adults	National	both	15+	15+	32725	47863	
2813	Russian Federation	2023	ROSSTAT Health of the Population Study - children	National	both	5-14	5-14	6087	5815	
2814	Russian Federation	2023	Rosstat sample observation of the population's diet	National	both	5+	5+	29126	41351	
2815	Russian Federation	2023	School Children Moscow	Community	urban	9-16	9-16	113	97	
2816	Rwanda	2000	DHS	National	both		15-49		9175	
2817	Rwanda	2005	DHS	National	both		15-49		5211	
2818	Rwanda	2010	DHS	National	both	15-59	15-49	6472	6572	
2819	Rwanda	2012	STEPS	National	both	15-64	15-64	2644	4243	
2820	Rwanda	2012	Comprehensive Food Security and Vulnerability Analysis and Nutrition Survey	National	both		15-49		3792	
2821	Rwanda	2014-2015	DHS	National	both	15-59	15-49	6366	6313	
2822	Rwanda	2015	Comprehensive Food Security and Vulnerability Analysis and Nutrition Survey	National	both		15-49		6220	
2823	Rwanda	2019-2020	DHS	National	both		15-49		6885	
2824	Rwanda	2021-2022	STEPS	National	both	18-69	18-69	2126	3383	
2825	Saint Kitts and Nevis	2007	STEPS	Subnational	both	25-64	25-64	510	852	
2826	Saint Kitts and Nevis	2011	Global School-based Student Health Survey	National	both	13-17	13-17	650	814	
2827	Saint Lucia	1981	Population Study of Blood Pressure and Associated Factors in St Lucia, West Indies	National	both	15+	15+	168	191	
2828	Saint Lucia	1991-1994	Cooper et al., Am J Public Health 87(2):160-68, 1997	Community	urban	25-100	25-100	491	593	
2829	Saint Lucia	2012	STEPS	National	both	25-64	25-64	665	1097	
2830	Saint Lucia	2019-2020	STEPS	National	both	18-69	18-69	1242	1567	
2831	Saint Vincent and the Grenadines	2013-2014	STEPS	National	both	18-69	18-69	1524	1897	
2832	Saint Vincent and the Grenadines	2018	Global School-based Student Health Survey	National	both	13-17	13-17	737	839	
2833	Samoa	1979-1982	McGarvey, Am J Clin Nutr 53(6 Suppl):1586S-1594S, 1991	National	both	5+	5+	469	501	
2834	Samoa	1991	Non-Communicable Disease Risk Factor (NCDRF)	Subnational	rural	25+	25+	465	496	
2835	Samoa	1991	Non-Communicable Disease Risk Factor (NCDRF)	Subnational	urban	25+	25+	330	444	
2836	Samoa	1991	McGarvey, Pac Health Dialog 8(1):157-62, 2001	National	both	25+	25+	347	381	
2837	Samoa	1993	McGarvey, Pac Health Dialog 8(1):157-62, 2001	National	both	27+	27+	285	336	
2838	Samoa	1995	McGarvey, Pac Health Dialog 8(1):157-62, 2001	National	both	29+	29+	156	157	
2839	Samoa	2002	STEPS	National	both	25-64	25-64	1181	1334	
2840	Samoa	2010	Samoan Genome-Wide Association Study	National	both	24-65	24-65	1402	2061	30
2841	Samoa	2013	STEPS	National	both	18-64	18-64	605	918	
2842	Samoa	2014	DHS	National	both		15-49		3608	
2843	Samoa	2019-2020	Samoa Multiple Indicator Cluster Survey	National	both		15-49		3781	
2844	Sao Tome and Principe	2008-2009	DHS	National	both	15-59	15-49	2173	2238	
2845	Sao Tome and Principe	2009	STEPS	National	both	25-64	25-64	998	1286	
2846	Sao Tome and Principe	2019	STEPS	National	both	18-69	18-69	953	1336	
2847	Saudi Arabia	1985-1988	National Nutrition Survey	National	both	5-75	5-75	2311	3057	
2848	Saudi Arabia	1989-1994	National Nutrition Survey	National	both	18-40	18-40	2481	3294	

	Country	Study years	Survey/Study name/Citation	Level of representative-ness	Rural, urban, or both	Age range as in NCD-RisC database		Sample size		Note
						Male	Female	Male	Female	
2849	Saudi Arabia	1990-1993	National Epidemiological Household Survey	National	both	15-60	15-60	4882	4509	
2850	Saudi Arabia	1990-1993	Saudi National Survey	National	both	30-70	30-70	1612	1648	
2851	Saudi Arabia	1992-1995	Saudi Health Information Survey	National	both	14-50	14-50	4830	7707	
2852	Saudi Arabia	1995	National Household Survey	National	both	20-70	20-70	7114	7069	
2853	Saudi Arabia	1995-2000	National Epidemiological Health Survey	National	both	30-70	30-70	8215	9008	
2854	Saudi Arabia	2004-2005	Al-Baghli et al., Saudi Med J 29(9):1319-25, 2008	Subnational	both	30+	30+	97254	97254	
2855	Saudi Arabia	2005	El Mouzan et al., Ann Saudi Med 30(3):203-208, 2010	National	both	5-18	5-18	9853	9519	
2856	Saudi Arabia	2005	STEPS	National	both	15-64	15-64	2245	2345	
2857	Saudi Arabia	2007	Gulf Cooperation Council World Health Survey	National	both	18+	18+	4854	3610	
2858	Saudi Arabia	2009-2010	Arab Teens Lifestyle Study (ATLS)	Subnational	urban	14-19	14-19	1384	1479	
2859	Saudi Arabia	2009-2010	Biomarker Screening in Riyadh (BSR)	Subnational	both	7-80	7-80	4174	4581	
2860	Saudi Arabia	2011-2012	Jeeluna Study- National Assessment of the Health Needs of Adolescents in Saudi Arabia	National	both	12-19	12-19	6234	5790	
2861	Saudi Arabia	2011-2013	Jeddah City Study	Community	urban	5+	5+	957	867	
2862	Saudi Arabia	2013	Saudi Health Information Survey	National	both	15+	15+	5088	5249	
2863	Senegal	1984	Maire et al., Rev Epidemiol Sante Publique 40:252-58, 1992	National	rural		16-45		1628	
2864	Senegal	1986	Astagneau et al., J Hypertens 10(9):1095-101, 1992	Community	urban	15+	15+	651	707	
2865	Senegal	1986	Maire et al., Rev Epidemiol Sante Publique 40:252-58, 1992	Community	urban		16-45		616	
2866	Senegal	1992-1993	DHS	National	both		20-49		2713	
2867	Senegal	2003	Perceptions of healthy and desirable body size in urban Senegalese women	Community	urban		20-50		287	
2868	Senegal	2005	DHS	National	both		15-49		4166	
2869	Senegal	2010-2011	DHS	National	both	15-59	15-49	4715	5497	
2870	Senegal	2010-2012	Biocultural determinants of overweight and obesity in the context of nutrition transition in Senegal: a holistic anthropological approach	Subnational	both	18+	18+	280	307	
2871	Senegal	2015	National Nutritional Survey Using the SMART Methodology	National	both		12-49		15955	
2872	Senegal	2015	Les maladies chroniques au Sénégal: Une écologie de la santé comparative entre Dakar et Widou Thiengoly	Community	both	20+	20+	734	765	
2873	Senegal	2015	STEPS	National	both	18-70	18-70	1865	3270	
2874	Senegal	2019	National Food Security, Nutrition and Resilience Survey (ENSANR)	National	both		15-49		11173	
2875	Senegal	2024	STEPS	National	both	18-69	18-69	2457	3830	
2876	Serbia	1984	MONICA, Novi Sad	Community	urban	25-64	25-64	798	777	
2877	Serbia	1988-1989	MONICA, Novi Sad	Community	urban	25-64	25-64	778	791	
2878	Serbia	1990	Longitudinal monitoring of growth and nutritional status of children in North Backa Region of Serbia	Community	both	6-8	6-8	7	8	
2879	Serbia	1990-1991	Longitudinal monitoring of growth and nutritional status of children in North Backa Region of Serbia	Community	both	6-9	6-9	20	12	
2880	Serbia	1991-1992	Longitudinal monitoring of growth and nutritional status of children in North Backa Region of Serbia	Community	both	6-9	6-9	83	82	
2881	Serbia	1992-1993	Longitudinal monitoring of growth and nutritional status of children in North Backa Region of Serbia	Community	both	6-7	6-7	99	113	
2882	Serbia	1993-1994	Longitudinal monitoring of growth and nutritional status of children in North Backa Region of Serbia	Community	both	6-11	6-11	111	96	
2883	Serbia	1994-1995	MONICA, Novi Sad	Community	urban	25-64	25-64	600	670	
2884	Serbia	1994-1995	Longitudinal monitoring of growth and nutritional status of children in North Backa Region of Serbia	Community	both	6-11	6-11	1064	1003	
2885	Serbia	1995-1996	Longitudinal monitoring of growth and nutritional status of children in North Backa Region of Serbia	Community	both	6-13	6-13	1276	1224	
2886	Serbia	1996-1997	Longitudinal monitoring of growth and nutritional status of children in North Backa Region of Serbia	Community	both	5-13	5-13	2700	2602	
2887	Serbia	1996-2002	Systematic examinations of recruits in Subotica City in Serbia	Community	urban	17-19		3899		
2888	Serbia	1997-1998	Longitudinal monitoring of growth and nutritional status of children in North Backa Region of Serbia	Community	both	6-13	6-13	1088	1047	
2889	Serbia	1998	Yugoslav study of precursors of atherosclerosis in schoolchildren	National	both	9-10	9-10	266	216	
2890	Serbia	1998-1999	Longitudinal monitoring of growth and nutritional status of children in North Backa Region of Serbia	Community	both	6-16	6-16	944	910	
2891	Serbia	1999-2000	Longitudinal monitoring of growth and nutritional status of children in North Backa Region of Serbia	Community	both	6-12	6-12	567	521	
2892	Serbia	2000	Health Status, Health Needs and Utilization of Health Care of the Population of Serbia	National	both	7+	7+	5079	6189	
2893	Serbia	2000-2001	Longitudinal monitoring of growth and nutritional status of children in North Backa Region of Serbia	Community	both	9-18	9-18	2386	2285	
2894	Serbia	2001-2002	Longitudinal monitoring of growth and nutritional status of children in North Backa Region of Serbia	Community	both	9-18	9-18	2042	2105	
2895	Serbia	2002-2003	Longitudinal monitoring of growth and nutritional status of children in North Backa Region of Serbia	Community	both	5-18	5-18	3040	2516	
2896	Serbia	2003	Yugoslav study of precursors of atherosclerosis in schoolchildren	National	both	14-15	14-15	157	136	
2897	Serbia	2003-2004	Longitudinal monitoring of growth and nutritional status of children in North Backa Region of Serbia	Community	both	5-18	5-18	3242	2915	
2898	Serbia	2004-2005	Longitudinal monitoring of growth and nutritional status of children in North Backa Region of Serbia	Community	both	5-18	5-18	3592	3324	
2899	Serbia	2005	Longitudinal monitoring of growth and nutritional status of children in North Backa Region of Serbia	Community	both	8-13	8-13	666	652	
2900	Serbia	2006	The 2006 National Health Survey for the Population of Serbia	National	both	7+	7+	7888	8558	
2901	Serbia	2013	The 2013 National Health Survey for the Population of Serbia	National	both	7+	7+	7205	8137	
2902	Serbia	2013-2014	Stay Fit for Lifelong Health; the Prevalence of Lifestyle Health Conditions in Serbian Population	National	urban	18-65	18-65	1337	297	
2903	Serbia	2015-2016	Childhood Obesity Surveillance Initiative 4	National	both	6-8	6-8	2475	2386	
2904	Serbia	2017-2021	Serbian National Food Consumption Survey	National	both	5-10	5-10	102	107	
2905	Serbia	2019	Childhood Obesity Surveillance Initiative 5	National	both	7-9	7-9	1697	1504	
2906	Seychelles	1989	Seychelles Heart Survey I	National	both	25-64	25-64	513	568	
2907	Seychelles	1994	Seychelles Heart Survey II	National	both	25-64	25-64	499	563	
2908	Seychelles	1998	School Screening Program	National	both	5-16	5-16	1521	1391	
2909	Seychelles	1999	School Screening Program	National	both	5-16	5-16	2638	2764	
2910	Seychelles	2000	School Screening Program	National	both	5-16	5-16	1767	1820	

	Country	Study years	Survey/Study name/Citation	Level of representative-ness	Rural, urban, or both	Age range as in NCD-RisC database		Sample size		Note
						Male	Female	Male	Female	
2911	Seychelles	2001	School Screening Program	National	both	5-16	5-16	2558	2551	
2912	Seychelles	2002	School Screening Program	National	both	5-16	5-16	2445	2502	
2913	Seychelles	2003	School Screening Program	National	both	5-20	5-20	3383	3461	
2914	Seychelles	2004	School Screening Program	National	both	5-16	5-16	2339	2298	
2915	Seychelles	2004	Seychelles Heart Survey III	National	both	25-64	25-64	568	687	
2916	Seychelles	2005	School Screening Program	National	both	5-15	5-15	2678	2733	
2917	Seychelles	2006	School Screening Program	National	both	5-15	5-15	2657	2600	
2918	Seychelles	2007	Global School-based Student Health Survey	National	both	13-17	13-17	385	467	
2919	Seychelles	2011	School Screening Program	National	both	5-15	5-15	2233	2205	
2920	Seychelles	2012	School Screening Program	National	both	5-15	5-15	2261	2290	
2921	Seychelles	2013	School Screening Program	National	both	5-15	5-15	1921	2069	
2922	Seychelles	2013-2014	Seychelles Heart Survey IV	National	both	25-64	25-64	531	699	
2923	Seychelles	2014	School Screening Program	National	both	5-15	5-15	2083	2170	
2924	Seychelles	2015	Global School-based Student Health Survey	National	both	13-17	13-17	770	888	
2925	Seychelles	2015	School Screening Program	National	both	5-15	5-15	1963	1984	
2926	Seychelles	2016	School Screening Program	National	both	5-15	5-15	1737	1858	
2927	Seychelles	2017	School Screening Program	National	both	5-15	5-15	1497	1570	
2928	Seychelles	2018	School Screening Program	National	both	8-15	8-15	1712	1669	
2929	Seychelles	2019	School Screening Program	National	both	8-15	8-15	1606	1665	
2930	Seychelles	2020	School Screening Program	National	both	8-15	8-15	1349	1467	
2931	Seychelles	2022	School Screening Program	National	both	8-15	8-15	1465	1480	
2932	Seychelles	2023	School Screening Program	National	both	8-15	8-15	1113	1191	
2933	Seychelles	2023	Seychelles Heart Survey V	National	both	18-74	18-74	533	656	
2934	Sierra Leone	2008	DHS	National	both		15-49		3274	
2935	Sierra Leone	2009	STEPS	National	both	25-64	25-64	2200	2319	
2936	Sierra Leone	2010	Sierra Leone 2010 Nutrition Survey	National	both		15-49		12072	
2937	Sierra Leone	2013	DHS	National	both	15-59	15-49	7037	7459	
2938	Sierra Leone	2014	National Nutrition Survey	National	both		15-49		11144	
2939	Sierra Leone	2018	Sierra Leone Integrated Household Survey	National	both	5	5	501	495	
2940	Sierra Leone	2019	DHS	National	both	15-59	15-49	6440	7046	
2941	Sierra Leone	2021	National Nutrition Survey	National	both	10-19	10-49	4928	16756	
2942	Singapore	1982-1985	Thyroid Heart Study	National	both	18+	18+	1030	990	
2943	Singapore	1992	National Health Survey 1992	National	both	18-64	18-64	1741	1701	
2944	Singapore	1993-1995	NUH Heart Study	National	both	26-89	26-89	498	484	
2945	Singapore	1998	National Health Survey 1998	National	both	18-69	18-69	2281	2262	
2946	Singapore	2003-2005	Singapore Longitudinal Ageing Study - Cohort 1; SLAS-1	Community	both	55+	55+	1029	1759	
2947	Singapore	2004	National Health Survey 2004	National	both	18-74	18-74	2057	2091	
2948	Singapore	2004-2007	Singapore Cardiovascular Cohort Study and Singapore Prospective Study Program	National	both	21+	21+	2691	3080	31
2949	Singapore	2008-2013	Singapore Longitudinal Ageing Study - Cohort 2; SLAS-2	Community	both	55+	55+	1138	1883	
2950	Singapore	2009	Social Isolation, Health and Lifestyles Survey (SIHLS) 2009	National	both	60+	60+	2038	2382	
2951	Singapore	2009-2011	The Singapore Chinese Eye Study	Community	both	40-80	40-80	1652	1679	
2952	Singapore	2012-2013	Singapore Health Study	National	both	18-79	18-79	954	1021	32
2953	Singapore	2014-2015	Singapore Health 2 Study	National	both	18-79	18-79	775	978	32
2954	Singapore	2015-2017	The Singapore Chinese Eye Study Follow-Up	Community	both	50+	50+	1278	1348	
2955	Singapore	2016-2017	Transitions in Health, Employment, Social Engagement and Inter-generational Transfers in Singapore Study	National	both	60+	60+	1723	2131	
2956	Singapore	2016-2021	Singapore Health Study (T2)	National	both	22-86	22-86	519	527	
2957	Singapore	2017-2019	The PopulatION HEalth and Eye Disease PRofile in Elderly Singaporeans Study	National	both	60+	60+	1169	1438	
2958	Singapore	2018-2020	Singapore Health 2 Study (T2)	National	both	22-83	22-83	430	499	
2959	Slovakia	1985	Effects of somatic development and environmental factors on blood pressure in children	Community	urban	5-7	5-7	412	388	
2960	Slovakia	1993	Countrywide Integrated Noncommunicable Diseases Intervention Programme	National	both	15-64	15-64	876	1293	
2961	Slovakia	1998	Countrywide Integrated Noncommunicable Diseases Intervention Programme	National	both	15-64	15-64	923	1122	
2962	Slovakia	2001	National Anthropological Survey	National	both	5-6	5-6	896	815	
2963	Slovakia	2001	National Anthropological Survey	National	both	6-18	6-18	10881	10667	
2964	Slovakia	2003	Countrywide Integrated Noncommunicable Diseases Intervention Programme	National	both	15-64	15-64	664	905	
2965	Slovakia	2008	Countrywide Integrated Noncommunicable Diseases Intervention Programme	National	both	15-64	15-64	412	584	
2966	Slovakia	2011	National Anthropological Survey	National	both	7-18	7-18	9064	9028	
2967	Slovakia	2011-2012	European Health Examination Survey	National	both	18-64	18-64	884	1080	
2968	Slovakia	2015-2016	Childhood Obesity Surveillance Initiative 4	National	both	7	7	1390	1379	
2969	Slovakia	2018	Childhood Obesity Surveillance Initiative 5	National	both	7-8	7-8	2975	3004	
2970	Slovakia	2022	Childhood Obesity Surveillance Initiative 6	National	both	7-8	7-8	2308	2356	
2971	Slovenia	1982	The SLOFIT monitoring system	National	both	6-19	6-19	16381	17183	
2972	Slovenia	1983	Analysis of Children's Development in Slovenia (ACDSi)	National	both	7-14	7-14	1580	1576	
2973	Slovenia	1983	The SLOFIT monitoring system	National	both	6-19	6-19	16365	17435	

	Country	Study years	Survey/Study name/Citation	Level of representative-ness	Rural, urban, or both	Age range as in NCD-RisC database		Sample size		Note
						Male	Female	Male	Female	
2974	Slovenia	1984	The SLOFIT monitoring system	National	both	6-19	6-19	21438	22933	
2975	Slovenia	1985	The SLOFIT monitoring system	National	both	6-19	6-19	22496	23043	
2976	Slovenia	1986	The SLOFIT monitoring system	National	both	6-19	6-19	22891	23009	
2977	Slovenia	1987	The SLOFIT monitoring system	National	both	6-19	6-19	49089	48784	
2978	Slovenia	1988	The SLOFIT monitoring system	National	both	6-19	6-19	85444	83390	
2979	Slovenia	1989	The SLOFIT monitoring system	National	both	6-19	6-19	106538	106639	
2980	Slovenia	1990	The SLOFIT monitoring system	National	both	6-19	6-19	129317	128572	
2981	Slovenia	1991	The SLOFIT monitoring system	National	both	6-19	6-19	130726	129842	
2982	Slovenia	1992	The SLOFIT monitoring system	National	both	6-19	6-19	135239	134853	
2983	Slovenia	1993	Analysis of Children's Development in Slovenia (ACDSi)	National	both	6-14	6-14	1674	1678	
2984	Slovenia	1993	The SLOFIT monitoring system	National	both	6-19	6-19	143182	141966	
2985	Slovenia	1994	Analysis of Children's Development in Slovenia (ACDSi)	National	both	14-18	14-18	683	696	
2986	Slovenia	1994	The SLOFIT monitoring system	National	both	6-19	6-19	145348	143290	
2987	Slovenia	1995	The SLOFIT monitoring system	National	both	6-19	6-19	142245	140692	
2988	Slovenia	1996	The SLOFIT monitoring system	National	both	6-19	6-19	140714	138120	
2989	Slovenia	1997	The SLOFIT monitoring system	National	both	6-19	6-19	127551	120331	
2990	Slovenia	1998	The SLOFIT monitoring system	National	both	6-19	6-19	126342	121549	
2991	Slovenia	1999	The SLOFIT monitoring system	National	both	6-19	6-19	122655	119408	
2992	Slovenia	2000	The SLOFIT monitoring system	National	both	6-19	6-19	121918	116281	
2993	Slovenia	2001	The SLOFIT monitoring system	National	both	6-19	6-19	118997	114606	
2994	Slovenia	2001	National Programme of Primary CVD Prevention in Slovenia	National	both	35-65	45-70	1079	953	
2995	Slovenia	2002	The SLOFIT monitoring system	National	both	6-19	6-19	114716	110691	
2996	Slovenia	2002	National Programme of Primary CVD Prevention in Slovenia	National	both	35-65	45-70	34539	34384	
2997	Slovenia	2003	Analysis of Children's Development in Slovenia (ACDSi)	National	both	5-14	5-14	2061	1939	
2998	Slovenia	2003	The SLOFIT monitoring system	National	both	6-19	6-19	117278	112944	
2999	Slovenia	2003	National Programme of Primary CVD Prevention in Slovenia	National	both	35-65	45-70	32472	34448	
3000	Slovenia	2004	Analysis of Children's Development in Slovenia (ACDSi)	National	both	14-19	14-19	950	712	
3001	Slovenia	2004	The SLOFIT monitoring system	National	both	6-19	6-19	117768	112575	
3002	Slovenia	2004	National Programme of Primary CVD Prevention in Slovenia	National	both	35-65	45-70	33872	35277	
3003	Slovenia	2005	The SLOFIT monitoring system	National	both	6-19	6-19	114966	109542	
3004	Slovenia	2005	National Programme of Primary CVD Prevention in Slovenia	National	both	35-65	45-70	27146	26180	
3005	Slovenia	2006	The SLOFIT monitoring system	National	both	6-19	6-19	109877	102838	
3006	Slovenia	2006	National Programme of Primary CVD Prevention in Slovenia	National	both	35-65	45-70	23887	21665	
3007	Slovenia	2007	The SLOFIT monitoring system	National	both	6-19	6-19	103105	100364	
3008	Slovenia	2007	National Programme of Primary CVD Prevention in Slovenia	National	both	35-65	45-70	17832	15634	
3009	Slovenia	2008	The SLOFIT monitoring system	National	both	6-19	6-19	103735	98070	
3010	Slovenia	2008	National Programme of Primary CVD Prevention in Slovenia	National	both	35-65	45-70	14764	13726	
3011	Slovenia	2009	The SLOFIT monitoring system	National	both	6-19	6-19	103669	98891	
3012	Slovenia	2009	National Programme of Primary CVD Prevention in Slovenia	National	both	35-65	45-70	13172	11454	
3013	Slovenia	2010	EuropeaN Energy balance Research to prevent excessive weight Gain among Youth - The ENERGY-project	National	both	10-12	10-12	548	579	
3014	Slovenia	2010	The SLOFIT monitoring system	National	both	6-19	6-19	101163	96686	
3015	Slovenia	2010	National Programme of Primary CVD Prevention in Slovenia	National	both	35-65	45-70	13752	12605	
3016	Slovenia	2011	The SLOFIT monitoring system	National	both	6-19	6-19	100802	96309	
3017	Slovenia	2011	National Programme of Primary CVD Prevention in Slovenia	National	both	35-65	45-70	12835	11953	
3018	Slovenia	2012	The SLOFIT monitoring system	National	both	6-19	6-19	101172	95917	
3019	Slovenia	2012	National Programme of Primary CVD Prevention in Slovenia	National	both	35-65	45-70	13881	14178	
3020	Slovenia	2013	The SLOFIT monitoring system	National	both	6-19	6-19	99191	95666	
3021	Slovenia	2013	National Programme of Primary CVD Prevention in Slovenia	National	both	35-65	45-70	10590	12118	
3022	Slovenia	2013-2014	Analysis of Children's Development in Slovenia (ACDSi)	National	both	6-15	6-15	1665	1627	
3023	Slovenia	2014	Analysis of Children's Development in Slovenia (ACDSi)	National	both	14-19	14-19	703	724	
3024	Slovenia	2014	The SLOFIT monitoring system	National	both	6-21	6-21	102790	97859	
3025	Slovenia	2014	National Programme of Primary CVD Prevention in Slovenia	National	both	35-65	45-70	10448	12467	
3026	Slovenia	2015	The SLOFIT monitoring system	National	both	6-19	6-19	103568	99293	
3027	Slovenia	2015	National Programme of Primary CVD Prevention in Slovenia	National	both	35-65	45-70	6877	7387	
3028	Slovenia	2016	The SLOFIT monitoring system	National	both	6-19	6-19	107421	102174	
3029	Slovenia	2017	The SLOFIT monitoring system	National	both	6-19	6-19	108780	104582	
3030	Slovenia	2018	The SLOFIT monitoring system	National	both	6-19	6-19	110213	105861	
3031	Slovenia	2019	The SLOFIT monitoring system	National	both	6-19	6-19	99445	94791	
3032	Slovenia	2020	The SLOfit monitoring system	Community	both	15-19	15-19	879	816	
3033	Slovenia	2020	The SLOfit monitoring system	National	both	6-14	6-14	34382	32921	
3034	Slovenia	2021	The SLOfit monitoring system	Community	both	15-19	15-19	6724	5950	
3035	Slovenia	2021	The SLOfit monitoring system	National	both	6-14	6-14	82486	78808	
3036	Slovenia	2022	The SLOfit monitoring system	National	both	6-19	6-19	100788	98820	

	Country	Study years	Survey/Study name/Citation	Level of representative-ness	Rural, urban, or both	Age range as in NCD-RisC database		Sample size		Note
						Male	Female	Male	Female	
3037	Slovenia	2023	The SLOfit monitoring system	National	both	6-19	6-19	106394	102927	
3038	Slovenia	2024	The SLOfit monitoring system	National	both	6-19	6-19	105510	102109	
3039	Solomon Islands	2004	A genetic-ecological study of the risk factors for lifestyle-related diseases in Oceanian populations	Community	rural	18+	18+	106	109	
3040	Solomon Islands	2004	A genetic-ecological study of the risk factors for lifestyle-related diseases in Oceanian populations	Community	urban	18+	18+	91	95	
3041	Solomon Islands	2006	STEPS	Subnational	both	15-64	15-64	1031	1375	
3042	Solomon Islands	2009-2010	Furusawa et al., N Z Med J 124(1333):17-28, 2011	Community	rural	5+	5+	256	317	
3043	Solomon Islands	2009-2010	Furusawa et al., N Z Med J 124(1333):17-28, 2011	Community	urban	5-70	5-70	78	118	
3044	Solomon Islands	2015	Solomon Islands Demographic and Health Survey	National	both	15-54	15-49	2792	5583	
3045	Solomon Islands	2015	STEPS	National	both	18-69	18-69	816	978	
3046	Solomon Islands	2017-2018	Impact of sea-level rise and relocation projects on health, ecology, and society in Oceania	Community	both	5+	5+	147	321	
3047	Somalia	2016	The prevalence of selected risk factors for non-communicable diseases in Hargeisa, Somaliland: a cross-sectional study	Community	urban	20-69	20-69	145	955	
3048	South Africa	1989	Temple et al., Ethn Dis 11(3):431-7, 2001	Community	both	15+	15+	457	614	
3049	South Africa	1990	Steyn et al., East Afr Med J 75(1):35-40, 1998	Community	urban	15-64	15-64	292	373	
3050	South Africa	1996	Ellisras Longitudinal Study	Community	rural	5-10	5-10	583	535	
3051	South Africa	1996	Temple et al., Ethn Dis 11(3):431-7, 2001	Community	both	15+	15+	302	406	
3052	South Africa	1997	Ellisras Longitudinal Study	Community	rural	5-11	5-11	1046	968	
3053	South Africa	1998	DHS	National	both	15+	15+	5645	7757	
3054	South Africa	1998	Ellisras Longitudinal Study	Community	rural	5-12	5-12	958	856	
3055	South Africa	1999	Ellisras Longitudinal Study	Community	rural	5-13	5-13	991	917	
3056	South Africa	2000	Ellisras Longitudinal Study	Community	rural	5-14	5-14	936	877	
3057	South Africa	2000-2001	Transition and Health during Urbanisation of South Africans: Children	Subnational	both	9-15	9-15	606	639	
3058	South Africa	2001	Ellisras Longitudinal Study	Community	rural	6-15	6-15	962	904	
3059	South Africa	2002	Ellisras Longitudinal Study	Community	rural	7-16	7-16	890	823	
3060	South Africa	2002	The 1st South African National Youth Risk Behaviour Survey	National	both	14-18	14-18	3609	4139	
3061	South Africa	2002-2003	SASPI	Community	rural	35+	35+	80	275	
3062	South Africa	2003	DHS	National	both	15+	15+	3200	4497	
3063	South Africa	2003	Ellisras Longitudinal Study	Community	rural	8-17	8-17	911	858	
3064	South Africa	2003-2004	Africa Centre Biomeasure Survey	Community	rural	25-49	25-49	778	1693	
3065	South Africa	2004-2006	Li et al., Curationis 30(4):79-87, 2007	Community	both	18-40	18-40	334	270	
3066	South Africa	2007-2008	Cardiometabolic risk profile of South African Learners	Subnational	both	10-16	10-16	496	776	
3067	South Africa	2007-2008	WHO Study on global AGEing and adult health (SAGE)	National	both	50+	50+	1541	2059	
3068	South Africa	2008	National Income Dynamics Study Wave I	National	both	5+	5+	8131	10624	
3069	South Africa	2008	The 2nd South African National Youth Risk Behaviour Survey	National	both	14-18	14-18	3910	4201	
3070	South Africa	2008-2009	Cape Town Bellville South Cohort Study - Baseline evaluation I	Community	urban	35-65	35-65	142	499	
3071	South Africa	2010	Africa Centre Biomeasure Survey	Community	rural	15+	15+	2933	6364	
3072	South Africa	2010-2011	National Income Dynamics Study Wave II	National	both	5+	5+	8383	10996	
3073	South Africa	2011-2012	South Africa National Health and Nutrition Examination Survey	National	both	5+	5+	3717	5640	
3074	South Africa	2011-2013	International Study of Childhood Obesity, Lifestyle and the Environment (ISCOLE)	Community	urban	9-11	9-11	222	327	
3075	South Africa	2012	National Income Dynamics Study Wave III	National	both	5+	5+	10956	14118	
3076	South Africa	2014-2015	National Income Dynamics Study Wave IV	National	both	5+	5+	13548	16775	
3077	South Africa	2014-2015	Health and Aging in Africa: A Longitudinal Study of an INDEPTH Community in South Africa (HAALSI)	Community	rural	40+	40+	2141	2502	
3078	South Africa	2016	DHS	National	both	15-59	15-49	2807	3263	
3079	South Africa	2017	National Income Dynamics Study Wave V	National	both	5+	5+	13996	17688	
3080	South Africa	2017-2019	The Exercise, Arterial Modulation and Nutrition in Youth South Africa (ExAMIN Youth SA) Study	Subnational	urban	5-9	5-9	483	575	
3081	South Africa	2018-2020	Vukuzazi Study	Community	both	15+	15+	5735	11836	
3082	South Korea	1986	INTERSALT	Community	urban	20-59	20-59	100	98	
3083	South Korea	1990	Korean National Blood Pressure Survey	National	both	30+	30+	9730	12617	
3084	South Korea	1992-1993	Park et al., Diabetes Res Clin Pract 34 Suppl:S65-72, 1996	Subnational	both	30-89	30+	1077	1392	
3085	South Korea	1997-1998	National Anthropometric Survey in Korean Children and Adolescents	National	both	5-19	5-19	29318	26469	
3086	South Korea	1998	Korea National Health and Nutrition Examination Survey	National	both	10+	10+	4514	5193	
3087	South Korea	1999	The South Korean Conscription Database	National	both	19		401721		
3088	South Korea	2000	The South Korean Conscription Database	National	both	19		402758		
3089	South Korea	2001	Korea National Health and Nutrition Examination Survey	National	both	5+	5+	4150	4815	
3090	South Korea	2001	Kim et al., Br J Psychiatry 185:102-7, 2004	Community	both	65+	65+	300	432	
3091	South Korea	2001	The South Korean Conscription Database	National	both	19		398653		
3092	South Korea	2002	The South Korean Conscription Database	National	both	19		367024		
3093	South Korea	2002-2003	Korean National Health Insurance	National	both	40+	40+	2993634	2483306	
3094	South Korea	2003	The South Korean Conscription Database	National	both	19		329626		
3095	South Korea	2004	The South Korean Conscription Database	National	both	19		323001		
3096	South Korea	2004-2005	Korean National Health Insurance	National	both	40+	40+	3604097	3261164	
3097	South Korea	2005	Korea National Health and Nutrition Examination Survey	National	both	5+	5+	3183	3896	
3098	South Korea	2005	National Anthropometric Survey in Korean Children and Adolescents	National	both	5-19	5-19	41727	39200	
3099	South Korea	2005	The South Korean Conscription Database	National	both	19		313378		

	Country	Study years	Survey/Study name/Citation	Level of representative-ness	Rural, urban, or both	Age range as in NCD-RisC database		Sample size		Note
						Male	Female	Male	Female	
3100	South Korea	2006	The South Korean Conscription Database	National	both	19		302587		
3101	South Korea	2006-2007	Korean National Health Insurance	National	both	40+	40+	4569655	4613826	
3102	South Korea	2007	Korea National Health and Nutrition Examination Survey	National	both	5+	5+	1753	2174	
3103	South Korea	2007	The South Korean Conscription Database	National	both	19		312795		
3104	South Korea	2007-2012	JS High-School Study	Community	rural	14-17	14-17	553	508	
3105	South Korea	2008	Korea National Health and Nutrition Examination Survey	National	both	5+	5+	3849	4824	
3106	South Korea	2008	The South Korean Conscription Database	National	both	19		312919		
3107	South Korea	2008-2009	Korean National Health Insurance	National	both	40+	40+	5763909	6089441	
3108	South Korea	2009	Korea National Health and Nutrition Examination Survey	National	both	5+	5+	4288	5182	
3109	South Korea	2009	Korea National School Health Examination Survey (KNSHES)	National	both	6-18	6-18	103997	89881	
3110	South Korea	2009	The South Korean Conscription Database	National	both	19		324818		
3111	South Korea	2010	Korea National Health and Nutrition Examination Survey	National	both	5+	5+	3583	4312	
3112	South Korea	2010	Korea National School Health Examination Survey (KNSHES)	National	both	6-18	6-18	99865	86167	
3113	South Korea	2010	The South Korean Conscription Database	National	both	19		347249		
3114	South Korea	2010-2011	Korean National Health Insurance	National	both	40+	40+	6671572	7127111	
3115	South Korea	2011	Korea National Health and Nutrition Examination Survey	National	both	5+	5+	3363	4193	
3116	South Korea	2011	Korea National School Health Examination Survey (KNSHES)	National	both	6-18	6-18	97363	83046	
3117	South Korea	2011	The South Korean Conscription Database	National	both	19		364982		
3118	South Korea	2012	Korea National Health and Nutrition Examination Survey	National	both	5+	5+	3194	4029	
3119	South Korea	2012	Korea National School Health Examination Survey (KNSHES)	National	both	6-18	6-18	45066	42005	
3120	South Korea	2012	The South Korean Conscription Database	National	both	19		361009		
3121	South Korea	2012-2013	Korean National Health Insurance	National	both	40+	40+	7256898	7782621	
3122	South Korea	2013	Korea National Health and Nutrition Examination Survey	National	both	5+	5+	3211	3934	
3123	South Korea	2013	Korea National School Health Examination Survey (KNSHES)	National	both	6-18	6-18	43667	40776	
3124	South Korea	2013	Panel Study on Korean Children (PSKC)	National	both	5-6	5-6	851	800	
3125	South Korea	2013	The South Korean Conscription Database	National	both	19		363914		
3126	South Korea	2014	Korea National Health and Nutrition Examination Survey	National	both	5+	5+	2966	3769	
3127	South Korea	2014	Korea National School Health Examination Survey (KNSHES)	National	both	6-18	6-18	42570	39987	
3128	South Korea	2014	Panel Study on Korean Children (PSKC)	National	both	6-7	6-7	790	753	
3129	South Korea	2014	The South Korean Conscription Database	National	both	19		363597		
3130	South Korea	2014-2015	Korean National Health Insurance	National	both	40+	40+	7869485	8354998	
3131	South Korea	2015	Korea National Health and Nutrition Examination Survey	National	both	5+	5+	3022	3640	
3132	South Korea	2015	Korea National School Health Examination Survey (KNSHES)	National	both	6-18	6-18	43141	41645	
3133	South Korea	2015	Panel Study on Korean Children (PSKC)	National	both	7-8	7-8	797	759	
3134	South Korea	2015	The South Korean Conscription Database	National	both	19		350518		
3135	South Korea	2016	Korea National Health and Nutrition Examination Survey	National	both	5+	5+	3288	4042	
3136	South Korea	2016	Korea National School Health Examination Survey (KNSHES)	National	both	6-18	6-18	42242	40631	
3137	South Korea	2016	Panel Study on Korean Children (PSKC)	National	both	8-9	8-9	769	726	
3138	South Korea	2016	The South Korean Conscription Database	National	both	19		339410		
3139	South Korea	2016-2017	Korean National Health Insurance	National	both	40+	40+	8534031	9071978	
3140	South Korea	2017	Korea National Health and Nutrition Examination Survey	National	both	5+	5+	3341	3986	
3141	South Korea	2017	Panel Study on Korean Children (PSKC)	National	both	9-10	9-10	740	709	
3142	South Korea	2017	The South Korean Conscription Database	National	both	19		323457		
3143	South Korea	2018	Korea National Health and Nutrition Examination Survey	National	both	5+	5+	3277	3985	
3144	South Korea	2018	Panel Study on Korean Children (PSKC)	National	both	10-11	10-11	667	638	
3145	South Korea	2018-2019	Korean National Health Insurance	National	both	40+	40+	9307694	9938357	
3146	South Korea	2019	Korea National Health and Nutrition Examination Survey	National	both	5+	5+	3377	3992	
3147	South Korea	2019	Panel Study on Korean Children (PSKC)	National	both	11-12	11-12	704	664	
3148	South Korea	2020	Korea National Health and Nutrition Examination Survey	National	both	5+	5+	3144	3610	
3149	South Korea	2020	Panel Study on Korean Children (PSKC)	National	both	12-13	12-13	486	494	
3150	South Korea	2021	Korea National Health and Nutrition Examination Survey	National	both	5+	5+	2907	3546	
3151	South Korea	2021	Panel Study on Korean Children (PSKC)	National	both	13-14	13-14	664	635	
3152	South Korea	2022	Korea National Health and Nutrition Examination Survey	National	both	5+	5+	2671	3297	
3153	South Korea	2023	Korea National Health and Nutrition Examination Survey	National	both	5+	5+	2969	3662	
3154	South Sudan	2017	Prevalence of hypertension and associated cardiovascular risk factors among adults aged 18-69 years in Juba City, South Sudan	Community	urban	18-69	18-69	365	464	
3155	Spain	1985	INTERSALT, Manresa	Community	urban	20-59	20-59	100	100	
3156	Spain	1986	INTERSALT, Torrejo	Community	urban	20-59	20-59	100	100	
3157	Spain	1986-1988	MONICA, Catalonia	Subnational	both	25-64	25-64	1251	1271	
3158	Spain	1989	Cardiovascular Risk Factors Study in Catalonia	Subnational	both	15+	15+	330	371	
3159	Spain	1989-1994	SEEDO	Subnational	both	25-60	25-60	2533	2855	
3160	Spain	1990	Banegas et al., Hypertension 32(6):998-1002, 1998	National	both	35-65	35-65	810	1203	
3161	Spain	1990-1992	MONICA, Catalonia	Subnational	both	25-64	25-64	1719	1191	

	Country	Study years	Survey/Study name/Citation	Level of representative-ness	Rural, urban, or both	Age range as in NCD-RisC database		Sample size		Note
						Male	Female	Male	Female	
3162	Spain	1990-2000	SEEDO	Subnational	both	25-60	25-60	4707	5178	
3163	Spain	1991-1993	Encuesta de Factores de Riesgo Cardiovascular en la Región de Murcia (Cardiovascular Risk Factors Survey)	Subnational	both	18-69	18-69	1512	1562	
3164	Spain	1992	CINDI	Subnational	both	25-64	25-64	1194	1454	
3165	Spain	1992	ENCAT	Community	both	15-80	15-80	786	952	
3166	Spain	1994-1995	Encuesta de Nutrición y Salud Comunidad Valenciana 1994-95 (ENCV)	Subnational	urban	15+	15+	830	959	
3167	Spain	1994-1996	MONICA, Catalonia	Subnational	both	25-64	25-64	1800	1628	
3168	Spain	1996	Guía Study	Community	urban	30+	30+	305	384	
3169	Spain	1996-2002	Castells et al., J Epidemiol Community Health 60(4):316-21, 2006	Community	urban		50-69		26963	
3170	Spain	1997	Soriguer et al., Eur J Epidemiol 19(1):33-40, 2004	Community	rural	18-65	18-65	611	610	
3171	Spain	1998-2000	EnKID study	National	both	5-24	5-24	1452	1730	
3172	Spain	1999-2000	Factores de riesgo en las islas Baleares: Estudio CORSAIB	Subnational	both	35-74	35-74	802	857	
3173	Spain	1999-2000	ENIB	Community	both	20-60	20-60	498	702	
3174	Spain	2000-2001	Regidor et al., J Hum Hypertens 20(1):73-82, 2006	National	both	60+	60+	1318	2281	
3175	Spain	2000-2001	EUREYE Study	Subnational	both	65+	65+	274	324	
3176	Spain	2000-2005	CDC of the Canary Islands	Subnational	both	18-75	18-75	2878	3719	
3177	Spain	2001-2002	Catalan Health Interview Survey	Subnational	both	18-74	18-74	597	745	
3178	Spain	2001-2003	DIabetes, Nutrición y Obesidad en la población adulta de la Región de Murcia (DINO)	Subnational	both	20+	20+	715	828	
3179	Spain	2002-2003	ENCAT	Community	both	15-80	15-80	712	813	
3180	Spain	2003	The European Male Ageing Study	Community	both	40+		405		
3181	Spain	2003-2005	Registre Gironi del Cor (REGICOR)	Subnational	both	35-79	35-79	2951	3266	
3182	Spain	2004	Cardiovascular Risk Study in Castilla y León (RECCyL)	Subnational	both	15+	15+	1903	2077	
3183	Spain	2004	Vioque J et al., Obesity 16(3):664-70, 2008	Community	urban	24+	24+	87	115	
3184	Spain	2004-2006	PREVICTUS	National	both	60+	60+	3193	3640	
3185	Spain	2006-2007	HELENA	Community	urban	12-17	12-17	188	193	
3186	Spain	2006-2008	Biblioni Mdel et al., Br J Nutr 103(1):99-106, 2010	Community	both	12-17	12-17	570	651	
3187	Spain	2006-2008	Peripheral Arterial Disease Study (ARTPER-1)	Community	both	49-97	49-97	1744	2037	
3188	Spain	2007-2009	Harmonizing Equation of Risk in Mediterraneon countries EXtremadura (HERMEX)	Subnational	both	25-79	25-79	1298	1498	
3189	Spain	2007-2010	Identification and prevention of Dietary- and lifestyle-induced health EFfects In Children and infantS (IDEFICS)	Community	urban	5-9	5-9	474	468	
3190	Spain	2008	The European Male Ageing Study	Community	both	45+		272		
3191	Spain	2008-2010	Study on Nutrition and Cardiovascular Risk in Spain	National	both	18+	18+	5756	6397	
3192	Spain	2009	Cardiovascular Risk Study in Castilla y León (RECCyL)	Subnational	both	20+	20+	1315	1590	
3193	Spain	2009-2010	Di@bet.es Study	National	both	18+	18+	2157	2874	
3194	Spain	2010	EuropeaN Energy balance Research to prevent excessive weight Gain among Youth - The ENERGY-project	Subnational	urban	10-12	10-12	479	520	
3195	Spain	2010-2011	ALimentación, Actividad Física, Desarrollo INfantil y Obesidad (ALADINO); Childhood Obesity Surveillance Initiative 2	National	both	6-9	6-9	3837	3817	
3196	Spain	2010-2013	Peripheral Arterial Disease Study (ARTPER-2)	Community	both	53-92	53-92	1233	1511	
3197	Spain	2012	Effects of a lifestyle intervention on the prevention of childhood obesity: a community-based model	Subnational	urban	8-12	8-12	1171	1081	
3198	Spain	2012-2013	Infancia y Medio Ambiente (Childhood and Environment) Birth Cohort study - Sabadell	Subnational	urban	5-7	5-7	280	260	33
3199	Spain	2012-2013	INfancia y Medio Ambiente (Childhood and Environment) Project - Valencia	Subnational	both	7-8	7-8	229	232	
3200	Spain	2012-2013	Brain Development and Air Pollution Ultrafine Particles in School Children-BREATHE Project	Subnational	urban	7-12	7-12	1338	1325	
3201	Spain	2012-2013	INfancia y Medio Ambiente (Childhood and Environment) Project - Menorca	Subnational	both	14-15	14-15	162	165	
3202	Spain	2013	ANIBES Study	National	both	9-75	9-75	1160	1125	
3203	Spain	2013	ALimentación, Actividad Física, Desarrollo INfantil y Obesidad (ALADINO); Childhood Obesity Surveillance Initiative 3	National	both	7-8	7-8	1682	1744	
3204	Spain	2013-2014	Effects of a lifestyle intervention on the prevention of childhood obesity: a community-based model	Subnational	urban	9-13	9-13	1086	1004	
3205	Spain	2013-2015	INfancia y Medio Ambiente (Childhood and Environment) Project - Valencia	Subnational	both	8-9	8-9	225	218	
3206	Spain	2013-2016	Infancia y Medio Ambiente (Childhood and Environment) Birth Cohort study - Sabadell	Subnational	urban	7-10	7-10	253	236	33
3207	Spain	2014	Cardiovascular Risk Study in Castilla y León (RECCyL)	Subnational	both	20+	20+	1215	1475	
3208	Spain	2014-2015	Evaluación del FITness en PREescolares (PREFIT) 2014-2015, Almeria	Community	urban	5	5	41	45	
3209	Spain	2014-2015	Evaluación del FITness en PREescolares (PREFIT) 2014-2015, Cadiz	Community	urban	5	5	44	44	
3210	Spain	2014-2015	Evaluación del FITness en PREescolares (PREFIT) 2014-2015, Castellon	Community	urban	5	5	36	46	
3211	Spain	2014-2015	Evaluación del FITness en PREescolares (PREFIT) 2014-2015, Cuenca	Community	urban	5	5	29	45	
3212	Spain	2014-2015	Evaluación del FITness en PREescolares (PREFIT) 2014-2015, Granada	Community	urban	5	5	88	65	
3213	Spain	2014-2015	Evaluación del FITness en PREescolares (PREFIT) 2014-2015, LasPalmas	Community	urban	5	5	58	44	
3214	Spain	2014-2015	Evaluación del FITness en PREescolares (PREFIT) 2014-2015, Madrid	Community	urban	5	5	43	42	
3215	Spain	2014-2015	Evaluación del FITness en PREescolares (PREFIT) 2014-2015, Mallorca	Community	urban	5	5	36	44	
3216	Spain	2014-2015	Evaluación del FITness en PREescolares (PREFIT) 2014-2015, Zaragoza	Community	urban	5	5	46	30	
3217	Spain	2015	Evaluación del FITness en PREescolares (PREFIT) 2014-2015, Vitoria	Community	urban	5	5	127	98	
3218	Spain	2015	Study on Nutrition and Cardiovascular Risk in Spain (ENRICA)	National	both	65+	65+	711	770	
3219	Spain	2015-2016	ALimentación, Actividad Física, Desarrollo INfantil y Obesidad (ALADINO); Childhood Obesity Surveillance Initiative 4	National	both	6-9	6-9	5532	5367	
3220	Spain	2015-2016	INfancia y Medio Ambiente (Childhood and Environment) Project - Gipuzkoa	Subnational	both	7-8	7-8	195	195	
3221	Spain	2015-2017	INfancia y Medio Ambiente (Childhood and Environment) Project - Valencia	Subnational	both	9-12	9-12	197	218	
3222	Spain	2016-2017	Estudio de Nutrición y Riesgo Cardiovascular en España (ENRICA)-Seniors cohort	Subnational	urban	65-94	65-94	1340	1478	
3223	Spain	2016-2018	Infancia y Medio Ambiente (Childhood and Environment) Birth Cohort study - Sabadell	Subnational	urban	9-12	9-12	256	239	33
3224	Spain	2017-2019	Urban environment and childhood obesity in Catalonia (ECHOCAT)	Community	urban	9-12	9-12	870	977	

	Country	Study years	Survey/Study name/Citation	Level of representative-ness	Rural, urban, or both	Age range as in NCD-RisC database		Sample size		Note
						Male	Female	Male	Female	
3225	Spain	2018	Childhood obesity cohort study of Sant Boi de Llobregat (SANTBOISA study)	Community	urban	5-10	5-10	299	266	
3226	Spain	2018-2019	INfancia y Medio Ambiente (Childhood and Environment) Project - Gipuzkoa	Subnational	both	10-11	10-11	174	204	
3227	Spain	2019	ALimentación, Actividad Física, Desarrollo INfantil y Obesidad (ALADINO); Childhood Obesity Surveillance Initiative 5	National	both	6-9	6-9	8512	8151	
3228	Spain	2019	Physical Activity, Sedentarism and Obesity of Spanish youth (PASOS study)	National	both	8-16	8-16	1804	1921	
3229	Spain	2019	Estudio de Nutrición y Riesgo Cardiovascular en España (ENRICA)-Seniors cohort	Subnational	urban	65-95	65-95	834	815	
3230	Spain	2019-2021	INfancia y Medio Ambiente (Childhood and Environment) Project - Valencia	Subnational	both	14-16	14-16	118	123	
3231	Spain	2020-2022	INfancia y Medio Ambiente (Childhood and Environment) Project - Sabadell	Subnational	urban	14-17	14-17	170	173	
3232	Spain	2021	INfancia y Medio Ambiente (Childhood and Environment) Project - Gipuzkoa	Subnational	both	13-15	13-15	121	148	
3233	Spain	2021-2022	EHDLA	Community	both	12-17	12-17	622	639	
3234	Spain	2021-2022	INfancia y Medio Ambiente (Childhood and Environment) Project - Gipuzkoa	Subnational	both	17-18	17-18	44	69	
3235	Spain	2022-2023	Physical Activity, Sedentarism and Obesity of Spanish youth (PASOS study)	National	both	8-16	8-16	3349	3513	
3236	Spain	2022-2024	Childhood obesity cohort study of Sant Boi de Llobregat (SANTBOISA study)	Community	urban	5-9	5-9	730	729	
3237	Spain	2023-2024	Childhood Obesity Surveillance Initiative 6	National	both	6-9	6-9	6559	6125	
3238	Sri Lanka	2003	Wijewardene et al., Ceylon Med J 50:62-70, 2005	Subnational	both	30-65	30-65	275	296	
3239	Sri Lanka	2003	Wijewardene et al., Ceylon Med J 50:62-70, 2005	Subnational	both	30-65	30-65	139	192	
3240	Sri Lanka	2003	Wijewardene et al., Ceylon Med J 50:62-70, 2005	Subnational	both	30-65	30-65	1891	2410	
3241	Sri Lanka	2003	Wijewardene et al., Ceylon Med J 50:62-70, 2005	Subnational	both	30-65	30-65	387	457	
3242	Sri Lanka	2005-2006	Sri Lanka Diabetes, Cardiovascular study (SLDCS)	National	both	18+	18+	1773	2714	
3243	Sri Lanka	2006	STEPS	National	both	15-64	15-64	6140	6213	
3244	Sri Lanka	2006-2007	DHS	National	both		15-49		12539	
3245	Sri Lanka	2014	STEPS	National	both	18-69	18-69	1863	2893	
3246	Sri Lanka	2016	Global School-based Student Health Survey	National	both	13-17	13-17	671	923	
3247	Sri Lanka	2016	DHS	National	both		20-49		16929	
3248	Sri Lanka	2018-2019	The Sri Lanka Health and Ageing Study (SLHAS)	National	both	18+	18+	3214	3373	
3249	Sri Lanka	2021	STEPS	National	both	18-69	18-69	2172	3458	
3250	Sri Lanka	2022	Sri Lanka National Nutrition Survey 2022	National	both	5+	5+	1499	2380	
3251	Sri Lanka	2024	Global School-based Student Health Survey	National	both	12-17	12-17	1233	1419	
3252	State of Palestine	1996	Stene et al., Eur J Clin Nutr 55(9):805-11, 2001	Community	rural	30-65	30-65	208	269	
3253	State of Palestine	1996-1998	Ramallah study	Community	rural	15-64	15-64	206	482	
3254	State of Palestine	1996-1998	Ramallah study	Community	urban	15-64	15-64	182	493	
3255	State of Palestine	1999-2000	The First National Health and Nutrition Survey	National	both	18-64	18-64	1736	1869	
3256	State of Palestine	2010	STEPS	National	both	15-64	15-64	2578	4052	
3257	State of Palestine	2010	Global School-based Student Health Survey	National	both	13-17	13-17	1822	1865	
3258	State of Palestine	2013	Palestine Micronutrient Survey	National	both	15-18	15-18	1201	1166	
3259	State of Palestine	2013	West Bank Schools Study	Subnational	both	10-15	10-15	986	2283	
3260	State of Palestine	2022	Global School-based Student Health Survey (UNRWA)	Subnational	both	12-15	12-15	2020	2529	
3261	State of Palestine	2022	STEPS	National	both	18-69	18-69	1677	3637	
3262	Sudan	2005	STEPS	Subnational	both	25-64	25-64	626	881	
3263	Sudan	2016	STEPS	National	both	18-69	18-69	2661	4544	
3264	Sudan	2018	Prevalence and associated factors of hypertension among adults in Gadarif in eastern Sudan: a community-based study	Community	urban	18+	18+	178	421	
3265	Suriname	2013-2015	The Healthy Life in Suriname Study (HELISUR)	Subnational	urban	18-70	18-70	424	722	
3266	Sweden	1977	The Swedish Conscription Database	National	both	17-19		47898		1
3267	Sweden	1978	The Swedish Conscription Database	National	both	17-19		11486		1
3268	Sweden	1979	The Swedish Conscription Database	National	both	17-19		37487		1
3269	Sweden	1979-1980	1973 Birth Cohort	National	both	6	6	451	439	
3270	Sweden	1980	BMI Epidemiology Study	Community	urban	9		324		
3271	Sweden	1980	The Swedish Conscription Database	National	both	17-18		49630		
3272	Sweden	1980-1981	1973 Birth Cohort	National	both	7	7	1422	1323	
3273	Sweden	1980-1981	Population Study of Women in Gothenburg	Community	urban		50-72		1153	
3274	Sweden	1980-1985	Uppsala Longitudinal Study of Adult Men	Community	both	55-64		1846		34
3275	Sweden	1981	The Swedish Conscription Database	National	both	17-18		52282		
3276	Sweden	1981-1982	1973 Birth Cohort	National	both	8	8	890	869	
3277	Sweden	1982	The Swedish Conscription Database	National	both	17-18		54288		
3278	Sweden	1982-1983	1973 Birth Cohort	National	both	9	9	670	635	
3279	Sweden	1983	BMI Epidemiology Study	Community	urban	7		103		
3280	Sweden	1983	The Swedish Conscription Database	National	both	17-18		52895		
3281	Sweden	1983-1984	1973 Birth Cohort	National	both	10	10	1448	1340	
3282	Sweden	1984	BMI Epidemiology Study	Community	urban	8		407		
3283	Sweden	1984	The Swedish Conscription Database	National	both	17-18		36273		
3284	Sweden	1984-1985	1973 Birth Cohort	National	both	11	11	487	498	
3285	Sweden	1985	BMI Epidemiology Study	Community	urban	9		357		
3286	Sweden	1985	The Swedish Conscription Database	National	both	17-18		14918		
3287	Sweden	1985	MONICA Gothenburg	Community	urban	25-64	25-64	666	702	

	Country	Study years	Survey/Study name/Citation	Level of representative-ness	Rural, urban, or both	Age range as in NCD-RisC database		Sample size		Note
						Male	Female	Male	Female	
3288	Sweden	1985-1986	1973 Birth Cohort	National	both	12	12	1238	1158	
3289	Sweden	1985-1989	Västerbotten Intervention Project	Subnational	both	25-64	25-64	1676	1554	
3290	Sweden	1985-1996	EPIC Umea	Subnational	both	24-72	24-72	12359	13217	
3291	Sweden	1986	The Swedish Conscription Database	National	both	17-18		48454		
3292	Sweden	1986	MONICA Northern Sweden	Subnational	both	25-64	25-64	822	798	
3293	Sweden	1986-1987	1973 Birth Cohort	National	both	13	13	709	729	
3294	Sweden	1986-1987	1981 Birth Cohort	National	both	5	5	935	958	
3295	Sweden	1987	The Swedish Conscription Database	National	both	17-18		49306		
3296	Sweden	1987-1988	1973 Birth Cohort	National	both	14	14	1405	1294	
3297	Sweden	1987-1988	1981 Birth Cohort	National	both	6	6	808	780	
3298	Sweden	1988	BMI Epidemiology Study	Community	urban	7		65		
3299	Sweden	1988	The Swedish Conscription Database	National	both	17-18		47923		
3300	Sweden	1988-1989	1973 Birth Cohort	National	both	15	15	964	929	
3301	Sweden	1988-1989	1981 Birth Cohort	National	both	7	7	1508	1489	
3302	Sweden	1989	BMI Epidemiology Study	Community	urban	8		407		
3303	Sweden	1989	The Swedish Conscription Database	National	both	17-18		48117		
3304	Sweden	1989-1990	1973 Birth Cohort	National	both	16	16	912	785	
3305	Sweden	1989-1990	1981 Birth Cohort	National	both	8	8	1497	1511	
3306	Sweden	1990	BMI Epidemiology Study	Community	urban	9		379		
3307	Sweden	1990	The Swedish Conscription Database	National	both	17-18		48882		
3308	Sweden	1990	MONICA Gothenburg	Community	urban	25-64	25-64	775	775	
3309	Sweden	1990	MONICA Northern Sweden	Subnational	both	25-64	25-64	773	806	
3310	Sweden	1990-1991	1973 Birth Cohort	National	both	17	17	881	588	
3311	Sweden	1990-1991	1981 Birth Cohort	National	both	9	9	1091	1108	
3312	Sweden	1990-1992	Västerbotten Intervention Project	Subnational	both	25-64	25-64	7263	7804	
3313	Sweden	1991	The Swedish Conscription Database	National	both	17-18		49150		
3314	Sweden	1991-1992	1981 Birth Cohort	National	both	10	10	1405	1415	
3315	Sweden	1991-1995	Uppsala Longitudinal Study of Adult Men	Community	both	69-74		1215		34
3316	Sweden	1991-1996	Malmö Diet and Cancer	Community	urban	45-73	45-73	12096	18293	
3317	Sweden	1992	The Swedish Conscription Database	National	both	17-18		47275		
3318	Sweden	1992	1973 Birth Cohort	National	both	18	18	1290	390	
3319	Sweden	1992-1993	1981 Birth Cohort	National	both	11	11	785	785	
3320	Sweden	1992-1993	Population Study of Women in Gothenburg	Community	urban		62-84		802	
3321	Sweden	1993	BMI Epidemiology Study	Community	urban	7		125		
3322	Sweden	1993	The Swedish Conscription Database	National	both	17-18		46245		
3323	Sweden	1993-1994	1981 Birth Cohort	National	both	12	12	1318	1378	
3324	Sweden	1993-1995	Västerbotten Intervention Project	Subnational	both	25-64	25-64	9804	10727	
3325	Sweden	1994	BMI Epidemiology Study	Community	urban	8		376		
3326	Sweden	1994	The Swedish Conscription Database	National	both	17-18		43648		
3327	Sweden	1994	Nilson et al., Scand J Prim Health Care 18(2):111-112, 2000	Community	urban	56-65	56-65	170	217	
3328	Sweden	1994	MONICA Northern Sweden	Subnational	both	25-74	25-74	940	961	
3329	Sweden	1994-1995	1981 Birth Cohort	National	both	13	13	924	954	
3330	Sweden	1994-1996	Kungsholmen Project	Community	urban	75+	75+	160	160	
3331	Sweden	1995	BMI Epidemiology Study	Community	urban	9		293		
3332	Sweden	1995	The Swedish Conscription Database	National	both	17-18		43674		
3333	Sweden	1995	MONICA Gothenburg	Community	urban	25-64	25-64	745	867	
3334	Sweden	1995-1996	1981 Birth Cohort	National	both	14	14	1202	1266	
3335	Sweden	1996	The Swedish Conscription Database	National	both	17-18		43794		
3336	Sweden	1996-1997	1981 Birth Cohort	National	both	15	15	1143	1134	
3337	Sweden	1996-1998	Västerbotten Intervention Project	Subnational	both	25-64	25-64	8327	8893	
3338	Sweden	1997	The Swedish Conscription Database	National	both	17-18		37078		
3339	Sweden	1997-1998	1981 Birth Cohort	National	both	16	16	819	862	
3340	Sweden	1997-2001	Uppsala Longitudinal Study of Adult Men	Community	both	73-80		783		34
3341	Sweden	1998	BMI Epidemiology Study	Community	urban	7		707		
3342	Sweden	1998	The Swedish Conscription Database	National	both	17-18		42564		
3343	Sweden	1998-1999	1981 Birth Cohort	National	both	17		406		
3344	Sweden	1998-1999	European Youth Heart Study	Subnational	urban	8-16	8-16	525	602	
3345	Sweden	1998-2001	The Kalixanda study	Community	both	20+	20+	508	483	
3346	Sweden	1999	BMI Epidemiology Study	Community	urban	8		980		
3347	Sweden	1999	The Swedish Conscription Database	National	both	17-18		37852		
3348	Sweden	1999	1981 Birth Cohort	National	both	18		831		
3349	Sweden	1999	MONICA Northern Sweden	Subnational	both	25-74	25-74	889	920	
3350	Sweden	1999-2003	Västerbotten Intervention Project	Subnational	both	25-64	25-64	6354	6384	

	Country	Study years	Survey/Study name/Citation	Level of representative-ness	Rural, urban, or both	Age range as in NCD-RisC database		Sample size		Note
						Male	Female	Male	Female	
3351	Sweden	2000	BMI Epidemiology Study	Community	urban	9		860		
3352	Sweden	2000	The Swedish Conscription Database	National	both	17-18		31328		
3353	Sweden	2000-2001	H70 Study	Community	urban	70	70	242	270	
3354	Sweden	2000-2002	The COMPASS study	Community	urban	14-16	14-16	1718	1597	
3355	Sweden	2001	The Swedish Conscription Database	National	both	17-18		29809		
3356	Sweden	2001-2004	Swedish INTERGENE Cohort Study	Subnational	both	24-76	24-76	1694	1906	
3357	Sweden	2001-2004	PIVUS Study	Community	urban	70	70	1521	1527	
3358	Sweden	2002	The Swedish Conscription Database	National	both	17-18		24580		
3359	Sweden	2003	BMI Epidemiology Study	Community	urban	7		15		
3360	Sweden	2003	The Swedish Conscription Database	National	both	17-18		29198		
3361	Sweden	2003	The European Male Ageing Study	Community	both	40+		396		
3362	Sweden	2003-2004	Welin et al., BMC Public Health 8:403, 2008	Community	urban	50	50	595	655	
3363	Sweden	2003-2004	Welin et al., BMC Public Health 8:403, 2008	Community	urban	60		667		
3364	Sweden	2003-2005	Uppsala Longitudinal Study of Adult Men	Community	both	80-83		512		
3365	Sweden	2004	BMI Epidemiology Study	Community	urban	8		285		
3366	Sweden	2004	The Swedish Conscription Database	National	both	17-18		27857		
3367	Sweden	2004	MONICA Northern Sweden	Subnational	both	26-75	26-75	926	964	
3368	Sweden	2004-2005	European Youth Heart Study	Subnational	urban	15-21	15-21	196	262	
3369	Sweden	2004-2005	Population Study of Women in Gothenburg	Community	urban		38-50		494	
3370	Sweden	2005	BMI Epidemiology Study	Community	urban	9		301		
3371	Sweden	2005	The Swedish Conscription Database	National	both	17-18		25836		
3372	Sweden	2005-2006	H70 Study	Community	urban	75	75	320	422	
3373	Sweden	2006-2007	HELENA	Community	urban	12-17	12-17	132	208	
3374	Sweden	2007	BMI Epidemiology Study	Community	urban	7		1327		
3375	Sweden	2007	Effects of the COVID-19 Pandemic on Children's Body Mass Index Trajectories in Sweden	Subnational	both	6-16	6-16	3469	3310	
3376	Sweden	2007-2009	PIVUS Study	Community	urban	75	75	1221	1257	
3377	Sweden	2007-2010	Identification and prevention of Dietary- and lifestyle-induced health EFfects In Children and infantS (IDEFICS)	Community	urban	5-9	5-9	557	557	
3378	Sweden	2008	BMI Epidemiology Study	Community	urban	7-8		2501		
3379	Sweden	2008	Childhood Obesity Surveillance Initiative 1	National	both	7-9	7-9	2374	2189	
3380	Sweden	2008	Effects of the COVID-19 Pandemic on Children's Body Mass Index Trajectories in Sweden	Subnational	both	6-16	6-16	3491	3342	
3381	Sweden	2008	The European Male Ageing Study	Community	both	45+		353		
3382	Sweden	2008-2009	Uppsala Longitudinal Study of Adult Men	Community	both	84-88		293		
3383	Sweden	2009	BMI Epidemiology Study	Community	urban	7-9		3706		
3384	Sweden	2009	Effects of the COVID-19 Pandemic on Children's Body Mass Index Trajectories in Sweden	Subnational	both	6-16	6-16	3378	3267	
3385	Sweden	2009	MONICA Northern Sweden	Subnational	both	25-74	25-74	849	857	
3386	Sweden	2010	BMI Epidemiology Study	Community	urban	8-9		2425		
3387	Sweden	2010	Effects of the COVID-19 Pandemic on Children's Body Mass Index Trajectories in Sweden	Subnational	both	6-16	6-16	3169	3025	
3388	Sweden	2011	BMI Epidemiology Study	Community	urban	9		1412		
3389	Sweden	2011	Effects of the COVID-19 Pandemic on Children's Body Mass Index Trajectories in Sweden	Subnational	both	6-16	6-16	3255	3005	
3390	Sweden	2011-2014	PIVUS Study	Community	urban	80	80	909	903	
3391	Sweden	2011-2014	EpiHealth	National	both	45-75	45-75	4731	6054	
3392	Sweden	2012	BMI Epidemiology Study	Community	urban	7		1551		
3393	Sweden	2012	Effects of the COVID-19 Pandemic on Children's Body Mass Index Trajectories in Sweden	Subnational	both	6-16	6-16	3196	2936	
3394	Sweden	2013	BMI Epidemiology Study	Community	urban	7-8		2875		
3395	Sweden	2013	Effects of the COVID-19 Pandemic on Children's Body Mass Index Trajectories in Sweden	Subnational	both	6-16	6-16	3160	2835	
3396	Sweden	2013-2015	Uppsala Longitudinal Study of Adult Men	Community	both	89-94		119		
3397	Sweden	2014	BMI Epidemiology Study	Community	urban	7-9		4251		
3398	Sweden	2014	Effects of the COVID-19 Pandemic on Children's Body Mass Index Trajectories in Sweden	Subnational	both	6-16	6-16	2709	2565	
3399	Sweden	2014	MONICA Northern Sweden	Subnational	both	25-74	25-74	753	795	
3400	Sweden	2014-2016	Swedish INTERGENE Cohort Study	Subnational	urban	37-88	37-88	602	653	
3401	Sweden	2015	BMI Epidemiology Study	Community	urban	8-9		1917		
3402	Sweden	2015	Effects of the COVID-19 Pandemic on Children's Body Mass Index Trajectories in Sweden	Subnational	both	6-16	6-16	2933	2699	
3403	Sweden	2015-2016	Childhood Obesity Surveillance Initiative 4	National	both	6-9	6-9	4070	3798	
3404	Sweden	2016	BMI Epidemiology Study	Community	urban	9		35		
3405	Sweden	2016	Effects of the COVID-19 Pandemic on Children's Body Mass Index Trajectories in Sweden	Subnational	both	6-16	6-16	4140	3856	
3406	Sweden	2016-2017	Population Study of Women in Gothenburg	Community	urban		38-50		570	
3407	Sweden	2017	Effects of the COVID-19 Pandemic on Children's Body Mass Index Trajectories in Sweden	Subnational	both	6-16	6-16	3278	3169	
3408	Sweden	2018	Effects of the COVID-19 Pandemic on Children's Body Mass Index Trajectories in Sweden	Subnational	both	6-16	6-16	4473	4238	
3409	Sweden	2019	Childhood Obesity Surveillance Initiative 5	National	both	6-9	6-9	31642	30002	
3410	Sweden	2019	Effects of the COVID-19 Pandemic on Children's Body Mass Index Trajectories in Sweden	Subnational	both	6-16	6-16	4177	3915	
3411	Sweden	2020	Effects of the COVID-19 Pandemic on Children's Body Mass Index Trajectories in Sweden	Subnational	both	6-16	6-16	3394	3270	
3412	Sweden	2021	Effects of the COVID-19 Pandemic on Children's Body Mass Index Trajectories in Sweden	Subnational	both	6-16	6-16	3454	3255	
3413	Sweden	2022	Childhood Obesity Surveillance Initiative 6	National	both	6-9	6-9	54895	51448	

	Country	Study years	Survey/Study name/Citation	Level of representative-ness	Rural, urban, or both	Age range as in NCD-RisC database		Sample size		Note
						Male	Female	Male	Female	
3414	Sweden	2022	Effects of the COVID-19 Pandemic on Children's Body Mass Index Trajectories in Sweden	Subnational	both	6-16	6-16	3844	3609	
3415	Sweden	2022	MONICA Northern Sweden	Subnational	both	25-75	25-75	451	565	
3416	Sweden	2023	Effects of the COVID-19 Pandemic on Children's Body Mass Index Trajectories in Sweden	Subnational	both	6-16	6-16	3781	3401	
3417	Sweden	2024	Effects of the COVID-19 Pandemic on Children's Body Mass Index Trajectories in Sweden	Subnational	both	6-16	6-16	3681	3416	
3418	Switzerland	1984-1986	The Swiss MONICA Study Wave I	Subnational	both	25-74	25-74	1744	1689	
3419	Switzerland	1988-1989	The Swiss MONICA Study Wave II	Subnational	both	25-74	25-74	1778	1684	
3420	Switzerland	1992-1993	The Swiss MONICA Study Wave III	Subnational	both	25-74	25-74	1577	1672	
3421	Switzerland	1995	Bus Santé Study	Subnational	urban	30-80	30-80	446	457	
3422	Switzerland	1996	Bus Santé Study	Subnational	urban	30-80	30-80	575	573	
3423	Switzerland	1997	Bus Santé Study	Subnational	urban	30-80	20-80	553	606	
3424	Switzerland	1998	Bus Santé Study	Subnational	urban	30-80	30-80	594	581	
3425	Switzerland	1999	Bus Santé Study	Subnational	urban	30-80	30-80	608	561	
3426	Switzerland	2000	Bus Santé Study	Subnational	urban	30-80	30-80	559	591	
3427	Switzerland	2001	Bus Santé Study	Subnational	urban	30-80	30-80	636	605	
3428	Switzerland	2002	Prevalence of overweight and obesity in 6-12-year old children in Switzerland	National	both	6-12	6-12	1196	1235	
3429	Switzerland	2002	Bus Santé Study	Subnational	urban	30-80	30-80	641	622	
3430	Switzerland	2003	Bus Santé Study	Subnational	urban	30-80	30-80	616	619	
3431	Switzerland	2003-2006	Cohorte Lausannoise	Community	urban	35-75	35-75	3186	3536	
3432	Switzerland	2004	Bus Santé Study	Subnational	urban	30-80	30-80	517	534	
3433	Switzerland	2004	The Swiss Conscription Database	National	both	18-20		20491		
3434	Switzerland	2005	Kinder- und Jugendsportstudie (KISS)	Subnational	both	6-13	6-13	239	256	
3435	Switzerland	2005	Bus Santé Study	Subnational	urban	30-80	30-80	102	121	
3436	Switzerland	2005	The Swiss Conscription Database	National	both	18-20		32131		
3437	Switzerland	2005-2006	Chiolero et al., J Hypertens 25(11):2209-17, 2007	Subnational	both	10-14	10-14	2621	2586	
3438	Switzerland	2005-2006	BMI Monitoring for Switzerland - Study 1	Community	urban	5-11	5-11	4477	4103	35
3439	Switzerland	2006	Kinder- und Jugendsportstudie (KISS)	Subnational	both	7-14	7-14	100	105	
3440	Switzerland	2006	Bus Santé Study	Subnational	urban	30-80	30-80	136	127	
3441	Switzerland	2006	The Swiss Conscription Database	National	both	18-20		34530		
3442	Switzerland	2006-2007	BMI Monitoring for Switzerland - Study 1	Community	urban	5-11	5-11	4061	3896	35
3443	Switzerland	2007	Prevalence of overweight and obesity in 6-12-year old children in Switzerland	National	both	6-12	6-12	1082	1136	
3444	Switzerland	2007	Bus Santé Study	Subnational	urban	30-80	30-80	133	131	
3445	Switzerland	2007	The Swiss Conscription Database	National	both	18-20		36194		
3446	Switzerland	2007-2008	BMI Monitoring for Switzerland - Study 1	Community	urban	5-11	5-11	4067	4009	35
3447	Switzerland	2008	Bus Santé Study	Subnational	urban	20-74	20-74	249	250	
3448	Switzerland	2008	The Swiss Conscription Database	National	both	18-20		34497		
3449	Switzerland	2008-2009	BMI Monitoring for Switzerland - Study 1	Community	urban	5-11	5-11	3998	3847	35
3450	Switzerland	2008-2010	BMI Monitoring for Switzerland - Study 2	Subnational	both	6	6	1048	1110	
3451	Switzerland	2009	Kinder- und Jugendsportstudie (KISS)	Subnational	both	10-17	10-17	44	65	
3452	Switzerland	2009	Bus Santé Study	Subnational	urban	20-74	20-74	512	564	
3453	Switzerland	2009	The Swiss Conscription Database	National	both	18-20		34896		
3454	Switzerland	2009-2010	BMI Monitoring for Switzerland - Study 1	Community	urban	5-11	5-11	4051	3913	35
3455	Switzerland	2009-2012	Cohorte Lausannoise	Community	urban	40-75	40-75	2176	2494	
3456	Switzerland	2010	EuropeaN Energy balance Research to prevent excessive weight Gain among Youth - The ENERGY-project	Subnational	both	10-12	10-12	287	270	
3457	Switzerland	2010	Bus Santé Study	Subnational	urban	30-80	30-80	498	518	
3458	Switzerland	2010	The Swiss Conscription Database	National	both	18-20		37214		
3459	Switzerland	2010-2011	BMI Monitoring for Switzerland - Study 1	Community	urban	5-11	5-11	4092	3734	35
3460	Switzerland	2010-2013	BMI Monitoring for Switzerland - Study 2	Subnational	both	5-11	5-11	9735	9346	
3461	Switzerland	2011	Bus Santé Study	Subnational	urban	30-80	30-80	458	482	
3462	Switzerland	2011	The Swiss Conscription Database	National	both	18-20		38108		
3463	Switzerland	2011-2012	BMI Monitoring for Switzerland - Study 1	Community	urban	5-11	5-11	4085	3940	35
3464	Switzerland	2012	Prevalence of overweight and obesity in 6-12-year old children in Switzerland	National	both	6-12	6-12	1499	1464	
3465	Switzerland	2012	Bus Santé Study	Subnational	urban	20+	20+	497	498	
3466	Switzerland	2012	The Swiss Conscription Database	National	both	18-20		36938		
3467	Switzerland	2012-2013	BMI Monitoring for Switzerland - Study 1	Community	urban	5-11	5-11	4224	4042	35
3468	Switzerland	2013	Bus Santé Study	Subnational	urban	19+	19+	504	556	
3469	Switzerland	2013	The Swiss Conscription Database	National	both	18-20		32890		
3470	Switzerland	2013-2014	BMI Monitoring for Switzerland - Study 1	Community	urban	5-11	5-11	4333	4073	35
3471	Switzerland	2014	Bus Santé Study	Subnational	urban	20-80	20-80	496	541	
3472	Switzerland	2014	The Swiss Conscription Database	National	both	18-20		32691		
3473	Switzerland	2014-2015	National Nutrition Survey menuCH	National	both	18-75	18-75	937	1107	
3474	Switzerland	2014-2015	BMI Monitoring for Switzerland - Study 1	Community	urban	5-11	5-11	4657	4625	35
3475	Switzerland	2014-2016	BMI Monitoring for Switzerland - Study 2	Subnational	both	5-11	5-11	7759	7343	
3476	Switzerland	2014-2017	Cohorte Lausannoise	Community	urban	45-87	45-87	2008	2473	

	Country	Study years	Survey/Study name/Citation	Level of representative-ness	Rural, urban, or both	Age range as in NCD-RisC database		Sample size		Note
						Male	Female	Male	Female	
3477	Switzerland	2015	Bus Santé Study	Subnational	urban	20-80	20-80	560	585	
3478	Switzerland	2015	The Swiss Conscription Database	National	both	18-20		32616		
3479	Switzerland	2015-2016	BMI Monitoring for Switzerland - Study 1	Community	urban	5-11	5-11	3326	3058	35
3480	Switzerland	2016	Bus Santé Study	Subnational	urban	20-80	20-80	497	528	
3481	Switzerland	2016	The Swiss Conscription Database	National	both	18-20		26716		
3482	Switzerland	2016-2017	BMI Monitoring for Switzerland - Study 1	Community	urban	5-11	5-11	3457	3121	35
3483	Switzerland	2017	Bus Santé Study	Subnational	urban	20+	20+	587	639	
3484	Switzerland	2017	The Swiss Conscription Database	National	both	18-20		25509		
3485	Switzerland	2017-2018	National Studie Gesundheit und Ernährung von Primarschülern (CHildHNS)	National	both	6-12	6-12	1135	1144	
3486	Switzerland	2017-2018	BMI Monitoring for Switzerland - Study 1	Community	urban	5-11	5-11	6044	5788	35
3487	Switzerland	2018	Bus Santé Study	Subnational	urban	20-80	19-80	556	597	
3488	Switzerland	2018	The Swiss Conscription Database	National	both	18-20		20906		
3489	Switzerland	2018-2019	BMI Monitoring for Switzerland - Study 1	Community	urban	5-11	5-11	6693	6370	35
3490	Switzerland	2018-2020	BMI Monitoring for Switzerland - Study 2	Subnational	both	5-11	5-11	9161	8694	
3491	Switzerland	2018-2021	Cohorte Lausannoise	Community	urban	49-90	49-90	1600	1946	
3492	Switzerland	2018-2023	Cohorte Lausannoise Offspring	Community	urban	18-35	18-35	394	398	
3493	Switzerland	2019	Bus Santé Study	Subnational	urban	20-80	20-80	556	625	
3494	Switzerland	2019	The Swiss Conscription Database	National	both	18-20		18529		
3495	Switzerland	2019-2020	BMI Monitoring for Switzerland - Study 1	Community	urban	5-11	5-11	5873	5453	35
3496	Switzerland	2020	Bus Santé Study	Subnational	urban	20-80	20-80	105	130	
3497	Switzerland	2020	The Swiss Conscription Database	National	both	18-20		12531		
3498	Switzerland	2020-2021	BMI Monitoring for Switzerland - Study 1	Community	urban	5-11	5-11	5697	5586	35
3499	Switzerland	2021	The Swiss Conscription Database	National	both	18-20		4384		
3500	Switzerland	2021-2022	BMI Monitoring for Switzerland - Study 1	Community	urban	5-11	5-11	5458	5291	35
3501	Switzerland	2022	The Swiss Conscription Database	National	both	18-20		4732		
3502	Syrian Arab Republic	2002	National survey on non-communicable diseases and factors affecting their development	National	both	15-64	15-64	3155	4045	
3503	Taiwan	1985	INTERSALT	Community	rural	20-59	20-59	89	92	
3504	Taiwan	1989-1991	Chiu et al., J Gerontol A Biol Sci Med Sci 55(11):M684-90, 2000	Subnational	both	65+	65+	1322	1308	
3505	Taiwan	1993-1994	The Kinmen Neurological Disorders Survey	Community	urban	50+	50+	672	593	
3506	Taiwan	1993-1996	Nutrition and Health Survey in Taiwan	National	both	5+	5+	2959	3216	
3507	Taiwan	1999-2000	Nutrition and Health Survey in Taiwan	National	both	65+	65+	1271	1202	
3508	Taiwan	2000	Social Environment and Biomarkers of Aging Study	National	both	50+	50+	590	433	
3509	Taiwan	2001-2002	Nutrition and Health Survey in Taiwan	National	both	6-12	6-12	1334	1139	
3510	Taiwan	2004-2005	Taichung Community Health Study (TCHS)	Community	urban	40+	40+	1147	1212	
3511	Taiwan	2005-2008	Nutrition and Health Survey in Taiwan	National	both	19+	19+	1311	1355	
3512	Taiwan	2006	Social Environment and Biomarkers of Aging Study	National	both	53+	53+	548	476	
3513	Taiwan	2007	Taiwanese Survey on Hypertension, Hyperglycemia and Hyperlipidemia	National	both	20+	20+	2155	2490	
3514	Taiwan	2010	Nutrition and Health Survey in Taiwan	National	both	13-15	13-15	852	927	
3515	Taiwan	2011	Nutrition and Health Survey in Taiwan	National	both	16-18	16-18	580	591	
3516	Taiwan	2012	Global School-based Student Health Survey	National	both	13-17	13-17	2998	2927	
3517	Taiwan	2012	Nutrition and Health Survey in Taiwan	National	both	7-12	7-12	510	499	
3518	Taiwan	2013	Global School-based Student Health Survey	National	both	13-17	13-17	2030	1664	
3519	Taiwan	2013-2016	Nutrition and Health Survey in Taiwan	National	both	5+	5+	1505	1583	
3520	Taiwan	2014	Global School-based Student Health Survey	National	both	12-17	12-17	2705	2434	
3521	Taiwan	2015	Global School-based Student Health Survey	National	both	13-17	13-17	1891	1798	
3522	Taiwan	2016	Global School-based Student Health Survey	National	both	12-17	12-17	2570	2445	
3523	Taiwan	2017	Global School-based Student Health Survey	National	both	13-17	13-17	1583	2003	
3524	Taiwan	2017-2020	Nutrition and Health Survey in Taiwan	National	both	5+	5+	2199	2195	
3525	Taiwan	2018	Global School-based Student Health Survey	National	both	12-17	12-17	2618	2414	
3526	Taiwan	2019	Global School-based Student Health Survey	National	both	15-17	15-17	1701	1854	
3527	Tajikistan	2003	Micronutrient Status Survey	National	both		15-49		2044	
3528	Tajikistan	2012	DHS	National	both		15-49		8930	
3529	Tajikistan	2015-2016	Childhood Obesity Surveillance Initiative 4	National	both	7	7	1438	1457	
3530	Tajikistan	2016	National Micronutrient Status Survey in Tajikistan	National	both		15-49		2132	
3531	Tajikistan	2016	STEPS	National	both	18-69	18-69	1091	1553	
3532	Tajikistan	2017	DHS	National	both		15-49		9922	
3533	Tajikistan	2019	Childhood Obesity Surveillance Initiative 5	National	both	7-8	7-8	1809	1622	
3534	Tajikistan	2023	STEPS	National	both	18-69	18-69	767	1683	
3535	Tanzania	1991-1992	DHS	National	both		20-49		4039	
3536	Tanzania	1996	DHS	National	both		20-49		3512	
3537	Tanzania	1996-1997	Aspray et al., Trans R Soc Trop Med Hyg 94:637-44, 2000	Community	rural	15+	15+	251	324	
3538	Tanzania	1996-1997	Aspray et al., Trans R Soc Trop Med Hyg 94:637-44, 2000	Community	urban	15+	15+	117	118	
3539	Tanzania	1998-1999	Bovet et al., Int J Epidemiol 31(1):240-7, 2002	Community	urban	25-64	25-64	3593	5646	

	Country	Study years	Survey/Study name/Citation	Level of representative-ness	Rural, urban, or both	Age range as in NCD-RisC database		Sample size		Note
						Male	Female	Male	Female	
3540	Tanzania	2003-2004	Rural-urban disparities in the nutritional status of younger adolescents in Tanzania	Community	rural	10-19	10-19	375	312	
3541	Tanzania	2004-2005	DHS	National	both		15-49		9160	
3542	Tanzania	2008-2009	The Tanzania National Panel Survey (TZNPS)	National	both	5+	5+	5659	6465	
3543	Tanzania	2009	Ilembula School Study	Community	rural	8-9	8-9	151	150	
3544	Tanzania	2010	DHS	National	both		15-49		9099	
3545	Tanzania	2010-2011	The Tanzania National Panel Survey (TZNPS)	National	both	5+	5+	6585	7617	
3546	Tanzania	2011	STEPS	Subnational	both	25-64	25-64	1008	1517	
3547	Tanzania	2012	STEPS	National	both	25-64	25-64	2581	2827	
3548	Tanzania	2012-2013	The Tanzania National Panel Survey (TZNPS)	National	both	5+	5+	7399	8952	
3549	Tanzania	2014	Dar es Salaam Urban Cohort Hypertension Study	Community	urban	40+	40+	965	1266	
3550	Tanzania	2014-2015	The Tanzania National Panel Survey (TZNPS)	National	both	5-15	5-49	1790	5005	
3551	Tanzania	2015-2016	DHS	National	both		15-49		12036	
3552	Tanzania	2017	ARISE Network Adolescent Health Study (Dodoma)	Community	rural	10-19	10-19	471	585	
3553	Tanzania	2019-2020	The Tanzania Extended National Panel Survey (TZENPS)	National	both	5-15	5-49	514	1706	
3554	Tanzania	2020-2022	The Tanzania National Panel Survey (TZNPS)	National	both	5-15	5-49	2256	6285	
3555	Tanzania	2022	DHS	National	both		15-49		7021	
3556	Tanzania	2023	STEPS	National	both	18-69	18-69	1448	1927	
3557	Thailand	1987	INCLEN	Community	rural	35-65		244		
3558	Thailand	1989	INCLEN	Community	rural	35-65		209		
3559	Thailand	1989	INCLEN	Community	urban	35-65		207		
3560	Thailand	1991	Thailand National Health Examination Survey I	National	both	5+	5+	8698	11027	
3561	Thailand	1995	The Fourth National Nutrition Survey of Thailand- 1995	National	both	20-60	20-60	1405	3631	
3562	Thailand	1997	Thailand National Health Examination Survey II	National	both	5-59	5-59	4117	4876	
3563	Thailand	2000	InterASIA	National	both	35+	35+	2092	3211	
3564	Thailand	2003	Socio Fitness of Thai Children	National	rural	6-12	6-12	1180	1023	
3565	Thailand	2003	Socio Fitness of Thai Children	National	urban	6-12	6-12	2010	1722	
3566	Thailand	2003	The Fifth National Nutrition Survey of Thailand	National	both	19-74	19-74	1960	3366	
3567	Thailand	2004	Thailand National Health Examination Survey III	National	both	15+	15+	18819	20143	
3568	Thailand	2006-2007	Health Checks Ubon Ratchathani (HCUR) Study	Subnational	both	15+	15+	361554	388898	
3569	Thailand	2009	Thailand National Health Examination Survey IV	National	both	18+	18+	12970	13838	
3570	Thailand	2011	SEANUTS	National	both	5-12	5-12	922	939	
3571	Thailand	2013-2014	The Metabolic Syndrome and Health Behaviours in School Children Aged 13-16 Years in Ubon Ratchathani	Community	both	13-17	13-17	98	278	
3572	Thailand	2014	Thailand National Health Examination Survey V (10-17)	National	rural	10-17	10-17	875	872	
3573	Thailand	2014	Thailand National Health Examination Survey V (10-17)	National	urban	10-17	10-17	657	705	
3574	Thailand	2014	Thailand National Health Examination Survey V (18+)	National	both	18+	18+	7721	10567	
3575	Thailand	2015	Global School-based Student Health Survey	National	both	12-17	12-17	2240	3106	
3576	Thailand	2019-2020	Thailand National Health Examination Survey VI	National	both	10+	10+	10732	14197	
3577	Thailand	2021	Global School-based Student Health Survey	National	both	12-16	12-17	1911	2703	
3578	Timor-Leste	2009-2010	DHS	National	both		15-49		11983	
3579	Timor-Leste	2009-2010	Timor-Leste Eye Health Survey	Subnational	both	40+	40+	245	247	
3580	Timor-Leste	2013	Child measurements in Ossu and in Natarbora, Timor Leste - Harvest season	Subnational	rural	5-19	5-19	245	239	
3581	Timor-Leste	2014	STEPS	National	both	18-69	18-69	1048	1437	
3582	Timor-Leste	2016	DHS	National	both	15-59	15-49	4556	11823	
3583	Timor-Leste	2018	Child measurements in Ossu and in Natarbora, Timor Leste - Harvest season	Subnational	rural	5-19	5-19	234	246	
3584	Timor-Leste	2018	Child measurements in Ossu and in Natarbora, Timor Leste - Post rainy season	Subnational	rural	5-19	5-19	97	132	
3585	Timor-Leste	2020	Timor-Leste Food and Nutrition Survey (TLFNS)	National	both		15-49		12380	
3586	Timor-Leste	2023	Child measurements in Ossu, Natarbora, and Atauro, Timor Leste - Harvest season	Subnational	rural	5-18	5-18	288	320	
3587	Timor-Leste	2023	Child measurements in Ossu, Natarbora, and Atauro, Timor Leste - Post rainy season	Subnational	rural	5-18	5-18	268	281	
3588	Timor-Leste	2023	STEPS	National	both	18-69	18-69	1412	2018	
3589	Timor-Leste	2024	Child measurements in Ossu, Natarbora, and Atauro, Timor Leste - Harvest season	Subnational	rural	5-18	5-18	246	292	
3590	Timor-Leste	2024	Child measurements in Ossu, Natarbora, and Atauro, Timor Leste - Post rainy season	Subnational	rural	5-18	5-18	303	325	
3591	Togo	1998	DHS	National	both		20-49		3114	
3592	Togo	2010	STEPS	National	both	15-64	15-64	2063	2095	
3593	Togo	2013-2014	DHS	National	both		15-49		4398	
3594	Togo	2014	Impact evaluation of a cash transfer program in North Togo	Subnational	rural		20-65		3588	
3595	Togo	2021-2022	STEPS	National	both	18-69	18-69	1585	2165	
3596	Tokelau	2005	STEPS	National	both	15-64	15-64	270	296	
3597	Tokelau	2014	STEPS	National	both	18-64	18-64	261	276	
3598	Tonga	2004	STEPS	National	both	15-64	15-64	403	552	
3599	Tonga	2005-2007	Pacific Obesity Prevention in Communities - Ma’alahi Youth Project	Subnational	rural	11-19	11-19	1206	1445	
3600	Tonga	2007-2008	Pacific Obesity Prevention in Communities - Ma’alahi Youth Project	Subnational	rural	13-22	13-22	434	579	
3601	Tonga	2010	Global School-based Student Health Survey	National	both	13-17	13-17	926	1069	
3602	Tonga	2011	STEPS	National	both	15-64	15-64	878	1401	

	Country	Study years	Survey/Study name/Citation	Level of representative-ness	Rural, urban, or both	Age range as in NCD-RisC database		Sample size		Note
						Male	Female	Male	Female	
3603	Tonga	2017	Global School-based Student Health Survey	National	both	12-17	12-17	1254	1452	
3604	Tonga	2017	STEPS	National	both	18-69	18-69	1260	2327	
3605	Trinidad and Tobago	1985	INTERSALT	Community	urban	20-59	20-59	84	92	
3606	Trinidad and Tobago	1999	Child Health Survey	National	both	5-9	5-9	3060	3272	
3607	Trinidad and Tobago	2001	Adult Survey	National	rural	25+	25+	198	267	
3608	Trinidad and Tobago	2003	Child Health Survey	National	both	5-9	5-9	1832	1974	
3609	Trinidad and Tobago	2003	National Survey of Senior School Health	National	both	15-16	15-16	828	1112	
3610	Trinidad and Tobago	2011	STEPS	National	both	15-64	15-64	1112	1608	
3611	Trinidad and Tobago	2013-2014	National Eye Survey	National	both	40+	40+	1077	1396	
3612	Trinidad and Tobago	2024	STEPS	National	both	18-69	18-69	1712	2085	
3613	Tunisia	1996-1997	Tunisian National Nutrition Survey 1996-1997	National	both	5+	5+	2724	4125	
3614	Tunisia	1996-1997	Ariana Healthy Project 1997	Community	both	35-65	35-65	2645	2701	
3615	Tunisia	2005	Aounallah et al., Public Health 12(1):98, 2012	National	both	15-19	15-19	1290	1566	
3616	Tunisia	2005	Tunisian National Survey 2005 (TAHINA)	National	both	35-71	35-71	3265	4313	
3617	Tunisia	2009-2010	ObeMaghreb	Subnational	urban	5-49	5-49	1841	1601	
3618	Tunisia	2020	School and Community Drivers of Child Diets in Arab Cities: Identifying Levers for Intervention (SCALE)	Subnational	urban	9-11	9-11	1221	1232	
3619	Türkiye	1990	Turkish Adult Risk Factor Study	National	both	20+	20+	1338	1369	
3620	Türkiye	1993	DHS	National	both		20-49		2294	
3621	Türkiye	1995	Turkish Adult Risk Factor Study	National	both	25+	25+	855	878	
3622	Türkiye	1998	DHS	National	both		20-49		2210	
3623	Türkiye	1998	Turkish Adult Risk Factor Study	National	both	28+	28+	877	909	
3624	Türkiye	1998-1999	Erem et al., Diabetes Res Clin Pract 54(3):203-08, 2001	Community	urban	20+	20+	1324	1322	
3625	Türkiye	2000	Turkish Adult Risk Factor Study	National	both	30+	30+	890	938	
3626	Türkiye	2000	Manisa Demographic and Health Survey	Subnational	urban		15-49		1420	
3627	Türkiye	2000-2002	The Healthy Nutrition for Healthy Heart Study	National	both	25-84	25-84	4718	10631	
3628	Türkiye	2001	Yumuk et al., Diabetes Res Clin Pract 70(2):151-58, 2005	Community	urban	20+	20+	1042	1789	
3629	Türkiye	2001-2002	Turkish Adult Risk Factor Study	National	both	32+	32+	1098	1209	
3630	Türkiye	2002	Onal et al., Blood Press 13(1):31-6, 2004	Subnational	urban	25+	25+	67	355	
3631	Türkiye	2003	DHS	National	both		20-49		2934	
3632	Türkiye	2003	Prevalence, awareness, treatment and control of hypertension in Turkey in 2003	National	both	18+	18+	1988	2847	
3633	Türkiye	2003-2004	Turkish Adult Risk Factor Study	National	both	34+	34+	1097	1130	
3634	Türkiye	2003-2005	Prevalence of prehypertension and associated risk factors among Turkish adults: Trabzon Hypertension Study	Subnational	both	20+	20+	2208	2601	
3635	Türkiye	2004	Nationally Representative Cross-sectional Survey	National	both	20+	20+	2110	2154	
3636	Türkiye	2005-2006	Turkish Adult Risk Factor Study	National	both	35+	35+	965	1029	
3637	Türkiye	2007	Natinal Household survey	National	both	20-85	20-85	2263	1842	
3638	Türkiye	2007-2008	Turkish Adult Risk Factor Study	National	both	37+	37+	1048	1070	
3639	Türkiye	2007-2009	Balcova Heart Study	Community	urban	30+	30+	4274	8609	
3640	Türkiye	2008	DHS	National	both		15-49		6167	
3641	Türkiye	2009-2010	Turkish Adult Risk Factor Study	National	both	39+	39+	462	501	
3642	Türkiye	2009-2012	Prevalence of diabetes and associated risk factors among adult population in Trabzon city	Subnational	both	20+	20+	1562	2115	
3643	Türkiye	2011	Chronic Diseases and Risk Factors Survey in Turkey	National	both	15+	15+	8058	8920	
3644	Türkiye	2012-2013	Turkish Adult Risk Factor Study	National	both	37+	37+	1012	1087	
3645	Türkiye	2013	DHS	National	both		15-49		8270	
3646	Türkiye	2013	Childhood Obesity Surveillance Initiative 3	National	both	7-8	7-8	2483	2475	
3647	Türkiye	2014-2015	Turkish Adult Risk Factor Study	National	both	44+	44+	437	484	
3648	Türkiye	2015-2016	Childhood Obesity Surveillance Initiative 4	National	both	6-7	6-7	5479	5336	
3649	Türkiye	2016-2017	Erasmus plus KA2, Healthyland	Community	urban	6	6	29	22	
3650	Türkiye	2017	STEPS	National	both	15+	15+	2306	3426	
3651	Türkiye	2017-2018	Erasmus plus KA2, Healthyland	Community	urban	6	6	50	50	
3652	Türkiye	2018	DHS	National	both		15-49		6464	
3653	Türkiye	2022	Childhood Obesity Surveillance Initiative 6	National	both	7	7	5186	5112	
3654	Türkiye	2023	STEPS	National	both	15+	15+	2166	3035	
3655	Turkmenistan	2000	DHS	National	both		15-49		2084	
3656	Turkmenistan	2013	STEPS	National	both	18-64	18-64	1879	2741	
3657	Turkmenistan	2015-2016	Childhood Obesity Surveillance Initiative 4	National	both	7-8	7-8	1952	1956	
3658	Turkmenistan	2018	STEPS	National	both	18-69	18-69	1713	2236	
3659	Tuvalu	2013	Global School-based Student Health Survey	National	both	13-17	13-17	210	215	
3660	Tuvalu	2015	STEPS	National	both	18-69	18-69	478	550	
3661	Uganda	1995	DHS	National	both		20-49		2831	
3662	Uganda	2000-2001	DHS	National	both		15-49		5829	
3663	Uganda	2006	DHS	National	both	15-54	15-49	2475	2538	
3664	Uganda	2011	DHS	National	both	15-54	15-49	2361	2501	
3665	Uganda	2011-2012	The Prevalence and Distribution of Non-communicable Diseases and their Risk Factors in Kasese District, Uganda	Subnational	both	25-79	25-79	277	221	

	Country	Study years	Survey/Study name/Citation	Level of representative-ness	Rural, urban, or both	Age range as in NCD-RisC database		Sample size		Note
						Male	Female	Male	Female	
3666	Uganda	2011-2013	Gulu Health and Demographic Surveillance Site (HDSS)	Community	rural	5+	5+	3938	4820	
3667	Uganda	2012	Prevalence, awareness and control of hypertension in Uganda	Subnational	both	15+	15+	1619	2740	
3668	Uganda	2014	STEPS	National	both	18-69	18-69	1560	2120	
3669	Uganda	2014-2015	Gulu Health and Demographic Surveillance Site (HDSS)	Community	rural	15-24	15-24	671	517	
3670	Uganda	2015	HopeNet	Community	rural	18+	18+	314	503	
3671	Uganda	2016	DHS	National	both	15-54	15-54	5191	5415	
3672	Uganda	2017	HopeNet	Community	rural	18+	18+	188	289	
3673	Uganda	2017-2018	Uganda National Panel Survey 2017-2018	National	both	5+	5+	3902	5029	
3674	Uganda	2018-2019	Scaling up Packages of Intervention for Cardiovascular disease prevention in selected sites in Europe and Sub-Saharan Africa (SPICES)	Subnational	both	25-70	25-70	1740	2479	
3675	Uganda	2019	HopeNet	Community	rural	18+	18+	287	475	
3676	Uganda	2019-2020	Uganda National Panel Survey 2019-2020	National	both	5+	5+	3425	4506	
3677	Uganda	2023	STEPS	National	both	20-69	20-69	1347	1975	
3678	Uganda	2023	Estimating the Knowledge and Health Impact of Viamo's 3-2-1 Service	Subnational	both		18+		4203	
3679	Ukraine	2002	National Micronutrient Survey	National	both		15-50		816	
3680	Ukraine	2006-2007	Physical Development of School Children Ukraine	Community	rural	6-17	6-17	1226	1336	
3681	Ukraine	2007-2008	Physical Development of School Children Ukraine	Community	urban	6-17	6-17	1224	1126	
3682	Ukraine	2012	Epidemiological aspects of obesity and systemic hypertension among school children of Western Ukraine	Community	urban	10-17	10-17	271	257	
3683	Ukraine	2013-2014	The prevalence of underweight, overweight and obesity in children and adolescents from Ukraine	National	both	6-18	6-18	6596	7143	
3684	Ukraine	2018	Prevalence of obesity in Ukrainian children and adolescents	National	both	7-17	7-17	4471	4667	
3685	Ukraine	2018-2020	Physical development of children 3-6 years old - Kyiv	Community	urban	5-6	5-6	407	334	
3686	Ukraine	2019	Prevalence of obesity in Ukrainian children and adolescents	National	both	7-17	7-17	4245	4760	
3687	Ukraine	2019	STEPS	National	both	18-69	18-69	1569	2600	
3688	Ukraine	2023-2024	Childhood Obesity Surveillance Initiative 6	National	both	6-8	6-8	1816	1710	
3689	United Arab Emirates	1989-1990	El Mugamer et al., J Trop Med Hyg 98(6):407-15, 1995	Community	both	20+	20+	122	197	
3690	United Arab Emirates	1999-2000	Emirates National Diabetes and Coronary Artery Disease Risk Factor Study	National	both	20-80	20-80	2822	3743	
3691	United Arab Emirates	2000-2001	Carter et al., J Health Popul Nutr 22(1):75-83, 2004	Community	both		20-79		521	
3692	United Arab Emirates	2005	Global School-based Student Health Survey	National	both	12-15	12-15	5595	6268	
3693	United Arab Emirates	2009	Gulf Cooperation Council World Health Survey	National	both	18+	18+	605	645	
3694	United Arab Emirates	2010	Global School-based Student Health Survey	National	both	13-17	13-17	948	1257	
3695	United Arab Emirates	2016	Global School-based Student Health Survey	National	both	12-17	12-17	2323	2674	
3696	United Arab Emirates	2017-2018	STEPS	National	both	18+	18+	2148	2323	
3697	United Kingdom	1980	British Cohort Study 1970	National	both	10	10	6252	5907	
3698	United Kingdom	1982	MRC National Survey of Health and Development	National	both	36-37	36-37	1632	1648	
3699	United Kingdom	1983-1984	MONICA, Belfast	Subnational	both	25-64	25-64	1158	1183	
3700	United Kingdom	1984-1986	Scottish Heart Health Survey	Subnational	both	40-59	40-59	4364	4465	
3701	United Kingdom	1985	INTERSALT, Birmingham	Community	urban	20-59	20-59	100	100	
3702	United Kingdom	1985	INTERSALT, South Wales	Community	urban	20-59	20-59	100	99	
3703	United Kingdom	1985-1986	INTERSALT, Belfast	Community	urban	20-59	20-59	99	100	
3704	United Kingdom	1986	British Cohort Study 1970	National	both	16	16	2773	2950	
3705	United Kingdom	1986-1987	Dietary and Nutritional Survey of British Adults 1986-1987	National	both	16-64	16-64	1158	1161	
3706	United Kingdom	1986-1987	MONICA, Belfast	Subnational	both	25-64	25-64	1155	1185	
3707	United Kingdom	1987-1988	Edinburgh Artery Study	Community	urban	54-75	54-75	808	783	
3708	United Kingdom	1989	MRC National Survey of Health and Development	National	both	42-44	42-44	1617	1608	
3709	United Kingdom	1991	National Child Development Study (1958 British Cohort Study)	National	both	33	33	5426	5605	
3710	United Kingdom	1991-1992	Health Survey for England	National	both	16+	16+	3114	3430	
3711	United Kingdom	1991-1992	MONICA, Belfast	Subnational	both	25-64	25-64	998	996	
3712	United Kingdom	1992	MONICA, Glasgow	Community	urban	25-64	25-64	696	775	
3713	United Kingdom	1992-1993	Whickham Survey	Community	urban	35+	35+	676	784	
3714	United Kingdom	1992-1994	Edinburgh Artery Study	Community	urban	60-81	60-81	580	582	
3715	United Kingdom	1993	Health Survey for England	National	both	16+	16+	7461	8297	
3716	United Kingdom	1993-1997	EPIC Norfolk	Subnational	both	40-79	40-79	11574	13995	
3717	United Kingdom	1994	Health Survey for England	National	both	16+	16+	6825	7939	
3718	United Kingdom	1994-1995	Hertfordshire Ageing Study	Subnational	both	63-73	63-73	411	304	
3719	United Kingdom	1994-1995	National Diet and Nutrition Survey (NDNS)	National	both	65+	65+	701	687	
3720	United Kingdom	1995	Health Survey for England	National	both	5+	5+	8038	9027	
3721	United Kingdom	1995	Scottish Health Survey (SHeS)	Subnational	both	16-64	16-64	3303	4005	
3722	United Kingdom	1995	MONICA, Glasgow	Community	urban	25-64	25-64	855	958	
3723	United Kingdom	1996	Health Survey for England	National	both	5+	5+	8469	9461	
3724	United Kingdom	1996	British Cohort Study 1970	National	both	26	26	81	78	
3725	United Kingdom	1997	Health Survey for England	National	both	5+	5+	6285	6841	
3726	United Kingdom	1997	National Diet and Nutrition Survey (NDNS)	National	both	5-18	5-18	933	896	
3727	United Kingdom	1997-1999	INTERMAP, West Bromwich	Community	urban	40-59	40-59	141	138	

	Country	Study years	Survey/Study name/Citation	Level of representative-ness	Rural, urban, or both	Age range as in NCD-RisC database		Sample size		Note
						Male	Female	Male	Female	
3728	United Kingdom	1998	Health Survey for England	National	both	5+	5+	7980	9047	
3729	United Kingdom	1998	Scottish Health Survey (SHeS)	Subnational	both	5-74	5-74	5047	5908	
3730	United Kingdom	1998-1999	INTERMAP, Belfast	Community	urban	40-59	40-59	125	97	
3731	United Kingdom	1998-1999	Avon Longitudinal Study of Parents and Children	Community	both	7	7	3693	3567	
3732	United Kingdom	1998-1999	SportsLinx	Community	urban	9-10	9-10	1429	1364	
3733	United Kingdom	1998-2000	The British Regional Heart Study	National	urban	60-79		4138		
3734	United Kingdom	1998-2002	The Southampton Women's Survey	Community	both		19-36		12414	37
3735	United Kingdom	1999	Health Survey for England	National	both	5+	5+	3880	4304	
3736	United Kingdom	1999	MRC National Survey of Health and Development	National	both	53-54	53-54	1452	1496	
3737	United Kingdom	1999-2000	Avon Longitudinal Study of Parents and Children	Community	both	8	8	3048	3017	
3738	United Kingdom	1999-2000	SportsLinx	Community	urban	9-10	9-10	1469	1439	
3739	United Kingdom	1999-2001	British Women's Heart and Health Study	National	both		60-79		3678	36
3740	United Kingdom	1999-2001	Edinburgh Artery Study	Community	urban	66-87	66-87	373	404	
3741	United Kingdom	1999-2004	Hertfordshire Cohort Study	Subnational	both	59-73	59-73	1571	1416	
3742	United Kingdom	2000	Health Survey for England	National	both	5+	5+	4073	4607	
3743	United Kingdom	2000-2001	Avon Longitudinal Study of Parents and Children	Community	both	9	9	3360	3411	
3744	United Kingdom	2000-2001	SportsLinx	Community	urban	9-10	9-10	1166	1154	
3745	United Kingdom	2000-2001	National Diet and Nutrition Survey (NDNS)	National	both	19-64	19-64	807	973	
3746	United Kingdom	2001	Health Survey for England	National	both	5+	5+	7463	8657	
3747	United Kingdom	2001-2002	Avon Longitudinal Study of Parents and Children	Community	both	10	10	3298	3338	
3748	United Kingdom	2001-2002	SportsLinx	Community	urban	9-10	9-10	866	743	
3749	United Kingdom	2002	Health Survey for England	National	both	5+	5+	6797	7578	
3750	United Kingdom	2002-2003	Avon Longitudinal Study of Parents and Children	Community	both	11	11	3132	3207	
3751	United Kingdom	2002-2003	SportsLinx	Community	urban	9-10	9-10	725	749	
3752	United Kingdom	2003	Health Survey for England	National	both	5+	5+	7136	8268	
3753	United Kingdom	2003	Scottish Health Survey (SHeS)	Subnational	both	5+	5+	3988	4687	
3754	United Kingdom	2003	The European Male Ageing Study	Community	both	40+		394		
3755	United Kingdom	2003-2004	SportsLinx	Community	urban	9-10	9-10	1906	1931	
3756	United Kingdom	2003-2005	Hertfordshire Ageing Study	Subnational	both	72-82	72-82	171	119	
3757	United Kingdom	2004	Health Survey for England	National	both	5+	5+	2975	3608	
3758	United Kingdom	2004-2005	Avon Longitudinal Study of Parents and Children	Community	both	13	13	2939	3016	
3759	United Kingdom	2004-2005	SportsLinx	Community	urban	9-10	9-10	1724	1712	
3760	United Kingdom	2004-2005	English Longitudinal Study of Ageing Wave 2	National	both	52+	52+	3259	3966	
3761	United Kingdom	2005	Health Survey for England	National	both	5+	5+	4839	5503	
3762	United Kingdom	2005-2006	Avon Longitudinal Study of Parents and Children	Community	both	14	14	2699	2761	
3763	United Kingdom	2005-2006	SportsLinx	Community	urban	9-10	9-10	1455	1409	
3764	United Kingdom	2006	Health Survey for England	National	both	5+	5+	8005	8912	
3765	United Kingdom	2006	Millennium Cohort Study	National	both	5-6	5-6	6258	5999	
3766	United Kingdom	2006-2007	Avon Longitudinal Study of Parents and Children	Community	both	15	15	2289	2537	
3767	United Kingdom	2006-2007	National Child Measurement Programme	National	both	5-11	5-11	380586	358276	38
3768	United Kingdom	2006-2007	SportsLinx	Community	urban	9-10	9-10	1759	1683	
3769	United Kingdom	2006-2010	MRC National Survey of Health and Development	National	both	60-65	60-65	1061	1156	
3770	United Kingdom	2007	Health Survey for England	National	both	5+	5+	5354	5708	
3771	United Kingdom	2007	Welsh Health Survey (WHS)	Subnational	both	5-15	5-15	782	751	
3772	United Kingdom	2007-2008	National Child Measurement Programme	National	both	5-11	5-11	396795	374775	38
3773	United Kingdom	2007-2008	SportsLinx	Community	urban	9-10	9-10	1852	1815	
3774	United Kingdom	2007-2010	Southampton Women's Survey - children 6 year visit	Community	both	6-10	6-10	305	292	37
3775	United Kingdom	2008	Health Survey for England	National	both	5+	5+	8317	9472	
3776	United Kingdom	2008	Millennium Cohort Study	National	both	6-8	6-8	6961	6836	
3777	United Kingdom	2008	Scottish Health Survey (SHeS)	Subnational	both	5+	5+	2970	3533	
3778	United Kingdom	2008	Welsh Health Survey (WHS)	Subnational	both	5-15	5-15	737	624	
3779	United Kingdom	2008	The European Male Ageing Study	Community	both	45+		301		
3780	United Kingdom	2008-2009	National Child Measurement Programme	National	both	5-11	5-11	398496	377146	38
3781	United Kingdom	2008-2009	SportsLinx	Community	urban	9-10	9-10	1824	1854	
3782	United Kingdom	2008-2009	English Longitudinal Study of Ageing Wave 4	National	both	50+	50+	3540	4296	
3783	United Kingdom	2008-2012	National Diet and Nutrition Survey (NDNS)	National	both	5+	5+	2580	3094	
3784	United Kingdom	2009	Health Survey for England	National	both	5+	5+	3242	3384	
3785	United Kingdom	2009	Scottish Health Survey (SHeS)	Subnational	both	5+	5+	3621	4168	
3786	United Kingdom	2009	Welsh Health Survey (WHS)	Subnational	both	5-15	5-15	907	857	
3787	United Kingdom	2009-2010	National Child Measurement Programme	National	both	5-11	5-11	398272	377960	38
3788	United Kingdom	2009-2010	SportsLinx	Community	urban	9-10	9-10	1493	1429	
3789	United Kingdom	2009-2010	Avon Longitudinal Study of Parents and Children	Community	both	18	18	1950	2486	
3790	United Kingdom	2009-2015	Southampton Women's Survey - children 8 year visit	Community	both	8-10	8-10	598	613	37

	Country	Study years	Survey/Study name/Citation	Level of representative-ness	Rural, urban, or both	Age range as in NCD-RisC database		Sample size		Note
						Male	Female	Male	Female	
3791	United Kingdom	2010	Health Survey for England	National	both	5+	5+	4959	5547	
3792	United Kingdom	2010	Scottish Health Survey (SHeS)	Subnational	both	5+	5+	3206	3767	
3793	United Kingdom	2010	Understanding Society: the UK Household Longitudinal Study	National	both	16+	16+	6507	8422	39
3794	United Kingdom	2010	Welsh Health Survey (WHS)	Subnational	both	5-15	5-15	892	859	
3795	United Kingdom	2010-2011	National Child Measurement Programme	National	both	5-11	5-11	393146	373472	38
3796	United Kingdom	2010-2011	SportsLinx	Community	urban	9-10	9-10	1332	1252	
3797	United Kingdom	2010-2012	The British Regional Heart Study	National	urban	72-91		1676		
3798	United Kingdom	2011	British Household Panel Survey	National	both	16+	16+	1917	2391	39
3799	United Kingdom	2011	Health Survey for England	National	both	5+	5+	3680	4404	
3800	United Kingdom	2011	Scottish Health Survey (SHeS)	Subnational	both	5+	5+	3253	3862	
3801	United Kingdom	2011	Welsh Health Survey (WHS)	Subnational	both	5-15	5-15	949	836	
3802	United Kingdom	2011-2012	National Child Measurement Programme	National	both	5-11	5-11	390383	373067	38
3803	United Kingdom	2011-2012	SportsLinx	Community	urban	9-10	9-10	1286	1330	
3804	United Kingdom	2011-2013	International Study of Childhood Obesity, Lifestyle and the Environment (ISCOLE)	Community	urban	9-11	9-11	237	287	
3805	United Kingdom	2012	Health Survey for England	National	both	5+	5+	3648	4278	
3806	United Kingdom	2012	Millennium Cohort Study	National	both	10-12	10-12	6574	6451	
3807	United Kingdom	2012	Scottish Health Survey (SHeS)	Subnational	both	5+	5+	2378	2734	
3808	United Kingdom	2012	Welsh Health Survey (WHS)	Subnational	both	5-15	5-15	804	712	
3809	United Kingdom	2012-2013	National Child Measurement Programme	National	both	5-11	5-11	396700	377109	38
3810	United Kingdom	2012-2013	English Longitudinal Study of Ageing Wave 6	National	both	50+	50+	3257	4015	
3811	United Kingdom	2013	Health Survey for England	National	both	5+	5+	3910	4577	
3812	United Kingdom	2013	Scottish Health Survey (SHeS)	Subnational	both	5+	5+	2340	2747	
3813	United Kingdom	2013-2014	National Child Measurement Programme	National	both	5-11	5-11	407631	389421	38
3814	United Kingdom	2013-2014	National Diet and Nutrition Survey (NDNS)	National	both	5+	5+	940	1194	
3815	United Kingdom	2013-2014	Swan-Linx Project	Community	both	9-11	9-11	329	333	
3816	United Kingdom	2014	Health Survey for England	National	both	5+	5+	3712	4332	
3817	United Kingdom	2014	Scottish Health Survey (SHeS)	Subnational	both	5+	5+	2248	2675	
3818	United Kingdom	2014-2015	National Child Measurement Programme	National	both	5-11	5-11	412685	395006	38
3819	United Kingdom	2015	Health Survey for England	National	both	5+	5+	4837	5491	
3820	United Kingdom	2015	Millennium Cohort Study	National	both	13-15	13-15	5635	5474	
3821	United Kingdom	2015	Scottish Health Survey (SHeS)	Subnational	both	5+	5+	2264	2530	
3822	United Kingdom	2015	Swan-Linx Project	Community	both	9-11	9-11	420	395	
3823	United Kingdom	2015	MRC National Survey of Health and Development	National	both	69-70	69-70	1040	1082	
3824	United Kingdom	2015-2016	National Child Measurement Programme	National	both	5-11	5-11	427039	410085	38
3825	United Kingdom	2015-2016	National Diet and Nutrition Survey (NDNS)	National	both	5+	5+	1021	1188	
3826	United Kingdom	2015-2018	British Cohort Study 1970	National	both	45-48	45-48	3587	3826	
3827	United Kingdom	2016	Health Survey for England	National	both	5+	5+	3432	4098	
3828	United Kingdom	2016	Scottish Health Survey (SHeS)	Subnational	both	5+	5+	2041	2395	
3829	United Kingdom	2016	Swan-Linx Project	Community	both	9-11	9-11	604	653	
3830	United Kingdom	2016-2017	National Child Measurement Programme	National	both	5-11	5-11	430101	412604	38
3831	United Kingdom	2016-2019	National Diet and Nutrition Survey (NDNS)	National	both	5+	5+	1322	1588	
3832	United Kingdom	2017	Health Survey for England	National	both	5+	5+	3393	4151	
3833	United Kingdom	2017	Scottish Health Survey	Subnational	both	5+	5+	1734	2056	
3834	United Kingdom	2017-2018	National Child Measurement Programme	National	both	5-11	5-11	444135	426632	38
3835	United Kingdom	2018	Health Survey for England	National	both	5+	5+	3581	4268	
3836	United Kingdom	2018	Scottish Health Survey	Subnational	both	5+	5+	2192	2546	
3837	United Kingdom	2018-2019	National Child Measurement Programme	National	both	5-11	5-11	449768	433526	38
3838	United Kingdom	2019	Health Survey for England	National	both	5+	5+	3622	4212	
3839	United Kingdom	2019	Scottish Health Survey	Subnational	both	5+	5+	2309	2661	
3840	United Kingdom	2019	Understanding Society: Innovation Panel	National	both	16+	16+	569	668	
3841	United Kingdom	2021	Health Survey for England	National	both	5+	5+	690	831	
3842	United States of America	1976-1980	US NHANES II	National	both	5-74	5-74	6952	7508	1
3843	United States of America	1979-1980	MONICA, Stanford	Subnational	urban	25-64	25-64	703	806	40
3844	United States of America	1980-1982	The Minnesota Heart Survey	Community	both	25-75	25-75	1611	1837	
3845	United States of America	1981-1982	The Bogalusa Heart Study	Community	rural	5-17	5-17	1630	1641	
3846	United States of America	1983-1985	The Bogalusa Heart Study	Community	rural	5-17	5-17	1227	1301	
3847	United States of America	1985-1986	Coronary Artery Risk Development in Young Adults (CARDIA)	Subnational	urban	18-30	18-30	2321	2775	
3848	United States of America	1985-1986	INTERSALT, Chicago	Community	urban	20-59	20-59	97	99	
3849	United States of America	1985-1986	MONICA, Stanford	Subnational	urban	25-64	25-64	713	848	40
3850	United States of America	1985-1987	The Minnesota Heart Survey	Community	both	25-75	25-75	5220	2421	
3851	United States of America	1986	INTERSALT, Goodman	Community	urban	20-59	20-59	192	192	
3852	United States of America	1987-1988	The Bogalusa Heart Study	Community	rural	5-17	5-17	1646	1588	
3853	United States of America	1987-1988	Coronary Artery Risk Development in Young Adults (CARDIA)	Subnational	urban	20-32	20-32	2082	2506	

	Country	Study years	Survey/Study name/Citation	Level of representative-ness	Rural, urban, or both	Age range as in NCD-RisC database		Sample size		Note
						Male	Female	Male	Female	
3854	United States of America	1987-1989	Atherosclerosis Risk in Communities Study	Subnational	both	44-66	44-66	5041	6213	41
3855	United States of America	1988-1994	US NHANES III	National	both	5+	5+	11389	12256	
3856	United States of America	1989-1990	Cardiovascular Health Study	Subnational	both	65+	65+	2458	3318	
3857	United States of America	1989-1990	MONICA, Stanford	Subnational	urban	25-64	25-64	720	842	40
3858	United States of America	1990-1991	Coronary Artery Risk Development in Young Adults (CARDIA)	Subnational	urban	23-35	23-35	1945	2382	
3859	United States of America	1990-1991	Cardiovascular Health Study	Subnational	both	65+	65+	2070	2707	
3860	United States of America	1990-1992	Atherosclerosis Risk in Communities Study	Subnational	both	46-70	46-70	4537	5624	41
3861	United States of America	1991-1992	Cardiovascular Health Study	Subnational	both	65+	65+	1919	2563	
3862	United States of America	1992-1993	Coronary Artery Risk Development in Young Adults (CARDIA)	Subnational	urban	25-37	25-37	1823	2163	
3863	United States of America	1992-1993	Cardiovascular Health Study	Subnational	both	65+	65+	1985	2764	
3864	United States of America	1992-1994	The Bogalusa Heart Study	Community	rural	5-17	5-17	1525	1593	
3865	United States of America	1993-1994	Cardiovascular Health Study	Subnational	both	65+	65+	1751	2471	
3866	United States of America	1993-1995	Atherosclerosis Risk in Communities Study	Subnational	both	48-73	48-73	4000	5015	41
3867	United States of America	1993-1998	Women's Health Initiative - Observational Study	National	both		50-79		92691	
3868	United States of America	1994-1995	Cardiovascular Health Study	Subnational	both	66+	66+	1617	2354	
3869	United States of America	1995-1996	Coronary Artery Risk Development in Young Adults (CARDIA)	Subnational	urban	28-40	28-40	1739	2145	
3870	United States of America	1995-1996	Cardiovascular Health Study	Subnational	both	66+	66+	1478	2194	
3871	United States of America	1996	National Longitudinal Study of Adolescent Health Wave II	National	both	11-21	11-21	2287	2459	42
3872	United States of America	1996-1997	Cardiovascular Health Study	Subnational	both	67+	67+	1356	2043	
3873	United States of America	1996-1997	INTERMAP, Baltimore	Community	urban	40-59	40-59	146	134	
3874	United States of America	1996-1997	INTERMAP, Jackson	Community	urban	40-59	40-59	132	134	
3875	United States of America	1996-1997	INTERMAP, Pittsburgh	Community	urban	40-59	40-59	132	128	
3876	United States of America	1996-1997	Study of Women's Health Across the Nation	Subnational	both		40-55		3200	43
3877	United States of America	1996-1998	Atherosclerosis Risk in Communities Study	Subnational	both	50-75	50-75	3550	4485	41
3878	United States of America	1996-1998	INTERMAP, Minneapolis	Community	urban	40-59	40-59	130	130	
3879	United States of America	1997-1998	INTERMAP, Chicago	Community	urban	40-59	40-59	156	159	
3880	United States of America	1997-1998	INTERMAP, Corpus Christi	Community	urban	40-59	40-59	271	276	
3881	United States of America	1997-1998	Cardiovascular Health Study	Subnational	both	68+	68+	1172	1801	
3882	United States of America	1997-1999	Study of Women's Health Across the Nation	Subnational	both		40-55		2761	43
3883	United States of America	1998-1999	Coronary Artery Risk Detection in Appalachian Communities (CARDIAC), 5th Grade	Subnational	both	10-12	10-12	541	461	
3884	United States of America	1998-1999	Cardiovascular Health Study	Subnational	both	69+	69+	1092	1684	
3885	United States of America	1998-2000	Study of Women's Health Across the Nation	Subnational	both		40-55		2596	43
3886	United States of America	1999	Early Childhood Longitudinal Study	National	both	5-7	5-7	2540	2495	
3887	United States of America	1999-2000	US NHANES 1999-2000	National	both	5+	5+	3809	3791	
3888	United States of America	1999-2001	Study of Women's Health Across the Nation	Subnational	both		40-56		2507	43
3889	United States of America	2000	Early Childhood Longitudinal Study	National	both	6-8	6-8	7950	7598	
3890	United States of America	2000-2001	Coronary Artery Risk Detection in Appalachian Communities (CARDIAC), 5th Grade	Subnational	both	10-12	10-12	639	569	
3891	United States of America	2000-2001	Coronary Artery Risk Development in Young Adults (CARDIA)	Subnational	urban	33-45	33-45	1570	1949	
3892	United States of America	2000-2002	Study of Women's Health Across the Nation	Subnational	both		40-57		2441	43
3893	United States of America	2001	Coronary Artery Risk Detection in Appalachian Communities (CARDIAC), 5th Grade	Subnational	both	10-12	10-12	1732	1631	
3894	United States of America	2001-2002	US NHANES 2001-2002	National	both	5+	5+	4045	4006	
3895	United States of America	2001-2002	National Longitudinal Study of Adolescent Health Wave III	National	both	18-28	18-28	2139	2443	42
3896	United States of America	2002	Early Childhood Longitudinal Study	National	both	8-9	8-9	7043	6821	
3897	United States of America	2002-2003	Coronary Artery Risk Detection in Appalachian Communities (CARDIAC), 5th Grade	Subnational	both	10-12	10-12	2635	2594	
3898	United States of America	2003-2004	Coronary Artery Risk Detection in Appalachian Communities (CARDIAC), 5th Grade	Subnational	both	10-12	10-12	4581	4061	
3899	United States of America	2003-2004	US NHANES 2003-2004	National	both	5+	5+	3938	3838	
3900	United States of America	2004	Early Childhood Longitudinal Study	National	both	10-11	10-11	5342	5335	
3901	United States of America	2004	Health and Retirement Study	National	both	24+	24+	240	260	
3902	United States of America	2004	2004 New York City HANES	Community	urban	20+	20+	810	1105	
3903	United States of America	2004-2005	Coronary Artery Risk Detection in Appalachian Communities (CARDIAC), 5th Grade	Subnational	both	10-12	10-12	4340	4277	
3904	United States of America	2005-2006	Coronary Artery Risk Detection in Appalachian Communities (CARDIAC), 2nd Grade	Subnational	both	7-9	7-9	327	270	
3905	United States of America	2005-2006	Coronary Artery Risk Detection in Appalachian Communities (CARDIAC), 5th Grade	Subnational	both	10-12	10-12	4859	4205	
3906	United States of America	2005-2006	US NHANES 2005-2006	National	both	5+	5+	3984	3835	
3907	United States of America	2005-2006	Coronary Artery Risk Development in Young Adults (CARDIA)	Subnational	urban	38-50	38-50	1528	2000	
3908	United States of America	2005-2006	Cardiovascular Health Study	Subnational	both	70+	70+	375	684	
3909	United States of America	2005-2006	National Social Life Health and Aging Project	National	both	57-85	57-85	1355	1435	44
3910	United States of America	2006	Health and Retirement Study	National	both	53+	53+	2809	3788	
3911	United States of America	2006-2007	Coronary Artery Risk Detection in Appalachian Communities (CARDIAC), 2nd Grade	Subnational	both	7-9	7-9	843	798	
3912	United States of America	2006-2007	Coronary Artery Risk Detection in Appalachian Communities (CARDIAC), 5th Grade	Subnational	both	10-12	10-12	4039	3418	
3913	United States of America	2007	Early Childhood Longitudinal Study	National	both	13-14	13-14	4256	4251	
3914	United States of America	2007-2008	Coronary Artery Risk Detection in Appalachian Communities (CARDIAC), 2nd Grade	Subnational	both	7-9	7-9	3873	3975	
3915	United States of America	2007-2008	Coronary Artery Risk Detection in Appalachian Communities (CARDIAC), 5th Grade	Subnational	both	10-12	10-12	4046	3634	
3916	United States of America	2007-2008	US NHANES 2007-2008	National	both	5+	5+	4086	4038	

	Country	Study years	Survey/Study name/Citation	Level of representative-ness	Rural, urban, or both	Age range as in NCD-RisC database		Sample size		Note
						Male	Female	Male	Female	
3917	United States of America	2008	Health and Retirement Study	National	both	55+	55+	2469	3448	
3918	United States of America	2008-2009	Coronary Artery Risk Detection in Appalachian Communities (CARDIAC), 2nd Grade	Subnational	both	7-9	7-9	4907	5178	
3919	United States of America	2008-2009	Coronary Artery Risk Detection in Appalachian Communities (CARDIAC), 5th Grade	Subnational	both	10-12	10-12	4152	3593	
3920	United States of America	2008-2009	National Longitudinal Study of Adolescent Health Wave IV	National	both	24-34	24-34	2317	2725	42
3921	United States of America	2009-2010	Coronary Artery Risk Detection in Appalachian Communities (CARDIAC), 2nd Grade	Subnational	both	7-9	7-9	5217	5562	
3922	United States of America	2009-2010	Coronary Artery Risk Detection in Appalachian Communities (CARDIAC), 5th Grade	Subnational	both	10-12	10-12	3990	3513	
3923	United States of America	2009-2010	US NHANES 2009-2010	National	both	5+	5+	4291	4332	
3924	United States of America	2010	Early Childhood Longitudinal Study	National	both	5-6	5-6	7811	7391	
3925	United States of America	2010-2011	Coronary Artery Risk Detection in Appalachian Communities (CARDIAC), 2nd Grade	Subnational	both	7-9	7-9	4994	5286	
3926	United States of America	2010-2011	Coronary Artery Risk Detection in Appalachian Communities (CARDIAC), 5th Grade	Subnational	both	10-12	10-12	3313	2819	
3927	United States of America	2010-2011	Coronary Artery Risk Development in Young Adults (CARDIA)	Subnational	urban	43-55	43-55	1513	1976	
3928	United States of America	2010-2011	Health and Retirement Study	National	both	57+	57+	2533	3465	
3929	United States of America	2010-2011	National Social Life Health and Aging Project	National	both	61-91	61-91	1440	1640	44
3930	United States of America	2011	Early Childhood Longitudinal Study - Fall	National	both	5-7	5-7	2722	2493	
3931	United States of America	2011	Early Childhood Longitudinal Study - Spring	National	both	5-7	5-7	8729	8373	
3932	United States of America	2011-2012	Coronary Artery Risk Detection in Appalachian Communities (CARDIAC), 2nd Grade	Subnational	both	7-9	7-9	4122	4412	
3933	United States of America	2011-2012	Coronary Artery Risk Detection in Appalachian Communities (CARDIAC), 5th Grade	Subnational	both	10-12	10-12	2644	2112	
3934	United States of America	2011-2012	US NHANES 2011-2012	National	both	5+	5+	3951	3887	
3935	United States of America	2011-2013	International Study of Childhood Obesity, Lifestyle and the Environment (ISCOLE)	Community	urban	9-11	9-11	281	368	
3936	United States of America	2011-2013	Atherosclerosis Risk in Communities Study	Subnational	both	67-90	67-90	1787	2431	41
3937	United States of America	2012	Early Childhood Longitudinal Study - Fall	National	both	6-8	6-8	2446	2262	
3938	United States of America	2012	Early Childhood Longitudinal Study - Spring	National	both	6-8	6-8	7667	7358	
3939	United States of America	2012	Health and Retirement Study	National	both	59+	59+	2294	3095	
3940	United States of America	2012-2013	Coronary Artery Risk Detection in Appalachian Communities (CARDIAC), 2nd Grade	Subnational	both	7-9	7-9	4565	5184	
3941	United States of America	2012-2013	Coronary Artery Risk Detection in Appalachian Communities (CARDIAC), 5th Grade	Subnational	both	10-12	10-12	2796	2332	
3942	United States of America	2013	Early Childhood Longitudinal Study - Spring	National	both	7-9	7-9	6975	6705	
3943	United States of America	2013-2014	Coronary Artery Risk Detection in Appalachian Communities (CARDIAC), 2nd Grade	Subnational	both	7-9	7-9	4994	5415	
3944	United States of America	2013-2014	Coronary Artery Risk Detection in Appalachian Communities (CARDIAC), 5th Grade	Subnational	both	10-12	10-12	2408	1987	
3945	United States of America	2013-2014	US NHANES 2013-2014	National	both	5+	5+	4105	4225	
3946	United States of America	2014	Early Childhood Longitudinal Study - Spring	National	both	8-10	8-10	6458	6158	
3947	United States of America	2014	Health and Retirement Study	National	both	61+	61+	2152	2972	
3948	United States of America	2014-2015	Coronary Artery Risk Detection in Appalachian Communities (CARDIAC), 2nd Grade	Subnational	both	7-9	7-9	5182	5495	
3949	United States of America	2014-2015	Coronary Artery Risk Detection in Appalachian Communities (CARDIAC), 5th Grade	Subnational	both	10-12	10-12	2286	1889	
3950	United States of America	2015	Early Childhood Longitudinal Study - Spring	National	both	9-11	9-11	5954	5679	
3951	United States of America	2015-2016	US NHANES 2015-2016	National	both	5+	5+	3959	4091	
3952	United States of America	2015-2016	National Social Life Health and Aging Project	Community	both	57-96	57-96	1577	1895	45
3953	United States of America	2016	Early Childhood Longitudinal Study - Spring	National	both	10-12	10-12	5625	5337	
3954	United States of America	2016-2017	Atherosclerosis Risk in Communities Study	Subnational	both	73-94	73-94	1465	2042	41
3955	United States of America	2016-2018	Health and Retirement Study	National	both	63+	63+	1638	2321	
3956	United States of America	2017-2018	US NHANES 2017-2018	National	both	5+	5+	3642	3784	
3957	United States of America	2018-2019	Atherosclerosis Risk in Communities Study	Subnational	both	76-96	76-96	1242	1672	41
3958	United States of America	2018-2019	Health and Retirement Study	National	both	65+	65+	1424	2034	
3959	United States of America	2019-2020	US NHANES 2019-2020	Subnational	both	5+	5+	2396	2354	46
3960	United States of America	2021-2023	US NHANES 2021-2023	National	both	5+	5+	3698	4282	
3961	Uruguay	1999-2000	The Survey on Health, Well-Being, and Aging in Latin America and the Caribbean (SABE)	Community	urban	60+	60+	492	828	4
3962	Uruguay	2004	CUiiDARTE Project	National	urban	5-8	5-8	127	149	
3963	Uruguay	2005	CUiiDARTE Project	National	urban	6-10	6-10	105	105	
3964	Uruguay	2006	STEPS	National	urban	25-64	25-64	261	641	
3965	Uruguay	2009-2010	CUiiDARTE Project	National	urban	10-14	10-14	117	129	
3966	Uruguay	2011-2012	CESCAS Study	Community	urban	35-74	35-74	640	900	
3967	Uruguay	2012	Global School-based Student Health Survey	National	both		13-15		1377	
3968	Uruguay	2012-2016	Genotype, Phenotype and Environment of Hypertension in Uruguay (GEFA-HT-UY)	Community	urban	19+	19+	124	189	
3969	Uruguay	2013	STEPS	National	urban	15-64	15-64	821	1400	
3970	Uruguay	2015	CUiiDARTE Project	National	urban	17-20	17-20	135	142	
3971	Uruguay	2015-2016	Survey of Nutrition, Child Development and Health	National	urban	5-6	5-6	355	326	
3972	Uruguay	2016-2017	CUiiDARTE Project	Community	urban	5-6	5-6	395	375	
3973	Uruguay	2018	Evaluation of the School Feeding Program and monitoring of the nutritional status of children in public and private schools in Uruguay	National	both	5-11	5-11	1447	1538	
3974	Uruguay	2019	Global School-based Student Health Survey	National	both	13-16	13-16	901	1022	
3975	Uruguay	2019	Survey of Nutrition, Child Development and Health	National	urban	5-10	5-10	1253	1165	
3976	Uzbekistan	1996	DHS	National	both		15-49		4082	
3977	Uzbekistan	2002	DHS	National	both	15-59	15-49	2331	5275	
3978	Uzbekistan	2014	STEPS	National	both	18-64	18-64	1533	2164	

	Country	Study years	Survey/Study name/Citation	Level of representative-ness	Rural, urban, or both	Age range as in NCD-RisC database		Sample size		Note
						Male	Female	Male	Female	
3979	Uzbekistan	2015-2016	Epidemiology of Diabetes and Prediabetes in Uzbekistan Screening Results	Subnational	both	35+	35+	714	1511	
3980	Uzbekistan	2017	Nutrition Survey	National	both		15-49		2198	
3981	Uzbekistan	2019	STEPS	National	both	18-69	18-69	1462	2226	
3982	Uzbekistan	2022	Childhood Obesity Surveillance Initiative 6	National	both	7-8	7-8	1952	1741	
3983	Vanuatu	1996	Second National Nutrition Survey	National	both		15-50		1353	
3984	Vanuatu	1998	Vanuatu Non-comunicable Disease Survey	National	both	20-60	20-60	533	730	
3985	Vanuatu	2005	STEPS	Subnational	both	15-60	15-60	626	759	
3986	Vanuatu	2011	STEPS	National	both	25-64	25-64	2251	2183	
3987	Venezuela	1998-2001	Maracaibo aging study Santa lucia cohort	Community	urban	55+	55+	760	1526	
3988	Venezuela	1999-2001	Florez et al., Diabetes Res Clin Pract 69(1):63-77, 2005	Subnational	both	15+	15+	1134	2599	
3989	Venezuela	2000	Diaz et al., Invest Clin 46(2):111-19, 2005	Community	urban	60+	60+	42	59	
3990	Venezuela	2004-2005	CArdiovascular Risk factors Multiple Evaluation in Latin America (CARMELA)	Community	urban	25-64	25-64	713	1123	
3991	Venezuela	2005-2006	Brajkovich et al., Rev Ven Endoc Metab 4(3):31-32, 2006	Community	urban	20-65	20-65	205	439	
3992	Venezuela	2007-2008	Venezuelan Study of Metabolic Syndrome, Obesity and Lifestyle (VEMSOLS)	Community	urban	20+	20+	107	230	
3993	Venezuela	2008-2009	Venezuelan Study of Metabolic Syndrome, Obesity and Lifestyle (VEMSOLS)	Community	rural	20+	20+	51	89	
3994	Venezuela	2010-2011	Cardiometabolic risk factors in schoolchildren and adolescents of Mérida, Venezuela (CREDEFAR)	Community	urban	9-18	9-18	443	475	
3995	Venezuela	2010-2011	Venezuelan Study of Metabolic Syndrome, Obesity and Lifestyle (VEMSOLS)	Community	urban	20+	20+	66	193	
3996	Venezuela	2014-2015	Latin American Study of Nutrition and Health (ELANS)	National	urban	15-65	15-65	552	580	
3997	Venezuela	2014-2017	Maracaibo aging study Santa Rosa cohort	Community	urban	37+	37+	115	292	
3998	Venezuela	2015-2017	Cardio-Metabolic Health Venezuelan Study (EVESCAM)	National	both	20+	20+	1056	2346	
3999	Venezuela	2018-2020	Cardio-metabolic Health Venezuelan Study (EVESCAM) follow-up	National	both	22+	22+	355	892	
4000	Viet Nam	1981-1985	National Nutrition Survey	Subnational	rural	18+	18+	4815	7985	
4001	Viet Nam	1987-1989	General Nutrition Survey	National	both	15-70	15-70	13776	17271	
4002	Viet Nam	1992-1993	Living Standard Survey	National	both	5+	5+	9418	10209	
4003	Viet Nam	1997-1998	Living Standard Survey	National	both	5+	5+	12052	13118	
4004	Viet Nam	2000	National Nutrition Survey	National	both	20+	20+	8985	9464	
4005	Viet Nam	2001-2002	Viet Nam National Health Survey 2001-2002	National	both	5+	5+	66723	71616	
4006	Viet Nam	2001-2003	The National Epidemiological Survey on Hypertension and Its Risk Factors (North)	Subnational	both	25-74	25-74	2386	3604	
4007	Viet Nam	2003-2004	The Survey on Heart Failure and Its Risk Factors	Subnational	both	25-74	25-74	1853	2636	
4008	Viet Nam	2004	The Hypertension Management Programme in Rural Communes (Hanoi)	Community	rural	25-74	25-74	855	1288	
4009	Viet Nam	2004	Cuong et al., Eur J Clin Nutr 61(5):673-81, 2007	Community	urban	20-60	20-60	717	771	
4010	Viet Nam	2005	The Survey on Non-Communicable Disease Risk Factors	Subnational	both	25-74	25-74	1136	1220	
4011	Viet Nam	2005	Non-communicable disease risk factors in Ho Chi Minh City	Community	urban	25-64	25-64	908	1052	
4012	Viet Nam	2005	STEPS Bavi district	Subnational	rural	25-64	25-64	987	997	
4013	Viet Nam	2005	National Adult Overweight Survey	National	both	25-64	25-64	8474	8725	
4014	Viet Nam	2006	Qualitative and quantitative assessment of nutritional status and lifestyles of Vietnamese adolescents	Subnational	rural	15-17	15-17	252	363	
4015	Viet Nam	2006	Qualitative and quantitative assessment of nutritional status and lifestyles of Vietnamese adolescents	Subnational	urban	15-17	15-17	254	334	
4016	Viet Nam	2006	The Hypertension Management Programme in Rural Communes (Bavi)	Community	rural	25-74	25-74	395	643	
4017	Viet Nam	2006-2008	The National Epidemiological Survey on Hypertension and Its Risk Factors (South)	Subnational	both	25-74	25-74	1310	2078	
4018	Viet Nam	2007	The Hypertension Management Programme in Rural Communes (Phu Phuong)	Community	rural	25-74	25-74	364	616	
4019	Viet Nam	2008-2009	The Survey on Diabetes and Its Risk Factors	Subnational	both	25+	25+	830	1446	
4020	Viet Nam	2009	The Hypertension Management Programme in Rural Communes (Phu Cuong)	Community	rural	25-74	25-74	362	677	
4021	Viet Nam	2009	STEPS	National	both	25-64	25-64	6703	7776	
4022	Viet Nam	2009-2010	Vietnam National Nutrition Survey 2009-2010	National	both	5+	5+	16036	16619	
4023	Viet Nam	2011	SEANUTS	National	both	5-11	5-11	975	980	
4024	Viet Nam	2012	National Survey of Diabetes in Vietnam	National	both	30-69	30-69	5319	5855	
4025	Viet Nam	2013	Global School-based Student Health Survey	National	both	13-17	13-17	1368	1578	
4026	Viet Nam	2015	STEPS	National	both	18-69	18-69	1316	1722	
4027	Viet Nam	2019	Global School-based Student Health Survey	National	both	13-18	13-18	3572	4117	
4028	Viet Nam	2021	STEPS	National	both	18+	18+	1837	1886	
4029	Yemen	1997	DHS	National	both		15-49		5123	
4030	Yemen	2005-2006	Yemen Household Budget Survey 2005-2006	National	both	5+	5+	3290	3307	
4031	Yemen	2007-2009	Hypertension and Diabetes in Yemen (HYDY)	National	rural	6-70	6-70	3023	3065	
4032	Yemen	2007-2009	Hypertension and Diabetes in Yemen (HYDY)	National	urban	6-70	6-70	2996	3077	
4033	Yemen	2013	DHS	National	both		15-49		22527	
4034	Zambia	1992	DHS	National	both		20-49		2829	
4035	Zambia	1996	DHS	National	both		20-49		3485	
4036	Zambia	2001-2002	DHS	National	both		15-49		6732	
4037	Zambia	2007	DHS	National	both		15-49		6378	
4038	Zambia	2008	STEPS	Subnational	urban	25+	25+	626	1214	
4039	Zambia	2013-2014	DHS	National	both		15-49		14837	
4040	Zambia	2017	STEPS	National	both	18-69	18-69	1565	2439	
4041	Zimbabwe	1985-1986	INTERSALT	Community	urban	20-59	20-59	100	95	

	Country	Study years	Survey/Study name/Citation	Level of representative-ness	Rural, urban, or both	Age range as in NCD-RisC database		Sample size		Note
						Male	Female	Male	Female	
4042	Zimbabwe	1991	Zinyowera et al., Cent Afr J Med 40(2):33-8, 1994	Community	both	18+	18+	775	734	
4043	Zimbabwe	1994	DHS	National	both		20-49		1776	
4044	Zimbabwe	1995	Mufunda et al., J Hum Hypertens 14(1):65-73, 2000	Community	urban	25+	25+	384	391	
4045	Zimbabwe	1999	DHS	National	both		15-49		5169	
4046	Zimbabwe	2005	STEPS	National	both	25+	25+	569	1808	
4047	Zimbabwe	2005-2006	DHS	National	both		15-49		8186	
4048	Zimbabwe	2010-2011	DHS	National	both	15-54	15-49	7383	8329	
4049	Zimbabwe	2015	DHS	National	both	15-54	15-49	8386	9396	
4050	Zimbabwe	2021-2022	Zimbabwe CHIEDZA	Subnational	both	18-24	18-24	6908	9968	

1. National studies for the 3 years prior to 1980 were assigned to 1980 so that they can inform the estimates in countries with slightly earlier national data.
2. NSW Ministry of Health and the Physical Activity, Nutrition and Obesity Research Group (PANORG).
3. This research uses data from Australia Health Survey (AHS). We thank the Health Section, Australian Bureau of Statistics, Belconnen, ACT, Australia for support for AHS 2011-2012.
4. The bibliographic citation for this data source is: Pelaez, Martha, Alberto Palloni, Cecilia Albala, Juan C. Alfonso, Roberto Ham-Chande, Anselm Hennis, Maria Lucia Lebrao, Esther Lesn-Diaz, Edith Pantelides, and Omar Prats. SABE - SURVEY ON HEALTH, WELL-BEING, AND AGING IN LATIN AMERICA AND THE CARIBBEAN, 2000 [Computer file]. ICPSR version. Washington, D.C.: Pan American Health Organization/World Health Organization (PAHO/WHO) [producers], 2004. Ann Arbor, MI: Inter-university Consortium for Political and Social Research [distributor], 2005.
5. Sciensano, OD Public health and surveillance (2020). Health Interview Survey 2018 [Data file and code book]. Conditionally obtainable from the Sciensano website: https://www.sciensano.be/en/node/55737/health-interview-survey-microdata-request-procedure.
6. The EPIDEMCA study was funded by the French National Research Agency (ANR) through the ANR-09-MNPS-009-01 grant; the AXA Research Fund (grant 2012–Project Public Health Institute [Inserm]–PREUX Pierre-Marie), and the Limoges University Hospital through its Appel à Projet des Equipes Émergentes et Labellisées scheme (APREL).
7. This research used information from the Health Surveys for epidemiological surveillance of the Undersecretary of Public Health. The author would like to thank the Chilean Ministry of Health for allowing him to have the database. All the results obtained from the study or research are the responsibility of the author and do not commit the institution in any way.
8. This research uses data from China Health and Nutrition Survey (CHNS). We thank the National Institute for Health (NIH), the Eunice Kennedy Shriver National Institute of Child Health and Human Development (NICHD) for R01 HD30880, National Institute on Aging (NIA) for R01 AG065357, National Institute of Diabetes and Digestive and Kidney Diseases (NIDDK) for R01 DK104371 and P30 DK056350, National Heart, Lung, and Blood Institute (NHLBI) for R01 HL108427, the NIH Fogarty grant D43 TW009077, the Carolina Population Center for P2C HD050924 and P30 AG066615. We also thank the National Institute for Nutrition and Health, China Center for Disease Control and Prevention; Beijing Municipal Center for Disease Control and Prevention; the Chinese National Human Genome Center at Shanghai; and the China-Japan Friendship Hospital, National Health Commission of China.
9. The bibliographic citation for this data source is: Yi, Zeng, James W. Vaupel, Xiao Zhenyu, Liu Yuzhi, and Zhang Chunyuan. Chinese Longitudinal Healthy Longevity Survey (CLHLS), 1998-2005. ICPSR24901-v2. Ann Arbor, MI: Inter-university Consortium for Political and Social Research [distributor], 2009-06-04. http://doi.org/10.3886/ICPSR24901.v2.
10. This research uses data from China Health and Nutrition Survey (CHNS). We thank the National Institute of Nutrition and Food Safety, China Center for Disease Control and Prevention, Carolina Population Center (5 R24 HD050924), the University of North Carolina at Chapel Hill, the NIH (R01 HD30880, DK056350, R24-HD050924, and R01 HD38700) and the Fogarty International Center, NIH for financial support for the CHNS data collection and analysis files from 1989 to 2011 and future surveys, and the China Japan Friendship Hospital, Ministry of Health for support for CHNS 2009.
11. The bibliographic citation for this data source is: Center for Healthy Aging and Development Studies, 2020, "The Chinese Longitudinal Healthy Longevity Survey (CLHLS)-Longitudinal Data（1998-2018）", https://doi.org/10.18170/DVN/WBO7LK, Peking University Open Research Data Platform, V2.
12. The bibliographic citation for this data source is: Central Statistics Agency of Ethiopia (CSA) & Living Standards Measurement Study Integrated Surveys of Agriculture (LSMS-ISA), 2015, "Socioeconomic Survey 2013-2014" [Data set]. World Bank, Development Data Group. https://doi.org/10.48529/MCCP-Y123.
13. Santé publique France, en tant qu'investigateur principal, promoteur et financeur de l'étude ENNS.; Aux Centres d'examen de santé de la Caisse nationale d'assurance maladie des travailleurs salariés (CnamTS) et leurs laboratoires.
14. Santé publique France, en tant qu'investigateur principal, promoteur et financeur de l'étude Esteban.; Aux Centres d'examen de santé de la Caisse nationale d'assurance maladie des travailleurs salariés (CnamTS) et leurs laboratoires.
15. Data have been provided by the Study of Health in Pomerania (SHIP) from the University Medicine Greifswald.
16. The authors thank the Heinz Nixdorf Foundation [Chairman: Martin Nixdorf; Past Chairman: Dr jur. Gerhard Schmidt], for their generous support of this study. Parts of the study were also supported by the German Research Council (DFG) [DFG project: EI 969/2-3, ER 155/6-1;6-2, HO 3314/2-1;2-2;2-3;4-3, INST 58219/32-1, JO 170/8-1, KN 885/3-1, PE 2309/2-1, SI 236/8-1;9-1;10-1,], the German Ministry of Education and Science [BMBF project: 01EG0401, 01GI0856, 01GI0860, 01GS0820_WB2-C, 01ER1001D, 01GI0205], the Ministry of Innovation, Science, Research and Technology, North Rhine-Westphalia (MIWFT-NRW), the Else Kröner-Fresenius-Stiftung [project: 2015_A119] and the German Social Accident Insurance [DGUV project: FF-FP295]. Furthermore the study was supported by the Competence Network for HIV/AIDS, the deanship of the University Hospital and IFORES of the University Duisburg-Essen, the European Union, the German Competence Network Heart Failure, Kulturstiftung Essen, the Protein Research Unit within Europe (PURE), the Dr. Werner-Jackstädt Stiftung and the following companies: Celgene GmbH München, Imatron/GE-Imatron, Janssen, Merck KG, Philips, ResMed Foundation, Roche Diagnostics, Sarstedt AG&Co, Siemens HealthCare Diagnostics, Volkswagen Foundation. The authors express their gratitude to all study participants of the Heinz Nixdorf Recall (HNR) Study, the personnel of the HNR study center and the EBT-scanner facilities, the investigative group and all former employees of the HNR study. The authors also thank the Advisory Board of the HNR Study: T. Meinertz, Hamburg, Germany (Chair); C. Bode, Freiburg, Germany; P.J. de Feyter, Rotterdam, Netherlands; B. Güntert, Hall i.T., Austria; F. Gutzwiller, Bern, Switzerland; H. Heinen, Bonn, Germany; O. Hess (†), Bern, Switzerland; B. Klein (†), Essen, Germany; H. Löwel, Neuherberg, Germany; M. Reiser, Munich, Germany; G. Schmidt (†), Essen, Germany; M. Schwaiger, Munich, Germany; C. Steinmüller, Bonn, Germany; T. Theorell, Stockholm, Sweden; and S.N Willich, Berlin, Germany.
17. The bibliographic citation for this data source is: Desai, Sonalde, Vanneman, Reeve, and National Council of Applied Economic Research, New Delhi. India Human Development Survey (IHDS), 2005. Inter-university Consortium for Political and Social Research [distributor], 2018-08-08. https://doi.org/10.3886/ICPSR22626.v12.
18. The CARRS Study was funded in part by the National Heart, Lung, and Blood Institute (NHLBI), National Institutes of Health (NIH), Department of Health and Human Services, under Contract No. HHSN268200900026C, the United Health Group, Minneapolis, MN, USA, and by the by the National Heart, Lung, And Blood Institute of the National Institutes of Health under Award Number P01HL154996.
19. The bibliographic citation for this data source is: Desai, Sonalde, Reeve Vanneman and National Council of Applied Economic Research. India Human Development Survey-II (IHDS-II), 2011-12. Inter-university Consortium for Political and Social Research [distributor], 2018-08-08. https://doi.org/10.3886/ICPSR36151.v6.
20. Accessed via the Irish Social Science Data Archive - www.ucd.ie/issda.
21. The Older Persons in Jamaica Study was funded by the National Health Fund, Jamaica.
22. National studies from 2025 were assigned to 2024 so that they can inform the estimates in countries with slightly later national data.
23. The study was supported by the grant of the Ministry of Healthcare of the Republic of Kazakhstan "National Programme for the Introduction of Personalized and Preventive Medicine in The Republic of Kazakhstan (2021–2023)" (Grant number OR12165486).
24. The National Nutrition Survey of Kuwait (2008-2009) was supported by The Kuwait Foundation for Advancement Sciences (KFAS) Grant # 2003-1202-02.
25. The MHAS Cognitive Aging Ancillary Study (Mex-Cog) is sponsored by the National Institutes of Health/National Institute on Aging (NIH R01AG051158). Data files and documentation are public use and available at www.MHASweb.org.
26. The Longitudinal Aging Study Amsterdam is supported by a grant from the Netherlands Ministry of Health Welfare and Sports, Directorate of Long-Term Care.
27. The data collection [in 2012-2013 and 2013-2014] was financially supported by the Netherlands Organization for Scientific Research (NWO) in the framework of the project "New Cohorts of young old in the 21st century" (file number 480-10-014).
28. The bibliographic citation for this data source is: Am J Hypertens 2009 Jan;22(1):100-5 and Atherosclerosis. 2009 Mar;203(1):257-62.
29. The bibliographic citation for this data source is: Palloni, Alberto, Ana Luisa Davila, and Melba Sanchez-Ayendez. Puerto Rican Elderly: Health Conditions (PREHCO) Project, 2002-2003, 2006-2007. ICPSR34596-v1. Ann Arbor, MI: Inter-university Consortium for Political and Social Research[distributor], 2013-09-13. doi:10.3886/ICPSR34596.v1.

30. Dr Take Naseri (Ministry of Health, Samoa), and Muagututia Sefuiva Reupena (Lutia I Puava Ae Mapu I Fagalele) contributed to the GWAS studies in Samoa.
31. The SP2 and SCCS2 studies are supported by individual research and clinical scientist award schemes from National Medical Research Council (NMRC) and the Biomedical Research Council (BMRC) of Singapore, the Singapore Ministry of Health, National University of Singapore and University Health System, Singapore.
32. The SH2012 and SH2 studies are supported by infrastructure funding from the Singapore Ministry of Health (Population Health Metrics Population Health Metrics and Analytics PHMA), National University of Singapore and National University Health System, Singapore.
33. Data used for this research was provided by the INMA – INfancia y Medio Ambiente [Environment and Childhood] Project (www.proyectoinma.org), which is supported in part by funds. This study was funded by grants from Instituto de Salud Carlos III (Red INMA G03/176 and CB06/02/0041), Spanish Ministry of Health (FIS-97/1102, FIS-07/0252, FIS-PS09/00362, 97/0588, 00/0021-2, PI061756, PS0901958, PI14/00677 incl.FEDER funds, FIS-PS09/00090, PI041436, FIS-PI042018, FIS-PI06/0867, PI081151 incl. FEDER funds, FIS-PI09/02311 and
34. The ULSAM study was supported by Uppsala University and Uppsala University Hospital.
35. The Swiss BMI Monitoring Study was supported by the Health Service of the town of Berne, Basel Health Service for Children and Adolescents, and the School-Medical Service of the town of Zurich.
36. The British Women's Heart and Health Study is supported by the British Heart Foundation (PG/13/66/30442). British Women's Heart and Health Study data are available to bona fide researchers for research purposes. Please refer to the BWHHS data sharing policy at http://www.ucl.ac.uk/british-womens-heart-health-study.
37. Prof Keith M Godfrey is supported by the UK National Institute for Health and Care Research (NIHR Senior Investigator (NF-SI-0515-10042) and NIHR Southampton Biomedical Research Centre (NIHR203319)) and the Wessex Medical Trust, Gerald Kerkut Charitable Trust and Rosetrees Trust.
38. NHS digital was the data source for NCMP data.
39. University of Essex. Institute for Social and Economic Research and National Centre for Social Research, Understanding Society: Waves 2 and 3 Nurse Health Assessment, 2010- 2012 [data collection]. 5th Edition. UK Data Service. SN:7251. http://doi.org/10.5255/UKDA-SN-7251-5."
40. Prof Stephen Fortmann contributed data from the Stanford Five-City Project.
41. This Manuscript was prepared using ARIC Research Materials obtained from the NHLBI Biologic Specimen and Data Repository Information Coordinating Center and does not necessarily reflect the opinions or views of the ARIC or the NHLBI.
42. This research uses data from Add Health, a program project designed by J. Richard Udry, Peter S. Bearman, and Kathleen Mullan Harris, and funded by a grant P01-HD31921 from the Eunice Kennedy Shriver National Institute of Child Health and Human Development, with cooperative funding from 17 other agencies. Special acknowledgment is due Ronald R. Rindfuss and Barbara Entwisle for assistance in the original design. Persons interested in obtaining data files from Add Health should contact Add Health, Carolina Population Center, 123 W. Franklin Street, Chapel Hill, NC 27516-2524 (addhealth@unc.edu). No direct support was received from grant P01-HD31921 for this analysis.
43. The bibliographic citation for this data source is: Sutton-Tyrrell, Kim, Faith Selzer, MaryFran Sowers, Robert Neer, Lynda Powell, Ellen Gold, Gail Greendale, Gerson Weiss, Karen Matthews, and Sonja McKinlay. Study of Women's Health Across the Nation (SWAN), 1996-1997: Baseline Dataset. ICPSR28762-v2. Ann Arbor, MI: Inter-university Consortium for Political and Social Research[distributor], 2014-02-04. http://doi.org/10.3886/ICPSR28762.v2.
44. The bibliographic citation for this data source is: Waite, Linda J., Kathleen Cagney, William Dale, Elbert Huang, Edward O. Laumann, Martha McClintock, Colm A. O'Muircheartaigh, L. Phillip Schumm, and Benjamin Cornwell. National Social Life, Health, and Aging Project (NSHAP): Wave 2 and Partner Data Collection. ICPSR34921-v1. Ann Arbor, MI: Inter-university Consortium for Political and Social Research [distributor], 2014-04-29. https://doi.org/10.3886/ICPSR34921.v1.
45. The bibliographic citation for this data source is: Waite, Linda J., Cagney, Kathleen A., Dale, William, Hawkley, Louise C., Huang, Elbert S., Lauderdale, Diane S., … Schumm, L. Philip. National Social Life, Health, and Aging Project (NSHAP): Round 3 and COVID-19 Study, [United States], 2015-2016, 2020-2021. Inter-university Consortium for Political and Social Research [distributor], 2021-12-13. https://doi.org/10.3886/ICPSR36873.v5.
46. Due to the COVID-19 pandemic the NHANES 2019-2020 cycle was not completed. As a result the data are not nationally representative and considered subnational.

Supplementary Table 3. Specification of the Bayesian hierarchical model.

Subscripts: i – study, j – country, l – region, m – super-region, k – age term. Superscripts: c – country, r – region, s – super-region. Unless otherwise specified, we denote a normal distribution as $N(\mu, v)$ where μ is the mean and v the variance. All standard deviations are constrained to be positive.

Parameter name	Symbol	Prior	Constraints
Global intercepts	a^g	$a^g \sim N(0, 1000)$	
Country, region and super-region intercepts	a_j^c, a_l^r, a_m^s	$a^x \sim N(0, \kappa_a^x)$, $x \in \{c, r, s\}$	
standard deviation	$\sqrt{\kappa_a^x}$	$\sqrt{\kappa_a^x} \propto 1$, $x \in \{c, r, s\}$	
Global linear slope	b^g	$b^g \sim N(0, 1000)$	
Country, region and super-region linear slopes	b_j^c, b_l^r, b_m^s	$b^x \sim N(0, \kappa_b^x)$, $x \in \{c, r, s\}$	
standard deviation	$\sqrt{\kappa_b^x}$	$\sqrt{\kappa_b^x} \propto 1$, $x \in \{c, r, s\}$	
Country, region, super-region and global non-linear change	u_j^c, u_l^r, u_m^s, u^g	$N(0,\ precision = \lambda_x P)$, $x \in \{c, r, s, g\}$	Mean and slope of each of u^g, u^s, u^r, and $u^c = 0$; $\lambda_c < \lambda_r < \lambda_s < \lambda_g < e^{20}$
standard deviation	$1/\sqrt{\lambda_x}$	$1/\sqrt{\lambda_x} \propto 1$, $x \in \{c, r, s, g\}$	
Global intercept in k^{th} age term coefficient	ψ_k^g	$\psi_k^g \sim N(0, 1000)$	
Country, region and super-region intercepts in k^{th} age term coefficient	$\psi_{k,j}^c$, $\psi_{k,l}^r$, $\psi_{k,m}^s$	$\psi_k^x \sim N(0, \sigma_{\psi,k,x}^2)$, $x \in \{c, r, s\}$	
standard deviation	$\sigma_{\psi,k,x}$	$\sigma_{\psi,k,x} \propto 1$, $x \in \{c, r, s\}$	
Global time slope in k^{th} age term coefficient	ϕ_k^g	$\phi_k^g \sim N(0, 1000)$	
Country, region and super-region time slope in k^{th} age term coefficient	$\phi_{k,j}^c$, $\phi_{k,l}^r$, $\phi_{k,m}^s$	$\phi_k^x \sim N(0, \sigma_{\phi,k,x}^2)$, $x \in \{c, r, s\}$	
standard deviation	$\sigma_{\phi,k,x}$	$\sigma_{\phi,k,x} \propto 1$, $x \in \{c, r, s\}$	
Study level fixed effects	β_p	$\beta_p \sim N(0, 1000), p \in \{1, \dots, 8\}$	
Study specific random effects	e_i	$e_i \sim N(0,\ v_{national})$<br>$e_i \sim N(0,\ v_{subnational})$<br>$e_i \sim N(0,\ v_{community})$	$v_{national} < v_{subnational} < v_{community}$
standard deviation	$\sqrt{v_{national}}$<br>$\sqrt{v_{subnational}}$<br>$\sqrt{v_{community}}$	$\sqrt{v_{national}} \propto 1$<br>$\sqrt{v_{subnational}} \propto 1$<br>$\sqrt{v_{community}} \propto 1$	
Standard deviation of residual age-by-study variability	τ	$\tau \propto 1$	

Supplementary Table 4. Results of model validation.

Test 1 and Test 2 are described in Methods.

Q1: first quartile; Q3: third quartile.

† Estimated values minus held-out values.

Children and adolescents, Test 1		Number of held out observations	Percent covered	Error (percentage points) †			Absolute error (percentage points)		
				Median	Q1	Q3	Median	Q1	Q3
Girls		7942	97%	0.61	-1.79	3.01	2.49	1.11	4.80
Super-region	Central Asia, Middle East and north Africa	666	98%	2.93	-0.23	5.59	4.56	2.30	6.96
	Central and eastern Europe	1364	98%	-0.53	-1.74	1.56	1.68	0.78	3.22
	East and southeast Asia	1399	96%	0.91	-0.94	2.46	1.97	0.93	3.28
	High-income western	2535	99%	0.96	-1.71	3.61	2.85	1.26	5.14
	Latin America and the Caribbean	741	96%	1.48	-2.51	4.94	3.89	1.95	6.24
	Pacific island nations	305	98%	-1.66	-8.65	4.59	6.78	3.53	10.62
	South Asia	46	96%	0.86	-1.56	3.78	3.09	1.36	4.09
	Sub-Saharan Africa	886	94%	0.14	-2.47	1.34	1.62	0.80	3.13
Study representativeness	National	5654	97%	0.20	-2.06	2.37	2.24	1.00	4.39
	Subnational	937	99%	1.71	-0.90	4.14	2.92	1.43	5.54
	Community	1351	99%	1.89	-0.82	4.49	3.53	1.56	5.80
Years	1980-2009	3511	97%	0.54	-1.36	2.54	2.02	0.87	3.89
	2010-2024	4431	98%	0.69	-2.23	3.45	2.94	1.37	5.57
Data density	Data poor	206	97%	1.25	-2.71	3.57	3.46	1.55	6.76
	Average data density	1222	94%	1.33	-0.80	3.09	2.23	1.13	4.25
	Data rich	6514	98%	0.44	-1.97	2.95	2.53	1.10	4.86
Boys		6723	97%	1.02	-2.22	4.49	3.53	1.48	6.88
Super-region	Central Asia, Middle East and north Africa	350	94%	1.62	-3.05	5.44	4.52	2.11	8.15
	Central and eastern Europe	567	97%	0.95	-2.01	4.68	3.24	1.44	5.96
	East and southeast Asia	2037	95%	0.48	-2.90	5.86	4.19	1.76	8.73
	High-income western	1772	99%	1.88	-1.43	5.09	3.79	1.72	6.85
	Latin America and the Caribbean	515	98%	0.96	-5.22	4.54	4.91	2.82	7.54
	Pacific island nations	132	92%	4.08	-0.27	9.57	6.53	2.97	12.59
	South Asia	491	98%	1.52	0.12	2.48	2.04	1.11	3.13
	Sub-Saharan Africa	859	96%	0.54	-1.74	1.81	1.81	0.70	4.77
Study representativeness	National	4677	96%	0.68	-2.67	3.96	3.36	1.41	6.59
	Subnational	949	99%	2.28	-0.54	7.21	4.13	1.71	8.76
	Community	1097	98%	1.41	-1.67	5.10	3.77	1.49	6.87
Years	1980-2009	3014	97%	0.69	-1.88	3.36	2.68	1.16	5.19
	2010-2024	3709	97%	1.39	-2.82	5.69	4.50	1.91	8.48
Data density	Data poor	136	96%	1.01	-3.09	3.18	3.18	1.57	5.40
	Average data density	919	94%	0.84	-0.76	3.63	2.47	0.83	5.82
	Data rich	5668	97%	1.08	-2.42	4.63	3.71	1.64	7.03

Children and adolescents, Test 2		Number of held out observations	Percent covered	Error (percentage points) †			Absolute error (percentage points)		
				Median	Q1	Q3	Median	Q1	Q3
Girls		8235	94%	0.17	-1.75	1.70	1.72	0.70	3.73
Super-region	Central Asia, Middle East and north Africa	635	91%	0.38	-3.61	2.86	3.10	1.44	5.97
	Central and eastern Europe	1184	99%	-0.06	-1.28	1.41	1.33	0.53	2.79
	East and southeast Asia	1662	92%	0.13	-0.98	1.13	1.07	0.45	2.24
	High-income western	2284	97%	0.13	-2.53	2.45	2.49	1.10	4.54
	Latin America and the Caribbean	964	95%	0.49	-1.78	2.30	2.10	0.96	4.22
	Pacific island nations	286	97%	-0.07	-6.86	4.53	5.62	2.96	8.96
	South Asia	354	83%	0.28	-0.97	0.78	0.79	0.43	1.69
	Sub-Saharan Africa	866	92%	0.33	-1.15	1.38	1.27	0.54	2.61
Study representativeness	National	5738	94%	0.09	-1.52	1.42	1.47	0.60	3.29
	Subnational	985	94%	0.47	-1.96	1.93	1.95	0.82	4.06
	Community	1512	96%	0.49	-2.67	2.85	2.77	1.27	4.98
Years	1980-2009	3542	95%	0.18	-1.35	1.38	1.36	0.54	2.83
	2010-2024	4693	94%	0.15	-2.15	2.13	2.14	0.86	4.42
Data density	Data poor	146	91%	0.75	-1.45	2.91	2.41	1.01	6.07
	Average data density	846	93%	0.41	-1.40	2.06	1.74	0.69	3.84
	Data rich	7243	95%	0.12	-1.79	1.65	1.70	0.69	3.69
Boys		7472	92%	0.63	-1.76	3.09	2.48	0.99	5.18
Super-region	Central Asia, Middle East and north Africa	610	86%	0.56	-3.90	3.37	3.60	1.77	6.75
	Central and eastern Europe	1173	95%	0.59	-1.93	2.97	2.54	1.07	4.60
	East and southeast Asia	1501	89%	1.03	-0.55	3.45	2.08	0.89	4.77
	High-income western	2230	95%	0.62	-2.21	3.58	2.95	1.31	5.84
	Latin America and the Caribbean	762	94%	0.67	-2.33	3.12	2.79	1.22	5.35
	Pacific island nations	215	88%	2.05	-5.40	6.92	6.61	3.30	10.38
	South Asia	354	85%	0.50	-1.06	0.94	0.98	0.60	1.84
	Sub-Saharan Africa	627	89%	0.22	-0.69	0.99	0.86	0.29	2.62
Study representativeness	National	5052	90%	0.51	-1.66	2.61	2.20	0.86	4.60
	Subnational	938	94%	1.21	-1.44	5.40	3.36	1.33	7.70
	Community	1482	96%	0.71	-2.41	3.81	3.18	1.32	6.13
Years	1980-2009	2122	88%	0.40	-1.53	2.16	1.88	0.75	4.13
	2010-2024	5350	93%	0.75	-1.91	3.51	2.75	1.10	5.55
Data density	Data poor	74	93%	-0.31	-3.40	1.87	2.49	0.64	4.53
	Average data density	794	92%	0.54	-1.04	2.64	1.98	0.72	4.94
	Data rich	6604	92%	0.66	-1.83	3.14	2.56	1.03	5.22

Adults, Test 1		Number of held out observations	Percent covered	Error (percentage points) †			Absolute error (percentage points)		
				Median	Q1	Q3	Median	Q1	Q3
Women		6988	89%	1.82	-2.87	5.43	4.57	2.17	8.56
Super-region	Central Asia, Middle East and north Africa	357	89%	1.32	-5.51	4.81	4.99	2.64	11.00
	Central and eastern Europe	403	94%	1.46	-3.51	4.73	4.37	2.19	7.70
	East and southeast Asia	1490	86%	2.43	0.34	4.68	3.28	1.56	5.35
	High-income western	1957	93%	2.00	-3.28	6.69	5.31	2.62	9.54
	Latin America and the Caribbean	1357	96%	2.65	-2.44	6.79	5.19	2.57	9.13
	Pacific island nations	175	70%	-4.51	-17.07	8.03	13.27	7.12	20.96
	South Asia	262	77%	1.76	-3.01	5.29	5.06	2.27	8.26
	Sub-Saharan Africa	987	81%	0.11	-6.69	2.96	4.09	1.57	10.08
Study representativeness	National	3587	88%	1.44	-2.90	4.23	3.83	1.81	7.24
	Subnational	1323	87%	3.42	-2.14	8.20	5.93	2.98	10.50
	Community	2078	91%	2.10	-3.25	6.18	5.31	2.55	9.54
Years	1980-2009	3914	88%	1.56	-2.79	5.16	4.33	1.96	8.11
	2010-2024	3074	90%	2.17	-2.96	5.78	4.85	2.43	9.30
Data density	Data poor	424	86%	-1.59	-7.21	3.81	5.26	2.74	10.39
	Average data density	1517	86%	1.40	-4.09	5.02	4.73	2.08	9.73
	Data rich	5047	90%	2.14	-2.12	5.62	4.49	2.13	8.26
Men		7160	90%	1.46	-1.05	4.17	3.12	1.34	6.58
Super-region	Central Asia, Middle East and north Africa	498	78%	-0.57	-10.05	5.46	7.13	3.37	12.57
	Central and eastern Europe	156	99%	0.21	-3.10	2.79	2.85	1.50	5.35
	East and southeast Asia	1649	90%	1.48	0.46	2.83	1.69	0.87	3.10
	High-income western	1871	95%	2.43	-1.21	6.40	4.14	2.01	8.11
	Latin America and the Caribbean	1067	93%	2.84	-1.69	6.92	5.46	2.42	8.90
	Pacific island nations	214	74%	4.94	-11.64	14.85	13.98	7.45	21.19
	South Asia	851	88%	1.48	-0.16	3.12	2.31	1.14	4.07
	Sub-Saharan Africa	854	90%	0.29	-3.22	1.60	2.13	0.84	4.62
Study representativeness	National	3482	87%	1.20	-0.85	3.57	2.69	1.10	6.18
	Subnational	1201	92%	2.08	-0.82	5.11	3.71	1.69	6.66
	Community	2477	95%	1.69	-1.39	4.51	3.51	1.62	6.90
Years	1980-2009	4264	92%	1.14	-1.15	3.48	2.70	1.14	5.74
	2010-2024	2896	88%	2.13	-0.76	5.28	3.79	1.76	7.87
Data density	Data poor	233	89%	1.47	-3.32	4.91	4.19	1.97	9.36
	Average data density	1434	84%	0.74	-3.73	5.01	4.51	1.68	9.12
	Data rich	5493	92%	1.59	-0.45	4.02	2.84	1.27	5.82

Adults, Test 2		Number of held out observations	Percent covered	Error (percentage points) †			Absolute error (percentage points)		
				Median	Q1	Q3	Median	Q1	Q3
Women		7291	87%	0.21	-2.52	2.60	2.58	0.99	5.65
Super-region	Central Asia, Middle East and north Africa	832	79%	1.05	-4.26	6.08	5.22	2.31	10.01
	Central and eastern Europe	549	87%	0.01	-2.82	3.02	2.87	1.17	6.29
	East and southeast Asia	1340	88%	0.17	-0.93	1.21	1.05	0.42	2.58
	High-income western	2067	92%	0.07	-2.92	3.12	3.04	1.43	6.04
	Latin America and the Caribbean	884	90%	0.23	-3.87	3.35	3.59	1.53	6.73
	Pacific island nations	137	87%	-1.26	-6.71	5.51	6.26	2.44	12.37
	South Asia	576	78%	0.29	-2.15	1.59	1.78	0.76	3.82
	Sub-Saharan Africa	906	87%	0.29	-1.96	2.27	2.22	0.89	4.67
Study representativeness	National	3874	87%	0.12	-1.82	1.92	1.88	0.70	4.20
	Subnational	1255	89%	0.06	-3.09	2.92	2.99	1.31	5.86
	Community	2162	87%	0.63	-4.13	3.94	4.00	1.75	8.18
Years	1980-2009	3771	87%	0.12	-2.49	2.14	2.28	0.85	5.08
	2010-2024	3520	87%	0.38	-2.56	3.16	2.89	1.19	6.12
Data density	Data poor	296	90%	0.29	-3.79	3.73	3.78	1.57	6.61
	Average data density	852	87%	0.38	-2.40	2.28	2.35	1.08	5.59
	Data rich	6143	87%	0.19	-2.49	2.60	2.56	0.97	5.61
Men		7105	91%	0.15	-2.20	2.21	2.21	0.81	4.97
Super-region	Central Asia, Middle East and north Africa	820	89%	-0.66	-4.67	2.62	3.60	1.64	6.79
	Central and eastern Europe	676	91%	0.23	-2.20	2.95	2.73	0.89	5.77
	East and southeast Asia	1188	92%	0.10	-0.77	0.95	0.87	0.35	1.81
	High-income western	2218	94%	0.10	-2.65	3.12	2.89	1.13	5.34
	Latin America and the Caribbean	768	91%	0.55	-3.28	3.48	3.46	1.55	6.61
	Pacific island nations	177	73%	1.13	-7.33	9.77	8.84	4.57	14.08
	South Asia	566	86%	0.30	-1.16	1.12	1.15	0.53	2.17
	Sub-Saharan Africa	692	88%	0.30	-1.51	1.70	1.61	0.59	3.74
Study representativeness	National	3958	90%	0.09	-1.65	1.77	1.71	0.62	4.04
	Subnational	1204	91%	0.02	-3.22	2.48	2.84	1.01	5.65
	Community	1943	93%	0.46	-3.08	3.10	3.09	1.29	6.39
Years	1980-2009	3401	92%	0.08	-2.22	1.84	1.99	0.72	4.64
	2010-2024	3704	90%	0.24	-2.18	2.61	2.45	0.90	5.26
Data density	Data poor	166	95%	0.63	-2.89	3.08	2.94	1.44	6.38
	Average data density	1045	85%	0.24	-3.09	2.94	3.02	1.21	6.39
	Data rich	5894	92%	0.12	-2.07	2.06	2.07	0.76	4.71

Supplementary Table 5. Average Jaccard index of the clusters.

	Percent of countries used in subsamples				
**Cluster**	**90%**	**80%**	**70%**	**60%**	**50%**
Girls					
Recent acceleration	0.98	0.96	0.93	0.89	0.86
Accelerating or steady increase	0.93	0.91	0.88	0.86	0.83
Decelerating increase	0.81	0.78	0.74	0.72	0.69
Plateau	0.87	0.85	0.83	0.82	0.80
Approximately flat	0.94	0.89	0.85	0.82	0.78
Early decline	0.95	0.92	0.91	0.90	0.89
Boys					
Recent acceleration	0.95	0.92	0.87	0.84	0.80
Accelerating increase	0.91	0.87	0.83	0.80	0.76
Steady or decelerating increase	0.95	0.92	0.89	0.87	0.83
Plateau	0.97	0.93	0.89	0.86	0.83
Decline	1.00	1.00	1.00	1.00	1.00
Recent decline	0.89	0.77	0.67	0.61	0.59
Women					
Accelerating increase	0.97	0.95	0.93	0.89	0.86
Steady increase	0.95	0.89	0.85	0.80	0.77
Decelerating increase or plateau	0.90	0.79	0.73	0.67	0.62
Approximately flat	0.95	0.89	0.84	0.81	0.78
Early decline	1.00	1.00	1.00	0.99	0.98
Recent decline	1.00	1.00	1.00	0.99	0.99
Men					
Recent acceleration	0.94	0.89	0.86	0.83	0.82
Accelerating increase	0.95	0.92	0.90	0.88	0.87
Steady increase	0.97	0.93	0.91	0.88	0.85
Decelerating increase	0.94	0.89	0.85	0.80	0.77
Plateau	0.84	0.75	0.71	0.67	0.66
Recent decline	0.74	0.65	0.62	0.63	0.66

Supplementary Fig. 1. Number of data sources used in the analysis, by country.

School-aged children and adolescents

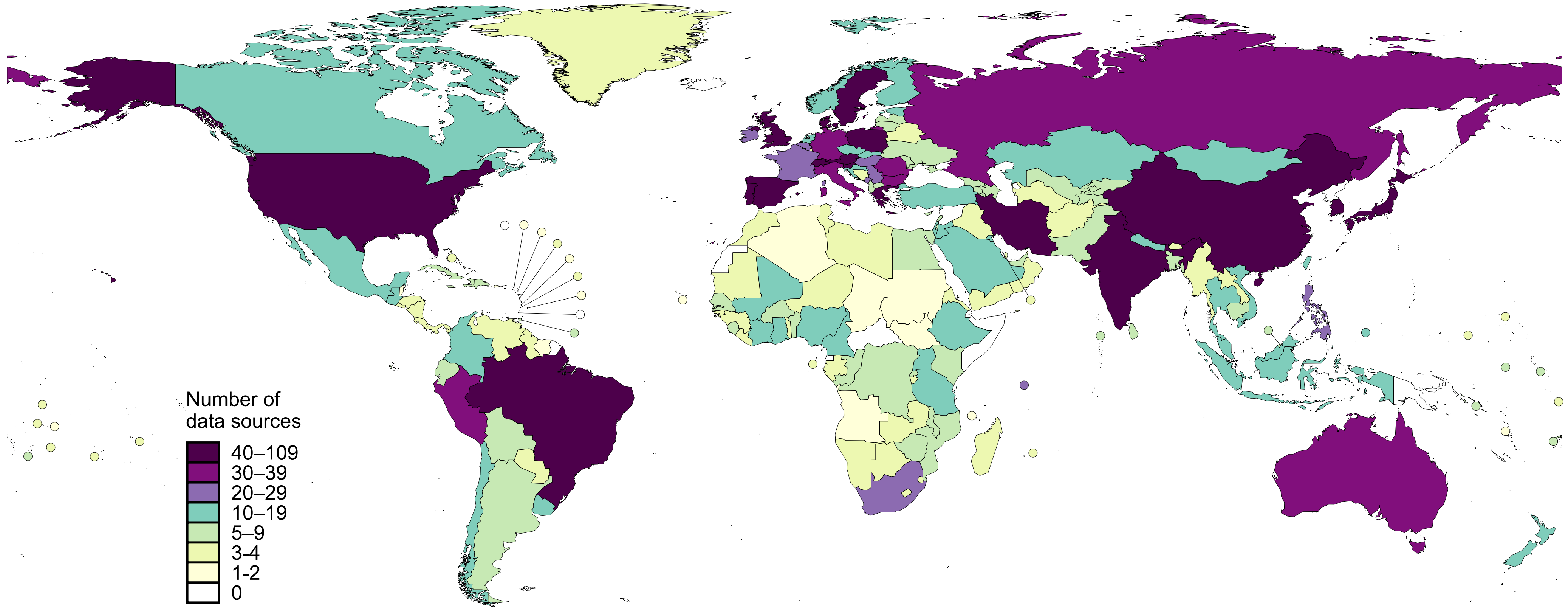

Adults

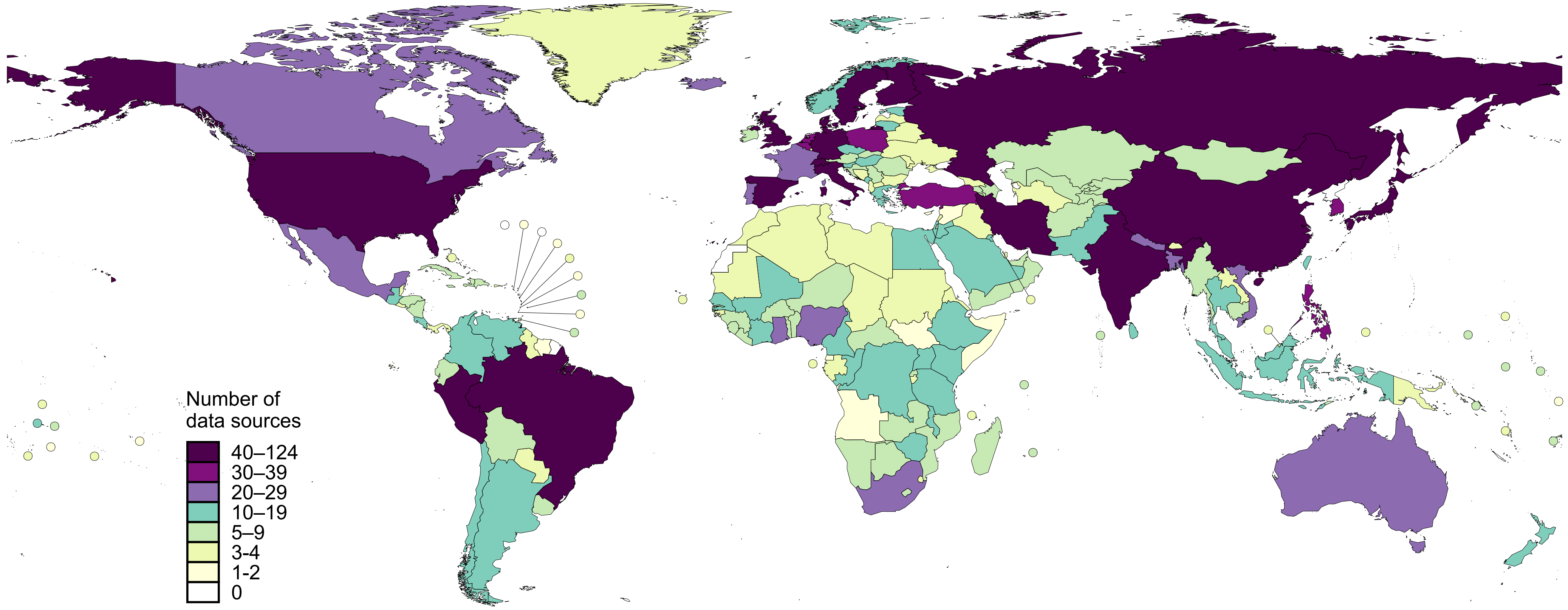

Supplementary Fig. 2. Number of data sources used in the analysis, by region and year.

The size of each circle shows the number of data sources for each region and year, and the colours indicate the relative count of national, subnational and community data sources.

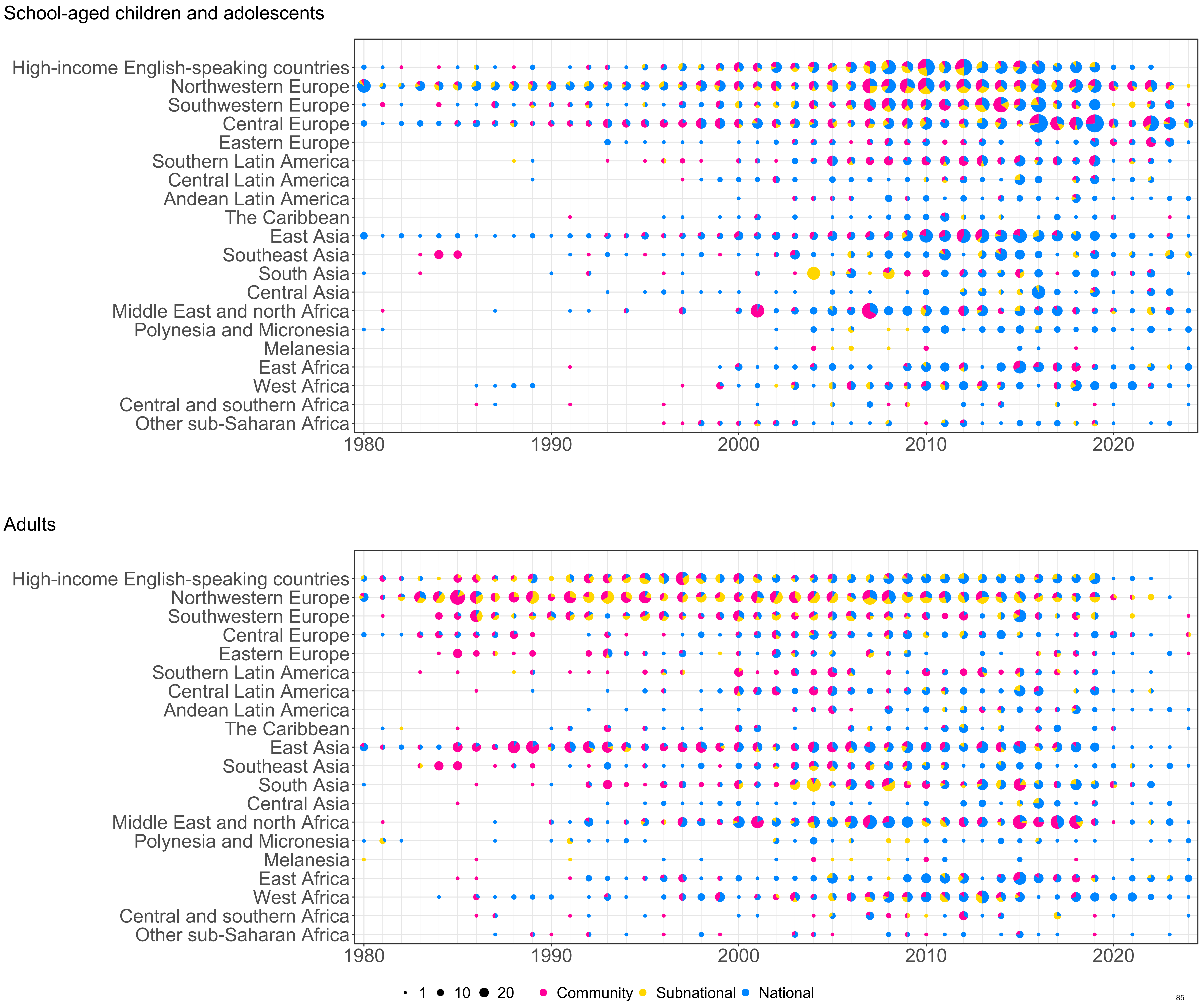
School-aged children and adolescents
High-income English-speaking countries
Northwestern Europe
Southwestern Europe
Central Europe
Eastern Europe
Southern Latin America
Central Latin America
Andean Latin America
The Caribbean
East Asia
Southeast Asia
South Asia
Central Asia
Middle East and north Africa
Polynesia and Micronesia
Melanesia
East Africa
West Africa
Central and southern Africa
Other sub-Saharan Africa
1980
1990
2000
2010
2020
Adults
High-income English-speaking countries
Northwestern Europe
Southwestern Europe
Central Europe
Eastern Europe
Southern Latin America
Central Latin America
Andean Latin America
The Caribbean
East Asia
Southeast Asia
South Asia
Central Asia
Middle East and north Africa
Polynesia and Micronesia
Melanesia
East Africa
West Africa
Central and southern Africa
Other sub-Saharan Africa
1980
1990
2000
2010
2020
1
10
20
Community
Subnational
National

Supplementary Fig. 3. Age-standardised prevalence of obesity in children and adolescents from 1980 to 2024 by country.

The shaded areas around the lines show the 95% credible intervals of the estimates. Countries are labelled by their ISO 3166-1 alpha-3 codes (Supplementary Note 1) and coloured by their cluster allocation.

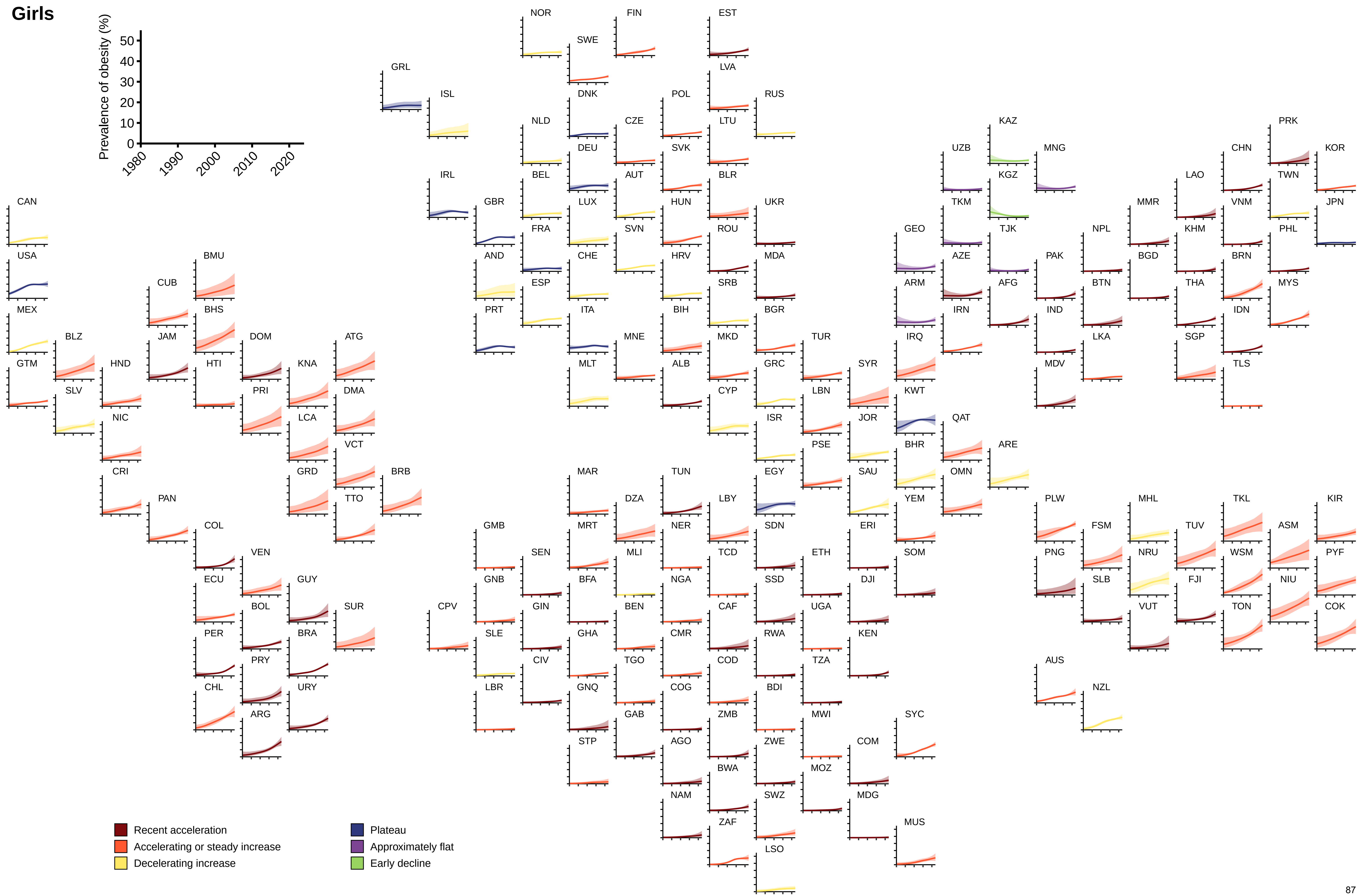
Girls
Prevalence of obesity (%)
50
40
30
20
10
0
1980
1990
2000
2010
2020
NOR FIN EST
SWE
GRL LVA
ISL DNK POL RUS
NLD CZE LTU KAZ PRK
DEU SVK UZB MNG CHN KOR
IRL BEL AUT BLR KGZ LAO TWN
CAN GBR LUX HUN UKR TKM MMR VNM JPN
FRA SVN ROU GEO TJK NPL KHM PHL
USA BMU AND CHE HRV MDA AZE PAK BGD BRN
CUB ESP SRB ARM AFG BTN THA MYS
MEX BHS PRT ITA BIH BGR IRN IND IDN
BLZ JAM DOM ATG MNE MKD TUR IRQ LKA SGP
GTM HND HTI KNA MLT ALB GRC SYR MDV TLS
SLV PRI DMA CYP LBN KWT
NIC LCA ISR JOR QAT
VCT PSE BHR ARE
CRI GRD BRB MAR TUN EGY SAU OMN
PAN TTO DZA LBY YEM PLW MHL TKL KIR
COL GMB MRT NER SDN ERI FSM TUV ASM
VEN SEN MLI TCD ETH SOM PNG NRU WSM PYF
ECU GUY GNB BFA NGA SSD DJI SLB FJI NIU
BOL SUR CPV GIN BEN CAF UGA VUT TON COK
PER BRA SLE GHA CMR RWA KEN
PRY CIV TGO COD TZA AUS
CHL URY LBR GNQ COG BDI NZL
ARG GAB ZMB MWI SYC
STP AGO ZWE COM
BWA MOZ
NAM SWZ MDG
ZAF MUS
LSO
Recent acceleration
Accelerating or steady increase
Decelerating increase
Plateau
Approximately flat
Early decline

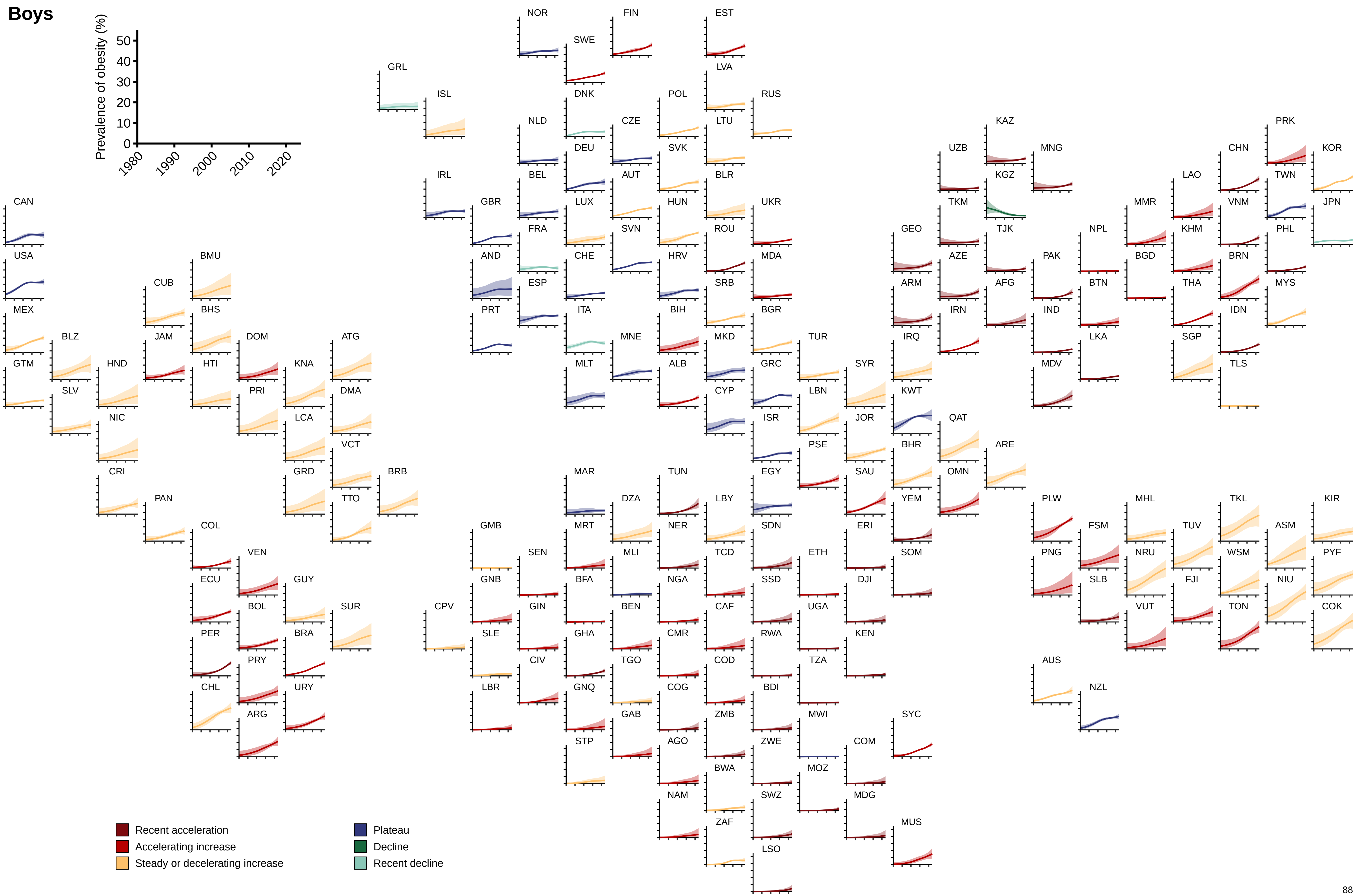
Boys
Prevalence of obesity (%)
0
10
20
30
40
50
1980
1990
2000
2010
2020
NOR FIN EST SWE GRL LVA ISL DNK POL RUS NLD CZE LTU KAZ PRK DEU SVK UZB MNG CHN KOR IRL BEL AUT BLR KGZ LAO TWN CAN GBR LUX HUN UKR TKM MMR VNM JPN FRA SVN ROU GEO TJK NPL KHM PHL USA BMU AND CHE HRV MDA AZE PAK BGD BRN CUB ESP SRB ARM AFG BTN THA MYS MEX BHS PRT ITA BIH BGR IRN IND IDN BLZ JAM DOM ATG MNE MKD TUR IRQ LKA SGP GTM HND HTI KNA MLT ALB GRC SYR MDV TLS SLV PRI DMA CYP LBN KWT NIC LCA ISR JOR QAT VCT PSE BHR ARE CRI GRD BRB MAR TUN EGY SAU OMN PAN TTO DZA LBY YEM PLW MHL TKL KIR COL GMB MRT NER SDN ERI FSM TUV ASM VEN SEN MLI TCD ETH SOM PNG NRU WSM PYF ECU GUY GNB BFA NGA SSD DJI SLB FJI NIU BOL SUR CPV GIN BEN CAF UGA VUT TON COK PER BRA SLE GHA CMR RWA KEN PRY CIV TGO COD TZA AUS CHL URY LBR GNQ COG BDI NZL ARG GAB ZMB MWI SYC STP AGO ZWE COM BWA MOZ NAM SWZ MDG ZAF MUS LSO
Recent acceleration
Accelerating increase
Steady or decelerating increase
Plateau
Decline
Recent decline

Supplementary Fig. 4. Age-standardised prevalence of obesity in adults from 1980 to 2024 by country.

The shaded areas around the lines show the 95% credible intervals of the estimates. Countries are labelled by their ISO 3166-1 alpha-3 codes (Supplementary Note 1) and coloured by their cluster allocation.

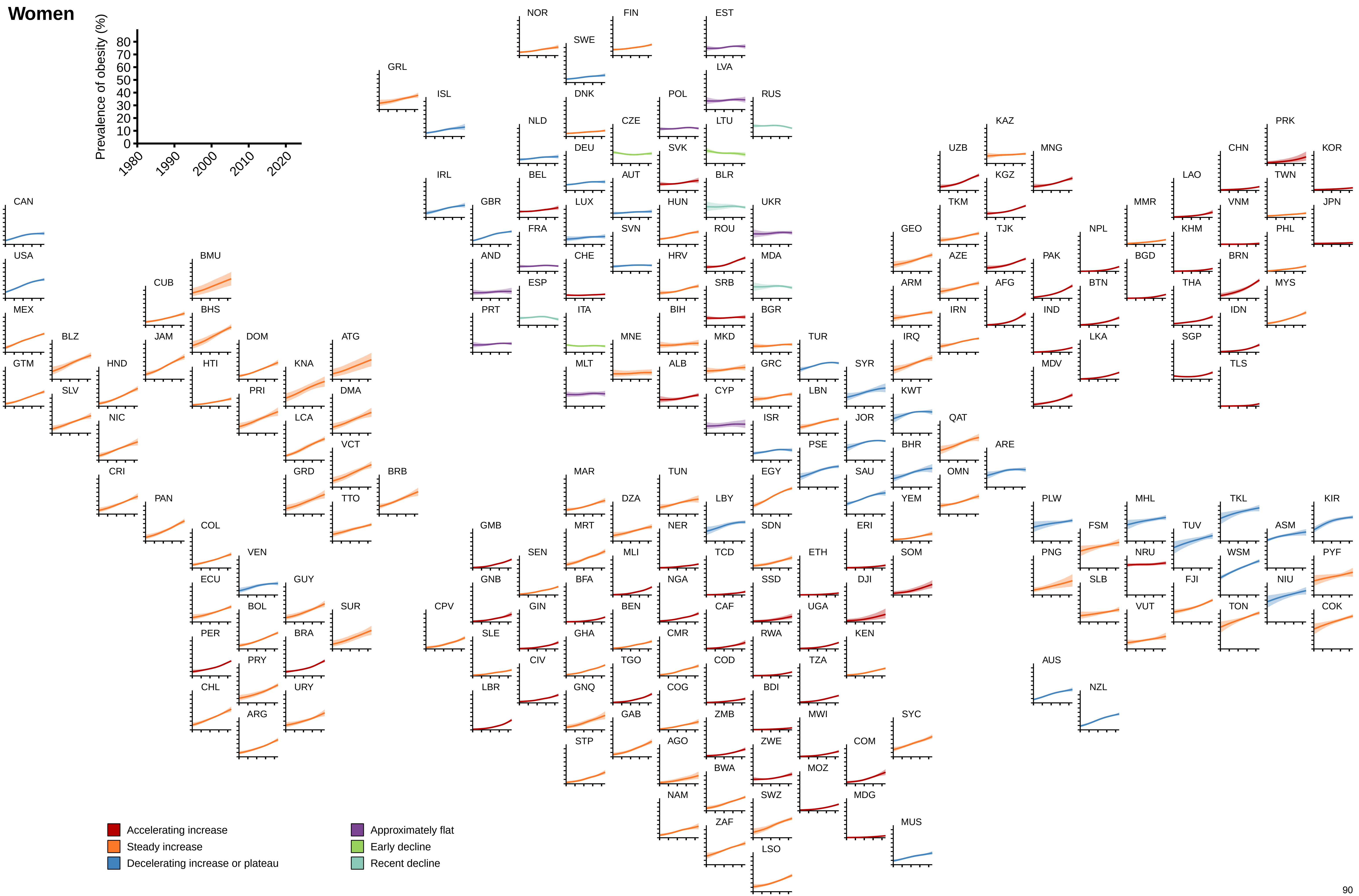
Women
Prevalence of obesity (%)
80
70
60
50
40
30
20
10
0
1980
1990
2000
2010
2020
NOR FIN EST
SWE
GRL LVA
ISL DNK POL RUS
NLD CZE LTU KAZ PRK
DEU SVK UZB MNG CHN KOR
IRL BEL AUT BLR KGZ LAO TWN
CAN GBR LUX HUN UKR TKM MMR VNM JPN
FRA SVN ROU GEO TJK NPL KHM PHL
USA BMU AND CHE HRV MDA AZE PAK BGD BRN
CUB ESP SRB ARM AFG BTN THA MYS
MEX BHS PRT ITA BIH BGR IRN IND IDN
BLZ JAM DOM ATG MNE MKD TUR IRQ LKA SGP
GTM HND HTI KNA MLT ALB GRC SYR MDV TLS
SLV PRI DMA CYP LBN KWT
NIC LCA ISR JOR QAT
VCT PSE BHR ARE
CRI GRD BRB MAR TUN EGY SAU OMN
PAN TTO DZA LBY YEM PLW MHL TKL KIR
COL GMB MRT NER SDN ERI FSM TUV ASM
VEN SEN MLI TCD ETH SOM PNG NRU WSM PYF
ECU GUY GNB BFA NGA SSD DJI SLB FJI NIU
BOL SUR CPV GIN BEN CAF UGA VUT TON COK
PER BRA SLE GHA CMR RWA KEN
PRY CIV TGO COD TZA AUS
CHL URY LBR GNQ COG BDI NZL
ARG GAB ZMB MWI SYC
STP AGO ZWE COM
BWA MOZ
NAM SWZ MDG
ZAF MUS
LSO
Accelerating increase
Steady increase
Decelerating increase or plateau
Approximately flat
Early decline
Recent decline

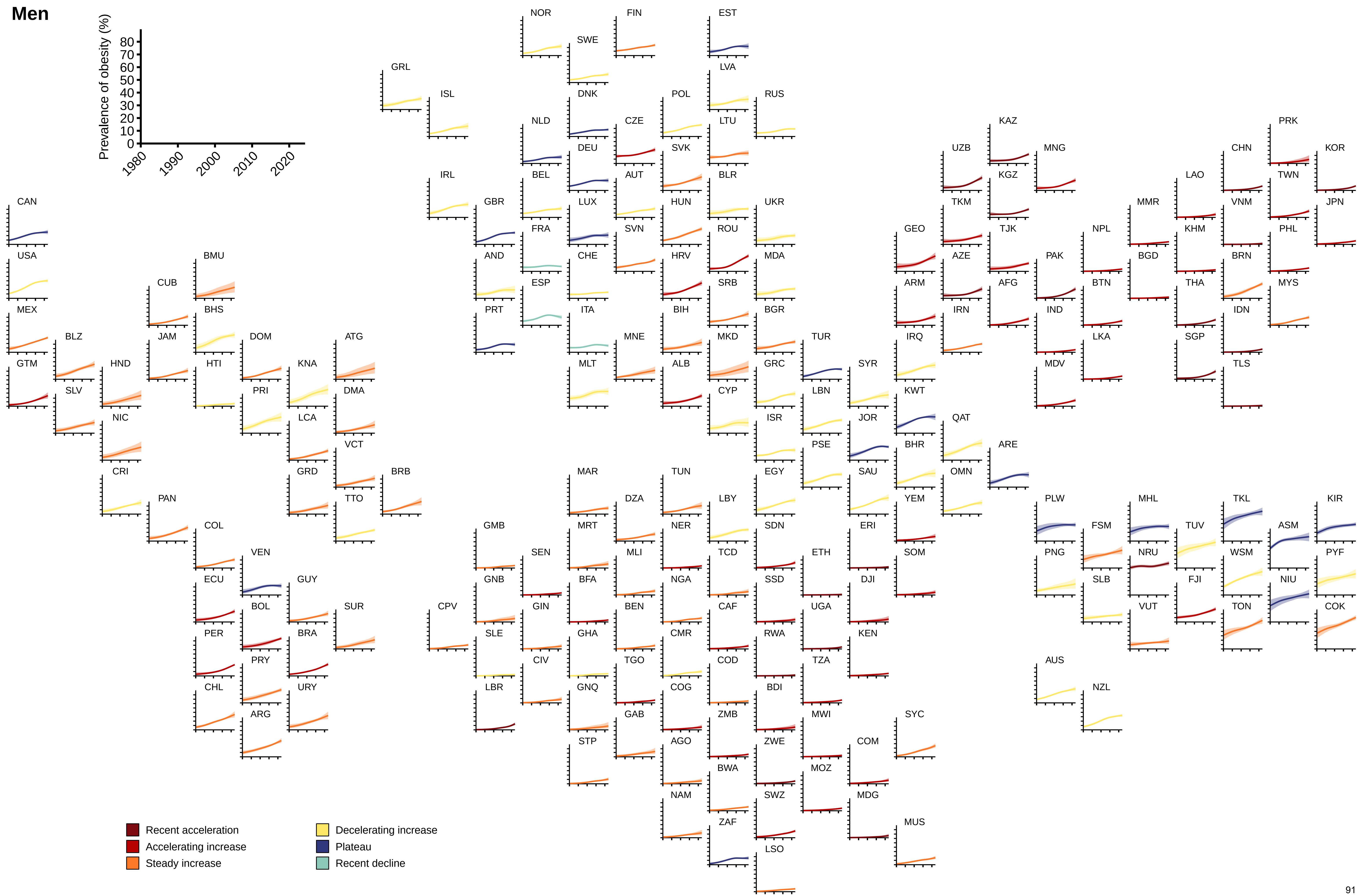
Men
Prevalence of obesity (%)
80
70
60
50
40
30
20
10
0
1980
1990
2000
2010
2020
NOR FIN EST
SWE
GRL LVA
ISL DNK POL RUS
NLD CZE LTU KAZ PRK
DEU SVK UZB MNG CHN KOR
IRL BEL AUT BLR KGZ LAO TWN
CAN GBR LUX HUN UKR TKM MMR VNM JPN
FRA SVN ROU GEO TJK NPL KHM PHL
USA BMU AND CHE HRV MDA AZE PAK BGD BRN
CUB ESP SRB ARM AFG BTN THA MYS
MEX BHS PRT ITA BIH BGR IRN IND IDN
BLZ JAM DOM ATG MNE MKD TUR IRQ LKA SGP
GTM HND HTI KNA MLT ALB GRC SYR MDV TLS
SLV PRI DMA CYP LBN KWT
NIC LCA ISR JOR QAT
VCT PSE BHR ARE
CRI GRD BRB MAR TUN EGY SAU OMN
PAN TTO DZA LBY YEM PLW MHL TKL KIR
COL GMB MRT NER SDN ERI FSM TUV ASM
VEN SEN MLI TCD ETH SOM PNG NRU WSM PYF
ECU GUY GNB BFA NGA SSD DJI SLB FJI NIU
BOL SUR CPV GIN BEN CAF UGA VUT TON COK
PER BRA SLE GHA CMR RWA KEN
PRY CIV TGO COD TZA AUS
CHL URY LBR GNQ COG BDI NZL
ARG GAB ZMB MWI SYC
STP AGO ZWE COM
BWA MOZ
NAM SWZ MDG
ZAF MUS
LSO
Recent acceleration
Accelerating increase
Steady increase
Decelerating increase
Plateau
Recent decline

Supplementary Fig. 5. Velocity of obesity in children and adolescents from 1980 to 2024 by country.

The shaded areas around the lines show the 95% credible intervals of the estimates. Countries are labelled by their ISO 3166-1 alpha-3 codes (Supplementary Note 1) and coloured by their cluster allocation.

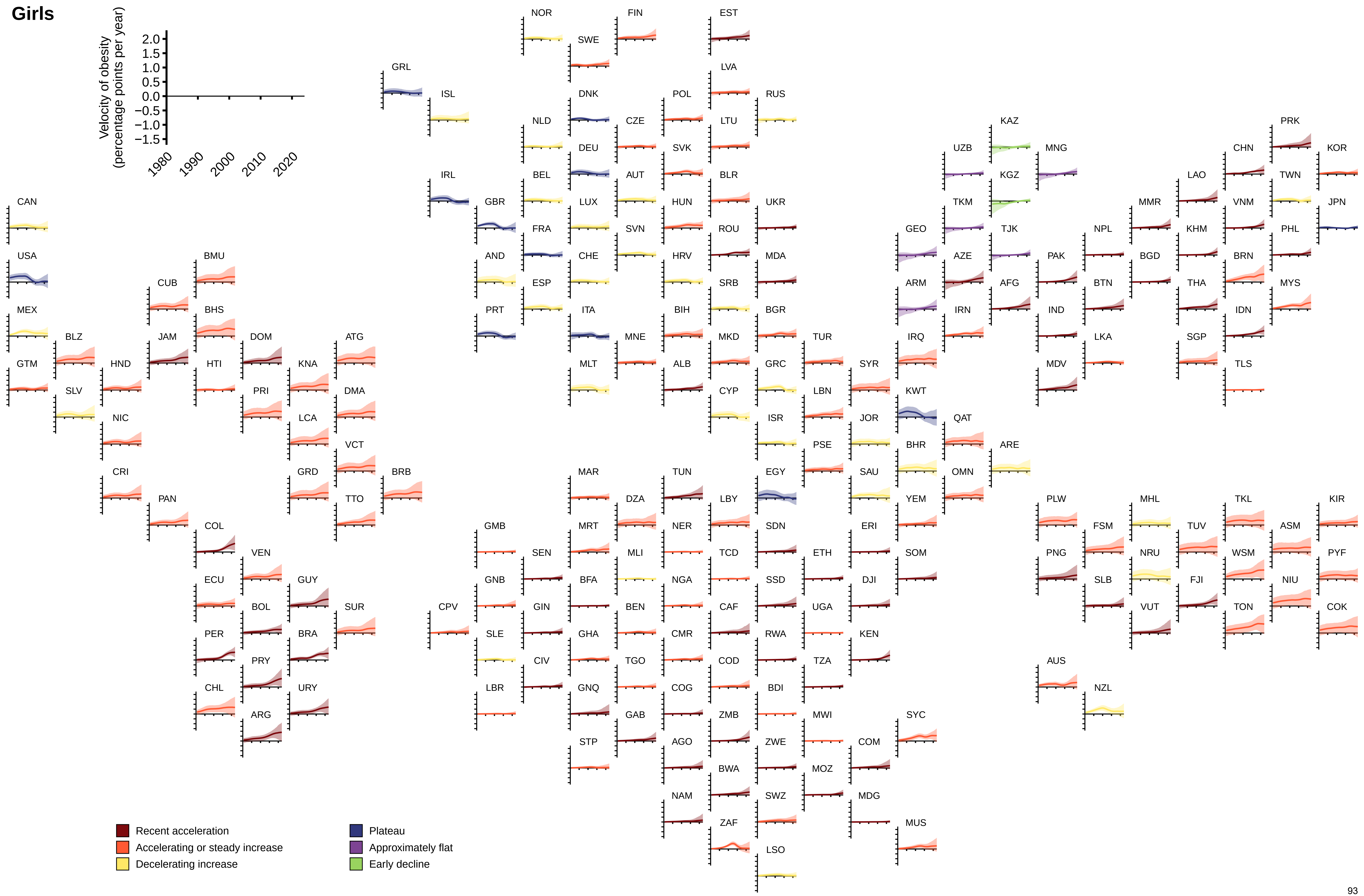
Girls
Velocity of obesity (percentage points per year)
2.0
1.5
1.0
0.5
0.0
−0.5
−1.0
−1.5
1980
1990
2000
2010
2020
NOR FIN EST
SWE
GRL LVA
ISL DNK POL RUS
NLD CZE LTU KAZ PRK
DEU SVK UZB MNG CHN KOR
IRL BEL AUT BLR KGZ LAO TWN
CAN GBR LUX HUN UKR TKM MMR VNM JPN
FRA SVN ROU GEO TJK NPL KHM PHL
USA BMU AND CHE HRV MDA AZE PAK BGD BRN
CUB ESP SRB ARM AFG BTN THA MYS
MEX BHS PRT ITA BIH BGR IRN IND IDN
BLZ JAM DOM ATG MNE MKD TUR IRQ LKA SGP
GTM HND HTI KNA MLT ALB GRC SYR MDV TLS
SLV PRI DMA CYP LBN KWT
NIC LCA ISR JOR QAT
VCT PSE BHR ARE
CRI GRD BRB MAR TUN EGY SAU OMN
PAN TTO DZA LBY YEM PLW MHL TKL KIR
COL GMB MRT NER SDN ERI FSM TUV ASM
VEN SEN MLI TCD ETH SOM PNG NRU WSM PYF
ECU GUY GNB BFA NGA SSD DJI SLB FJI NIU
BOL SUR CPV GIN BEN CAF UGA VUT TON COK
PER BRA SLE GHA CMR RWA KEN
PRY CIV TGO COD TZA AUS
CHL URY LBR GNQ COG BDI NZL
ARG GAB ZMB MWI SYC
STP AGO ZWE COM
BWA MOZ
NAM SWZ MDG
ZAF MUS
LSO
Recent acceleration
Accelerating or steady increase
Decelerating increase
Plateau
Approximately flat
Early decline

Boys

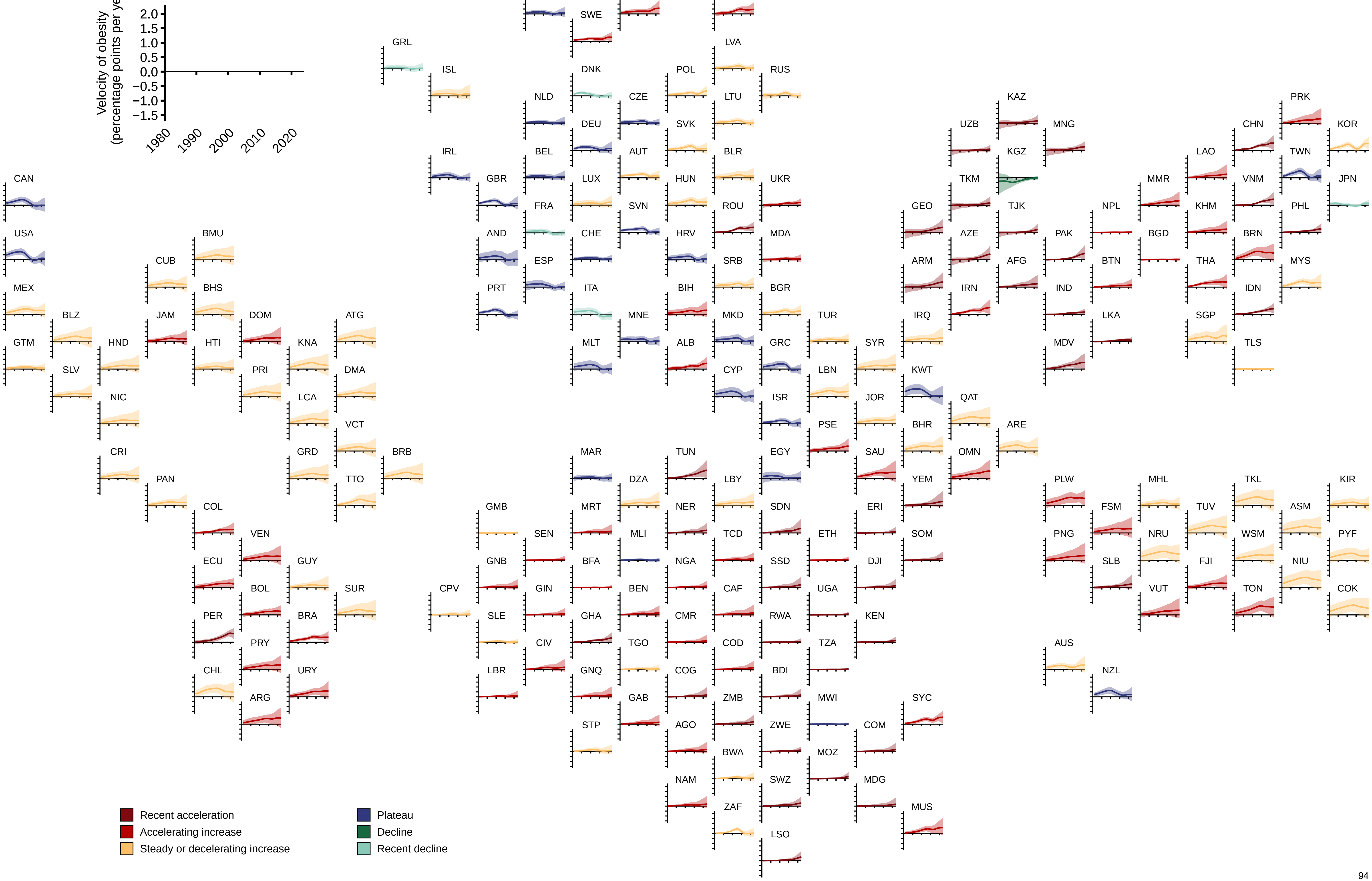
Velocity of obesity
(percentage points per year)
2.0
1.5
1.0
0.5
0.0
−0.5
−1.0
−1.5
1980
1990
2000
2010
2020
NOR FIN EST
SWE
GRL LVA
ISL DNK POL RUS
NLD CZE LTU KAZ PRK
DEU SVK UZB MNG CHN KOR
IRL BEL AUT BLR KGZ LAO TWN
CAN GBR LUX HUN UKR TKM MMR VNM JPN
FRA SVN ROU GEO TJK NPL KHM PHL
USA BMU AND CHE HRV MDA AZE PAK BGD BRN
CUB ESP SRB ARM AFG BTN THA MYS
MEX BHS PRT ITA BIH BGR IRN IND IDN
BLZ JAM DOM ATG MNE MKD TUR IRQ LKA SGP
GTM HND HTI KNA MLT ALB GRC SYR MDV TLS
SLV PRI DMA CYP LBN KWT
NIC LCA ISR JOR QAT
VCT PSE BHR ARE
CRI GRD BRB MAR TUN EGY SAU OMN
PAN TTO DZA LBY YEM PLW MHL TKL KIR
COL GMB MRT NER SDN ERI FSM TUV ASM
VEN SEN MLI TCD ETH SOM PNG NRU WSM PYF
ECU GUY GNB BFA NGA SSD DJI SLB FJI NIU
BOL SUR CPV GIN BEN CAF UGA VUT TON COK
PER BRA SLE GHA CMR RWA KEN
PRY CIV TGO COD TZA AUS
CHL URY LBR GNQ COG BDI NZL
ARG GAB ZMB MWI SYC
STP AGO ZWE COM
BWA MOZ
NAM SWZ MDG
ZAF MUS
LSO
Recent acceleration
Accelerating increase
Steady or decelerating increase
Plateau
Decline
Recent decline

Supplementary Fig. 6. Velocity of obesity in adults from 1980 to 2024 by country.

The shaded areas around the lines show the 95% credible intervals of the estimates. Countries are labelled by their ISO 3166-1 alpha-3 codes (Supplementary Note 1) and coloured by their cluster allocation.

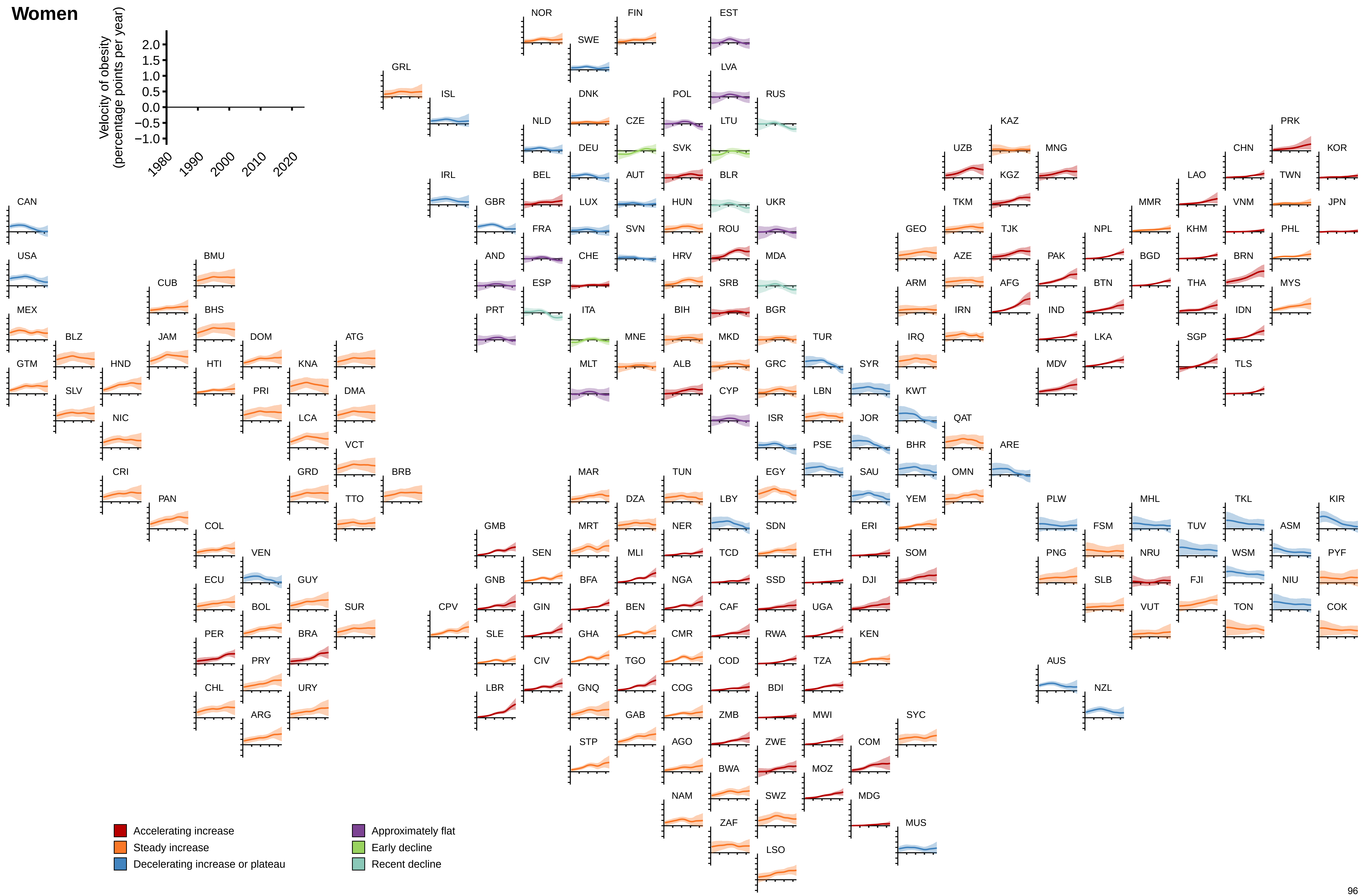
Women
Velocity of obesity
(percentage points per year)
2.0
1.5
1.0
0.5
0.0
−0.5
−1.0
1980
1990
2000
2010
2020
NOR
FIN
EST
SWE
GRL
LVA
ISL
DNK
POL
RUS
NLD
CZE
LTU
KAZ
PRK
DEU
SVK
UZB
MNG
CHN
KOR
IRL
BEL
AUT
BLR
KGZ
LAO
TWN
CAN
GBR
LUX
HUN
UKR
TKM
MMR
VNM
JPN
FRA
SVN
ROU
GEO
TJK
NPL
KHM
PHL
USA
BMU
AND
CHE
HRV
MDA
AZE
PAK
BGD
BRN
CUB
ESP
SRB
ARM
AFG
BTN
THA
MYS
MEX
BHS
PRT
ITA
BIH
BGR
IRN
IND
IDN
BLZ
JAM
DOM
ATG
MNE
MKD
TUR
IRQ
LKA
SGP
GTM
HND
HTI
KNA
MLT
ALB
GRC
SYR
MDV
TLS
SLV
PRI
DMA
CYP
LBN
KWT
NIC
LCA
ISR
JOR
QAT
VCT
PSE
BHR
ARE
CRI
GRD
BRB
MAR
TUN
EGY
SAU
OMN
PAN
TTO
DZA
LBY
YEM
PLW
MHL
TKL
KIR
COL
GMB
MRT
NER
SDN
ERI
FSM
ASM
VEN
SEN
MLI
TCD
ETH
SOM
PNG
NRU
TUV
WSM
PYF
ECU
GUY
GNB
BFA
NGA
SSD
DJI
SLB
FJI
NIU
BOL
SUR
CPV
GIN
BEN
CAF
UGA
VUT
TON
COK
PER
BRA
SLE
GHA
CMR
RWA
KEN
PRY
CIV
TGO
COD
TZA
AUS
CHL
URY
LBR
GNQ
COG
BDI
NZL
ARG
GAB
ZMB
MWI
SYC
STP
AGO
ZWE
COM
BWA
MOZ
NAM
SWZ
MDG
ZAF
MUS
LSO
Accelerating increase
Steady increase
Decelerating increase or plateau
Approximately flat
Early decline
Recent decline

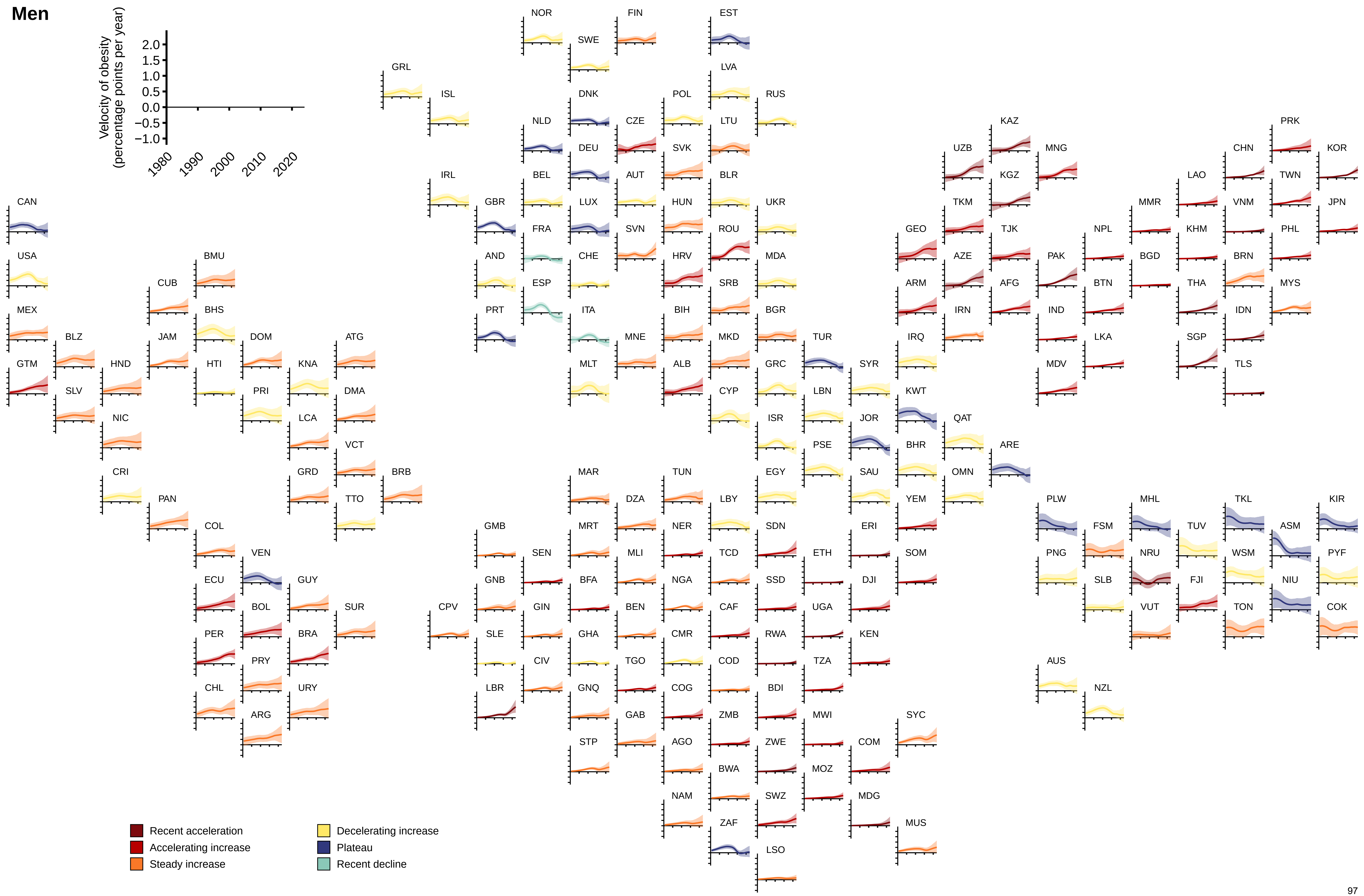

Men
Velocity of obesity (percentage points per year)
2.0
1.5
1.0
0.5
0.0
−0.5
−1.0
1980
1990
2000
2010
2020
NOR FIN EST
SWE
GRL LVA
ISL DNK POL RUS
NLD CZE LTU KAZ PRK
DEU SVK UZB MNG CHN KOR
IRL BEL AUT BLR KGZ LAO TWN
CAN GBR LUX HUN UKR TKM MMR VNM JPN
FRA SVN ROU GEO TJK NPL KHM PHL
USA BMU AND CHE HRV MDA AZE PAK BGD BRN
CUB ESP SRB ARM AFG BTN THA MYS
MEX BHS PRT ITA BIH BGR IRN IND IDN
BLZ JAM DOM ATG MNE MKD TUR IRQ LKA SGP
GTM HND HTI KNA MLT ALB GRC SYR MDV TLS
SLV PRI DMA CYP LBN KWT
NIC LCA ISR JOR QAT
VCT PSE BHR ARE
CRI GRD BRB MAR TUN EGY SAU OMN
PAN TTO DZA LBY YEM PLW MHL TKL KIR
COL GMB MRT NER SDN ERI FSM TUV ASM
VEN SEN MLI TCD ETH SOM PNG NRU WSM PYF
ECU GUY GNB BFA NGA SSD DJI SLB FJI NIU
BOL SUR CPV GIN BEN CAF UGA VUT TON COK
PER BRA SLE GHA CMR RWA KEN
PRY CIV TGO COD TZA AUS
CHL URY LBR GNQ COG BDI NZL
ARG GAB ZMB MWI SYC
STP AGO ZWE COM
BWA MOZ
NAM SWZ MDG
ZAF MUS
LSO
Recent acceleration
Accelerating increase
Steady increase
Decelerating increase
Plateau
Recent decline

References

1 NCD Risk Factor Collaboration (NCD-RisC). Worldwide trends in underweight and obesity from 1990 to 2022: a pooled analysis of 3663 population-representative studies with 222 million children, adolescents, and adults. *Lancet* **403**, 1027-1050 (2024). https://doi.org/10.1016/S0140-6736(23)02750-2

2 NCD Risk Factor Collaboration (NCD-RisC). Diminishing benefits of urban living for children and adolescents' growth and development. *Nature* **615**, 874-883 (2023). https://doi.org/10.1038/s41586-023-05772-8

3 NCD Risk Factor Collaboration (NCD-RisC). Rising rural body-mass index is the main driver of the global obesity epidemic in adults. *Nature* **569**, 260-264 (2019). https://doi.org/10.1038/s41586-019-1171-x

4 NCD Risk Factor Collaboration (NCD-RisC). Height and body-mass index trajectories of school-aged children and adolescents from 1985 to 2019 in 200 countries and territories: a pooled analysis of 2181 population-based studies with 65 million participants. *Lancet* **396**, 1511-1524 (2020). https://doi.org/10.1016/s0140-6736(20)31859-6

5 NCD Risk Factor Collaboration (NCD-RisC). Worldwide trends in body-mass index, underweight, overweight, and obesity from 1975 to 2016: a pooled analysis of 2416 population-based measurement studies in 128.9 million children, adolescents, and adults. *Lancet* **390**, 2627-2642 (2017). https://doi.org/10.1016/S0140-6736(17)32129-3

6 NCD Risk Factor Collaboration (NCD-RisC). Trends in adult body-mass index in 200 countries from 1975 to 2014: a pooled analysis of 1698 population-based measurement studies with 19.2 million participants. *Lancet* **387**, 1377-1396 (2016). https://doi.org/10.1016/s0140-6736(16)30054-x

7 NCD Risk Factor Collaboration (NCD-RisC). Worldwide trends in hypertension prevalence and progress in treatment and control from 1990 to 2019: a pooled analysis of 1201 population-representative studies with 104 million participants. *Lancet* **398**, 957-980 (2021). https://doi.org/10.1016/s0140-6736(21)01330-1

8 NCD Risk Factor Collaboration (NCD-RisC). Repositioning of the global epicentre of non-optimal cholesterol. *Nature* **582**, 73-77 (2020). https://doi.org/10.1038/s41586-020-2338-1
