# Supplementary material for: Obesity rise plateaus in developed nations and accelerates in developing nations
Source: Nature. 2026 May 13;653(8114):510–8. doi: 10.1038/s41586-026-10383-0 (PMC13171639; doi:10.1038/s41586-026-10383-0)
Supplement: Supplementary file 2 — Reporting Summary [file 41586_2026_10383_MOESM2_ESM.pdf]

Corresponding author(s): Majid Ezzati

Last updated by author(s): 28/01/26

Reporting Summary

Nature Portfolio wishes to improve the reproducibility of the work that we publish. This form provides structure for consistency and transparency in reporting. For further information on Nature Portfolio policies, see our Editorial Policies and the Editorial Policy Checklist.

Statistics

For all statistical analyses, confirm that the following items are present in the figure legend, table legend, main text, or Methods section.

n/a	Confirmed	
☐	☒	The exact sample size (*n*) for each experimental group/condition, given as a discrete number and unit of measurement
☒	☐	A statement on whether measurements were taken from distinct samples or whether the same sample was measured repeatedly
☒	☐	The statistical test(s) used AND whether they are one- or two-sided<br>*Only common tests should be described solely by name; describe more complex techniques in the Methods section.*
☒	☐	A description of all covariates tested
☒	☐	A description of any assumptions or corrections, such as tests of normality and adjustment for multiple comparisons
☐	☒	A full description of the statistical parameters including central tendency (e.g. means) or other basic estimates (e.g. regression coefficient) AND variation (e.g. standard deviation) or associated estimates of uncertainty (e.g. confidence intervals)
☒	☐	For null hypothesis testing, the test statistic (e.g. *F*, *t*, *r*) with confidence intervals, effect sizes, degrees of freedom and *P* value noted<br>*Give P values as exact values whenever suitable.*
☐	☒	For Bayesian analysis, information on the choice of priors and Markov chain Monte Carlo settings
☒	☐	For hierarchical and complex designs, identification of the appropriate level for tests and full reporting of outcomes
☒	☐	Estimates of effect sizes (e.g. Cohen's *d*, Pearson's *r*), indicating how they were calculated

Our web collection on statistics for biologists contains articles on many of the points above.

Software and code

Policy information about availability of computer code

Data collection	No software was used for secondary data collection.
Data analysis	All analyses were conducting using the statistical software R (version 4.3.0). We used R package ‘survey’ (version 4.4.2) and ‘rstan’ (version 2.26.15). The code for estimation of mean risk factor trends is available at www.ncdrisc.org and Zenodo (https://doi.org/10.5281/zenodo.18368827).

For manuscripts utilizing custom algorithms or software that are central to the research but not yet described in published literature, software must be made available to editors and reviewers. We strongly encourage code deposition in a community repository (e.g. GitHub). See the Nature Portfolio guidelines for submitting code & software for further information.

Data

Policy information about availability of data

All manuscripts must include a data availability statement. This statement should provide the following information, where applicable:

- Accession codes, unique identifiers, or web links for publicly available datasets
- A description of any restrictions on data availability
- For clinical datasets or third party data, please ensure that the statement adheres to our policy

Age-standardised and age-specific results of this study are available from www.ncdrisc.org in machine-readable numerical format and as visualization. Input data

from publicly available sources and contact information for data providers can be downloaded from www.ncdrisc.org and Zenodo (https://doi.org/10.5281/zenodo.18368827).

Research involving human participants, their data, or biological material

Policy information about studies with human participants or human data. See also policy information about sex, gender (identity/presentation), and sexual orientation and race, ethnicity and racism.

Reporting on sex and gender	N/A
Reporting on race, ethnicity, or other socially relevant groupings	N/A
Population characteristics	N/A
Recruitment	N/A
Ethics oversight	N/A

Note that full information on the approval of the study protocol must also be provided in the manuscript.

Field-specific reporting

Please select the one below that is the best fit for your research. If you are not sure, read the appropriate sections before making your selection.

☐ Life sciences ☒ Behavioural & social sciences ☐ Ecological, evolutionary & environmental sciences

For a reference copy of the document with all sections, see nature.com/documents/nr-reporting-summary-flat.pdf

Behavioural & social sciences study design

All studies must disclose on these points even when the disclosure is negative.

Study description	We pooled and re-analysed population-based data that had measured height and weight for children and adolescents and adults to estimate national trajectories in obesity from 1980 to 2024 for 200 countries and territories, using a Bayesian hierarchical model.
Research sample	We used 4,050 population-based studies that had measured height and weight in 232 million participants in 197 countries. Studies were representative of a national, subnational or community population. We used all available and accessible data which met the criteria described below.
Sampling strategy	This is a data pooling study which used all available and accessible data. These are population-based studies, each with sample size set to detect measure of interest in that study. These were pooled in a meta regression which provides more confidence in results by borrowing strength across studies. We included data collected using a probabilistic sampling method with a defined sampling frame. We therefore included studies with simple random and complex survey designs but excluded convenience samples.
Data collection	We used 4,050 population-based studies that had measured height and weight in 232 million participants in 197 countries. We used data on measured height and weight to calculate prevalence by sex and age group. We excluded self-reported data.
Timing	We pooled data collected from 1980 to 2024. We included national studies for the 3 years prior to start year, assigning them to the start year, and national studies for the year following the end year, assigning them to the end year, so that they can inform the estimates in countries with slightly earlier or more recent national data. We used all available data within these years which met the criteria described below.
Data exclusions	We excluded all data sources that were solely based on self-reported weight and height without a measurement component because these data are subject to biases that vary by geography, time, age, sex and socioeconomic characteristics. We also excluded data sources on population subgroups whose anthropometric status may differ systematically from the general population, including:<br>• studies that had included or excluded people based on their health status or cardiovascular risk;<br>• studies whose participants were only ethnic minorities;<br>• specific educational, occupational, or socioeconomic subgroups, with the exception noted below;<br>• those recruited through health facilities, with the exception noted below; and<br>• women aged 15-19 years in surveys which sampled only ever-married women or measured height and weight only among mothers.<br><br>We used school-based data in countries and age-sex groups with school enrolment of 70% or higher. We used data whose sampling frame was health insurance schemes in countries where at least 80% of the population were insured. Finally, we used data collected through general practice and primary care systems in high-income and central European countries with universal insurance, because contact with the primary care systems tends to be as good as or better than response rates for population-based surveys.
Non-participation	This was a secondary data analysis thus no participants were included in this study.

Randomization	Our study is an analysis of trends, and we did not carry out randomised experiments.

Reporting for specific materials, systems and methods

We require information from authors about some types of materials, experimental systems and methods used in many studies. Here, indicate whether each material, system or method listed is relevant to your study. If you are not sure if a list item applies to your research, read the appropriate section before selecting a response.

Materials & experimental systems

n/a	Involved in the study
☒	☐ Antibodies
☒	☐ Eukaryotic cell lines
☒	☐ Palaeontology and archaeology
☒	☐ Animals and other organisms
☒	☐ Clinical data
☒	☐ Dual use research of concern
☒	☐ Plants

Methods

n/a	Involved in the study
☒	☐ ChIP-seq
☒	☐ Flow cytometry
☒	☐ MRI-based neuroimaging

Plants

Seed stocks	N/A
Novel plant genotypes	N/A
Authentication	N/A
